# Direct Polymer-on-Polymer Grafting of Polyolefins under Visible Light

**DOI:** 10.1021/jacs.5c21265

**Published:** 2026-03-31

**Authors:** Hongsik Kim, Hyun Suk Wang, Namkyu Yun, Athina Anastasaki, Tae-Lim Choi

**Affiliations:** † Laboratory of Polymer Chemistry, Department of Materials, 27219 ETH Zurich, Zurich 8093, Switzerland; ‡ Laboratory of Sustainable Polymers, Department of Materials, 111950 ETH Zurich, Zurich 8093, Switzerland

## Abstract

Polyolefins dominate global plastic production but resist chemical transformation, leading to persistent waste accumulation. Developing new strategies that can repurpose these waste materials into higher-value products is therefore essential. Although conventional polar small-molecule grafting improves functionality, the resulting densely substituted polyolefins often become softer materials due to lower crystallinity and strength. Here, we report a visible-light-driven radical method that directly grafts diverse vinyl polymers onto polyolefins without the need for catalysts or initiators. The approach is broadly applicable to both pristine and postconsumer polyolefins on a multigram scale. Despite substantial functionalization that markedly increases polarity, the grafted polyolefins retain crystallinity, thermal stability, and mechanical robustness owing to their sparse yet extended polar polymer side chains. As a proof of concept, we demonstrate exceptional adhesion performance, with shear strengths approaching an order of magnitude higher than those of commercial hot-melt adhesives. This work establishes a general principle for polymer-on-polymer grafting of commodity plastics, expanding the conceptual space of polyolefin modification.

## Graphical abstract

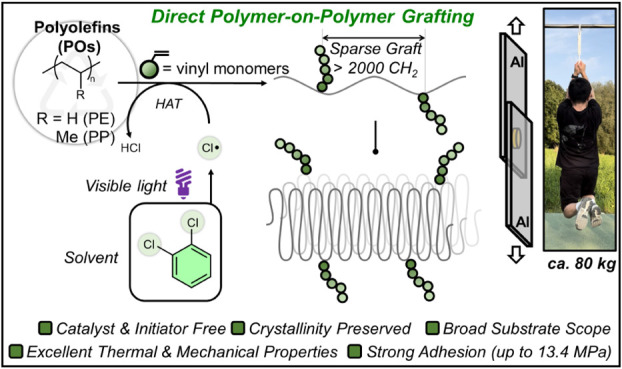

## Introduction

Polyolefins account for nearly 50% of the global plastics market, with annual production exceeding 300 million tons.^1,2^ The combination of low cost, chemical resistance, and robust mechanical performance has made them indispensable for packaging, consumer goods, and infrastructure.^3^ Yet these same virtues also make polyolefins among the most challenging materials to recycle or functionalize.^4–10^ As a result, the vast majority of postconsumer polyolefins are landfilled or incinerated, which contributes to persistent plastic accumulation wasting valuable hydrocarbon resources.^11–14^ Therefore, an ideal recycling strategy would be upgrading these materials into high-value products with new performance profiles.

Chemical upcycling offers one such route, with radical-mediated covalent grafting of polar small molecules onto otherwise inert polyolefin backbones (Figure 1a). This strategy can transform polyolefins into materials with tailored surface energy, adhesion, or compatibility with polar matrices, opening new opportunities in coatings, adhesives, and composites.^15–18^ Several recent ground-breaking advances illustrate its potential: nitroxide-based radical chain-transfer agents have been used to introduce diverse functional groups onto polyethylenes,^19–22^ transition metal-catalyzed radical relay C–H functionalization has incorporated various polar substituents,^23–27^ and iron­(III) chloride photocatalysis, via chlorine-radical-mediated hydrogen atom transfer, has enabled grafting of electron-deficient olefins.^28,29^ Despite their ingenuity, these approaches primarily attach large numbers of small polar fragments at high grafting density along the polymer backbone. Such dense substitution gives softer or less durable materials because it disrupts the crystalline domains leading to lower thermal and mechanical properties such as modulus and yield stress.^22,30^

**Figure 1:**
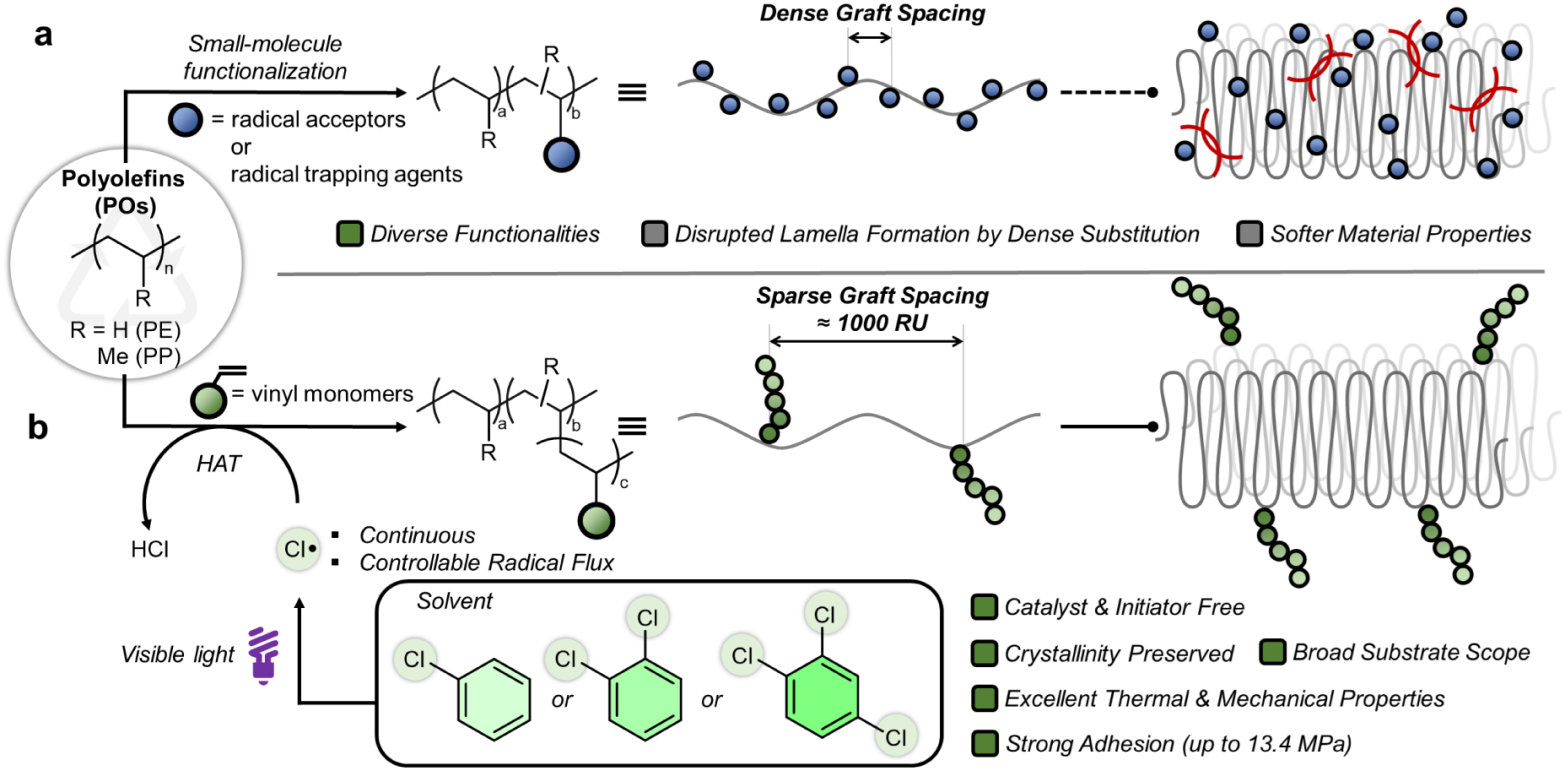
Schematic comparison of (a) the previous works: dense substitution in conventional methods disrupts crystallinity and softens materials; (b) the current work: sparse polymer grafting under visible light in chlorinated aromatic solvents preserves lamellar order yielding strong, crystalline polyolefin materials.

A promising alternative is to graft polar polymers (instead of small molecules) directly onto the polyolefin backbone with low grafting density. Such architecture would maintain high crystallinity and high incorporation of polar functionality (Figure 1b). Previously, well-defined model copolymers featuring this architecture were synthesized via bottom-up strategies, such as the ring-opening metathesis polymerization (ROMP) of functionalized cycloalkenes followed by hydrogenation.^31^ While these approaches provide precise structural control, they typically rely on multistep syntheses and specialized monomers. In the context of postpolymerization modification, however, this architecture remains largely underexplored, with two reports that elegantly demonstrated heterogeneous surface modification of polyolefins via organophotocatalysis.^32,33^ However, this method functionalizes only outer layers, leaving the vast majority of the polymers unmodified. Therefore, a general scalable bulk synthetic method for homogeneous polymer grafting onto pristine and postconsumer polyolefins is desirable for maximizing functionality leading to efficient upcycling into value-added materials.

Unlike the previous small-molecule grafting methods which often involve radical-quenching steps that are incompatible with graft-from polymerization, we envisioned that continuous, controlled radical generation could enable direct polymer growth from the polyolefin backbones.

Herein, we report the first general homogeneous method of grafting diverse vinyl polymers, including poly­(meth)­acrylates, polyacrylamides, polystyrene, poly­(vinylpyrrolidone) and poly­(vinyl acetate) onto polyolefins, without the need for designer radical precursors or metal catalysts. The photochemical approach is extremely practical (polymer, monomer and solvent in single-pot) but efficiently functionalizes both pristine and postconsumer polyolefins such as plastic bags, bottles, and laboratory tubes, with multigram scale while preserving or even enhancing the thermal and mechanical properties of the precursors. The resulting graft-polyolefins show greatly increased surface polarity and exceptional adhesion surpassing commercial hot-melt adhesives. We demonstrated that just 13 mg of the resulting adhesives bonded to metal strips was strong enough to support a male adult (ca. 80 kg), showcasing how the most inert and most produced plastics transformed into high-performance, value-added materials.

## Results and Discussion

Because polyolefins dissolve only in nonpolar solvents at elevated temperatures,^34^ grafting vinyl polymers is challenging as both backbone and growing chains are prone to chain scission under radical-rich conditions at such high temperature.^35^ In addition, excessive radical flux must be avoided to suppress unwanted side reactions and maintain controlled grafting. Therefore, two important criteria have to be satisfied simultaneously: sufficient solubility of polyolefins and controllable radical source. Inspired by the previous report on PMMA depolymerization,^36^ we selected a chlorinated solvent that both dissolves polyolefins and photochemically produces chlorine radicals capable of abstracting methylene hydrogens to initiate polymer growth. Importantly, the absorption onset of such solvents is red-shifted with increasing degree of chlorine substitution, which enhances the spectral overlap with LED light. Consequently, the radical concentration can be tuned by adjusting the solvent and light wavelength, allowing it to be maintained at a low level that effectively suppresses side reactions, such as degradation and cross-linking, thereby outperforming conventional initiators.^36^

With these hypotheses, we attempted to generate a moderate radical concentration by employing 1 M 1,2-dichlorobenzene (DCB) under 390 nm light to graft methyl acrylate (MA, 0.30 equiv) from low-density polyethylene (LDPE, *M*_w_ = 296 kDa) at 120 °C for 6 h (Figure 2a). After Soxhlet extraction to remove any residual PMA homopolymer, the grafted LDPE exhibited the molar fraction of vinyl repeat units (*f*_vinyl_) of 2.5% from ^1^H NMR analysis (MA conversion = 78%, entry 1 in Table 1). As intended, it showed a ∼3:1 ratio of MA methyl to carbonyl α-protons, consistent with PMA grafting rather than single monomer addition reported by others (a/b ratio in Figure 2b).^27,28^ 2D DOSY NMR revealed a single diffusion coefficient of 2.72 × 10^–7^ cm^2^ s^–1^ (Figure 2c), indicating that the two polymeric segments are indeed covalently linked rather than physically blended. Size-exclusion chromatography (SEC) confirmed the absence of cross-linking or chain scission (Figure 2d). Thus, this simple one-pot homogeneous protocol produced covalently linked polymer grafting, without damaging the polyolefin backbone.

**Figure 2:**
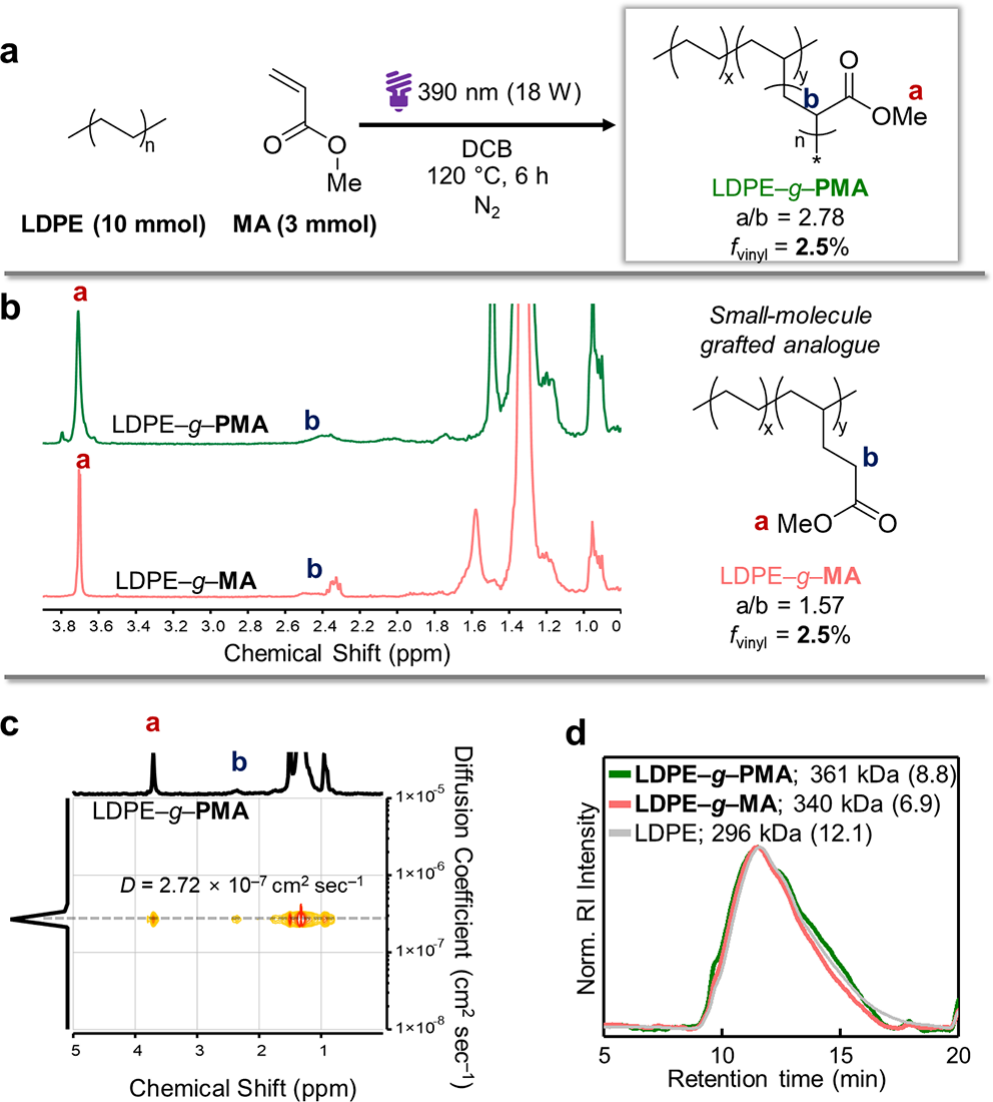
**Proof-of-concept synthesis of polymer-grafted LDPE**. (a) The initial reaction condition for **LDPE–** ***g*** **–PMA** (b) ^1^H NMR spectra of **LDPE–** ***g*** **–PMA** and **LDPE–** ***g*** **–MA**. (c) 2D DOSY NMR spectrum of LDPE–*g*–PMA. (d) SEC traces of **LDPE–** ***g*** **–PMA** (green), **LDPE–** ***g*** **–MA** (red), and LDPE (gray) (150 °C, TCB, polystyrene standard). The *M*_w_ and dispersity (in parentheses) are listed next to each legend entry.

**Table 1:**
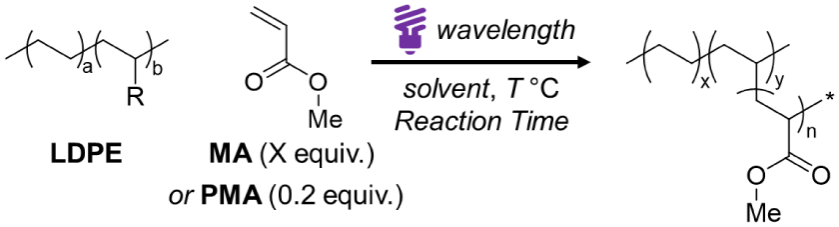
Reaction Optimization

Entry	λ (nm)	Substrate	Equiv.	Solvent	Temp. (°C)	Time (h)	Conv. (%)^a^	*f*_vinyl_ (%)^b^	Graft Ratio (%)^c^
1	390	MA	0.3	DCB	120	6	78	2.5	10.7
2	390	MA	0.2	DCB	120	6	67	2.4	17.9
3	390	MA	0.2	PhCl	120	6	7	*n.d.*	*n.a.*
4	390	MA	0.2	TCB	120	6	*n.a.*	*Gel*	*n.a.*
5	365	MA	0.2	PhCl	120	6	56	2.1	18.8
6	365	MA	0.2	DCB	120	6	*n.a.*	*Gel*	*n.a.*
7	425	MA	0.2	DCB	120	6	35	0.2	2.9
8	425	MA	0.2	TCB	120	6	49	0.5	5.1
9	390	MA	0.2	DCB	90	6	67	2.9	21.6
10	390	MA	0.2	DCB	150	6	80	1.5	9.4
11	390	MA	0.1	DCB	90	6	66	2.1	31.8
12	390	MA	0.3	DCB	90	6	71	3.9	18.3
13	390	MA	0.4	DCB	90	6	81	5.7	17.6
14^d^	390	MA	0.1	DCB	90	6	69	2.1	30.4
15^e^	390	MA	0.1	DCB	120	6	75	2.3	31.5
16	390	-	-	DCB	120	6	*n.a.*	*Gel*	*n.a.*
17	390	PMA	0.2	DCB	130	16	*n.a.*	1.3	6.5
18	390	MA	0.2	DCB	130	16	>99	5.2	26.3
19	390	MAH	0.2	DCB	90	6	20%	<0.2	*n.a.*

a Determined by ^1^H NMR using polyethylene as the internal standard.

b Determined by ^1^H NMR.

c Determined by comparing ^1^H NMR spectra before and after Soxhlet extraction.

d 2 M concentration.

e 4 M concentration. The product was isolated via simple precipitation in dichloromethane without Soxhlet extraction, yielding purity essentially identical to the Soxhlet-extracted sample.

To elucidate how side-chain microstructures (monomeric vs polymeric) influence material properties, we synthesized a small-molecule-grafted analogue (**LDPE–** ***g*** **–MA**, Figure 2b) under the reported conditions, in which the propagating radical is quenched by iron­(II) immediately after monomer addition, thereby closing the catalytic cycle and preventing further polymerization.^28^

First, ^1^H NMR showed distinct signals at 2.3–2.4 ppm with lower integration ratio (3:1.89 vs 3:1.08 for **LDPE–** ***g*** **–PMA**), suggesting structural differences (Figure 2b). We then compared their thermal properties by DSC using two copolymers with similar *f*_vinyl_s (∼2.5%).

Interestingly, **LDPE–*g*–PMA** showed a higher melting temperature (*T*_m_ = 106.9 °C) and crystallinity (*X*_c_ = 26.2%), closer to pristine LDPE (109.8 °C, 32.3%), compared to **LDPE–** ***g*** **–MA** (98.6 °C, 20.2%) (Figure 3a). This trend was corroborated by wide-angle X-ray scattering (WAXS) analysis, which yielded crystallinity values following the same order. Furthermore, the diffraction patterns revealed similar crystalline lamella structures across all samples, consistent with the grafted substituents primarily residing in the amorphous region (Figure 3b). On the other hand, although small-angle X-ray scattering (SAXS) showed similarly long periods (11–12 nm) for all samples, **LDPE–** ***g*** **–MA** did display a noticeably weaker lamellar peak even after accounting for film-thickness differences, suggesting a reduced lamellar population in line with its lower DSC crystallinity (Figure 3c). Atomic force microscopy further supported the superior order of **LDPE–** ***g*** **–PMA**, implying larger crystalline domains than **LDPE–** ***g*** **–MA** and no detectable macro-phase separation. In contrast, a physical PMA/LDPE blend exhibited clear separation (Figures S23–S26). Consistently, tensile test of **LDPE–** ***g*** **–PMA** displayed higher modulus and yield stress (*E* = 89 ± 8 MPa; ε_*y*_ = 5.4 ± 0.1 MPa) than **LDPE–** ***g*** **–MA** (50 ± 2 MPa; 4.6 ± 0.1 MPa; Figure 3d). Overall, the polymer grafting sample showed higher crystallinity, as well as improved thermal and mechanical properties compared to that from the monomeric addition.

**Figure 3:**
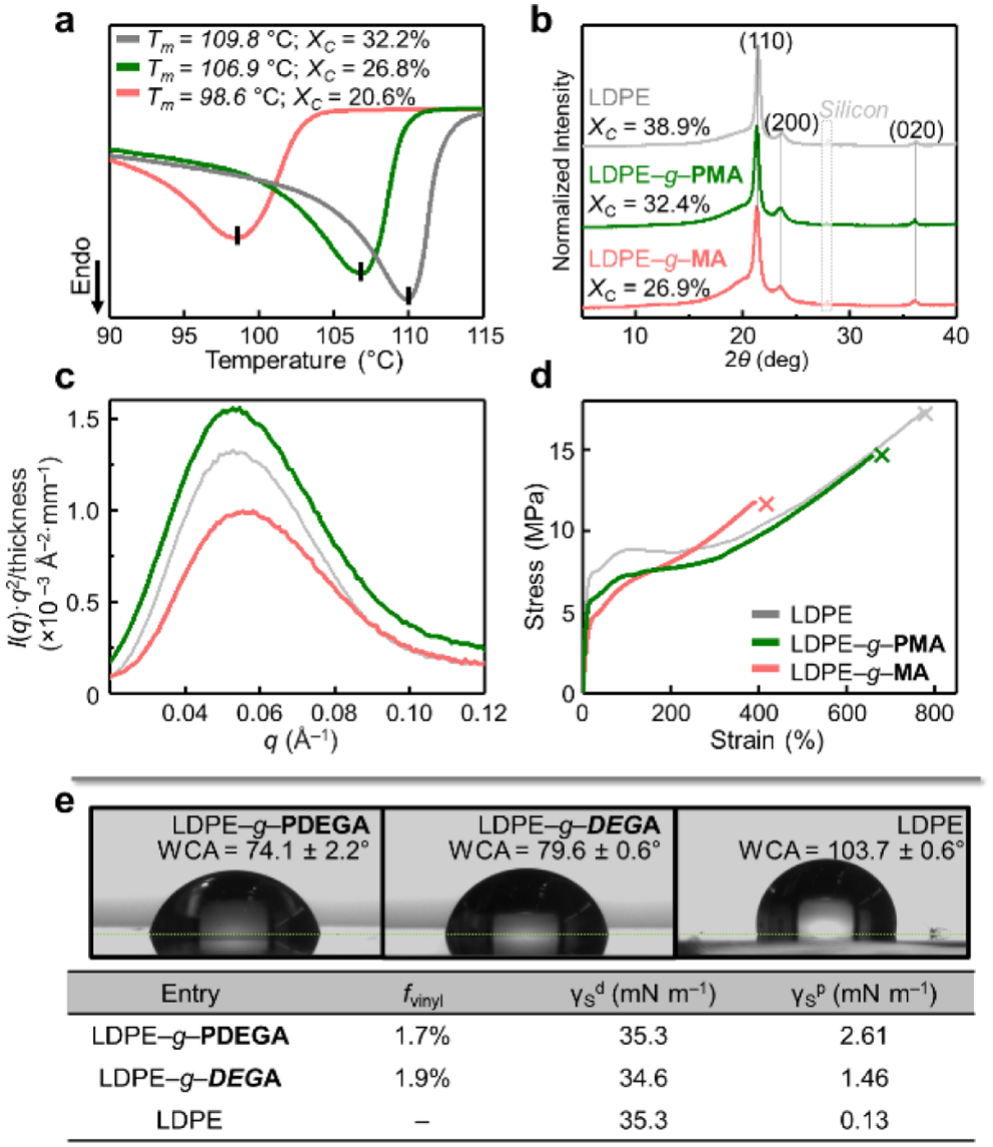
**Comparison between monomer- and polymer-grafted LDPEs.** (a) DSC thermograms. (b) X-ray diffraction patterns. (c) SAXS profiles normalized by sample thickness. (d) Stress–strain curves. (e) Water contact angle measurements for **LDPE–** ***g*** **–PDEGA**, **LDPE–** ***g*** **–DEGA**, and LDPE. (f) A table summarizes the corresponding surface energies, where γ_S_^d^ and γ_S_^p^ denote the dispersive and polar components, respectively. For a–d, green, red, and gray represent **LDPE–** ***g*** **–PMA**, **LDPE–** ***g*** **–MA**, and **LDPE**, respectively.

Introducing polar functionality to polyolefins gives new materials with diverse applications due to enhanced interfacial interactions and adhesion.^24^ Therefore, we grafted a polar monomer, di­(ethylene glycol) ethyl ether acrylate (DEGA), by the two methods for comparison (Figure S27). Similar to MA, polymer-grafted **LDPE–** ***g*** **–PDEGA** showed superior thermal and mechanical properties compared to the monomer-grafted analogue (Figures S27 and S28). Notably, **LDPE–** ***g*** **–PDEGA** displayed greater polarity with a lower water contact angle (WCA = 74.1 ± 2.2°) and higher polar surface energy (γ_s_^p^ = 2.61 mJ m^–2^) than monomer-grafted **LDPE–** ***g*** **–DEGA** (79.6 ± 0.6°, 1.49 mJ m^–2^) and the parent LDPE (103.7 ± 0.6°, 0.13 mJ m^–2^; Figures 3e and S29–S31). These results validate that polymer-grafting introduces polar functionality effectively while preserving the crystallinity and strength of the parent polyolefin.

Although polymeric grafting enhanced the thermal and mechanical properties, a moderate *f*_vinyl_ was obtained and a considerable amount of PMA homopolymer was also formed, suggesting that a portion of the monomer underwent free homopolymerization under the current conditions. These observations highlight the importance of controlling radical flux to favor graft propagation over homopolymerization. We, therefore, sought to optimize the radical concentration—a key factor governing the reaction pathway—by systematically varying the solvent chlorination level and irradiation wavelength (Table 1).

First, reducing the monomer feed ratio under the same reaction condition did not lower the *f*_vinyl_, but it improved the relative extent of grafting compared to free PMA formation (grafting ratio) from 11% to 18% (entry 2). Changing solvent from DCB to chlorobenzene (PhCl) under 390 nm light yielded very low monomer conversion and *f*_vinyl_, suggesting insufficient radical flux (entry 3). In contrast, changing to a more chlorinated solvent, 1,2,4-trichlorobenzene (TCB), generated excessive radicals, resulting in gelation within 6 h (entry 4). By changing the light source to 365 nm LED, PhCl provided a moderate conversion (56%) and *f*_vinyl_ (2.1%), while DCB again led to cross-linking (entries 5–6). On the other hand, with 425 nm LED, both DCB and TCB produced low conversions (and *f*_vinyl_s)—35% (0.2%) and 49% (0.5%), respectively (entries 7–8).

Temperature screening further revealed that lowering the reaction temperature from 120 to 90 °C increased both the *f*_vinyl_ and the degree of grafting (entry 2 vs 9), but elevating it to 150 °C reduced both by approximately 40% (entry 2 vs 10), presumably due to enhanced PMA chain scission at higher temperatures.

Collectively, these results identify 390 nm irradiation in DCB at 90 °C as the optimal condition, providing a balanced radical flux that achieves reasonable conversion and a moderate grafting level while avoiding undesired cross-linking. Although radical flux was not directly quantified, the observed trends in *f*_vinyl_ and gelation correlate well with the expected radical concentration derived from solvent absorption profiles (Figure S32). This control over the radical flux would provide a general guideline for tuning graft density. Under the optimized condition (390 nm, DCB, 90 °C; entry 9), we next examined how monomer feed ratio and reaction components influence the grafting mechanism (entries 11–18).

At a lower feed (0.1 equiv., entry 11), the *f*_vinyl_ remained moderate, but the degree of grafting reached to its highest level of 31.8% (entry 11), indicating that limited monomer availability effectively suppresses homopolymerization and enhances grafting efficiency. On the other hand, increasing the monomer feed to 0.3 and 0.4 equiv. decreased the graft ratio to approximately 18%, but led to higher *f*_vinyl_s (up to 5.7%, entry 12 and 13). Changing the polymer concentration had minimal influence, as increasing the concentration to 2 M (entry 14) or 4 M (11.2 wt %, at 120 °C, entry 15) produced nearly identical *f*_vinyl_ and grafting trends. To make the process more practical, the product could be purified by simple dichloromethane precipitation without Soxhlet extraction because even after the extraction, the values were essentially the same for *f*_vinyl_ (2.30% to 2.27%) and recovered mass (1.15 to 1.14 g).

Control experiments further provided mechanistic insights on the radical process. In the absence of monomer (entry 16), the polymer underwent gelation, consistent with radical generation along the polyolefin backbone and subsequent radical–radical couplings. Under the standard reaction condition, however, these backbone radicals are consumed by *grafting-from* polymerization of MA (Figure 4a) or by secondary hydrogen-atom transfer (HAT) with PMA, which helps to prevent extensive cross-linking. More interestingly, when presynthesized PMA was introduced instead of MA, an *f*_vinyl_ of 1.3% (graft ratio = 6.5%) and a single diffusion coefficient were observed by ^1^H and 2D DOSY NMR (entry 17 in Table 1 and Figure S33). These observations are consistent with PMA chains undergoing partial scission where the resulting fragments (having either radicals or activated alkenes) are incorporated into the LDPE backbone through radical–radical coupling or radical addition (*grafting-to*, Figure 4a), in line with the mechanism recently proposed for the one-pot HDPE–*g*–*i*PP synthesis by Coates group.^37^

**Figure 4:**
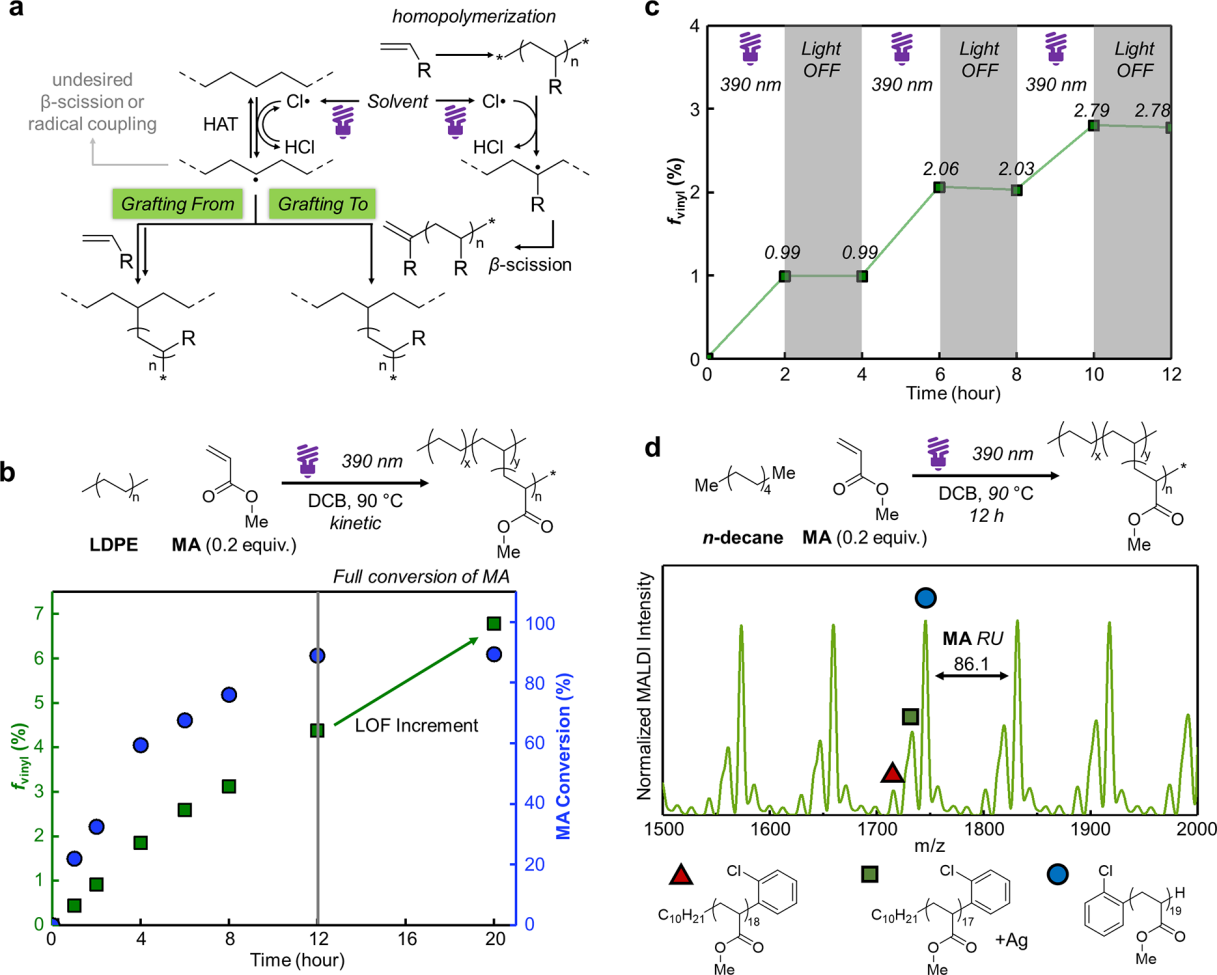
(**a**) Proposed mechanism. (**b**) Kinetic study. (**c**) Temporal control of LDPE functionalization with on–off light cycles. (**d**) Small molecule studies for structural characterization via MALDI-TOF.

Incorporation of commercial PMMA under the identical conditions further supported this pathway (*f*_vinyl_ = 1.3%, Figure S34). In contrast, using MA at this condition gave a much higher *f*_vinyl_ and graft ratio (5.2 and 26.3%, respectively, entry 18; Figures S35 and S36) implying a larger contribution of the grafting-from polymerization.

To investigate further on the mechanism, we, first, conducted kinetic analysis by monitoring the monomer conversion and the *f*_vinyl_ by ^1^H NMR. Interestingly, *f*_vinyl_ continued to increase from 4.5 to 6.7% even after the monomer reached its maximum conversion of 90% after 12 h (Figure 4b). These further suggest that the grafting-from is more prominent at the early stages of monomer conversion, while the grafting-to pathway may contribute more at the later stage especially once the full conversion is reached.

The use of a nonpolymerizable analogue, maleic anhydride (MAH), led to negligible grafting (<0.2% *f*_vinyl_, entry 19), consistent with the intrinsically low grafting density targeted in this chemistry and our design principle of sparse polymer grafting. Finally, periodic on/off light experiments confirmed the photochemical nature of the transformation, as *f*_vinyl_ increased only during the irradiation (Figure 4c).

To investigate structural features of grafting, we conducted model studies using linear decane as a surrogate for polyethylenes because broad dispersity and unresolved NMR signals of LDPE hindered direct quantification. Although decane does not fully capture the steric and topological complexity of polyolefins, it provides a simplified system to probe the fundamental grafting mechanism under the identical radical conditions. By treating MA with decane at either 90 and 120 °C, PMA homopolymer and **decane–** ***g*** **–PMA** were obtained altogether, while a control without decane gave PMA only (Figures S37 and S38). Both cases showed similar SEC traces with *M*_n_ = 3.82 kDa (*Đ* = 2.67) at 90 °C and *M*_n_ = 1.60 kDa (*Đ* = 1.86) at 120 °C. This lower *M*_n_ at higher temperature is likely due to more chain scission. Matrix-assisted laser desorption/ionization time-of-flight (MALDI-TOF) mass spectrometry revealed two distinct sets of peaks: DCB-derived end groups (for degree of polymerization (DP) = 19, calc. *m*/*z* = 1746.9; found = 1746.4) corresponding to PMA homopolymer, and *n*-decane-derived end groups (for DP = 18, calc. *m*/*z* = 1714.8; found = 1715.1) corresponding to **decane–** ***g*** **–PMA** (Figures 4d and S39). Notably, the *M*_n_ of the grafted and free PMA populations were comparable, enabling us to approximate the length of grafted chains (DP = 40–50 at 90 °C). Combining these values with the *f*_vinyl_, the grafting density was estimated at ca. 0.08% under optimal conditions (ca. 1 chain per 1,250 repeat units). At 120 °C, shorter grafts (DP = 15–20) were formed, but the calculated density doubled (ca. 0.16% or 1 chain per 630 repeat units), allowing for the tunable graft chain length and density by temperature. Most notably, the density remains intrinsically sparse, consistent with the extremely low *f*_vinyl_ (<0.2%) observed in the MAH model reaction (Table 1, entry 18), which is the key to preserving polyolefin crystallinity.

Building on these results, we expanded the scope to other monomers (Figure 5a). For methyl methacrylate (MMA), *f*_vinyl_ was strongly temperature-dependent: at 90 °C, 0.2 equiv. MMA gave 5.5%, whereas at 120 °C, it dropped to 0.2%, consistent with its reported polymerization–depolymerization equilibrium (Figure S40).^36^ Increasing the feed to 0.8 equiv. at 90 °C further improved the *f*_vinyl_ to 11.7% (Figure S41). Various polar acrylamides such as *N*-isopropylacrylamide (NIPAM), *N*-*n*-propylacrylamide (NNPAM), and *N*-ethylacrylamide (NEtAM) also produced graft copolymers with *f*_vinyl_s of 4–7%. Notably, *N*,*N*-dimethylacrylamide (NDMAM) yielded the highest *f*_vinyl_ of 10.5% with 0.2 equiv monomer feed ratio, indicating particularly efficient graft formation. Unlike the relatively less polar samples, high-temperature SEC traces of these polar-group grafted materials showed longer retention times (Figure S42), presumably due to reduced hydrodynamic volume from intramolecular interaction among the polar side chains rather than backbone degradation. To further support this interpretation, hydrophobic *N*-decylacrylamide (NDecAM) was prepared and showed the expected molecular weight increase over time (Figure S43). Reasonable *f*_vinyl_s were also obtained with less reactive monomers—vinyl acetate (2.6%), *N*-vinylpyrrolidone (3.0%), styrene (3.7%), and even halogenated styrenes (1.7% for 4-chlorostyrene and 0.9% for 4-bromostyrene)—where intact C–Cl/Br bonds were confirmed by ^1^H NMR (Figure S44), indicating some tolerance to halogen substituents and potential for further postfunctionalization via cross-coupling chemistry.

**Figure 5:**
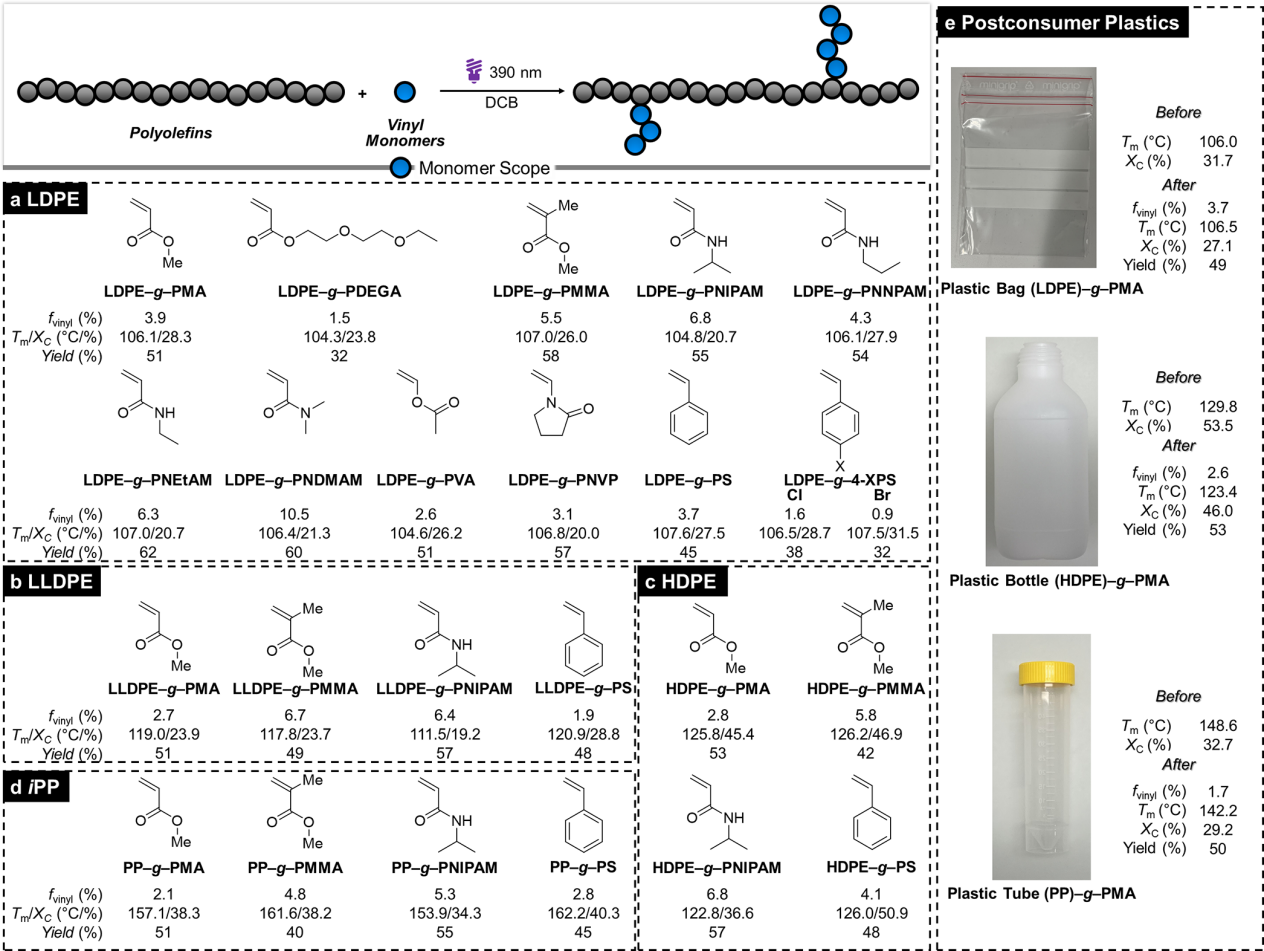
**Reaction scope** on (**a**) LDPE, (**b**) LLDPE, (**c**) HDPE, (**d**) *i*PP, and (**e**) postconsumer plastics. The yield was calculated as the ratio of the product mass to the total mass of the polyolefin and monomer used.

To further broaden the scope, we also tried other classes of polyolefins including linear low-density polyethylene (LLDPE), high-density polyethylene (HDPE), and isotactic polypropylene (*i*PP) with four representative monomers (MA, MMA, styrene, and NIPAM; Figure 5b–d). Because of their lower solubility compared to LDPE, elevated reaction temperatures were employed for these polyolefins (105 °C for LLDPE and 120 °C for HDPE and PP). Overall, comparable *f*_vinyl_s were observed with these polymers, highlighting the excellent versatility of the method. In particular, for polypropylene, conventional high-temperature radical conditions often led to chain scission and significant molecular-weight reduction, resulting in downcycling.^38^ However, no major molecular-weight loss was detected under our conditions, highlighting the mild radical flux achieved by this approach (Figure S45).

However, such sparse grafting raises a potential concern regarding the presence of unfunctionalized polyolefin chains. To address this point, we combined a Poisson model with the experimentally measured high-temperature SEC distribution to obtain the estimated grafted and ungrafted mass contributions across the trace (Figure S46). This distribution-resolved analysis indicates that the ungrafted contribution may be as low as ∼5% and is confined to the low-molecular-weight PE, while the majority of PE contains at least one graft. Notably, grafting the LLDPE of low dispersity with *n*-butyl methacrylate (**LLDPE–** ***g*** **–PBuMA)** showed the strongest support as SEC traces completely shifted uniformly across the entire distribution upon the grafting (Figure S47). Therefore, the material is best described as a predominantly covalently grafted copolymer rather than a physical blend.

We next evaluated whether this chemistry is applicable to postconsumer plastic wastes using a zip-lock plastic bag, a plastic bottle, and a Falcon tube to represent LDPE, HDPE, and PP waste, respectively. (Figure 5e). LDPE from the plastic bag was reacted with MA (0.3 equiv) at 90 °C, yielding an *f*_vinyl_ of 3.7%, comparable to the result obtained from reagent-grade LDPE. Similarly, reactions with postconsumer HDPE and PP yielded comparable or slightly lower *f*_vinyl_ values than those obtained with reagent-grade polyolefins. These results demonstrate great compatibility of the grafting chemistry toward plastic wastes even though they contain various additives such as radical scavengers, antioxidants, and plasticizers.

Thermal analysis of LDPE samples showed that *T*_m_s were largely retained, with decreases of <6 °C relative to pristine LDPE (Figure 5 and Table S1). The largest drop was for **LDPE–** ***g*** **–PDEGA** (104.3 °C vs 109.8 °C), attributed to the polar and sterically bulky side-chain, whereas even high-*f*_vinyl_ monomers such as MMA (5.5%), NEtAM (6.3%), and NDMAM (10.5%) resulted in smaller decreases of 2.8–3.4 °C, consistent with preserved crystalline domains. Melting enthalpies (Δ*H*_m_) decreased by 10–30 J/g (Table S1), but since Δ*H*_m_ is calculated per unit mass, amorphous side chains lowered apparent values and underestimated crystallinity.^39,40^ Accordingly, normalization using *f*_vinyl_-derived polyethylene mass fractions showed that crystallinity was largely maintained (mostly over 30%, Table S1), with the lowest value, 25.3% for **LDPE–** ***g*** **–PNEtAM**, still comparable to pristine LDPE (32.3%). Similar trends showing excellent thermal properties were observed for other polyolefins, while bulky polar NIPAM consistently gave the largest reductions in both *T*_m_ and Δ*H*_m_. Especially, new materials with high crystallinity up to 51% (or 59% after normalization) were obtained from HDPE precursors, including postconsumer plastics (Figure 5c). Thus, DSC analyses indicate that this polymer-grafting maintains the thermal robustness of polyolefin while introducing new functionalities, thereby opening new opportunities to tailor polyolefin properties.

To assess practical utility, we investigated the mechanical properties of grafted polyolefins. As observed with **LDPE–** ***g*** **–PMA**_**2.5**_ (subscript denotes *f*_vinyl_ %), grafting with soft polymers reduced strength relative to pristine LDPE (*E* = 145 ± 3 MPa; σ_*y*_ = 7.1 ± 0.04 MPa; Figure 3d). By contrast, grafting with mechanically robust or hydrogen-bonding polymers such as PMMA, PS, and PNIPAM yielded materials with higher elastic moduli and yield stresses while retaining considerable ductility (ε_break_ > 100%). For example, **LDPE–** ***g*** **–PMMA**_**5.5**_, **LDPE–** ***g*** **–PS**_**3.7**_, and **LDPE–** ***g*** **–PNIPAM**_**6.8**_ showed high elastic moduli of 256 ± 13, 163 ± 5, and 246 ± 13 MPa and high yield stresses of 10.3 ± 0.1, 8.0 ± 0.4, and 11.7 ± 0.3 MPa, respectively (Figures 6a and S48–S50). To examine the effect of steric bulk on acrylamides, we compared grafting with smaller NNPAM and NEtAM.

**Figure 6:**
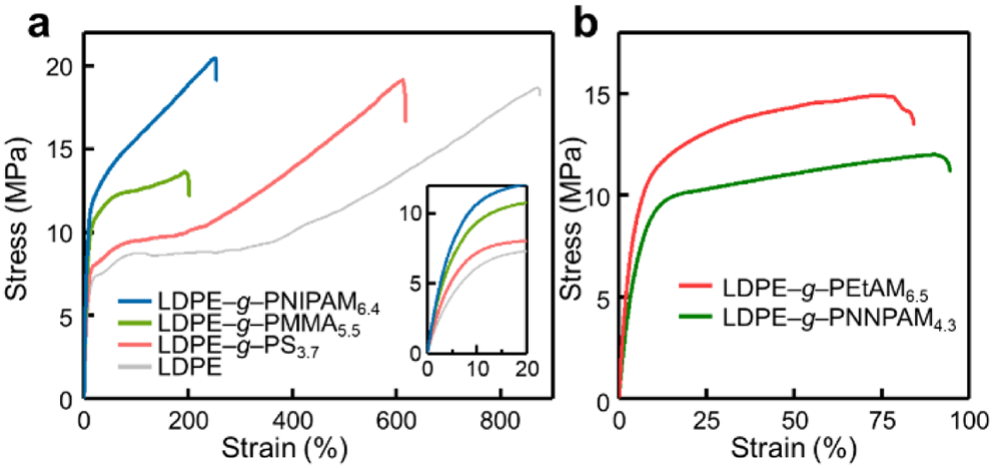
**Mechanical performance** (**a**) Tensile stress–strain curves of LDPE grafted with various vinyl polymers. Inset: magnified view of the initial elongation region. (**b**) Tensile stress–strain curves of **LDPE–** ***g*** **–poly­(** ***N*** **-alkylacrylamide)** samples.

The resulting **LDPE–** ***g*** **–PNNPAM**_**4.3**_ and **LDPE–** ***g*** **–PNEtAM**_**6.5**_ exhibited high elastic moduli and yield strengths (*E* = 232 ± 38 and 312 ± 9 MPa; σ_*y*_ = 10.3 ± 0.4 and 11.4 ± 0.2 MPa, respectively, Figures 6b and S51–S52). While the *f*_vinyl_s differ, making direct comparison not straightforward, these data suggest that smaller *N*-substituents may promote reinforcement, presumably through stronger hydrogen bonding. Similar trends were observed for LLDPE, HDPE, and *i*PP substrates (Figures S53–S55). However, these gains came at the expense of ductility, likely arising from graft-induced morphological microphase separation between the nonpolar backbone and the highly polar grafts, as evidenced by AFM and scanning transmission electron microscopy (STEM, Figure S56).

Because metal, wood, and glass surfaces present carbonyl and hydroxyl groups, the polar grafted materials were expected to form hydrogen-bonding interactions and display strong adhesion (Figure 7a).^41^ We therefore evaluated their interfacial adhesion using lap-shear tests on aluminum, a substrate with high yield strength and polar surface. Indeed, **LDPE–** ***g*** **–PMMA**_**5.5,**_ **LDPE–** ***g*** **–PNIPAM**_**3.8**_, **LDPE–** ***g*** **–PNNPAM**_**4.3**_, and **LDPE–** ***g*** **–PNEtAM**_**6.5**_ displayed strong adhesion with shear strengths of 5.2 ± 0.4, 6.0 ± 1.1, 9.8 ± 1.2, and 12.7 ± 0.6 (up to 13.4) MPa, respectively, after curing 1 h at 190 °C (Figure 7b, Tables S2–S4) while pristine LDPE with highly nonpolar surface could not even be processed into specimens. Remarkably, these values are close to the corresponding yield stresses of the bulk materials, which are often considered the theoretical limit of shear strength.^42^ This indicates that adhesion at the metal–polymer interface surpasses the intrinsic cohesive strength of the polymer matrix, supported by cohesive failures for samples showing higher shear strength than yield stress (Table S2). Notably, all four grafted samples far outperformed benchmark hot-melt adhesives, including commercial poly­(ethylene-*co*-vinyl acetate) (EVA, 1.59 ± 0.01 MPa) and Gorilla Glue hot melt adhesive (2.63 ± 0.3 MPa). The best-performing sample, **LDPE–** ***g*** **–PNEtAM**_**6.5**_ also showed strong adhesion to stainless steel (8.8 ± 2.1 MPa), copper (10.9 ± 0.8 MPa), and even wood (10.2 ± 0.8 MPa). Glass substrates prepared from standard microscope slides, fractured even before interfacial debonding, indicating that the adhesion strength exceeded the fracture resistance of the slide geometry under our test conditions. To further probe practical robustness, a brief water-immersion (10 min) test was conducted, revealing that the film of **LDPE–** ***g*** **–PNEtAM**_**6.5**_ retained ∼88% of its dry lap-shear strength (Table S4).

**Figure 7:**
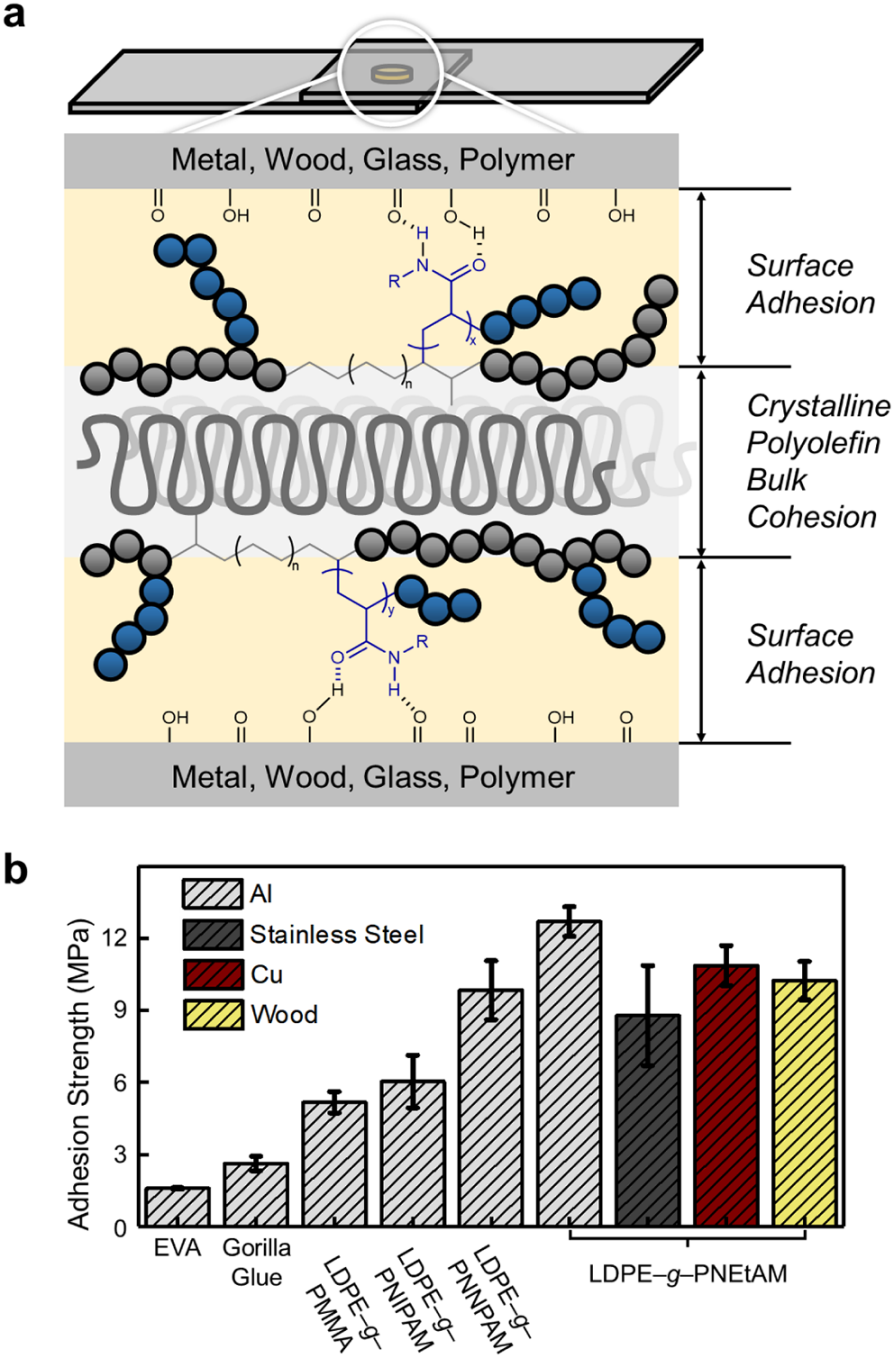
(a) Schematic illustration of the proposed adhesion mechanism. (b) Lap-shear test results comparing the adhesion strengths of the prepared samples.

Finally, we evaluated scalability by conducting a 10-gram-scale synthesis of **LDPE–** ***g*** **–PNEtAM** using 11.2 g of LDPE (400 mmol) and 0.2 equiv. NEtAM (Figure 8a). The reaction afforded 13.5 g of grafted polymer with an *f*_vinyl_ of 6.8% (isolated yield = 70%), demonstrating practical scalability. This outcome is facilitated by the low molar absorptivity of DCB (ε_390 nm_ = 4.5 × 10^–3^ L mol^–1^ cm^–1^) at 390 nm,^35^ which enables deep light penetration, and by its high refractive index, which enhances total internal reflection thereby minimizing the light loss. To test its adhesion, just 13 mg of this material (circular specimen: 0.5 cm radius, 0.67 mm thickness) was glued to bond two aluminum plates and this successfully lifted a 20 kg kettlebell (Figure 8b and Movie S1). Even more surprisingly, a parallel specimen supported the body weight of an adult (the first author, ∼80 kg) during hanging and pull-ups motions (Figure 8b and Movie S2). These results highlight both the scalability of the method and the exceptional adhesion strength of the resulting materials.

**Figure 8:**
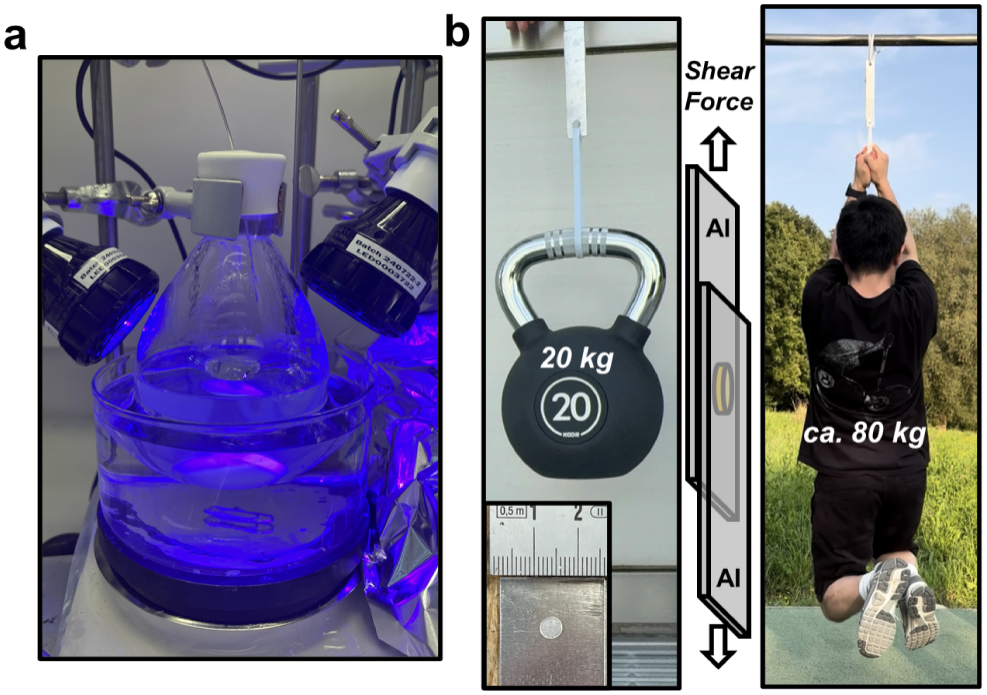
(a) Photograph of the reaction setup used for the large-scale synthesis. (b) Adhesion demonstration of large-scale **LDPE–** ***g*** **–PNEtAM**. Left: a 20 kg kettlebell lifted using a 13 mg **LDPE–** ***g*** **–PNEtAM** adhesive sample (diameter 0.5 cm, thickness 0.67 mm; inset). Right: the adhesive supported the weight of an adult male (approximately 80 kg) during a hanging and pull-up test.

## Conclusions

Taken together, we establish a general and scalable synthetic platform for homogeneous bulk polymer grafting of polyolefins that requires only a monomer, a polyolefin, and a solvent, without catalysts, additives, or monomer purification. The method is broadly applicable to diverse vinyl monomers and multiple polyolefin substrates, including postconsumer plastics, and is readily scalable to multigram quantities. Several grafted polymers showed enhanced modulus and yield strength compared to their parent materials. Most notably, LDPE grafted with poly­(*N*-alkylacrylamides) achieved shear strengths up to 13.4 MPa on metal surfaces, surpassing commercial hot-melt adhesives. Looking ahead, this approach offers a practical platform for transforming the most recalcitrant commodity plastics into high-performance functional materials. Beyond adhesives, tailoring graft composition and density could open up pathways to compatibilizers, barrier films, or responsive materials, advancing both the recycling of plastics and the design of next-generation polymers.

## Supplementary Material







## Notes

(MP4)
(MP4)
Materials; Methods; Figures; Tables (PDF)
