# Supplementary material for: Direct Polymer-on-Polymer Grafting of Polyolefins under Visible Light
Source: J Am Chem Soc. 2026 Mar 31;148(14):14924–33. doi: 10.1021/jacs.5c21265 (PMC13088238; doi:10.1021/jacs.5c21265)
Supplement: Supplementary file 3 [file ja5c21265_si_003.pdf]

Supporting Information

Direct polymer-on-polymer grafting of polyolefins under visible light

Hongsik Kim[1], Hyun Suk Wang[2], Namkyu Yun[1], Athina Anastasaki[2], Tae-Lim Choi[1*]

[1]Laboratory of Polymer Chemistry, Department of Materials, ETH Zurich, Vladimir-Prelog-Weg 5, 8093 Zurich, Switzerland

[2]Laboratory of Sustainable Polymers, Department of Materials, ETH Zurich, Vladimir-Prelog-Weg 5, 8093 Zurich, Switzerland

Corresponding author: Tae-Lim Choi, tae-lim.choi@mat.ethz.ch

Table of Contents

Materials 11
Methods........ 11
Supplementary Figures 14
Supplementary Tables........ 56
Reaction Procedures and Material Characterization........ 58
References........ 149

Table of Supplementary Tables

Table S1. Summary of melting enthalpies. 56
Table S2. Summary of lap shear test. Curing time = 1 hour. Temperature = 190 °C................... 57
Table S3. Results of curing time screening for LDPE–*g*–PNEtAM$_{6.3}$. Temperature = 190 °C ... 57
Table S4. Results of curing temperature screening for LDPE–*g*–PNEtAM$_{6.3}$. Curing Time = 60 min 57

Table of Supplementary Figures

Figure S1. Emission spectrum of the 365 nm LED lamp (HepatoChem, EvoluChem365PF EU, 190 mW cm^{-2})........ 14
Figure S2. Emission spectrum of the 390 nm LED lamp (HepatoChem, EvoluChem390PF EU, 294 mW cm^{-2})........ 14
Figure S3. Emission spectrum of the 425 nm LED lamp (HepatoChem, EvoluChem390PF EU, 166 mW cm^{-2})........ 15
Figure S4. Schematic depiction of the general reaction set up........ 15
Figure S5. HT SEC trace of LDPE (1,2,4-TCB, 1.0 mL min^{-1} at 150 °C)........ 16
Figure S6. SEC trace of LLDPE (1,2,4-TCB, 1.0 mL min^{-1} at 150 °C)........ 16
Figure S7. HT SEC trace of HDPE (1,2,4-TCB, 1.0 mL min^{-1} at 150 °C)........ 17
Figure S8. HT SEC trace of *i*PP (1,2,4-TCB, 1.0 mL min^{-1} at 150 °C)........ 17
Figure S9. HT SEC trace of a plastic bag (1,2,4-TCB, 1.0 mL min^{-1} at 150 °C)........ 18
Figure S10. HT SEC trace of a plastic bottle (1,2,4-TCB, 1.0 mL min^{-1} at 150 °C)........ 18
Figure S11. HT SEC trace of a falcon tube (1,2,4-TCB, 1.0 mL min^{-1} at 150 °C)........ 19
Figure S12. DSC Thermogram of LDPE used in this study. T_m = 109.83 °C, ΔH_m = 94.532 J g^{-1}, X_C = 32.3%........ 20
Figure S13. DSC Thermogram of LLDPE used in this study. T_m = 122.10 °C, ΔH_m = 88.084 J g^{-1}, X_C = 30.1%........ 20
Figure S14. DSC Thermogram of HDPE used in this study. T_m = 129.58 °C, ΔH_m = 153.61 J g^{-1}, X_C = 52.4%........ 21
Figure S15. DSC Thermogram of *i*PP used in this study. T_m = 163.41 °C, ΔH_m = 98.554 J g^{-1}, X_C = 47.6%........ 21
Figure S16. DSC Thermogram of a plastic bag used in this study. T_m = 106.01°C, ΔH_m = 92.945 J g^{-1}, X_C = 31.7%........ 22
Figure S17. DSC Thermogram of a plastic bottle used in this study. T_m = 129.75°C, ΔH_m = 156.89 J g^{-1}, X_C = 53.5%........ 22
Figure S18. DSC Thermogram of a falcon tube used in this study. T_m = 148.63 °C, ΔH_m = 67.75 J g^{-1}, X_C = 32.7%........ 23
Figure S19. Water contact angle measured for LDPE used in this study. WCA = 103.7°........ 24
Figure S20. Water contact angle measured for LLDPE used in this study. WCA = 100.0°........ 24
Figure S21. Water contact angle measured for HDPE used in this study. WCA = 93.6°........ 25
Figure S22. Water contact angle measured for *i*PP used in this study. WCA = 105.3°........ 25
Figure S23. AFM height (top) and phase (bottom) images of LDPE........ 26
Figure S24. AFM height (top) and phase (bottom) images of LDPE–*g*–PMA (Fig. 2)........ 26
Figure S25. AFM height (top) and phase (bottom) images of LDPE–*g*–MA (Fig. 2)........ 27
Figure S26. AFM height (top) and phase (bottom) images of LDPE/PMA (16.4 wt% of PMA) blend........ 27
Figure S27. Synthesis and characterization of LDPE–*g*–PDEGA and LDPE–*g*–DEGA........ 28
Figure S28. (a) Stress–strain curves of LDPE–*g*–PDEGA (green), LDPE–*g*–DEGA (red), and LDPE (gray). (b) Summary of mechanical tests........ 29
Figure S29. Contact angle analysis of LDPE........ 30
Figure S30. Contact angle analysis of LDPE–*g*–PDEGA........ 30
Figure S31. Contact angle analysis of LDPE–*g*–DEGA........ 31
Figure S32. Overlay of Solvent UV–vis absorption spectra and emission spectra of UV lamp........ 31

Figure S33. (A) Reaction scheme of the PMA grafting experiment (Fig. 3A, entry 11). (B) ^{1}H NMR spectrum (400 MHz, $C_2D_2Cl_4$). (C) 2D DOSY NMR spectrum.. 32
Figure S33. (A) Reaction scheme of the PMMA grafting experiment. (B) ^{1}H NMR spectrum (400 MHz, $C_2D_2Cl_4$). (C) 2D DOSY NMR spectrum. 33
Figure S35. ^{1}H NMR spectrum (400 MHz, $C_2D_2Cl_4$) of LDPE–*g*–PMA (Fig. 3A entry 12)....... 34
Figure S36. HT SEC trace of LDPE–*g*–PMA (Fig. 3A, entry 12, 1,2,4-TCB, 1.0 mL min^{-1} at 150 °C) 35
Figure S37. (A) Reaction scheme of the decane small-molecule study at 90 °C. (B) Control experiment in the absence of decane. (C) SEC traces of the two reactions (THF, 1.0 mL min^{-1}, 35 °C). 36
Figure S38. (A) Reaction scheme of the decane small-molecule study at 90 °C. (B) Control experiment in the absence of decane. (C) SEC traces of the two reactions (THF, 1.0 mL min^{-1}, 35 °C). 37
Figure S39. Full MALDI-TOF MS spectrum of a mixture of PMA homopolymer (highlighted in blue) and decane–g–PMA (highlighted in red). Notably, the two major species exhibit similar molar mass distributions. 38
Figure S40. ^{1}H NMR spectrum of LDPE–*g*–PMMA synthesized at 120 °C with 0.2 equiv. of MMA... 39
Figure S41. ^{1}H NMR and 2D DOSY NMR spectrum of LDPE–*g*–PMMA synthesized at 90 °C with 0.8 equiv. of MMA. 40
Figure S42. HT SEC traces of polar-polymer grafted LDPEs.. 41
Figure S43. Kinetic HT SEC study using NDecAM as a representative non-polar acrylamide (1,2,4-TCB, 1.0 mL min^{-1} at 150 °C). Unlike polar-polymer-grafted LDPEs, NDecAM exhibited an increase in molecular weight over time. Given its structural similarity to other acrylamides, this result supports that the lower apparent molecular weights of the polar samples originate from changes in hydrodynamic radius rather than from severe chain scission. 42
Figure S44. ^{1}H NMR (400 MHz, $C_2D_2Cl_4$) spectra of LDPE–*g*–P(4-BrS) (top), LDPE–*g*–P(4-ClS) (middle), and LDPE–*g*–PS (bottom). (T = 90 °C)... 43
Figure S45. HT SEC traces of grafted *i*PP samples (1,2,4-TCB, 1.0 mL min^{-1} at 150 °C) 44
Figure S46. Poisson-distribution-based analysis of graft statistics. (a) Probability of a polymer chain containing at least one graft as a function of degree of polymerization (DP), calculated using a Poisson model at different grafting densities (d_{graft}). (b,c) Corresponding fraction of chains containing at least one graft plotted as a function of retention time, obtained by mapping DP to molecular weight via column calibration for (b) PE and (c) PP. (d–h) High-temperature SEC traces of LDPE, LLDPE, HDPE, PP, and toluene-Soxhlet-purified LLDPE (black), overlaid with simulated grafted (solid) and ungrafted (dotted) contributions calculated by weighting the experimental RI signal with the Poisson-derived probability of at least one graft. Light-colored traces correspond to a graft density of 0.08%, whereas dark-colored traces correspond to a graft density of 0.15%. Importantly, the statistically ungrafted contribution is confined to the low-molecular-weight (high-retention-time) tail of the molecular weight distribution, whereas the high-molecular-weight region is overwhelmingly dominated by chains expected to contain at least one graft. (i) Summary of the expected mass fraction of polymer chains containing at least one graft, showing a maximum value of 94% for purified LLDPE at a graft density of 0.15%. . 45
Figure S47. Experimental verification of the statistical grafting model using LLDPE. (a) Reaction scheme for the visible-light-mediated grafting of PMMA or PBuMA from LLDPE pellets (or toluene-Soxhlet-purified LLDPE). (b) High-temperature SEC traces of LLDPE before

(gray) and after (black) grafting with PMMA after 12 h of reaction. (c) High-temperature SEC traces of toluene-Soxhlet-purified LLDPE before (gray) and after (black) grafting with PBuMA after 16 h of reaction. 46
Figure S48. Stress–strain curves for LDPE–*g*–$PMMA_{5.5}$ 47
Figure S49. Stress–strain curves for LDPE–*g*–$PS_{3.7}$ 48
Figure S50. Stress–strain curves for LDPE–*g*–$PNIPAM_{6.8}$ 49
Figure S51. Stress–strain curves for LDPE–*g*–$PNNPAM_{4.3}$ 50
Figure S52. Stress-strain curves for LDPE–*g*–$PNEtAM_{6.5}$ 51
Figure S53. (a) Enlarged view and (b) full Stress–strain curves of reinforced LLDPE samples . 52
Figure S54. (a) Enlarged view and (b) full Stress–strain curves of reinforced HDPE samples ... 53
Figure S55. (a) Enlarged view and (b) full Stress–strain curves of reinforced *i*PP samples 54
Figure S56. (a–f) Transmission electron microscopy (TEM) and energy-dispersive X-ray spectroscopy (EDX) analyses of LDPE–*g*–PNEtAM. (a) Bright-field TEM image. (b) Nitrogen elemental EDX map. (c) Oxygen elemental EDX map. (d) Overlay of the TEM image and the N EDX map. (e) Overlay of the TEM image and the O EDX map. (f) Overlay of the TEM image with N and O EDX maps. (g–i) Phase-mode AFM images at different magnifications. 55
Figure S57. ^{1}H NMR spectrum (400 MHz, $C_2D_2Cl_4$) of LDPE–*g*–$PMA_{2.5}$ (T = 90 °C) 58
Figure S58. 2D DOSY NMR spectrum (400 MHz, $C_2D_2Cl_4$) of LDPE–*g*–$PMA_{2.5}$ (T = 90 °C) . 59
Figure S59. DSC Thermogram of LDPE–*g*–PMA (f_{vinyl} = 2.5%). T_m = 106.91 °C, ΔH_m = 76.737 J g^{-1}, X_C = 26.2%. 59
Figure S60. ^{1}H NMR spectrum (400 MHz, $C_2D_2Cl_4$) of LDPE–*g*–$PDEGA_{1.6}$ (T = 90 °C) 60
Figure S61. 2D DOSY NMR spectrum (400 MHz, $C_2D_2Cl_4$) of LDPE–*g*–$PDEGA_{1.6}$ (T = 90 °C) 61
Figure S62. DSC Thermogram of LDPE–*g*–PDEGA (f_{vinyl} = 1.6%). T_m = 106.44 °C, ΔH_m = 77.440 J g^{-1}, X_C = 26.4%. 61
Figure S63. ^{1}H NMR spectrum (400 MHz, $C_2D_2Cl_4$) of LDPE–*g*–$MA_{2.5}$ (T = 90 °C) 62
Figure S64. DSC Thermogram of LDPE–*g*–MA (f_{vinyl} = 2.5%). T_m = 98.56 °C, ΔH_m = 60.341 J g^{-1}, X_C = 20.6%. 63
Figure S65. ^{1}H NMR spectrum (400 MHz, $C_2D_2Cl_4$) of LDPE–*g*–$DEGA_{1.9}$ (T = 90 °C) 64
Figure S66. DSC Thermogram of LDPE–*g*–DEGA (f_{vinyl} = 2.5%). T_m = 101.38 °C, ΔH_m = 61.940 J g^{-1}, X_C = 21.1%. 65
Figure S67. ^{1}H NMR spectrum (400 MHz, $C_2D_2Cl_4$) of LDPE–*g*–$PMA_{3.9}$ (T = 90 °C) 66
Figure S68. 2D DOSY NMR spectrum (400 MHz, $C_2D_2Cl_4$) of LDPE–*g*–$PMA_{3.9}$ (T = 90 °C) . 67
Figure S69. HT SEC trace of LDPE–*g*–$PMA_{3.9}$ (1,2,4-TCB, 1.0 mL min^{-1} at 150 °C) 67
Figure S70. DSC Thermogram of LDPE–*g*–PMA (f_{vinyl} = 3.9%). T_m = 106.06 °C, ΔH_m = 82.842 J g^{-1}, X_C = 28.3%. 68
Figure S71. Water contact angle measured for LDPE–*g*–PMA (f_{vinyl} = 3.9%). WCA = 90.4° 68
Figure S72. ^{1}H NMR spectrum (400 MHz, $C_2D_2Cl_4$) of LDPE–*g*–$PDEGA_{1.5}$ (T = 90 °C) 69
Figure S73. 2D DOSY NMR spectrum (400 MHz, $C_2D_2Cl_4$) of LDPE–*g*–$PDEGA_{1.5}$ (T = 90 °C) 70
Figure S74. HT SEC trace of LDPE–*g*–$PDEGA_{1.5}$ (1,2,4-TCB, 1.0 mL min^{-1} at 150 °C) 70
Figure S75. DSC Thermogram of LDPE–*g*–PDEGA (f_{vinyl} = 1.5%). T_m = 104.26 °C, ΔH_m = 69.815 J g^{-1}, X_C = 23.8%. 71
Figure S76. Water contact angle measured for LDPE–*g*–PDEGA (f_{vinyl} = 1.5%). WCA = 80.8° 71
Figure S77. ^{1}H NMR spectrum (400 MHz, $C_2D_2Cl_4$) of LDPE–*g*–$PMMA_{5.5}$ (T = 90 °C) 72

Figure S78. 2D DOSY NMR spectrum (400 MHz, $C_2D_2Cl_4$) of LDPE–*g*–$PMMA_{5.3}$ (T = 90 °C) 73
Figure S79. HT SEC trace of LDPE–*g*–$PMMA_{5.5}$ (1,2,4-TCB, 1.0 mL min^{-1} at 150 °C) 73
Figure S80. DSC Thermogram of LDPE–*g*–PMMA (f_{vinyl} = 5.3%). T_m = 107.0 °C, ΔH_m = 76.299 J g^{-1}, X_C = 26.0%. 74
Figure S81. Water contact angle measured for LDPE–*g*–PMMA (f_{vinyl} = 5.5%). WCA = 97.4° . 74
Figure S82. ^{1}H NMR spectrum (400 MHz, $C_2D_2Cl_4$) of LDPE–*g*–$PNIPAM_{6.8}$ (T = 90 °C) 75
Figure S83. 2D DOSY NMR spectrum (400 MHz, $C_2D_2Cl_4$) of LDPE–*g*–$PNIPAM_{6.8}$ (T = 90 °C) 76
Figure S84. HT SEC trace of LDPE–*g*–$PNIPAM_{6.8}$ (1,2,4-TCB, 1.0 mL min^{-1} at 150 °C) 76
Figure S85. DSC Thermogram of LDPE–*g*–PNIPAM (f_{vinyl} = 6.8%). T_m = 104.8 °C, ΔH_m = 60.709 J g^{-1}, X_C = 20.7%. 77
Figure S86. Water contact angle measured for LDPE–*g*–PNIPAM (f_{vinyl} = 6.8%). WCA = 87.3° 77
Figure S87. ^{1}H NMR spectrum (400 MHz, $C_2D_2Cl_4$) of LDPE–*g*–$PNNPAM_{4.3}$ (T = 90 °C) 78
Figure S88. 2D DOSY NMR spectrum (400 MHz, $C_2D_2Cl_4$) of LDPE–*g*–$PNNPAM_{4.3}$ (T = 90 °C) 79
Figure S89. HT SEC trace of LDPE–*g*–$PNNPAM_{4.3}$ (1,2,4-TCB, 1.0 mL min^{-1} at 150 °C)....... 79
Figure S90. DSC Thermogram of LDPE–*g*–PNNPAM (f_{vinyl} = 4.3%). T_m = 106.1 °C, ΔH_m = 81.66 J g^{-1}, X_C = 27.9%. 80
Figure S91. Water contact angle measured for LDPE–*g*–PNNPAM (f_{vinyl} = 4.3%). WCA = 96.3° 80
Figure S92. ^{1}H NMR spectrum (400 MHz, $C_2D_2Cl_4$) of LDPE–*g*–$PNEtAM_{6.3}$ (T = 90 °C) 81
Figure S93. 2D DOSY NMR spectrum (400 MHz, $C_2D_2Cl_4$) of LDPE–*g*–$PNEtAM_{6.3}$ (T = 90 °C) 82
Figure S94. HT SEC trace of LDPE–*g*–$PNEtAM_{6.3}$ (1,2,4-TCB, 1.0 mL min^{-1} at 150 °C) 82
Figure S95. DSC Thermogram of LDPE–*g*–PNEtPAM (f_{vinyl} = 6.3%). T_m = 107.04 °C, ΔH_m = 60.712 J g^{-1}, X_C = 20.7%. 83
Figure S96. Water contact angle measured for LDPE–*g*–PNEtAM (f_{vinyl} = 4.3%). WCA = 95.5° 83
Figure S97. ^{1}H NMR spectrum (400 MHz, $C_2D_2Cl_4$) of LDPE–*g*–$PNDMAM_{10.5}$ (T = 90 °C) ... 84
Figure S98. 2D DOSY NMR spectrum (400 MHz, $C_2D_2Cl_4$) of LDPE–*g*–$PNDMAM_{10.5}$ (T = 90 °C) 85
Figure S99. HT SEC trace of LDPE–*g*–$PNDMAM_{10.5}$ (1,2,4-TCB, 1.0 mL min^{-1} at 150 °C).... 85
Figure S100. DSC Thermogram of LDPE–*g*–PNDMAM (f_{vinyl} = 10.5%). T_m = 106.44 °C, ΔH_m = 62.275 J g^{-1}, X_C = 21.3%. 86
Figure S101. Water contact angle measured for LDPE–*g*–PNDMAM (f_{vinyl} = 10.5%). WCA = 88.0° 86
Figure S102. ^{1}H NMR spectrum (400 MHz, $C_2D_2Cl_4$) of LDPE–*g*–$PVA_{2.6}$ (T = 90 °C)............ 87
Figure S103. 2D DOSY NMR spectrum (400 MHz, $C_2D_2Cl_4$) of LDPE–*g*–$PVA_{2.6}$ (T = 90 °C) 88
Figure S104. HT SEC trace of LDPE–*g*–$PVA_{2.6}$ (1,2,4-TCB, 1.0 mL min^{-1} at 150 °C) 88
Figure S105. DSC Thermogram of LDPE–*g*–PVA (f_{vinyl} = 2.6%). T_m = 104.59 °C, ΔH_m = 76.713 J g^{-1}, X_C = 26.2%. 89
Figure S106. Water contact angle measured for LDPE–*g*–PVA (f_{vinyl} = 2.6%). WCA = 87.7° ... 89
Figure S107. ^{1}H NMR spectrum (400 MHz, $C_2D_2Cl_4$) of LDPE–*g*–$PVP_{3.1}$ (T = 90 °C) 90
Figure S108. 2D DOSY NMR spectrum (400 MHz, $C_2D_2Cl_4$) of LDPE–*g*–$PVP_{3.1}$ (T = 90 °C). 91

Figure S109. HT SEC trace of LDPE–*g*–$PVP_{3.1}$ (1,2,4-TCB, 1.0 mL min^{-1} at 150 °C) 91
Figure S110. DSC Thermogram of LDPE–*g*–PVP (f_{vinyl} = 3.1%). T_m = 106.82 °C, ΔH_m = 78.685 J g^{-1}, X_C = 26.9%. 92
Figure S111. Water contact angle measured for LDPE–*g*–PVP (f_{vinyl} = 3.1%). WCA = 83.4° 92
Figure S112. ^{1}H NMR spectrum (400 MHz, $C_2D_2Cl_4$) of LDPE–*g*–$PS_{3.7}$ (T = 90 °C) 93
Figure S113. 2D DOSY NMR spectrum (400 MHz, $C_2D_2Cl_4$) of LDPE–*g*–$PS_{3.7}$ (T = 90 °C).... 94
Figure S114. HT SEC trace of LDPE–*g*–$PS_{3.7}$ (1,2,4-TCB, 1.0 mL min^{-1} at 150 °C) 94
Figure S115. DSC Thermogram of LDPE–*g*–PS (f_{vinyl} = 3.7%). T_m = 107.56 °C, ΔH_m = 80.706 J g^{-1}, X_C = 27.5%. 95
Figure S116. Water contact angle measured for LDPE–*g*–PS (f_{vinyl} = 3.7%). WCA =103.0° 95
Figure S117. ^{1}H NMR spectrum (400 MHz, $C_2D_2Cl_4$) of LDPE–*g*–P(4-ClS)$_{1.6}$ (T = 90 °C) 96
Figure S118. 2D DOSY NMR spectrum (400 MHz, $C_2D_2Cl_4$) of LDPE–*g*–P(4-ClS)$_{1.6}$ (T = 90 °C) 97
Figure S119. HT SEC trace of LDPE–*g*–P(4-ClS)$_{1.6}$ (1,2,4-TCB, 1.0 mL min^{-1} at 150 °C) 97
Figure S120. DSC Thermogram of LDPE–*g*–P(4-ClS) (f_{vinyl} = 1.6%). T_m = 107.71 °C, ΔH_m = 87.02 J g^{-1}, X_C = 29.1%. 98
Figure S121. Water contact angle measured for LDPE–*g*–P(4-ClS) (f_{vinyl} = 1.6%). WCA = 104.3° 98
Figure S122. ^{1}H NMR spectrum (400 MHz, $C_2D_2Cl_4$) of LDPE–*g*–P(4-BrS)$_{0.9}$ (T = 90 °C) 99
Figure S123. 2D DOSY NMR spectrum (400 MHz, $C_2D_2Cl_4$) of LDPE–*g*–P(4-BrS)$_{0.9}$ (T = 90 °C) 100
Figure S124. HT SEC trace of LDPE–*g*–P(4-BrS)$_{0.9}$ (1,2,4-TCB, 1.0 mL min^{-1} at 150 °C)..... 100
Figure S125. DSC Thermogram of LDPE–*g*–P(4-BrS) (f_{vinyl} = 0.9%). T_m = 108.75 °C, ΔH_m = 92.45 J g^{-1}, X_C = 31.4%. 101
Figure S126. ^{1}H NMR spectrum (400 MHz, $C_2D_2Cl_4$) of LLDPE–*g*–$PMA_{2.7}$ (T = 105 °C) 102
Figure S127. 2D DOSY NMR spectrum (400 MHz, $C_2D_2Cl_4$) of LLDPE–*g*–$PMA_{2.7}$ (T = 105 °C) 103
Figure S128. HT SEC trace of LLDPE–*g*–PMA (1,2,4-TCB, 1.0 mL min^{-1} at 150 °C)............ 103
Figure S129. DSC Thermogram of LLDPE–*g*–PMA (f_{vinyl} = 2.7%). T_m = 119.00 °C, ΔH_m = 70.123 J g^{-1}, X_C = 23.9%. 104
Figure S130. Water contact angle measured for LLDPE–*g*–PMA (f_{vinyl} = 2.7%). WCA = 94.2° 104
Figure S131. ^{1}H NMR spectrum (400 MHz, $C_2D_2Cl_4$) of LLDPE–*g*–$PMMA_{6.7}$ (T = 105 °C).. 105
Figure S132. 2D DOSY NMR spectrum (400 MHz, $C_2D_2Cl_4$) of LLDPE–*g*–$PMMA_{6.7}$ (T = 105 °C) 106
Figure S133. HT SEC trace of LLDPE–*g*–PMMA (1,2,4-TCB, 1.0 mL min^{-1} at 150 °C) 106
Figure S134. DSC Thermogram of LLDPE–*g*–PMMA (f_{vinyl} = 6.7%). T_m = 117.82 °C, ΔH_m = 64.487 J g^{-1}, X_C = 23.7%. 107
Figure S135. Water contact angle measured for LLDPE–*g*–PMMA (f_{vinyl} = 6.7%). WCA = 100.0° 107
Figure S136. ^{1}H NMR spectrum (400 MHz, $C_2D_2Cl_4$) of LLDPE–*g*–$PNIPAM_{6.4}$ (T = 105 °C) 108
Figure S137. 2D DOSY NMR spectrum (400 MHz, $C_2D_2Cl_4$) of LLDPE–*g*–$PNIPAM_{6.4}$ (T = 105 °C) 109
Figure S138. HT SEC trace of LLDPE–*g*–PNIPAM (1,2,4-TCB, 1.0 mL min^{-1} at 150 °C)..... 109
Figure S139. DSC Thermogram of LLDPE–*g*–PNIPAM (f_{vinyl} = 6.4%). T_m = 111.45 °C, ΔH_m = 56.161 J g^{-1}, X_C = 19.2%. 110

Figure S140. Water contact angle measured for LLDPE–*g*–PNIPAM ($\boldsymbol{f}_{\mathbf{vinyl}}$ = 6.4%). WCA = 82.1° 110
Figure S141. ^{1}H NMR spectrum (400 MHz, $C_2D_2Cl_4$) of LLDPE–*g*–$PS_{1.9}$ (T = 105 °C) 111
Figure S142. 2D DOSY NMR spectrum (400 MHz, $C_2D_2Cl_4$) of LLDPE–*g*–$PS_{1.9}$ (T = 105 °C) 112
Figure S143. HT SEC trace of LLDPE–*g*–PS (1,2,4-TCB, 1.0 mL min^{-1} at 150 °C) 112
Figure S144. DSC Thermogram of LLDPE–*g*–PS ($\boldsymbol{f}_{\mathbf{vinyl}}$ = 1.9%). T_m = 120.93 °C, ΔH_m = 84.513 J g^{-1}, X_C = 28.8%. 113
Figure S145. Water contact angle measured for LLDPE–*g*–PS ($\boldsymbol{f}_{\mathbf{vinyl}}$ = 1.9%). WCA = 103.0° 113
Figure S146 ^{1}H NMR spectrum (400 MHz, $C_2D_2Cl_4$) of HDPE–*g*–$PMA_{2.8}$ (T = 105 °C) 114
Figure S147. 2D DOSY NMR spectrum (400 MHz, $C_2D_2Cl_4$) of HDPE–*g*–$PMA_{2.8}$ (T = 105 °C) 115
Figure S148. HT SEC trace of HDPE–*g*–PMA (1,2,4-TCB, 1.0 mL min^{-1} at 150 °C) 115
Figure S149. DSC Thermogram of HDPE–*g*–PMA ($\boldsymbol{f}_{\mathbf{vinyl}}$ = 2.8%). T_m = 125.81 °C, ΔH_m = 132.77 J g^{-1}, X_C = 45.4%. 116
Figure S150. Water contact angle measured for HDPE–*g*–PMA ($\boldsymbol{f}_{\mathbf{vinyl}}$ = 2.8%). WCA = 85.9° 116
Figure S151. ^{1}H NMR spectrum (400 MHz, $C_2D_2Cl_4$) of HDPE–*g*–$PMMA_{5.8}$ (T = 105 °C) 117
Figure S152. 2D DOSY NMR spectrum (400 MHz, $C_2D_2Cl_4$) of HDPE–*g*–$PMMA_{5.8}$ (T = 105 °C) 118
Figure S153. HT SEC trace of HDPE–*g*–PMA (1,2,4-TCB, 1.0 mL min^{-1} at 150 °C) 118
Figure S154. DSC Thermogram of HDPE–*g*–PMMA ($\boldsymbol{f}_{\mathbf{vinyl}}$ = 5.8%). T_m = 126.18 °C, ΔH_m = 137.52 J g^{-1}, X_C = 46.9%. 119
Figure S155. Water contact angle measured for HDPE–*g*–PMMA ($\boldsymbol{f}_{\mathbf{vinyl}}$ = 5.8%). WCA = 99.4° 119
Figure S156. ^{1}H NMR spectrum (400 MHz, $C_2D_2Cl_4$) of HDPE–*g*–$PNIPAM_{6.4}$ (T = 105 °C) . 120
Figure S157. 2D DOSY NMR spectrum (400 MHz, $C_2D_2Cl_4$) of HDPE–*g*–$PNIPAM_{6.4}$ (T = 105 °C) 121
Figure S158. HT SEC trace of HDPE–*g*–PNIPAM (1,2,4-TCB, 1.0 mL min^{-1} at 150 °C) 121
Figure S159. DSC Thermogram of HDPE–*g*–PNIPAM ($\boldsymbol{f}_{\mathbf{vinyl}}$ = 6.4%). T_m = 122.83 °C, ΔH_m = 107.23 J g^{-1}, X_C = 36.6%. 122
Figure S160. Water contact angle measured for HDPE–*g*–PNIPAM ($\boldsymbol{f}_{\mathbf{vinyl}}$ = 6.4%). WCA = 83.2° 122
Figure S161. ^{1}H NMR spectrum (400 MHz, $C_2D_2Cl_4$) of HDPE–*g*–$PS_{4.1}$ (T = 105 °C) 123
Figure S162. 2D DOSY NMR spectrum (400 MHz, $C_2D_2Cl_4$) of HDPE–*g*–$PS_{4.1}$ (T = 105 °C) 124
Figure S163. HT SEC trace of HDPE–*g*–PS (1,2,4-TCB, 1.0 mL min^{-1} at 150 °C) 124
Figure S164. DSC Thermogram of HDPE–*g*–PS ($\boldsymbol{f}_{\mathbf{vinyl}}$ = 4.1%). T_m = 125.99 °C, ΔH_m = 149.17 J g^{-1}, X_C = 50.9%. 125
Figure S165. Water contact angle measured for HDPE–*g*–PS ($\boldsymbol{f}_{\mathbf{vinyl}}$ = 4.1%). WCA = 97.8° 125
Figure S166. ^{1}H NMR spectrum (400 MHz, $C_2D_2Cl_4$) of *i*PP–*g*–$PMA_{2.1}$ (T = 105 °C) 126
Figure S167. 2D DOSY NMR spectrum (400 MHz, $C_2D_2Cl_4$) of *i*PP–*g*–$PMA_{2.1}$ (T = 105 °C) 127
Figure S168. HT SEC trace of *i*PP–*g*–PMA (1,2,4-TCB, 1.0 mL min^{-1} at 150 °C) 127
Figure S169. DSC Thermogram of *i*PP–*g*–PMA ($\boldsymbol{f}_{\mathbf{vinyl}}$ = 2.1%). T_m = 157.13 °C, ΔH_m = 79.242 J g^{-1}, X_C = 38.3%. 128
Figure S170. Water contact angle measured for *i*PP–*g*–PMA ($\boldsymbol{f}_{\mathbf{vinyl}}$ = 2.1%). WCA = 101.9° ... 128
Figure S171. ^{1}H NMR spectrum (400 MHz, $C_2D_2Cl_4$) of *i*PP–*g*–$PMMA_{4.8}$ (T = 105 °C) 129

Figure S172. 2D DOSY NMR spectrum (400 MHz, $C_2D_2Cl_4$) of *i*PP–*g*–PMMA$_{4.8}$ (T = 105 °C). 130
Figure S173. HT SEC trace of *i*PP–*g*–PMMA (1,2,4-TCB, 1.0 mL min^{-1} at 150 °C) 130
Figure S174. DSC Thermogram of *i*PP–*g*–PMMA (f_{vinyl} = 4.8%). T_m = 161.63 °C, ΔH_m = 78.988 J g^{-1}, X_C = 38.2%. 131
Figure S175. Water contact angle measured for *i*PP–*g*–PMMA (f_{vinyl} = 4.8%). WCA = 104.3° 131
Figure S176. ^{1}H NMR spectrum (400 MHz, $C_2D_2Cl_4$) of *i*PP–*g*–PNIPAM$_{5.3}$ (T = 105 °C) 132
Figure S177. 2D DOSY NMR spectrum (400 MHz, $C_2D_2Cl_4$) of PP–*g*–PNIPAM$_{5.3}$ (T = 105 °C) 133
Figure S178. HT SEC trace of *i*PP–*g*–PNIPAM$_{5.3}$ (1,2,4-TCB, 1.0 mL min^{-1} at 150 °C) 133
Figure S179. DSC Thermogram of *i*PP–*g*–PNIPAM (f_{vinyl} = 5.3%). T_m = 153.87 °C, ΔH_m = 71.097 J g^{-1}, X_C = 34.3%. 134
Figure S180. Water contact angle measured for *i*PP–*g*–PNIPAM (f_{vinyl} = 5.3%). WCA = 101.5° 134
Figure S181 ^{1}H NMR spectrum (400 MHz, $C_2D_2Cl_4$) of *i*PP–*g*–PS$_{2.8}$ (T = 105 °C) 135
Figure S182. 2D DOSY NMR spectrum (400 MHz, $C_2D_2Cl_4$) of *i*PP–*g*–PS$_{2.8}$ (T = 105 °C) 136
Figure S183. HT SEC trace of *i*PP–*g*–PS$_{2.8}$ (1,2,4-TCB, 1.0 mL min^{-1} at 150 °C) 136
Figure S184. DSC Thermogram of *i*PP–*g*–PS (f_{vinyl} = 2.8%). T_m = 162.22 °C, ΔH_m = 83.411 J g^{-1}, X_C = 40.3%. 137
Figure S185. Water contact angle measured for *i*PP–*g*–PS (f_{vinyl} = 2.8%). WCA = 105.4° 137
Figure S186. ^{1}H NMR spectrum (400 MHz, $C_2D_2Cl_4$) of Plastic bag–*g*–PMA$_{3.7}$ (T = 90 °C) .. 138
Figure S187. 2D DOSY NMR spectrum (400 MHz, $C_2D_2Cl_4$) of Plastic bag–*g*–PMA$_{3.7}$ (T = 90 °C) 139
Figure S188. HT SEC trace of Plastic bag–*g*–PMA (1,2,4-TCB, 1.0 mL min^{-1} at 150 °C) 139
Figure S189. DSC Thermogram of Plastic bag–*g*–PMA (f_{vinyl} = 3.7%). T_m = 106.46 °C, ΔH_m = 79.448 J g^{-1}, X_C = 27.1%. 140
Figure S190. ^{1}H NMR spectrum (400 MHz, $C_2D_2Cl_4$) of Plastic bottle–*g*–PMA$_{2.6}$ (T = 105 °C) 141
Figure S191. 2D DOSY NMR spectrum (400 MHz, $C_2D_2Cl_4$) of Plastic bottle–*g*–PMA$_{2.6}$ (T = 105 °C) 142
Figure S192. HT SEC trace of Plastic bottle–*g*–PMA (1,2,4-TCB, 1.0 mL min^{-1} at 150 °C) ... 142
Figure S193. DSC Thermogram of Plastic bottle–*g*–PMA (f_{vinyl} = 2.6%). T_m = 123.38 °C, ΔH_m = 135.02 J g^{-1}, X_C = 46.1%. 143
Figure S194. ^{1}H NMR spectrum (400 MHz, $C_2D_2Cl_4$) of Falcon tube–*g*–PMA$_{1.7}$ (T = 105 °C) 144
Figure S195. 2D DOSY NMR spectrum (400 MHz, $C_2D_2Cl_4$) of Falcon tube–*g*–PMA$_{1.7}$ (T = 105 °C) 145
Figure S196. HT SEC trace of Falcon tube–*g*–PMA (1,2,4-TCB, 1.0 mL min^{-1} at 150 °C) 145
Figure S197. DSC Thermogram of Falcon tube–*g*–PMA (f_{vinyl} = 1.7%). T_m = 142.19 °C, ΔH_m = 60.451 J g^{-1}, X_C = 29.2%. 146
Figure S198. ^{1}H NMR spectrum (400 MHz, $C_2D_2Cl_4$) of LDPE–*g*–PNEtAM$_{6.8}$ (T = 90 °C) ... 147
Figure S199. 2D DOSY NMR spectrum (400 MHz, $C_2D_2Cl_4$) of LDPE–*g*–PNEtAM$_{6.8}$ (T = 90 °C) 148

Materials

All materials were purchased from either Sigma Aldrich, Fisher Scientific, abcr GmbH, or Tokyo Chemical Industries unless otherwise noted. All the chemicals were used as received unless otherwise stated. *N*-ethyl acrylamide and *N*-n-propyl acrylamides were prepared according to the reported literatures.[1,2]

Methods

NMR Spectroscopy

Spectra were recorded on Bruker Avance spectrometers operating at 300, 400 MHz. Chemical shifts (δ) for NMR spectra were referenced to protons of the residual solvent.[3]

Molar vinyl fraction (f_{vinyl}) Determination

f_{vinyl}s were determined based on ^{1}H NMR spectra. First, the integration value of the polyolefin proton peaks was normalized to correspond to 100 repeat units. Then, the number of vinyl polymer units per 100 polyolefin repeat units was calculated from the integration value of a characteristic vinyl polymer peak (e.g., the methyl peak of methyl acrylate at ~3.72 ppm). In cases where proton signals from the vinyl polymer overlapped with those of the polyolefin region, the integration was corrected by back-calculating the number of protons contributing to the overlap based on the number of characteristic protons in the vinyl polymer.

Size-exclusion chromatography (SEC)

For polyolefin samples, analyses were carried out using an Agilent 1260 Infinity II equipped with two PSS POLEFIN analytical XL columns, eluted with 1,2,4-trichlorobenzene at 150 °C at a rate of 1.0 mL/min and calibrated using polystyrene standards. For the small molecule study samples, analyses were carried out using an Agilent 1260 Infinity II SEC system with a Wyatt OptiLab T-rEx refractive index (RI) detector. The SEC system was equipped with two Shodex LF-804 columns, eluted with THF at 35 °C at a rate of 1.0 mL/min and calibrated using polystyrene standards.

Atomic force microscopy (AFM)

AFM analyses were performed on a Bruker Multimode 8 in tapping (non-contact) mode using commercially available tips (Nanoworld, Pointprobe® NCHR; spring constant, 42 N/m; tip radius, ≤ 8 nm). Samples were prepared by spin-coating polymer solutions in boiling PhCl (10 g/L) onto preheated glass substrates (~200 °C) at 1000 rpm for 10 s.

Scanning transmission electron microscopy (STEM)

STEM analyses were performed on a TFS Talos F200X (Thermofisher Scientifc, USA) instrument operated at 200 kV in a STEM mode with a probe size of about 0.25 nm and 200 pA of the probe current. The STEM imaging was conducted using the simultaneous acquisition of signals by bright field (BF), low angle annular dark field (LAADF) and high angle annular dark field (HAADF) detectors, which allowed for the complementary "diffraction contrast" and "atomic number contrast" imaging. STEM studies also included the analytical, energy dispersive X-ray spectroscopy in a Spectrum Imaging (SI) mode using a SuperX EDS module of the Tal0os F200X. The STEM data (including EDS) were processed using the TFS Velox software.

Ultrathin sections of approximately 100nm thickness were obtained at cryoconditions from free-standing films (130µm) on an ultramicrotome Leica EM FC6 Cryo (Leica Microsystems, AT) and were collected on Quantifoil R2/1 with an additional layer of 2nm carbon (Quantifoil, DE).

Matrix-Assisted Laser Desorption/Ionization Time-of-Flight Mass Spectrometry (MALDI-TOF MS)

Analyses were performed on a Bruker Auto Flex MaX instrument (positive linear mode). *trans*-2-[3-(4-*tert*-Butylphenyl)-2-methyl-2-propenylidene]malononitrile (DCTB) was used as the matrix and silver triflate (AgOTf) was employed as the cation source.

Differential Scanning Calorimetry (DSC)

Data was recorded on a Waters Discovery DSC 2500 using 3–5 mg of a given sample. All melting temperature (T_m) and melting enthalpy (ΔH_m) values were obtained from a second scan, and the heating/cooling rate was 10 ºC/min. For LDPE samples, ΔH_m was determined by integrating the heating trace from 50 to 120 °C. For LLDPE, HDPE, and PP samples, the corresponding integration ranges were 60 to 140 °C, 60 to 150 °C, and 100 to 180 °C, respectively.

X-ray Diffraction (XRD)

XRD measurements were performed on free-standing film samples using a Panalytical X'Pert PRO MPD diffractometer equipped with Cu-Kα radiation (40 kV, 45 mA).

Small-Angle X-ray Scattering (SAXS)

SAXS measurements were conducted on free-standing film samples using a Xeuss 3.0 instrument with Cu-Kα radiation.

Wavelengths of LED Emission

The emission spectrum of the LED lamps employed in the study were recorded using a Horiba Duetta fluorescence and absorbance spectrometer.

Contact Angle (CA)

Measurements were carried out using KRUSS DSA 100 and deionized water (resistivity = 18.2 MΩ cm). For the samples in **Fig. 2G**, detailed analyses were performed using H_2O, CH_2I_2, EtOH/H_2O (8:2, v/v), benzyl alcohol, and ethylene glycol as probe liquids. The polar and dispersive components were determined using the Owens–Wendt–Rabel–Kaelble (OWRK) model (**Figs. 29–31**).[4,5]

Uniaxial Tensile Elongation Tests

Tensile tests were performed using a Zwick Z010 machine equipped with a 200 N load cell at a crosshead speed of 12 mm/min. A pre-load of 0.05 N was applied prior to testing. Free-standing films were prepared by hot-pressing at 150–180 °C and subsequently cut into dog-bone-shaped specimens with dimensions of $12 \times 2.0 \times 0.1–0.3\ mm^3$. Extension-to-failure tests were conducted on at least three specimens. The Young's modulus was determined from the slope of the stress–strain curve in the 0–1% strain range.

Lap Shear Tests

Lap shear tests were performed using a Zwick Z010 machine equipped with a 50 kN load cell at a crosshead speed of 1 mm/min. A free-standing polymer film was sandwiched between two metal strips (120 × 25 mm^2) or wooden sticks, and the three-layer specimen was clamped with two medium binder clips (25 mm) before curing at 190 °C for 1 h. After cooling to room temperature, extension-to-failure tests were performed on at least three specimens. Adhesion strength was calculated as the maximum load divided by the bonded area, with bonded areas measured from post-test images using ImageJ software.

General Synthetic Procedure for Direct Polymer Grafting

A 22 mL borosilicate test tube was charged with polyolefin (281 mg, 10 mmol for PE and 6.66 mmol for PP) and 1,2-dichlorobenzene (10 mL), and sealed with a rubber septum. The mixture was degassed by nitrogen bubbling at 120 °C until it became homogeneous, then cooled to the predetermined temperature. The monomer was then added via syringe. For solid monomers (e.g., *N*-isopropylacrylamide), the monomer was introduced as a stock solution in 1,2-dichlorobenzene. The reaction mixture was stirred and irradiated

with a 390 nm LED lamp for the predetermined time. Upon completion, the reaction mixture was precipitated into cold methanol or acetone (ca. 40 mL). The resulting solid was collected by filtration and purified by Soxhlet extraction with acetone or isopropanol overnight (ca. 4 min per cycle). After purification, the solid was dried in a vacuum oven (3 mbar) at 80 °C for 16 h. The products were characterized by ^{1}H NMR (90 or 105 °C in $C_2D_2Cl_4$, 400 MHz), 2D DOSY NMR, DSC, and high-temperature size-exclusion chromatography.

<u>Isolated Yield, Monomer Conversion, Graft Ratio and Graft Yield Calculations</u>

Isolated reaction yield was calculated as the ratio between the isolated product mass to the total mass of polyolefin and monomer charged at the start of the reaction.

Because most monomers used in this study are volatile, monomer conversion was determined as follows. After the reaction, a small aliquot of the reaction mixture (approximately 0.1 mL) was dried in a vacuum oven (2 mbar, 80 °C, 16 h). The resulting crude solid was dissolved in 0.5 mL of $C_2D_2Cl_4$. Monomer conversion was quantified from the remaining polymer signals in the ^{1}H NMR spectrum using the polyolefin backbone signal as an internal standard, assuming complete removal of unreacted monomer during drying.

The graft ratio was obtained by comparing the molar ratio of polyolefin to vinyl polymer signals before and after Soxhlet extraction.

The graft yield was calculated by dividing the final level of functionalization by the initial monomer feed ratio.

Supplementary Figures

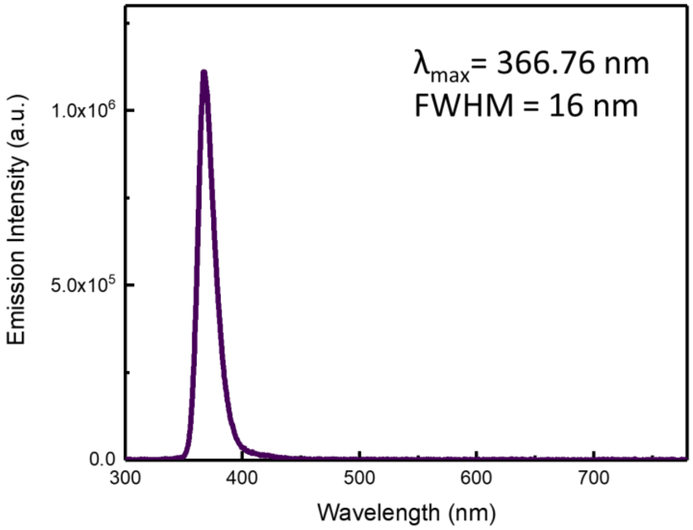

Figure S1. Emission spectrum of the 365 nm LED lamp (HepatoChem, EvoluChem365PF EU, 190 mW cm^{-2}).

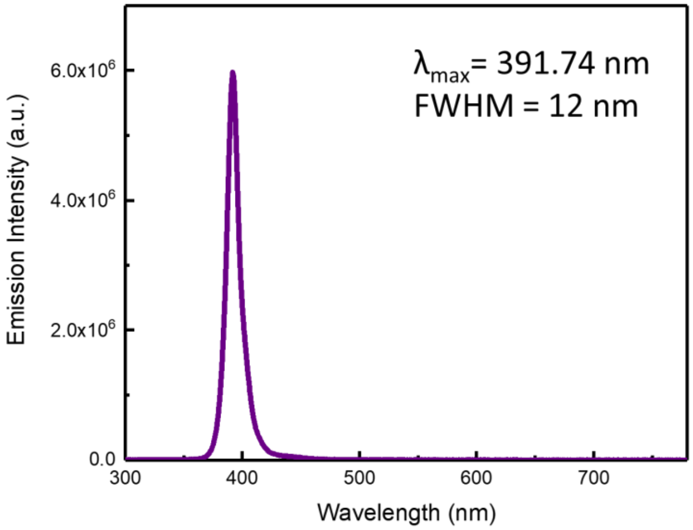

Figure S2. Emission spectrum of the 390 nm LED lamp (HepatoChem, EvoluChem390PF EU, 294 mW cm^{-2}).

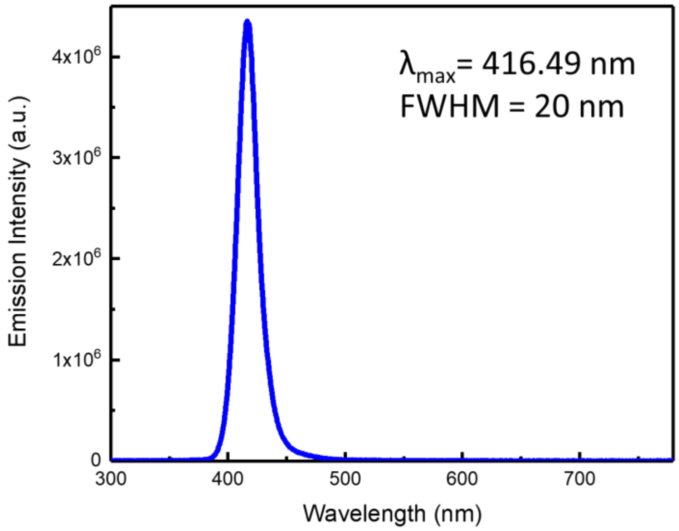

Figure S3. Emission spectrum of the 425 nm LED lamp (HepatoChem, EvoluChem390PF EU, 166 mW cm^{-2}).

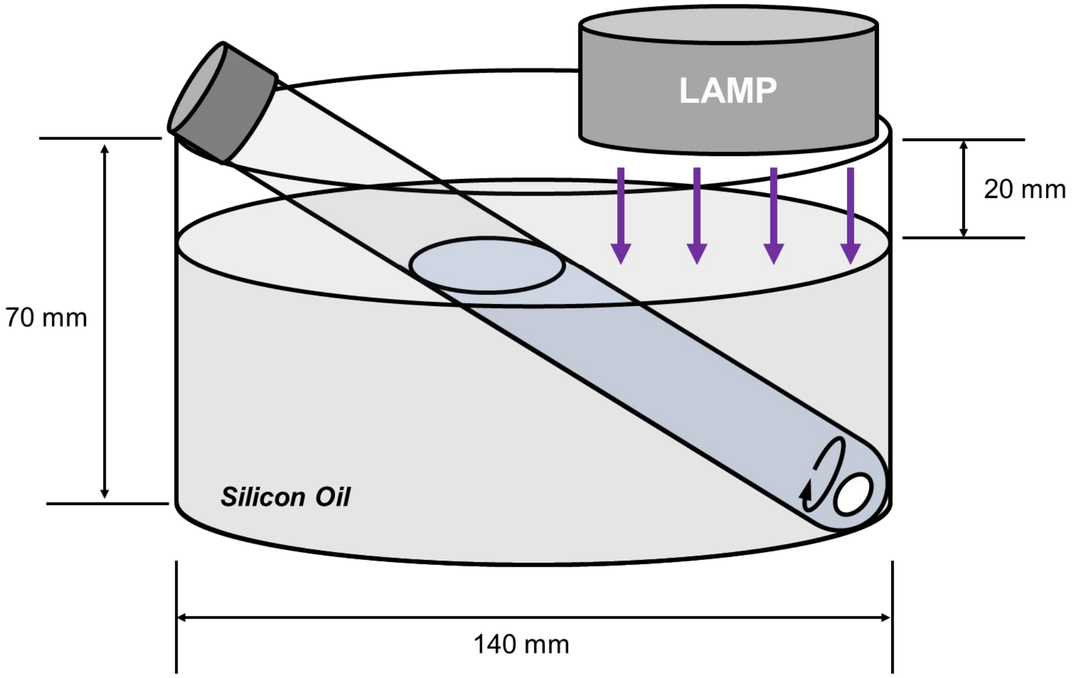

Figure S4. Schematic depiction of the general reaction set up.

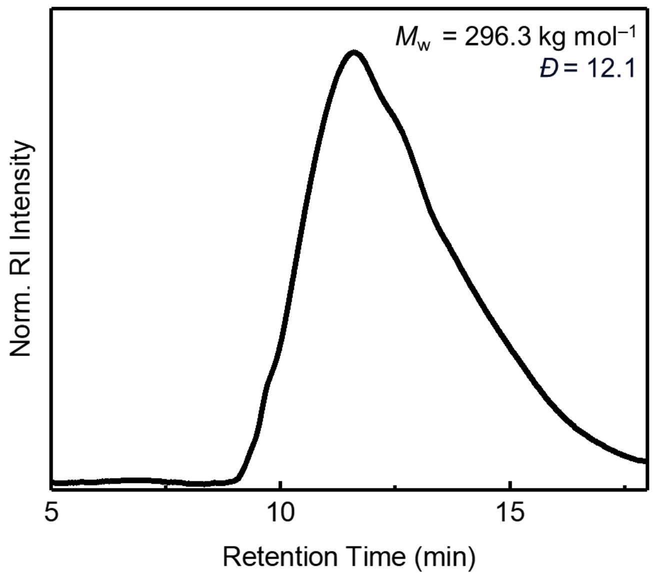

Figure S5. HT SEC trace of LDPE (1,2,4-TCB, 1.0 mL min^{-1} at 150 °C)

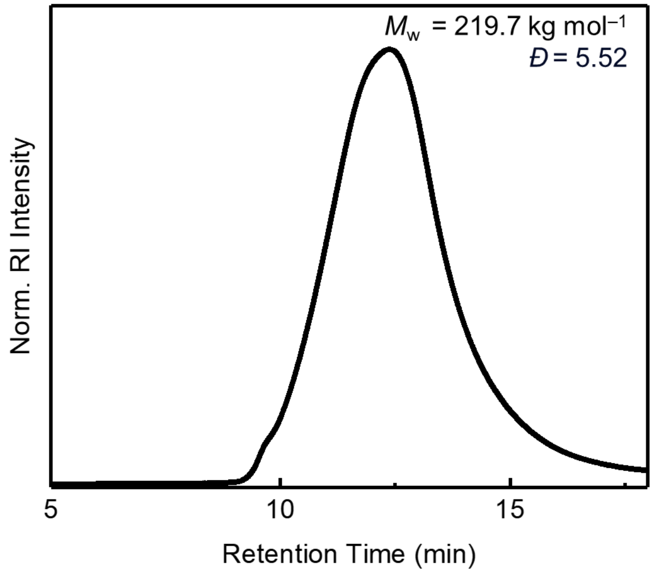

Figure S6. SEC trace of LLDPE (1,2,4-TCB, 1.0 mL min^{-1} at 150 °C)

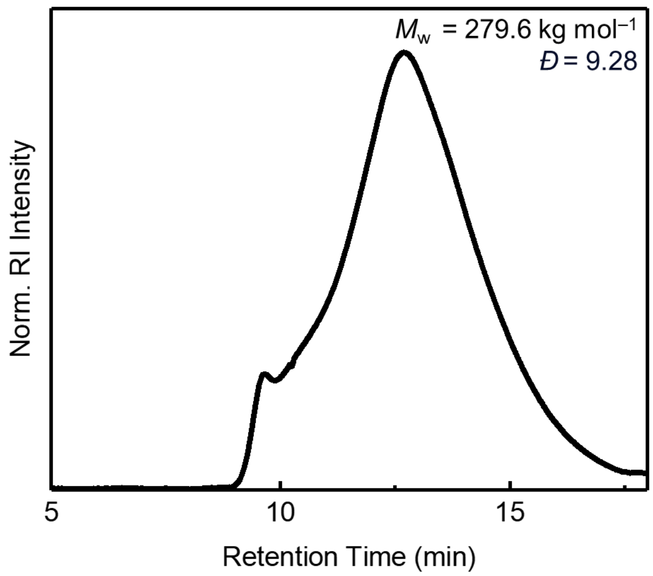

Figure S7. HT SEC trace of HDPE (1,2,4-TCB, 1.0 mL min^{-1} at 150 °C)

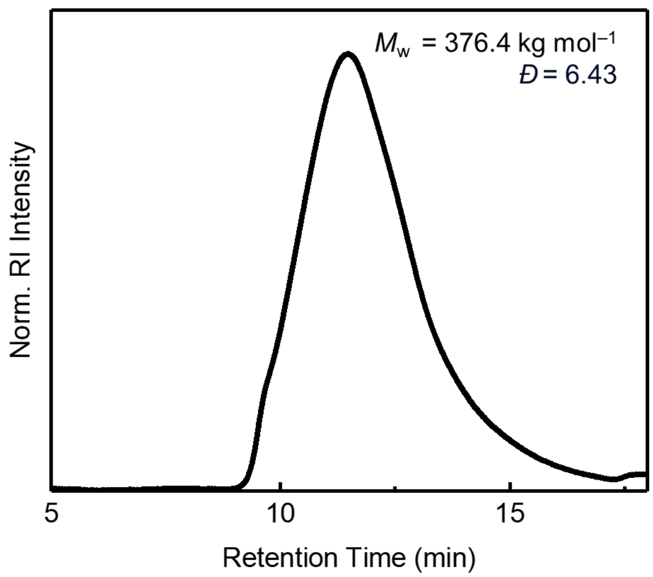

Figure S8. HT SEC trace of *i*PP (1,2,4-TCB, 1.0 mL min^{-1} at 150 °C)

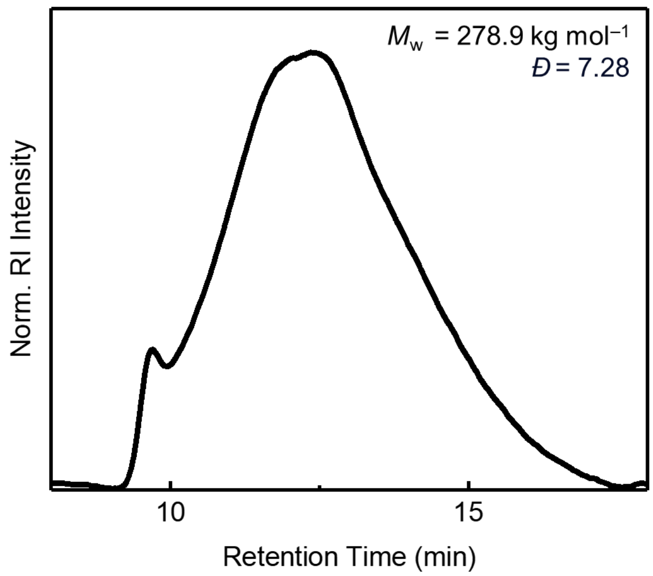

Figure S9. HT SEC trace of a plastic bag (1,2,4-TCB, 1.0 mL min^{-1} at 150 °C)

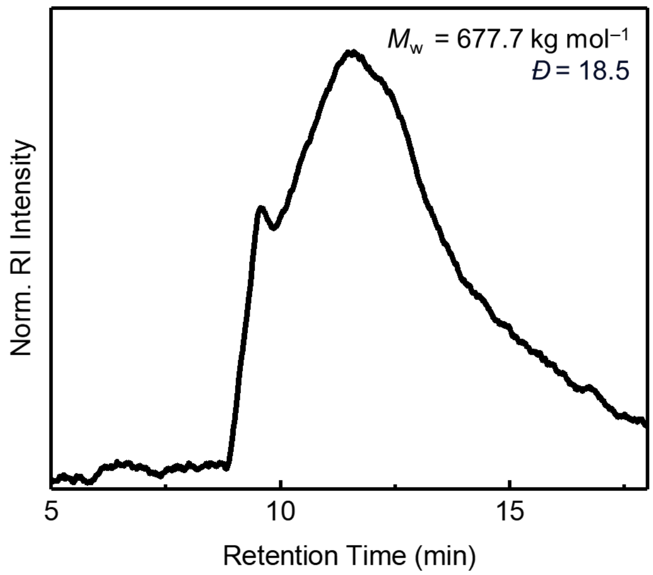

Figure S10. HT SEC trace of a plastic bottle (1,2,4-TCB, 1.0 mL min^{-1} at 150 °C)

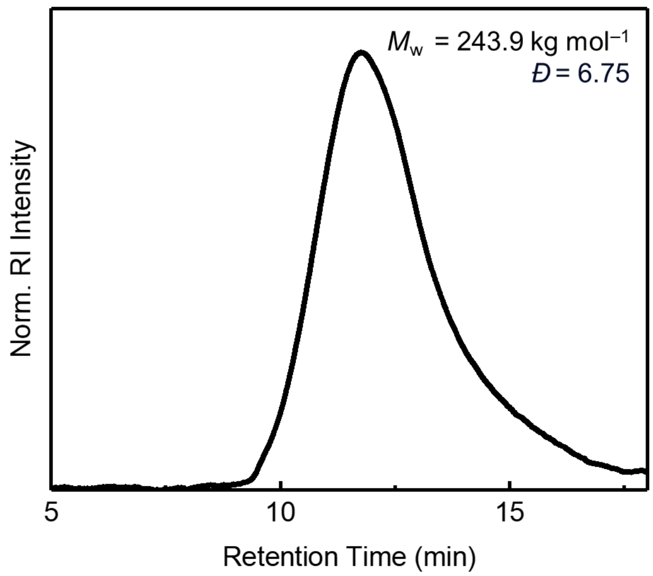

Figure S11. HT SEC trace of a falcon tube (1,2,4-TCB, 1.0 mL min^{-1} at 150 °C)

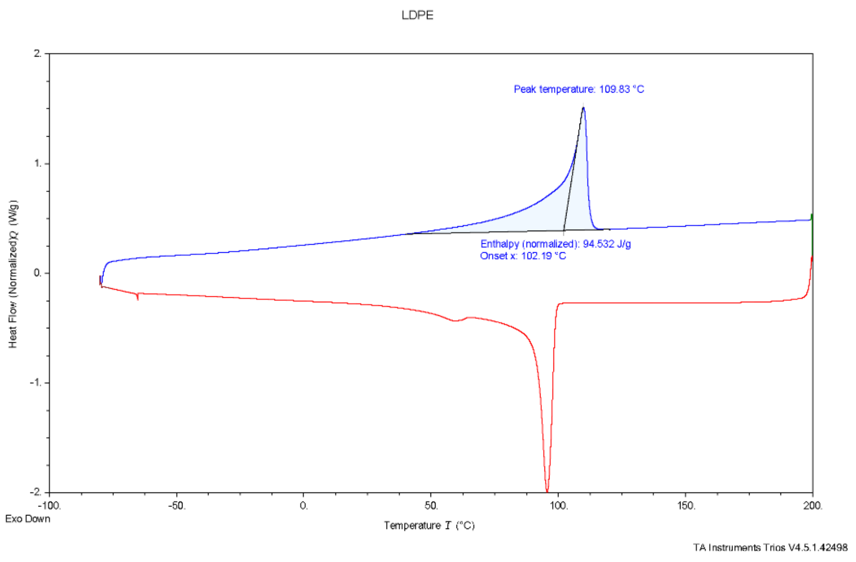

Figure S12. DSC Thermogram of LDPE used in this study. T_m = 109.83 °C, ΔH_m = 94.532 J g^{-1}, X_C = 32.3%.

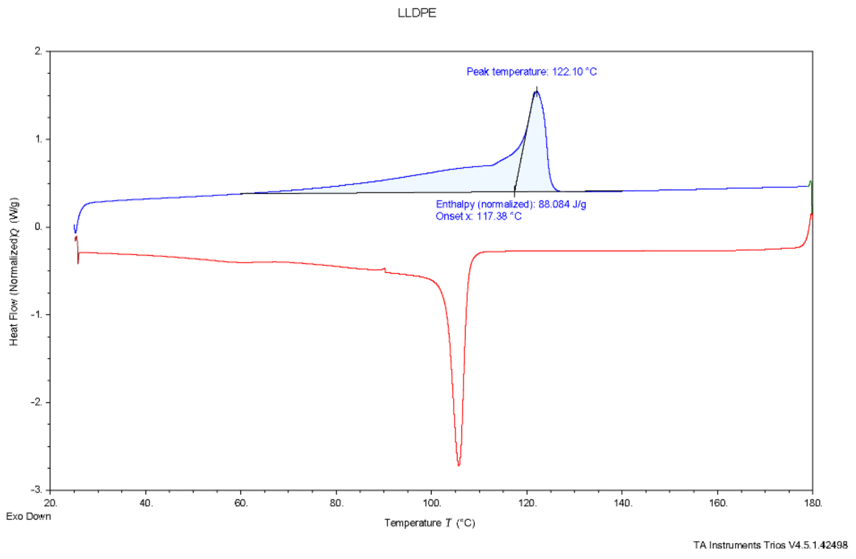

Figure S13. DSC Thermogram of LLDPE used in this study. T_m = 122.10 °C, ΔH_m = 88.084 J g^{-1}, X_C = 30.1%.

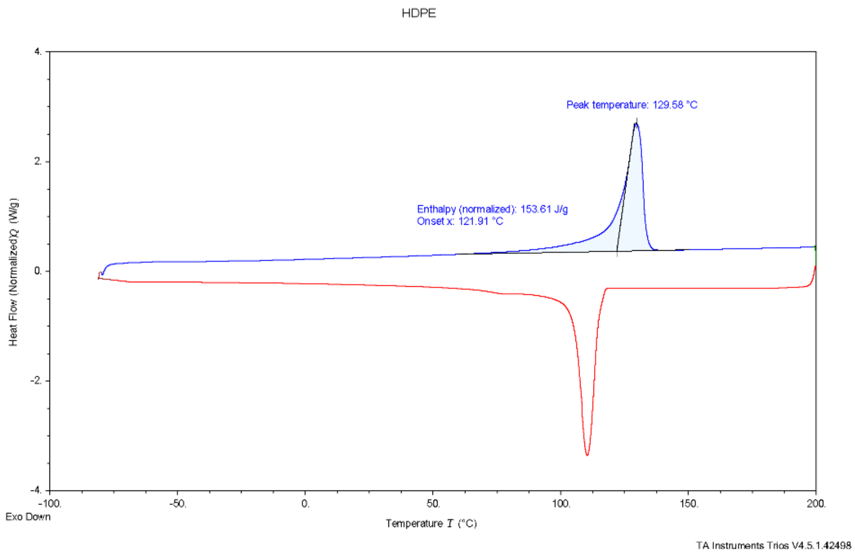

Figure S14. DSC Thermogram of HDPE used in this study. T_m = 129.58 °C, ΔH_m = 153.61 J g^{-1}, X_C = 52.4%.

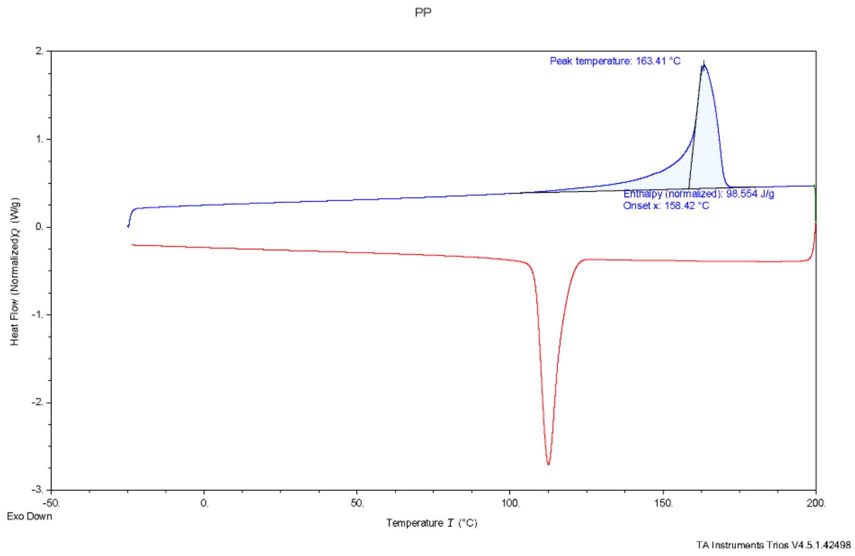

Figure S15. DSC Thermogram of *i*PP used in this study. T_m = 163.41 °C, ΔH_m = 98.554 J g^{-1}, X_C = 47.6%.

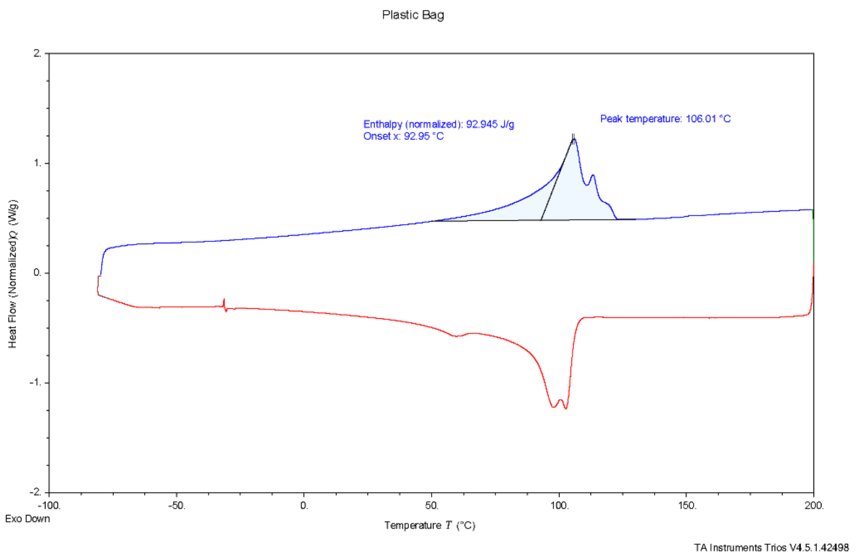

Figure S16. DSC Thermogram of a plastic bag used in this study. T_m = 106.01°C, ΔH_m = 92.945 J g^{-1}, X_C = 31.7%.

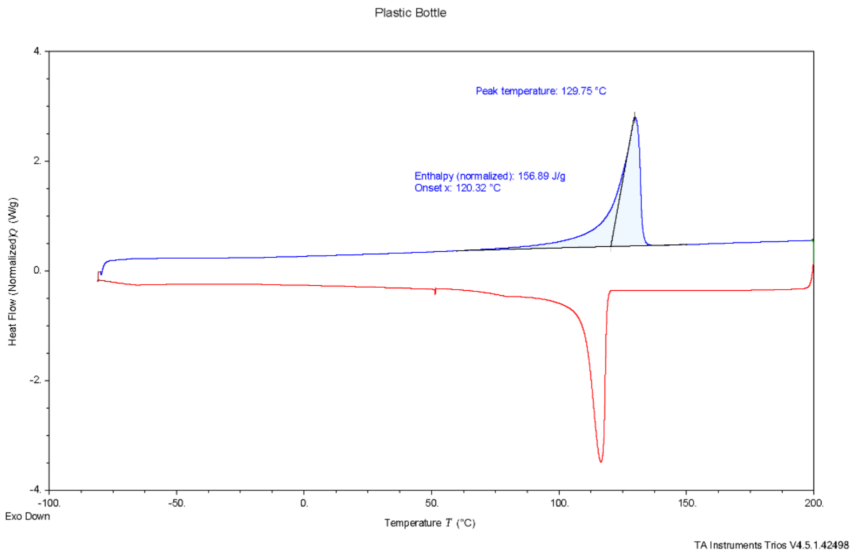

Figure S17. DSC Thermogram of a plastic bottle used in this study. T_m = 129.75°C, ΔH_m = 156.89 J g^{-1}, X_C = 53.5%.

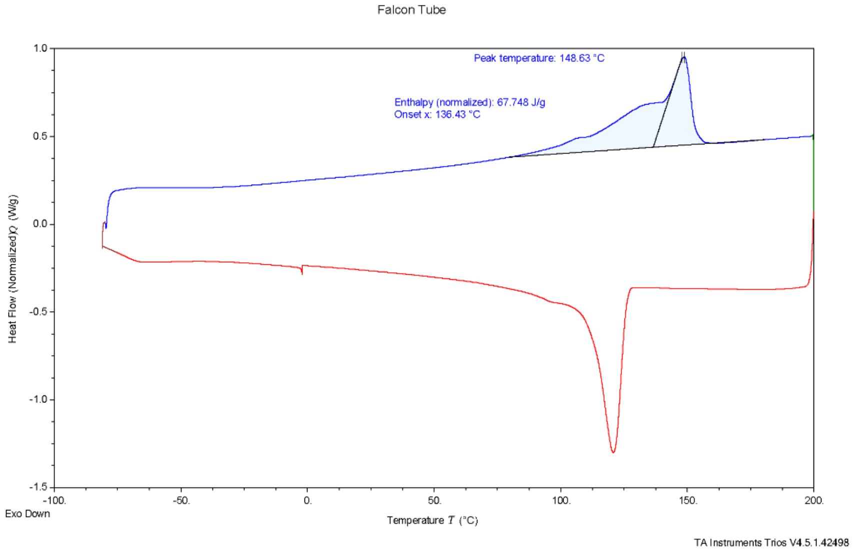

Figure S18. DSC Thermogram of a falcon tube used in this study. T_m = 148.63 °C, ΔH_m = 67.75 J g^{-1}, X_C = 32.7%.

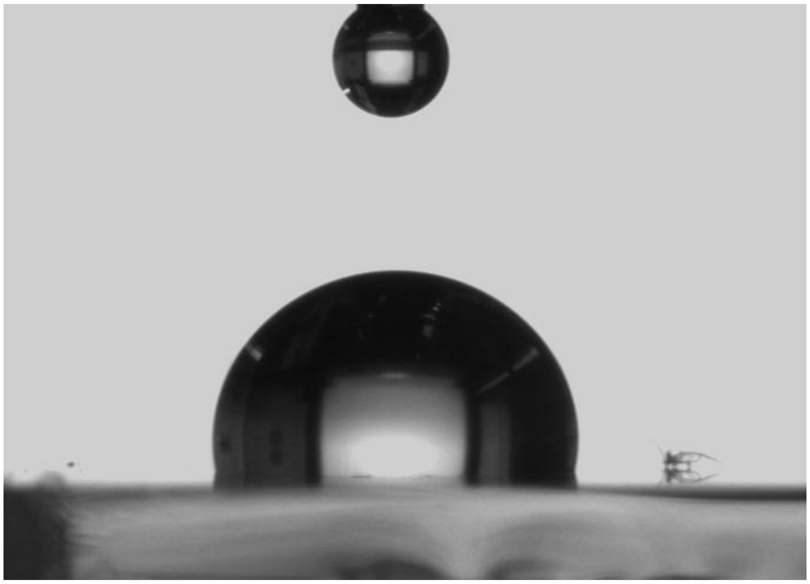

Figure S19. Water contact angle measured for LDPE used in this study. WCA = 103.7°

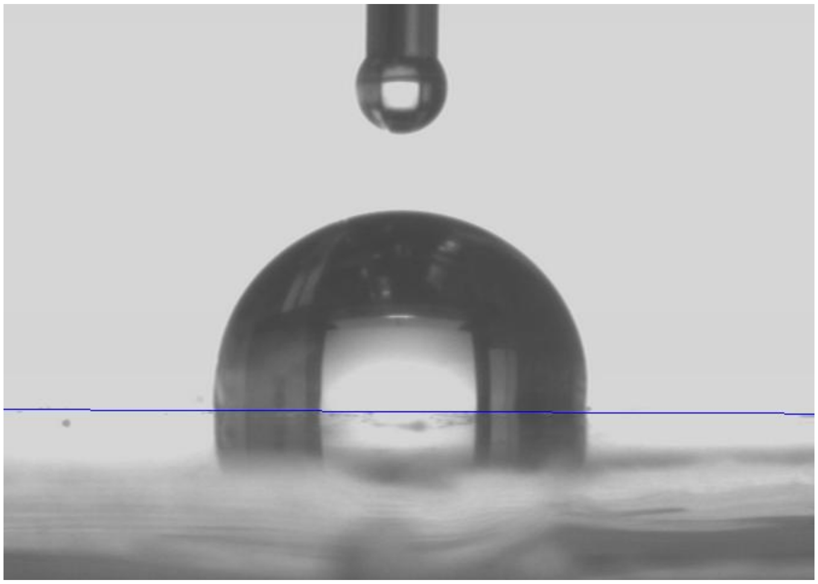

Figure S20. Water contact angle measured for LLDPE used in this study. WCA = 100.0°

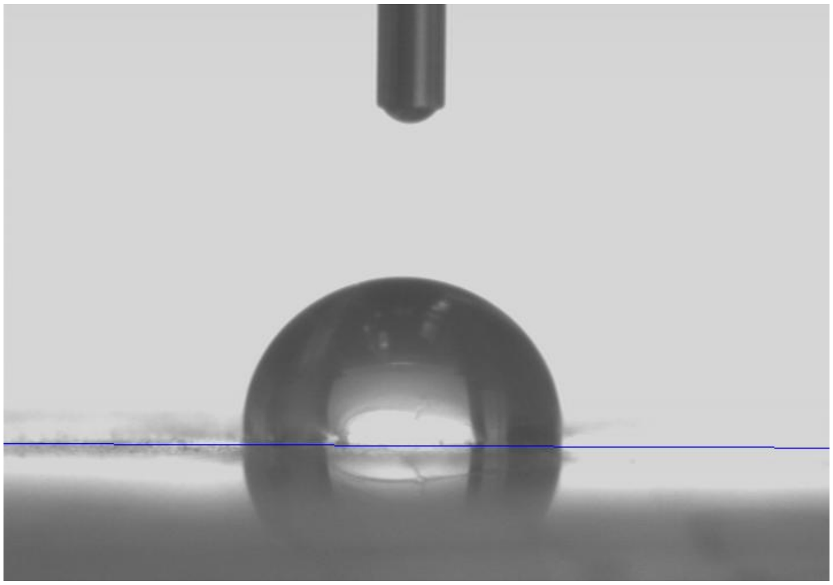

Figure S21. Water contact angle measured for HDPE used in this study. WCA = 93.6°

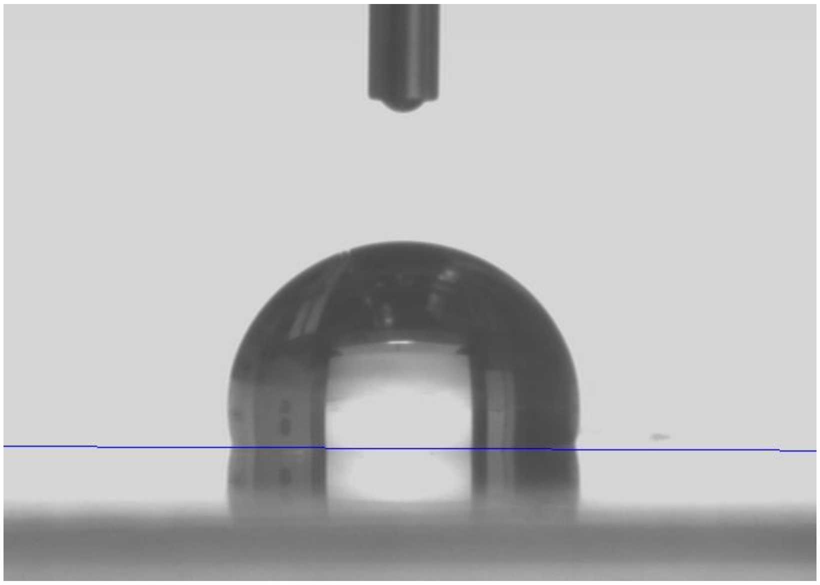

Figure S22. Water contact angle measured for *i*PP used in this study. WCA = 105.3°

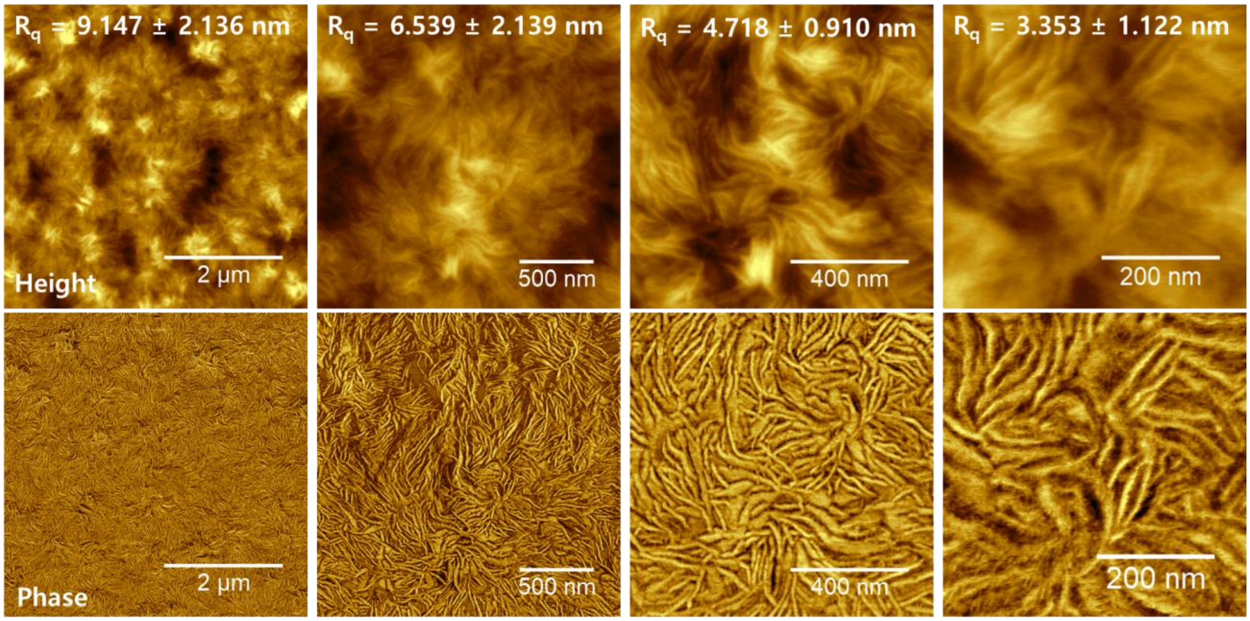

Figure S23. AFM height (top) and phase (bottom) images of LDPE.

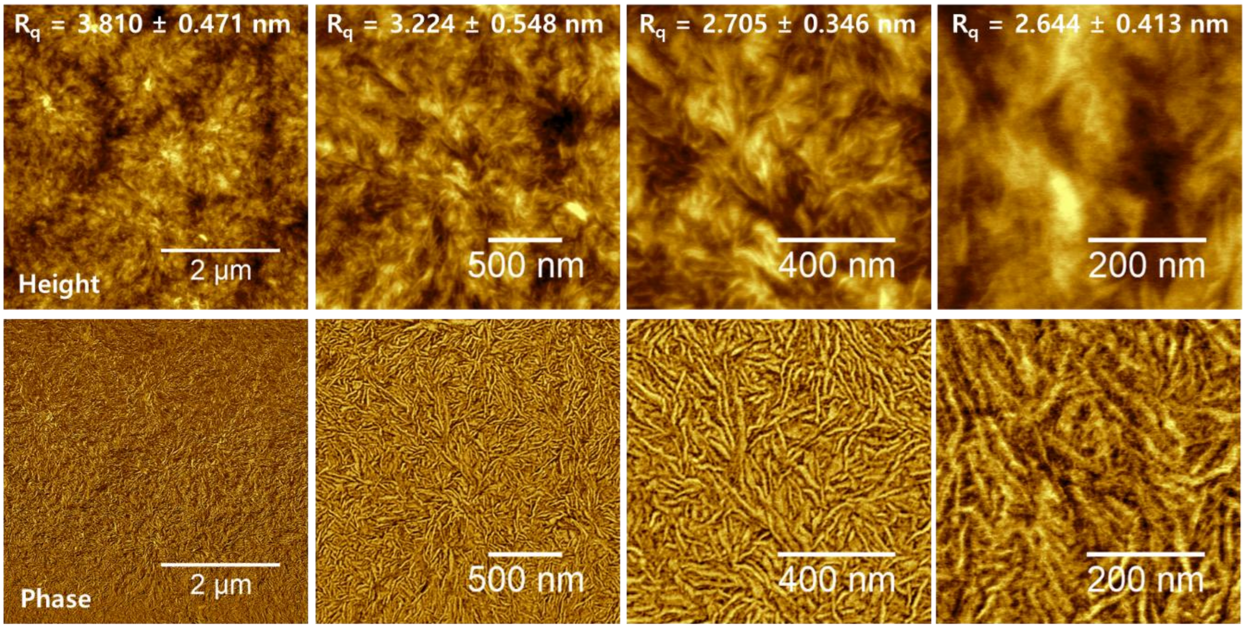

Figure S24. AFM height (top) and phase (bottom) images of LDPE–*g*–PMA (Fig. 2).

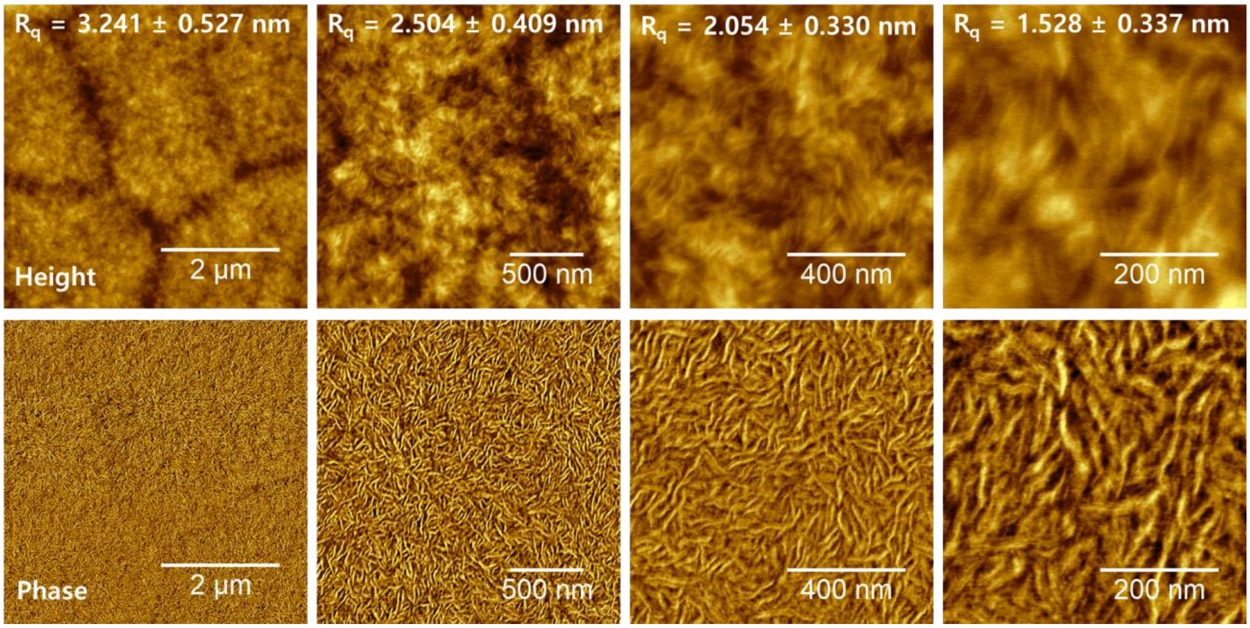

Figure S25. AFM height (top) and phase (bottom) images of LDPE–*g*–MA (Fig. 2).

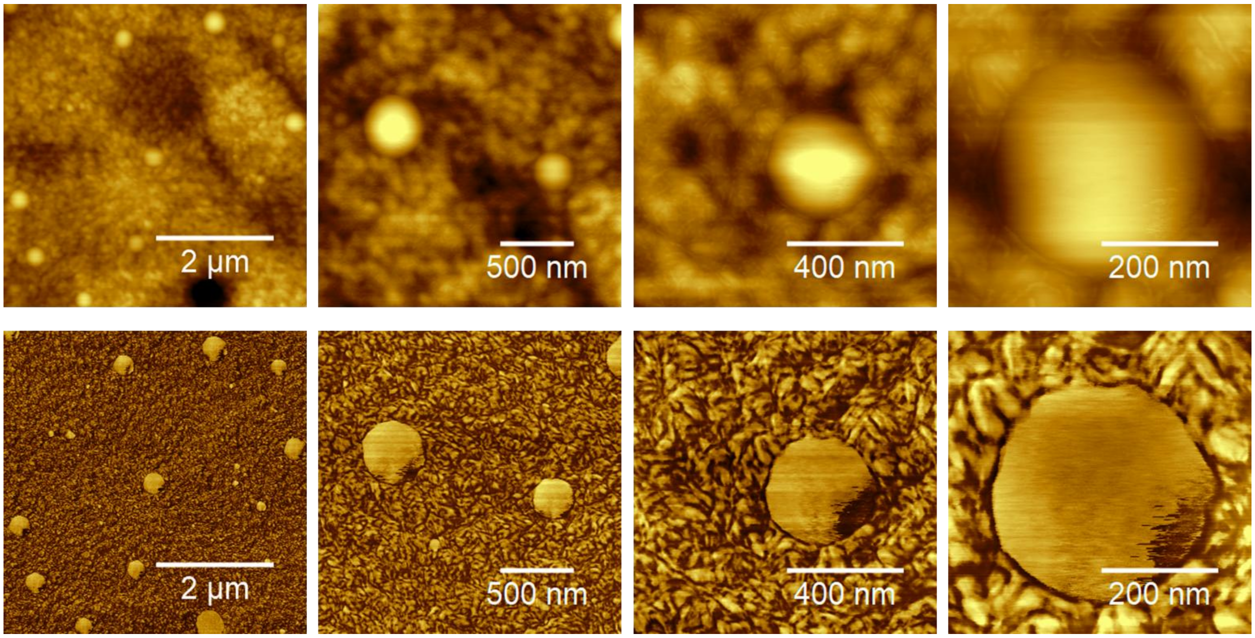

Figure S26. AFM height (top) and phase (bottom) images of LDPE/PMA (16.4 wt% of PMA) blend.

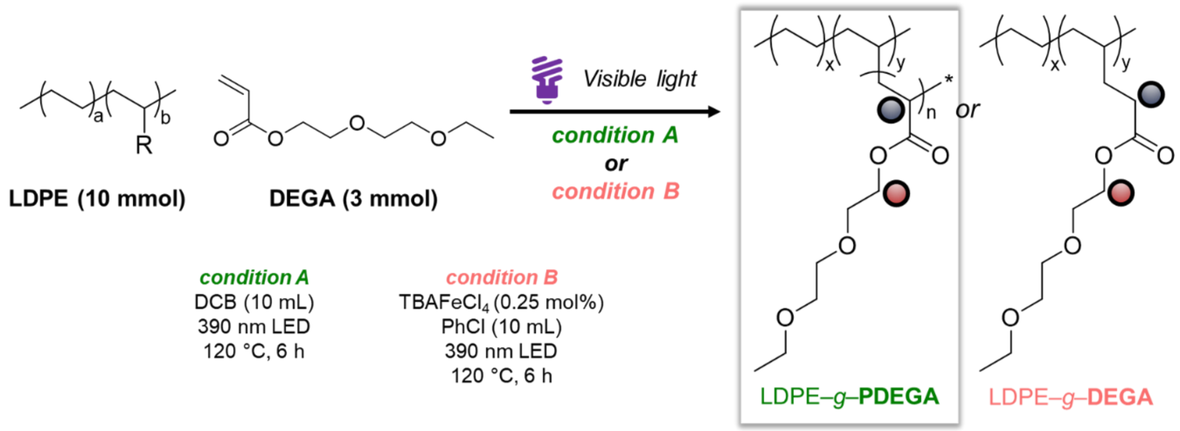

Entry	LOF (%)	●:●	T_m (°C)	X_c[c]
LDPE–*g*–**PDEGA**	1.6	2:0.91	**106.4**	**26.4**
LDPE–*g*–**DEGA**	1.9	2:2.37	101.4	21.1
**LDPE**	–	–	109.1	32.3

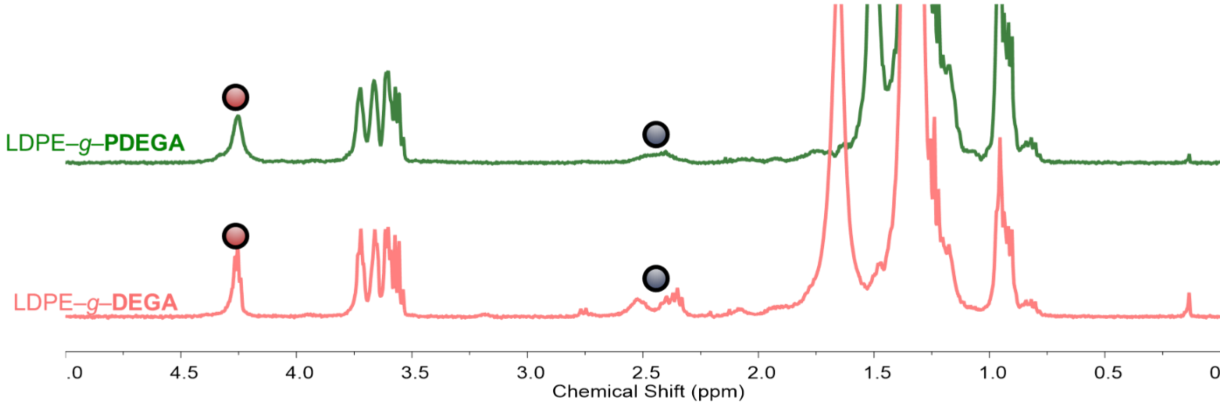

Figure S27. Synthesis and characterization of LDPE–*g*–PDEGA and LDPE–*g*–DEGA.

a

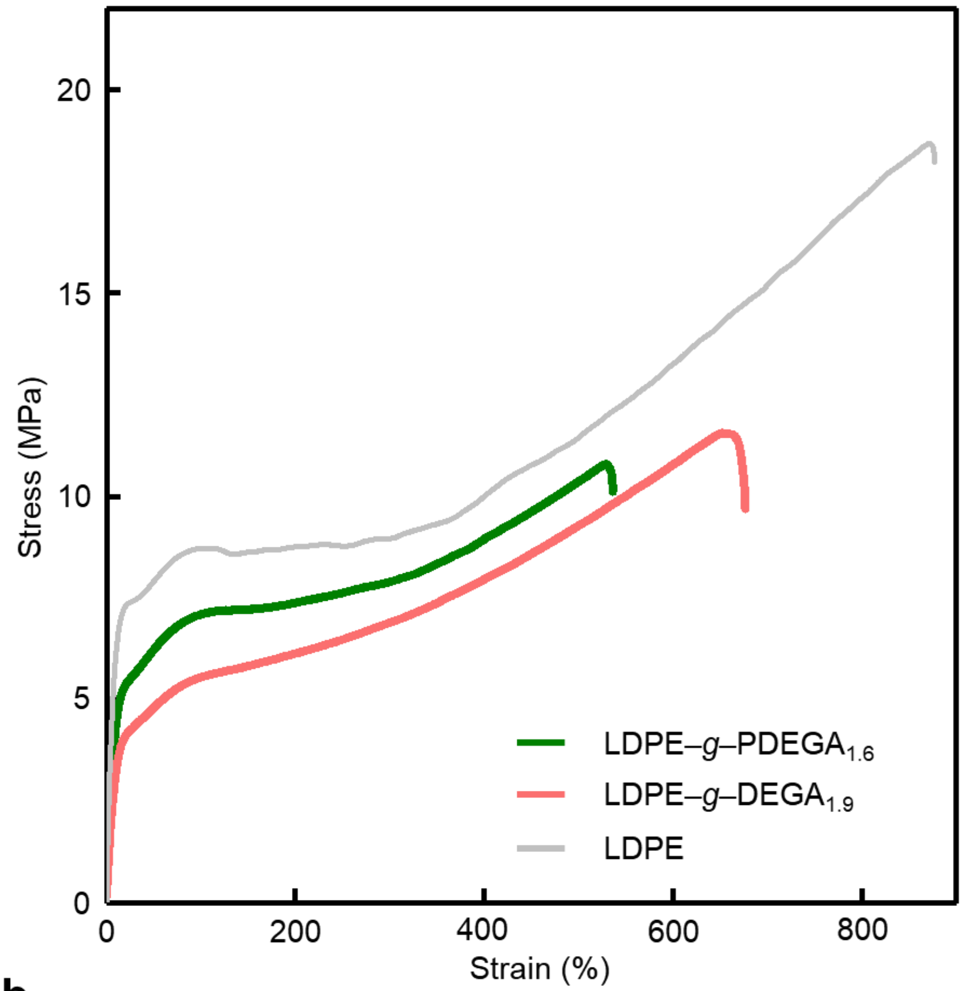

b

	E (MPa)[a]	σ_y (MPa)[b]	σ_u (MPa)[c]	ε_{break} (%)[d]
**LDPE–g–PDEGA**	79±4	5.3±0.1	11.4±1.0	531±26
**LDPE–g–DEGA**	48±3	4.0±0.1	10.4±2.8	522±222
LDPE	145±3	7.1±0.04	18.6±1.2	857±70

[a] Elastic modulus, [b] yield stress, [c] ultimate tensile strength, [d] elongation at break

Figure S28. (**a**) Stress–strain curves of LDPE–*g*–PDEGA (green), LDPE–*g*–DEGA (red), and LDPE (gray). (**b**) Summary of mechanical tests.

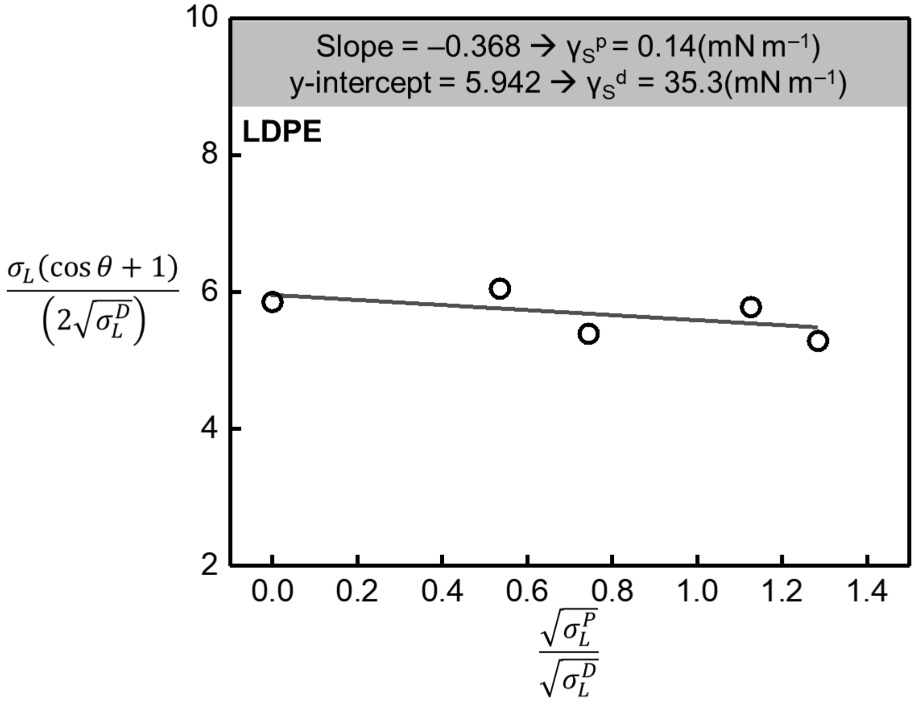

Figure S29. Contact angle analysis of LDPE

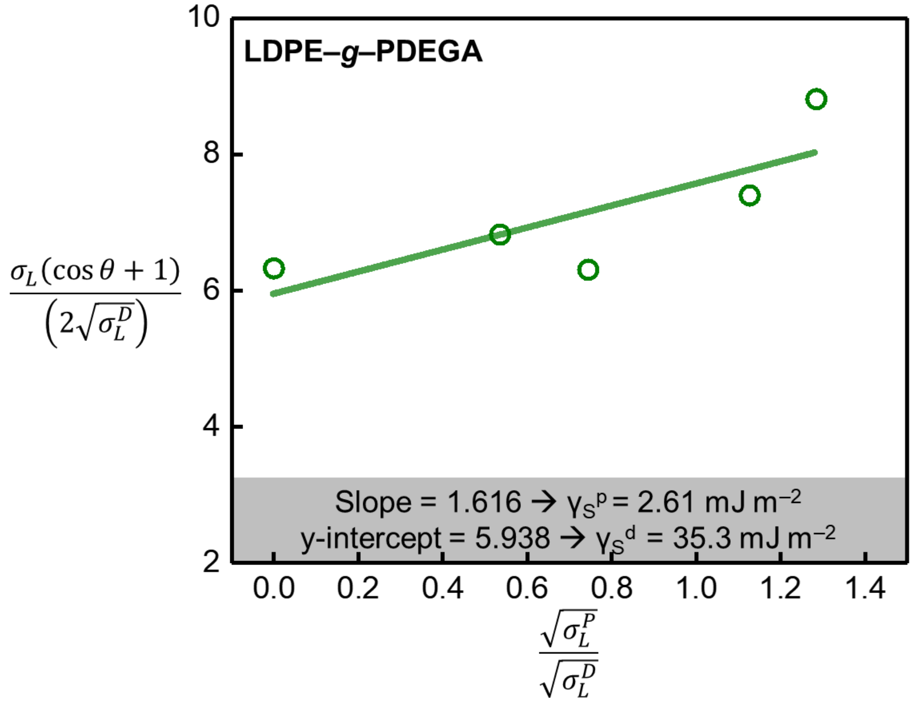

Figure S30. Contact angle analysis of LDPE–*g*–PDEGA.

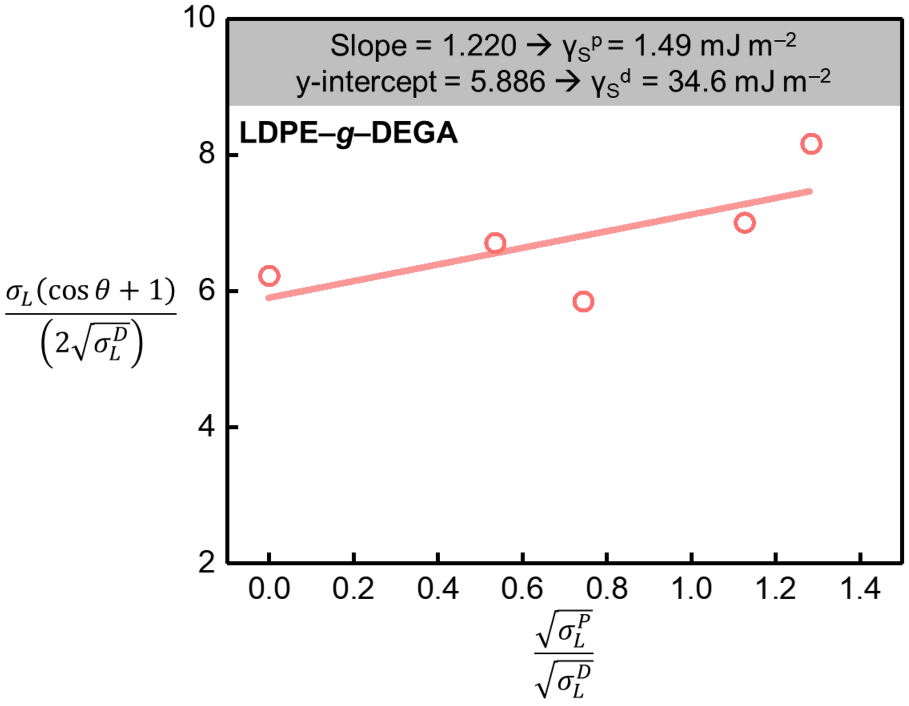

Figure S31. Contact angle analysis of LDPE–*g*–DEGA.

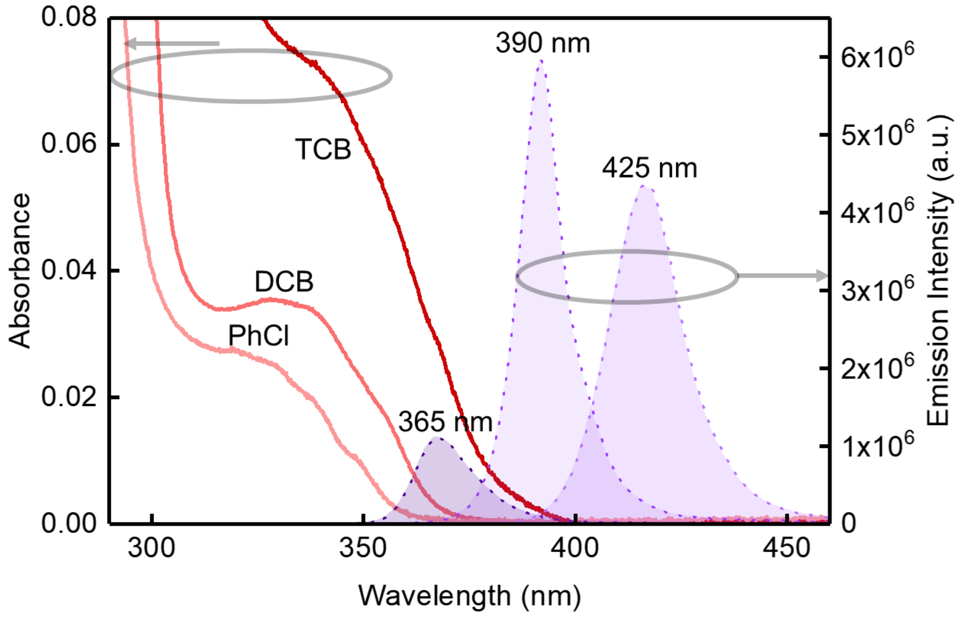

Figure S32. Overlay of Solvent UV–vis absorption spectra and emission spectra of UV lamp.

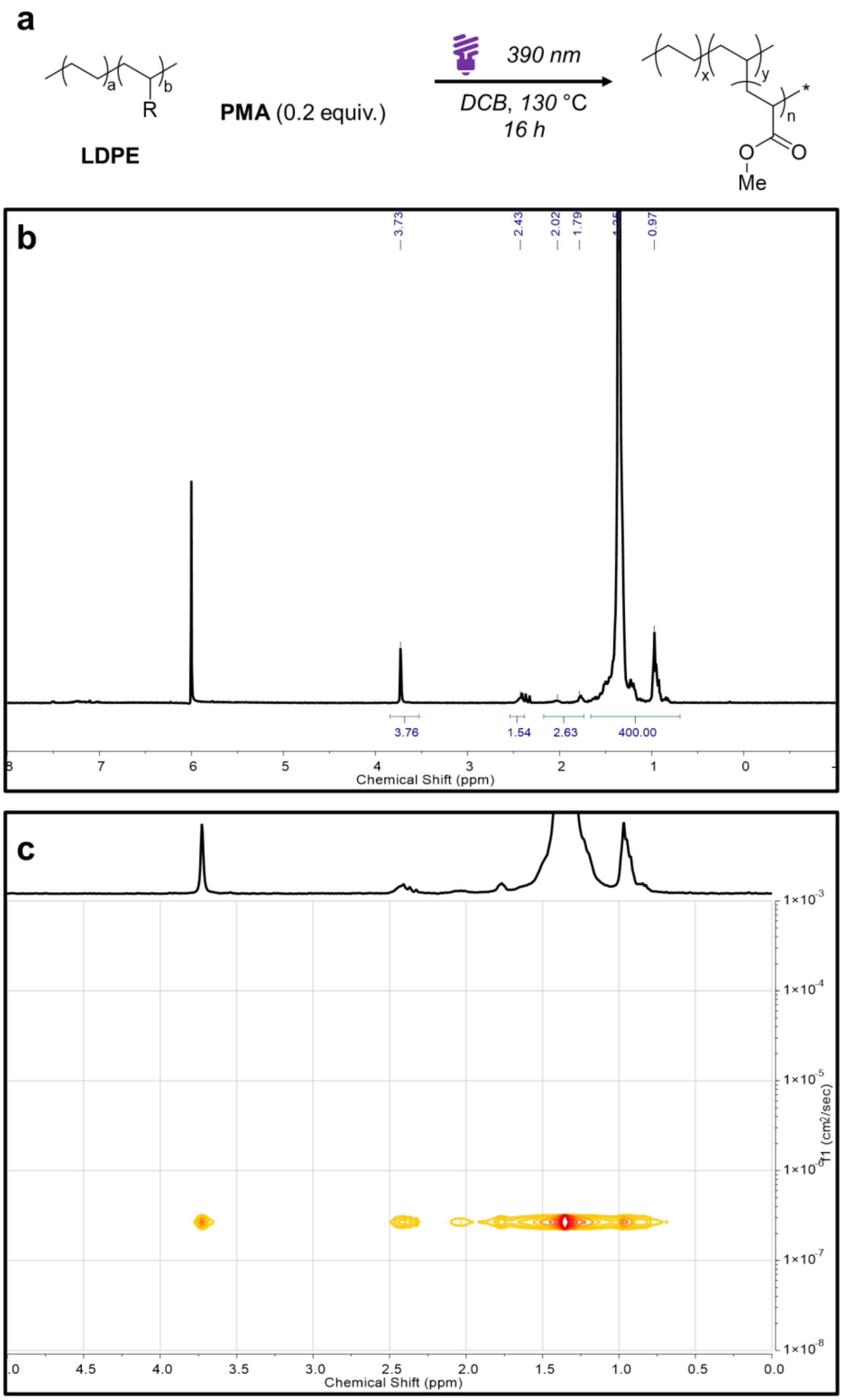

Figure S33. (**A**) Reaction scheme of the PMA grafting experiment (Fig. 3A, entry 11). (**B**) ^{1}H NMR spectrum (400 MHz, $C_2D_2Cl_4$). (**C**) 2D DOSY NMR spectrum.

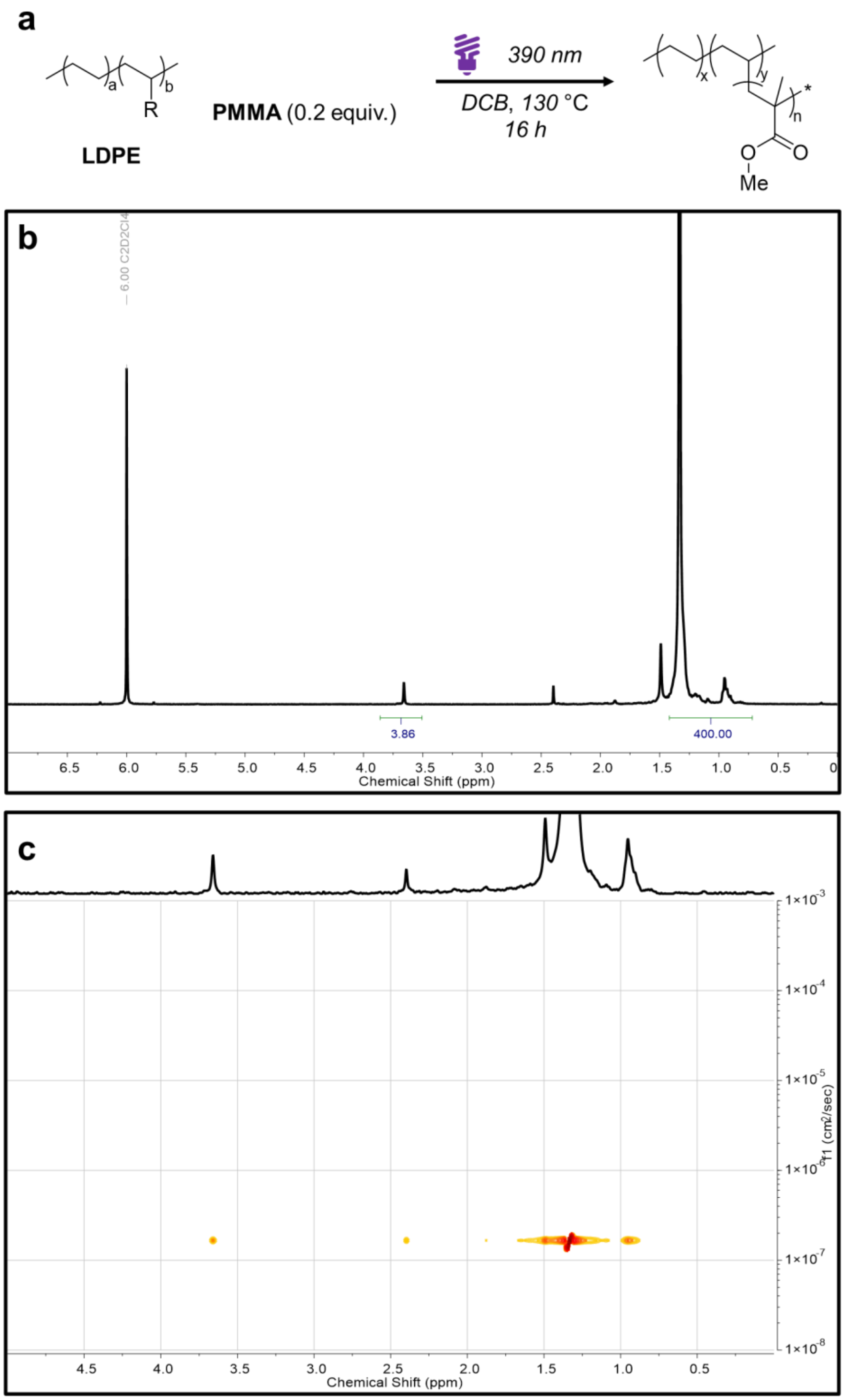

Figure S33. (**A**) Reaction scheme of the PMMA grafting experiment. (**B**) ^{1}H NMR spectrum (400 MHz, $C_2D_2Cl_4$). (**C**) 2D DOSY NMR spectrum.

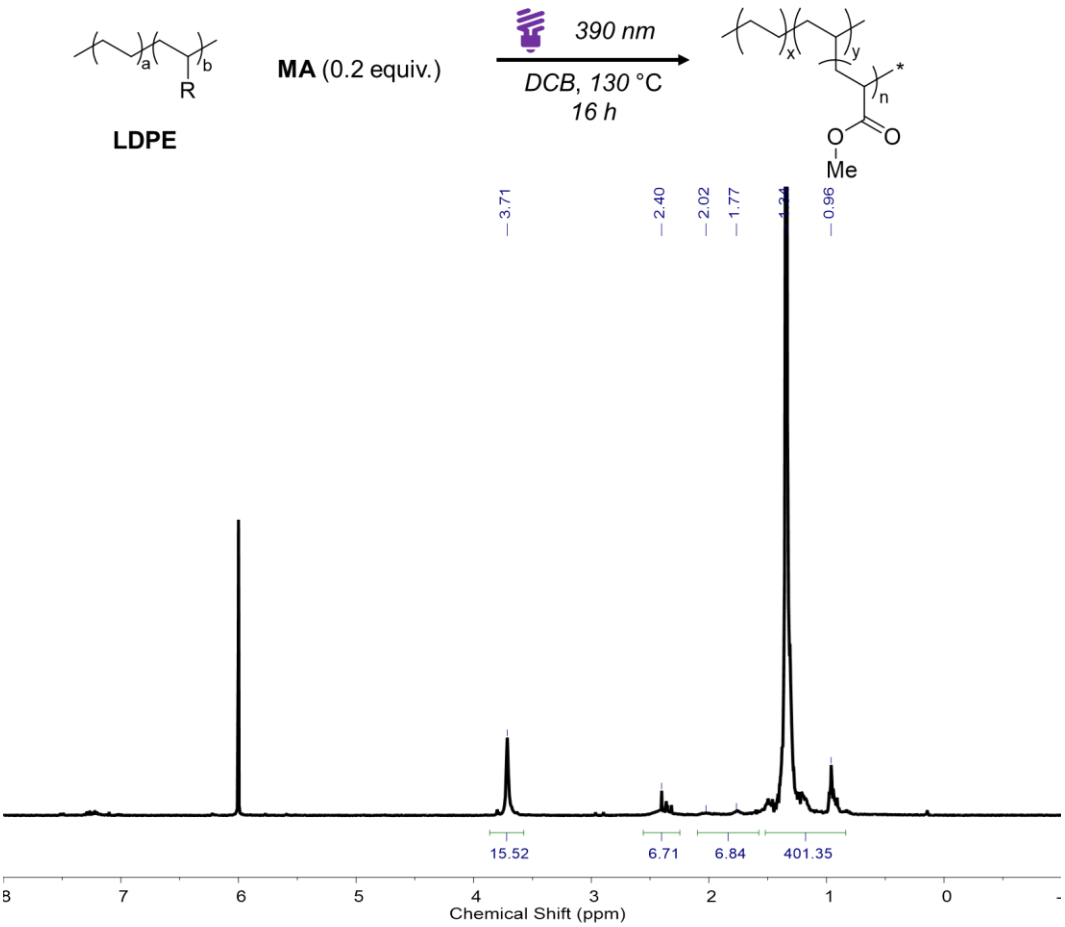

Figure S35. ^{1}H NMR spectrum (400 MHz, $C_2D_2Cl_4$) of LDPE–*g*–PMA (**Fig. 3A** entry 12).

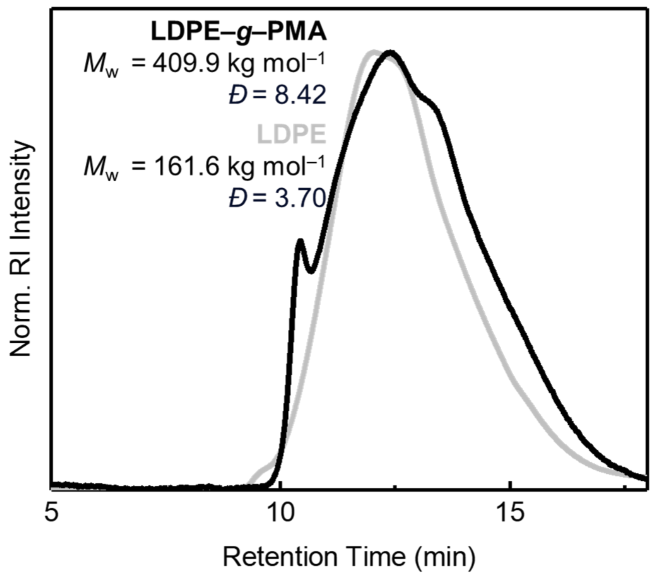

Figure S36. HT SEC trace of LDPE–*g*–PMA (**Fig. 3A**, entry 12, 1,2,4-TCB, 1.0 mL min^{-1} at 150 °C)

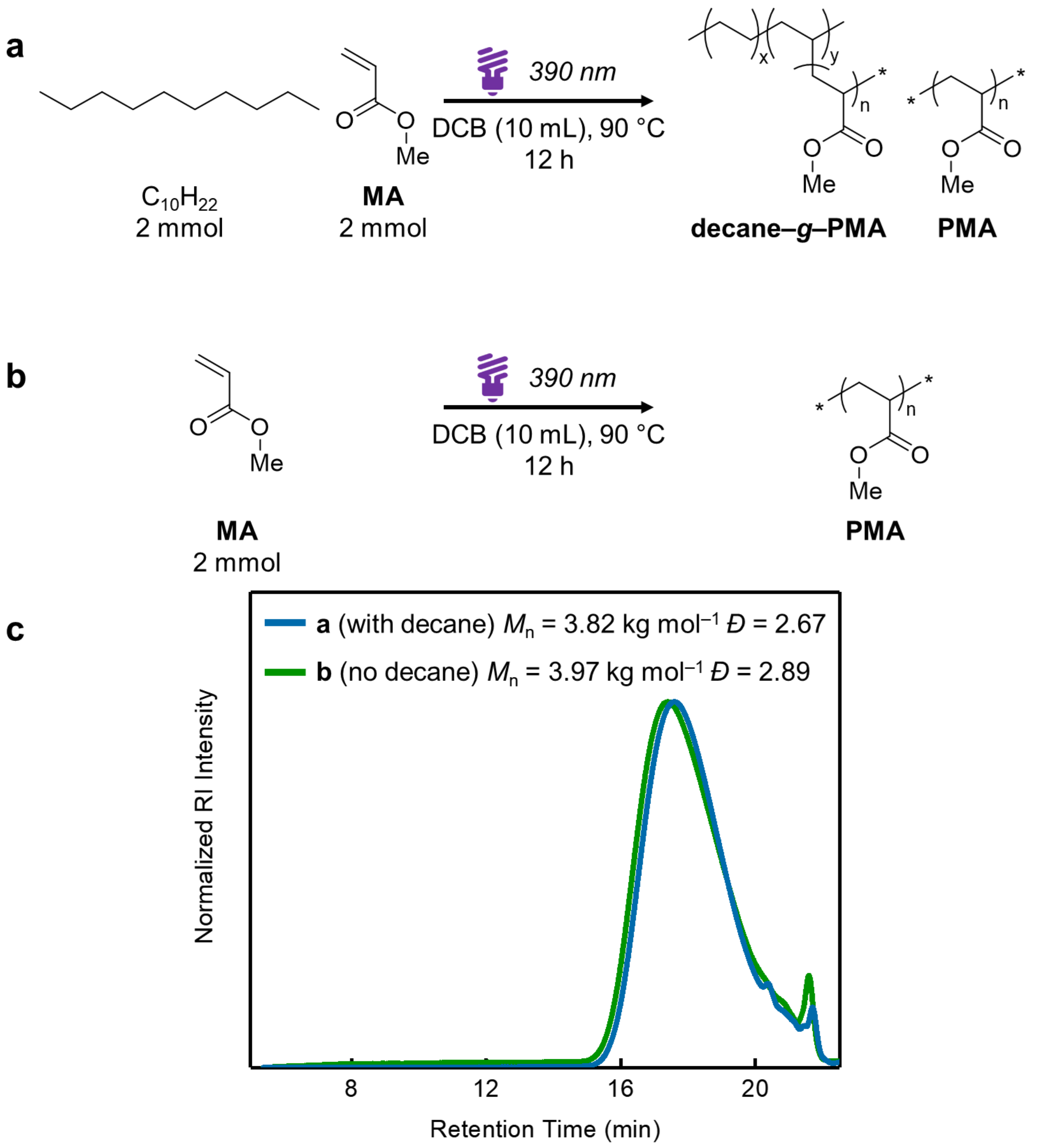

Figure S37. (**A**) Reaction scheme of the decane small-molecule study at 90 °C. (**B**) Control experiment in the absence of decane. (**C**) SEC traces of the two reactions (THF, 1.0 mL min^{-1}, 35 °C).

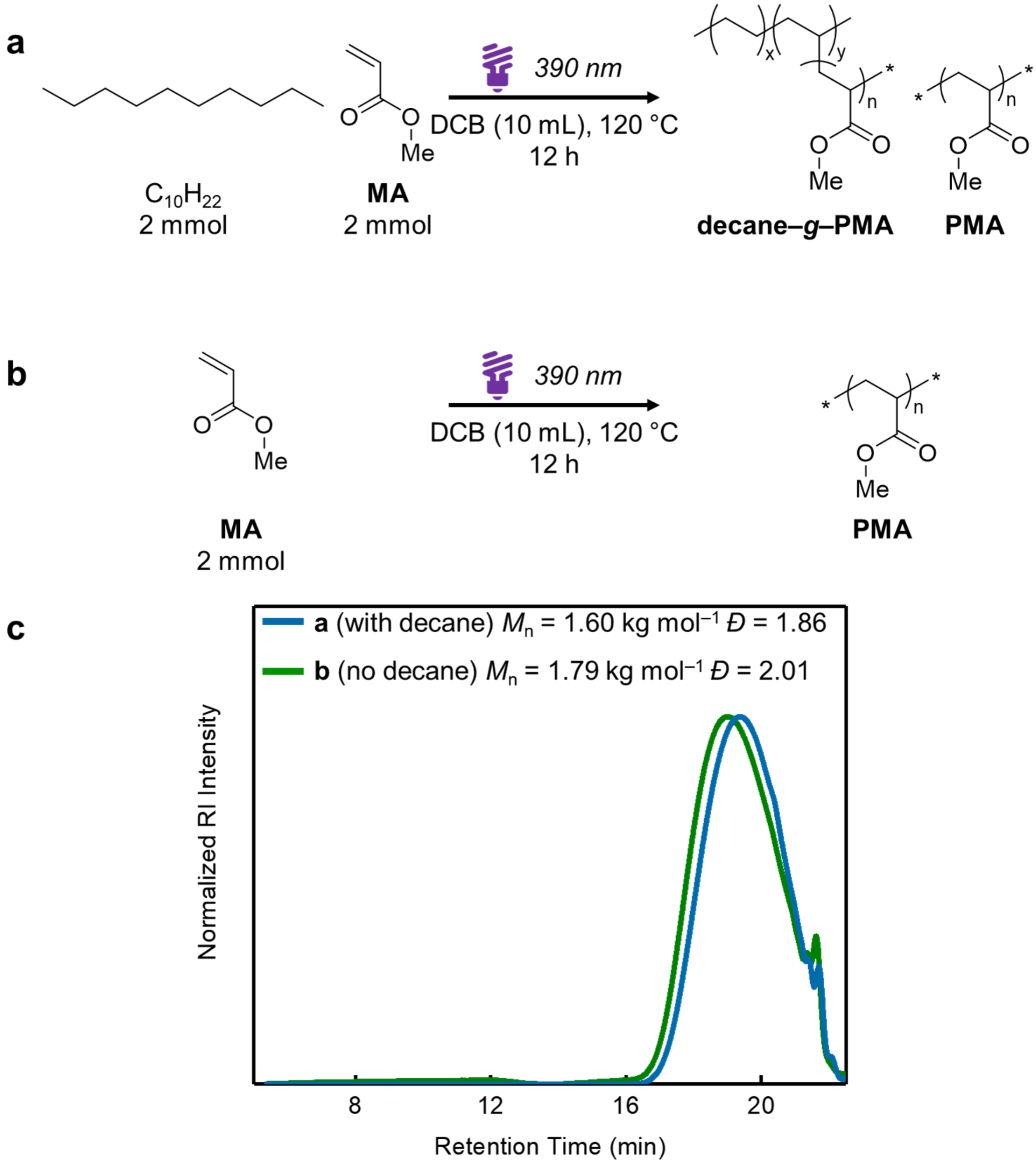

Figure S38. (**A**) Reaction scheme of the decane small-molecule study at 90 °C. (**B**) Control experiment in the absence of decane. (**C**) SEC traces of the two reactions (THF, 1.0 mL min^{-1}, 35 °C).

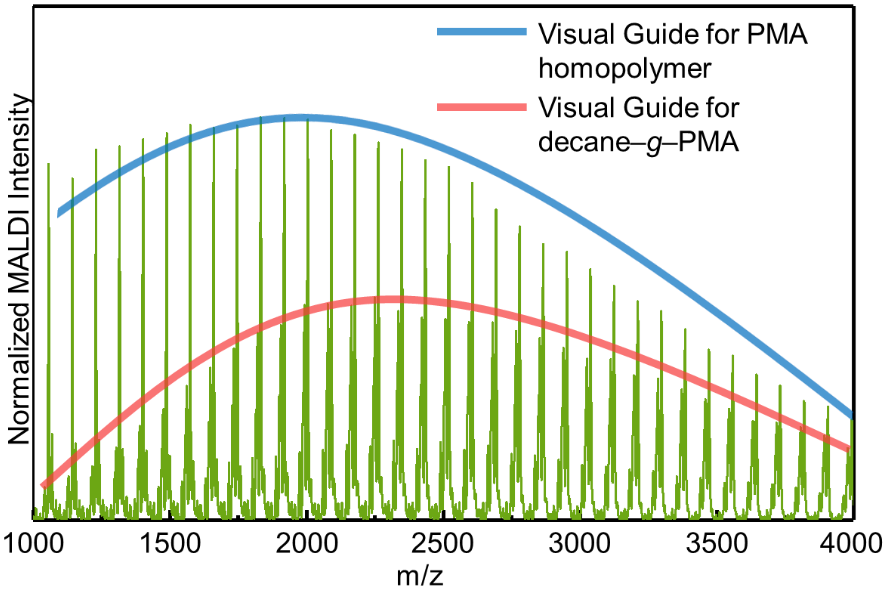

Figure S39. Full MALDI-TOF MS spectrum of a mixture of PMA homopolymer (highlighted in blue) and decane–g–PMA (highlighted in red). Notably, the two major species exhibit similar molar mass distributions.

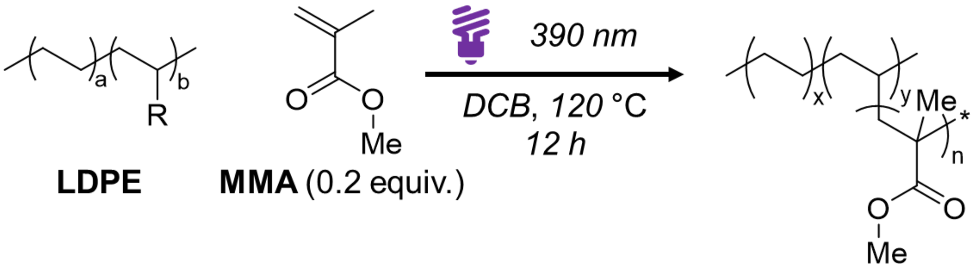

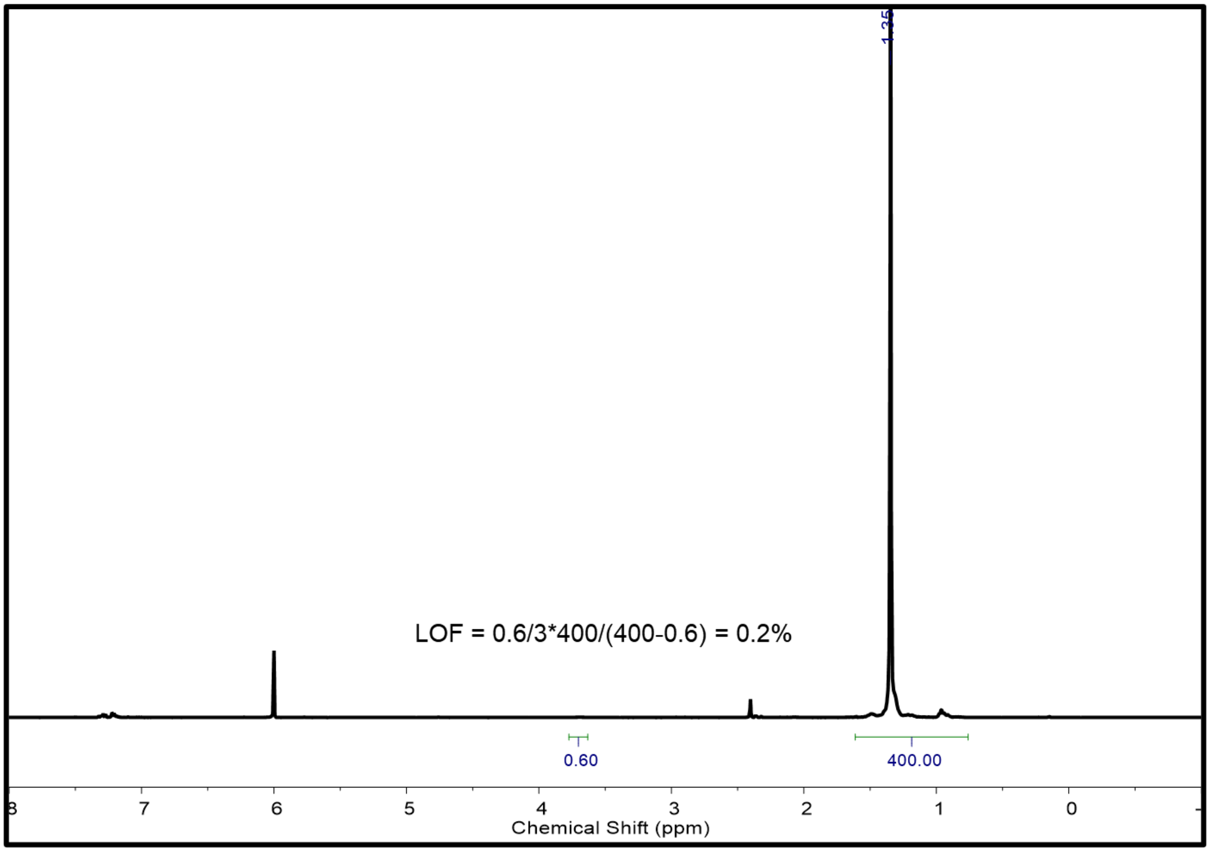

Figure S40. ^{1}H NMR spectrum of LDPE–*g*–PMMA synthesized at 120 °C with 0.2 equiv. of MMA.

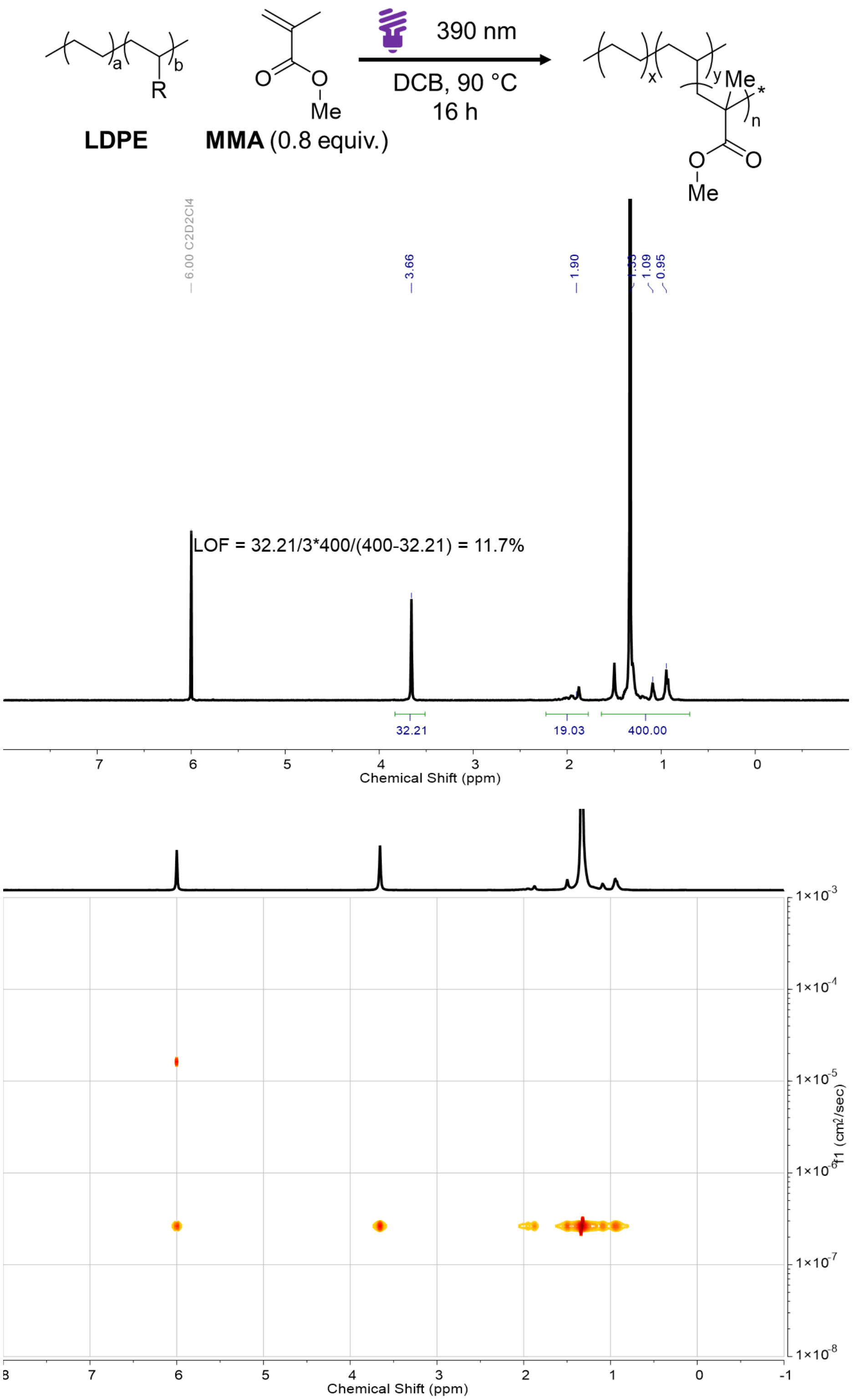

Figure S41. ^{1}H NMR and 2D DOSY NMR spectrum of LDPE–*g*–PMMA synthesized at 90 °C with 0.8 equiv. of MMA.

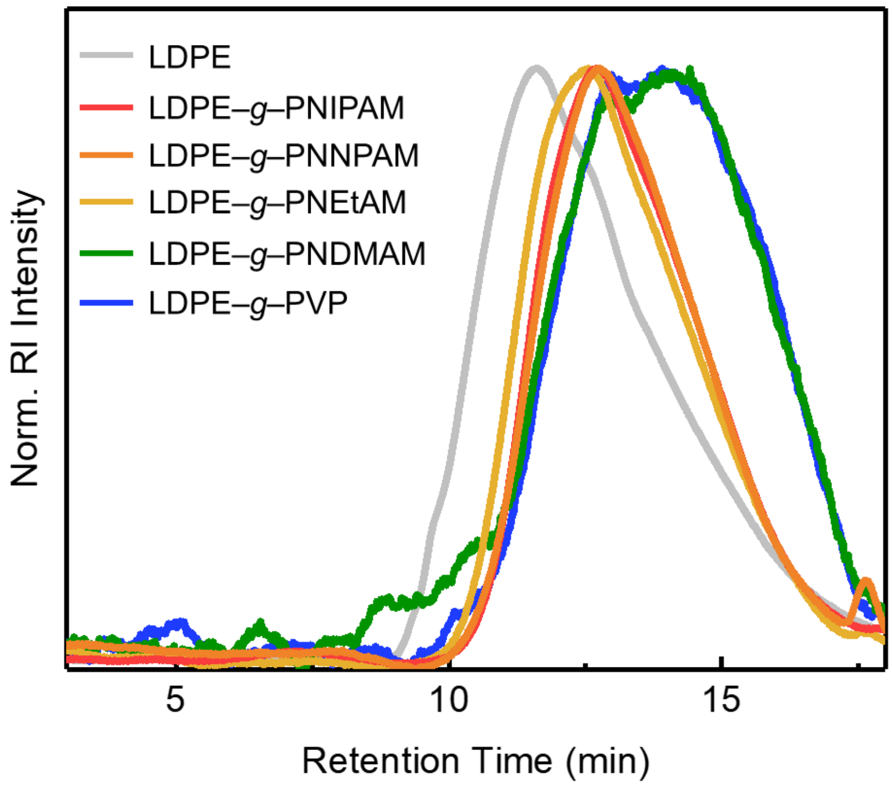

	LOF (%)	M_w (kg mol^{-1})	*Đ*
LDPE	–	296.3	12.1
LDPE–*g*–PNIPAM	6.8	84.3	3.51
LDPE–*g*–PNNPAM	4.3	83.6	2.90
LDPE–*g*–PNEtAM	6.3	106.8	3.24
LDPE–*g*–PNDMAM	10.5	79.2	6.04
LDPE–*g*–PVP	3.1	50.0	3.39

Figure S42. HT SEC traces of polar-polymer grafted LDPEs

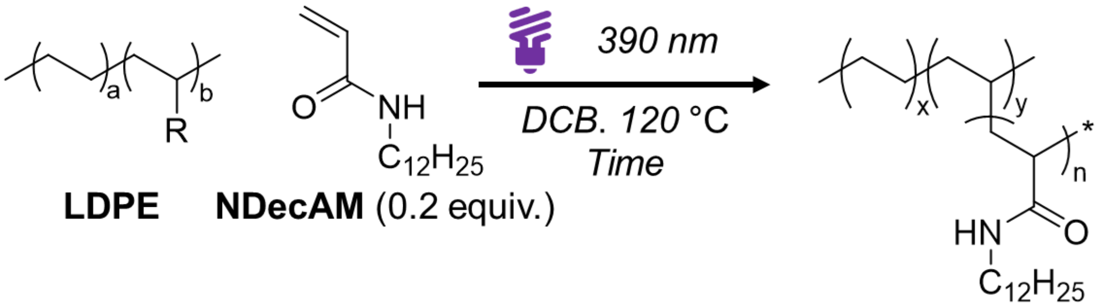

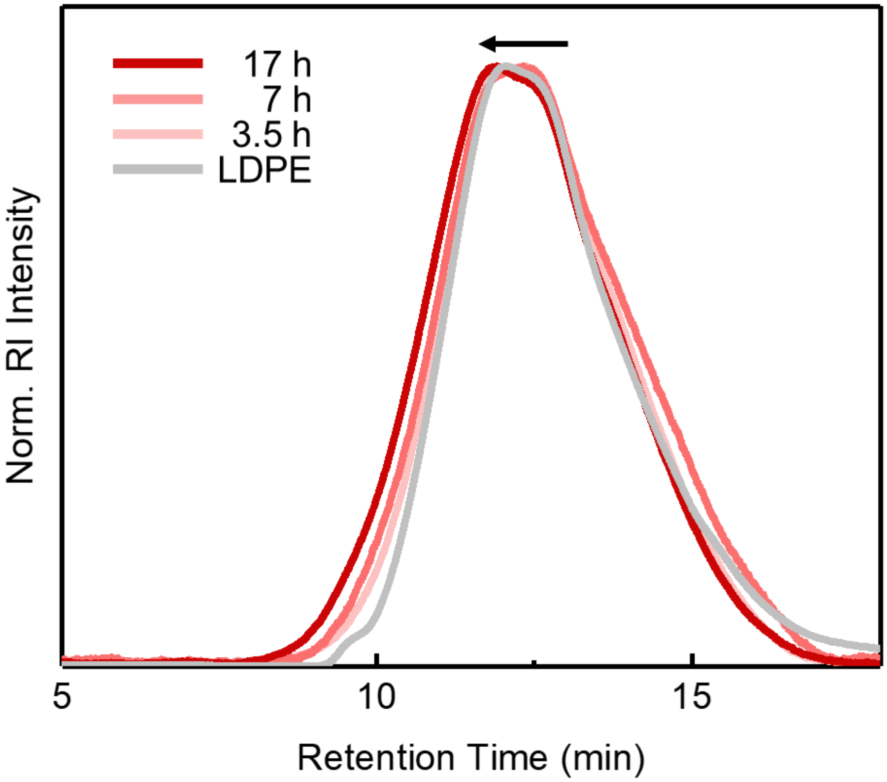

	Reaction Time (h)	LOF (%)	M_w (kg mol^{-1})	$Đ$
LDPE	–	–	161.6	3.70
LDPE–*g*–PNDecAM	3.5	0.9	204.1	5.30
LDPE–*g*–PNDecAM	7	1.2	210.4	5.11
LDPE–*g*–PNDecAM	17	2.4	290.7	7.36

Figure S43. Kinetic HT SEC study using NDecAM as a representative non-polar acrylamide (1,2,4-TCB, 1.0 mL min^{-1} at 150 °C). Unlike polar-polymer-grafted LDPEs, NDecAM exhibited an increase in molecular weight over time. Given its structural similarity to other acrylamides, this result supports that the lower apparent molecular weights of the polar samples originate from changes in hydrodynamic radius rather than from severe chain scission.

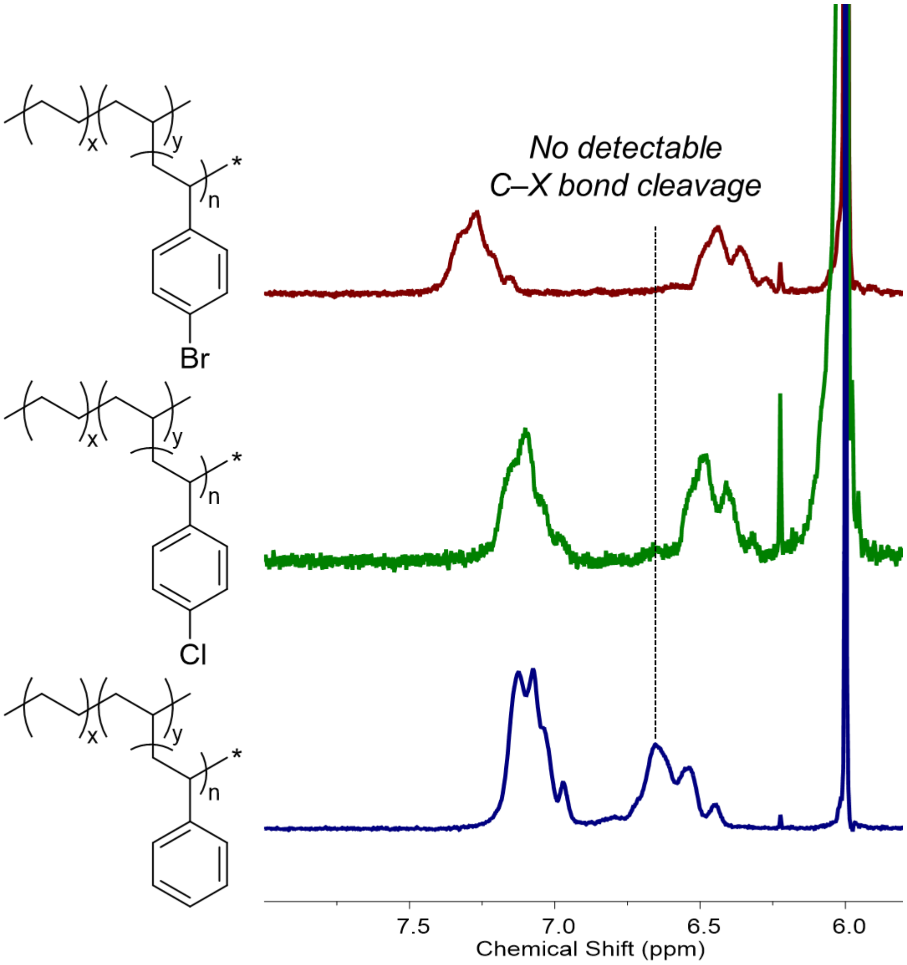

Figure S44. ^{1}H NMR (400 MHz, $C_2D_2Cl_4$) spectra of LDPE–*g*–P(4-BrS) (top), LDPE–*g*–P(4-ClS) (middle), and LDPE–*g*–PS (bottom). (T = 90 °C)

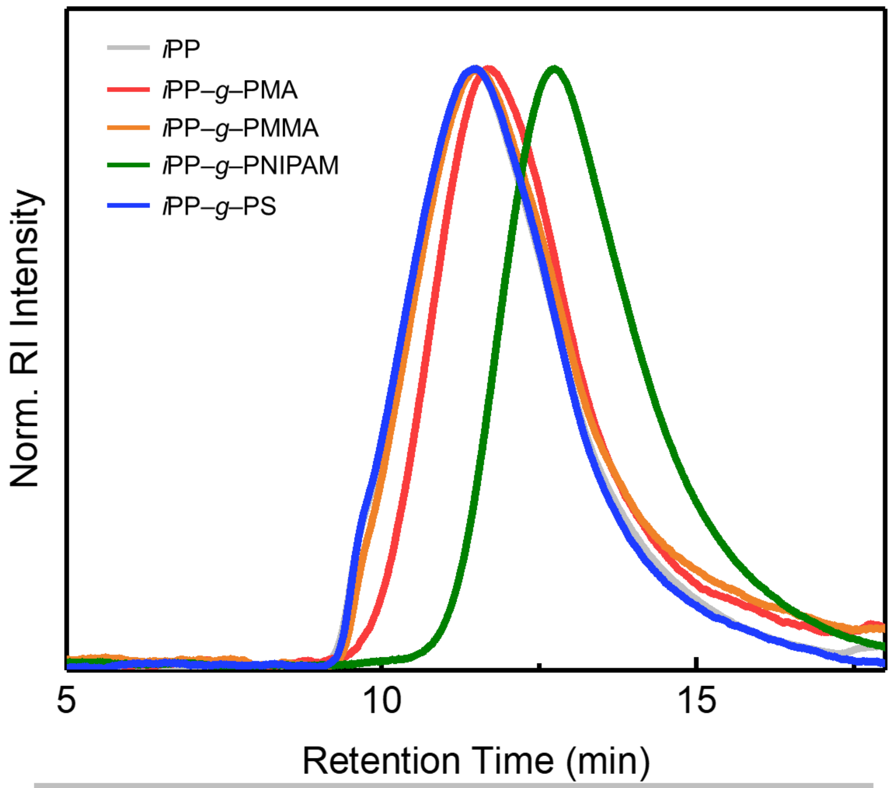

	LOF (%)	M_w (kg mol^{-1})	*Đ*
*i*PP	–	376.4	6.43
*i*PP–*g*–PMA	2.1	216.2	4.20
*i*PP–*g*–PMMA	4.8	313.8	5.36
*i*PP–*g*–PNIPAM	5.3	72.2	2.54
*i*PP–*g*–PS	2.8	383.8	4.96

Figure S45. HT SEC traces of grafted *i*PP samples (1,2,4-TCB, 1.0 mL min^{-1} at 150 °C)

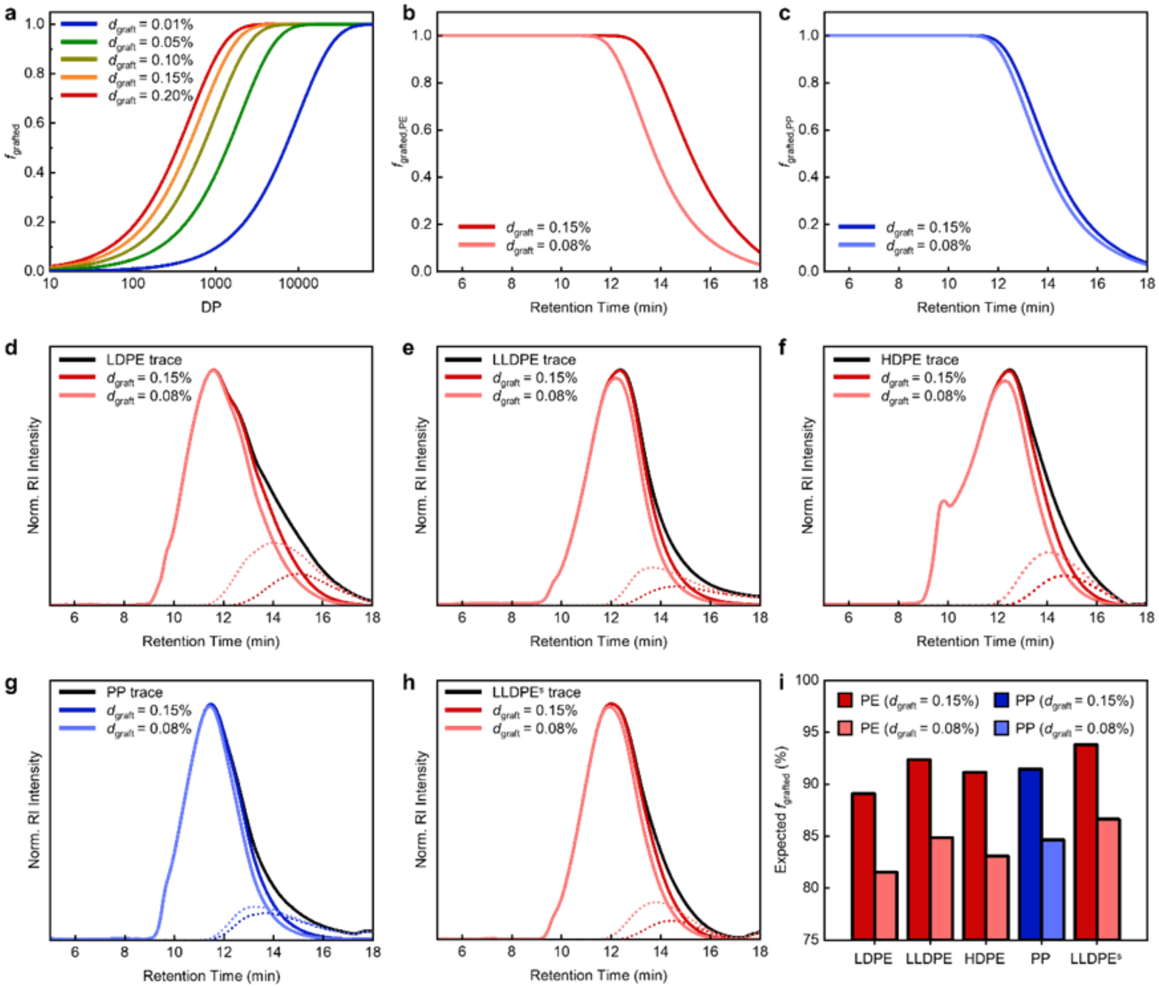

Figure S46. Poisson-distribution-based analysis of graft statistics. (a) Probability of a polymer chain containing at least one graft as a function of degree of polymerization (DP), calculated using a Poisson model at different grafting densities (d_{graft}). (b,c) Corresponding fraction of chains containing at least one graft plotted as a function of retention time, obtained by mapping DP to molecular weight via column calibration for (b) PE and (c) PP. (d–h) High-temperature SEC traces of LDPE, LLDPE, HDPE, PP, and toluene-Soxhlet-purified LLDPE (black), overlaid with simulated grafted (solid) and ungrafted (dotted) contributions calculated by weighting the experimental RI signal with the Poisson-derived probability of at least one graft. Light-colored traces correspond to a graft density of 0.08%, whereas dark-colored traces correspond to a graft density of 0.15%. Importantly, the statistically ungrafted contribution is confined to the low-molecular-weight (high-retention-time) tail of the molecular weight distribution, whereas the high-molecular-weight region is overwhelmingly dominated by chains expected to contain at least one graft. (i) Summary of the expected mass fraction of polymer chains containing at least one graft, showing a maximum value of 94% for purified LLDPE at a graft density of 0.15%.

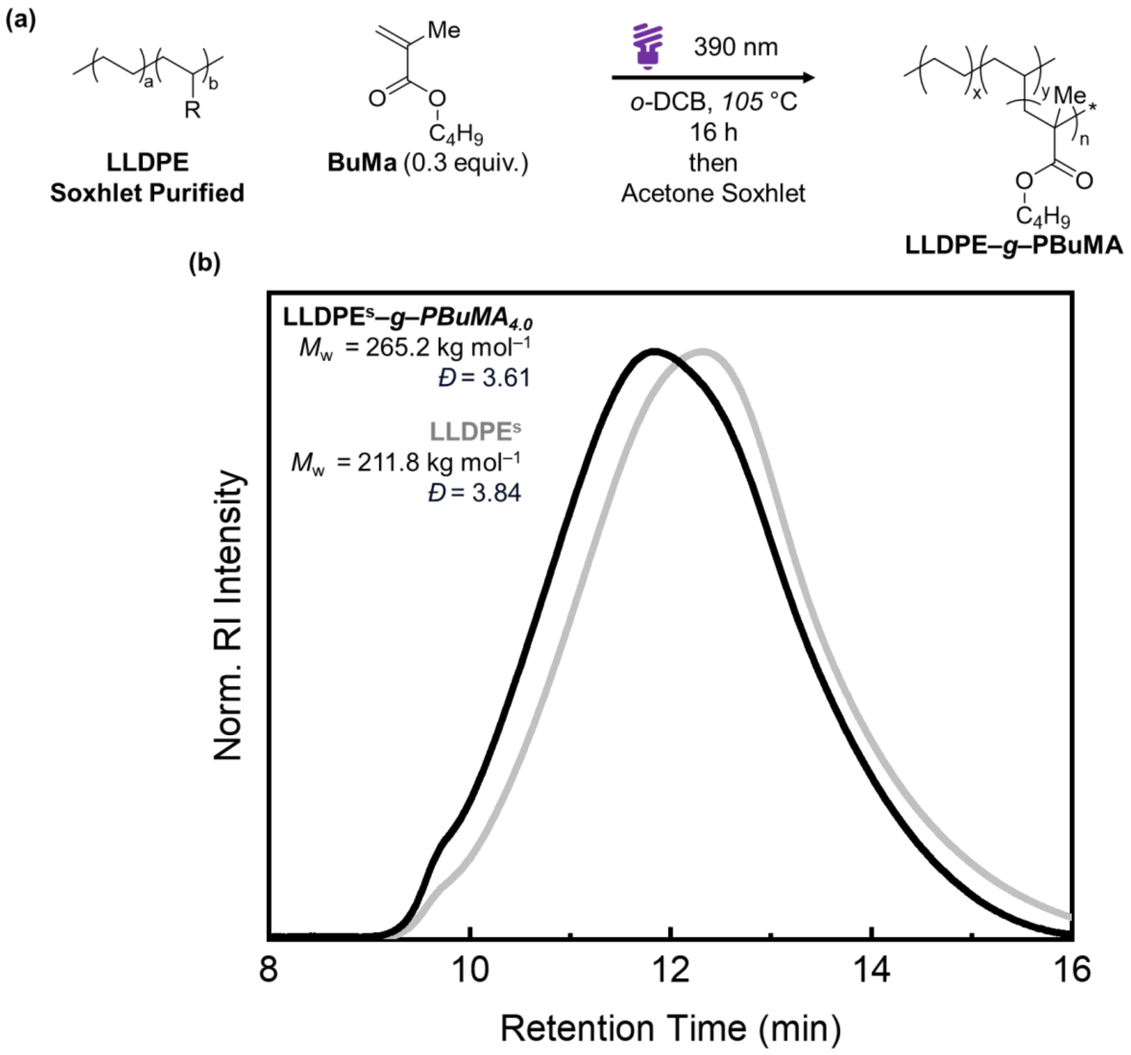

Figure S47. Experimental verification of the statistical grafting model using LLDPE. (a) Reaction scheme for the visible-light-mediated grafting of PBuMA from toluene-Soxhlet-purified LLDPE. (b) High-temperature SEC traces of toluene-Soxhlet-purified LLDPE before (gray) and after (black) grafting with PBuMA after 16 h of reaction.

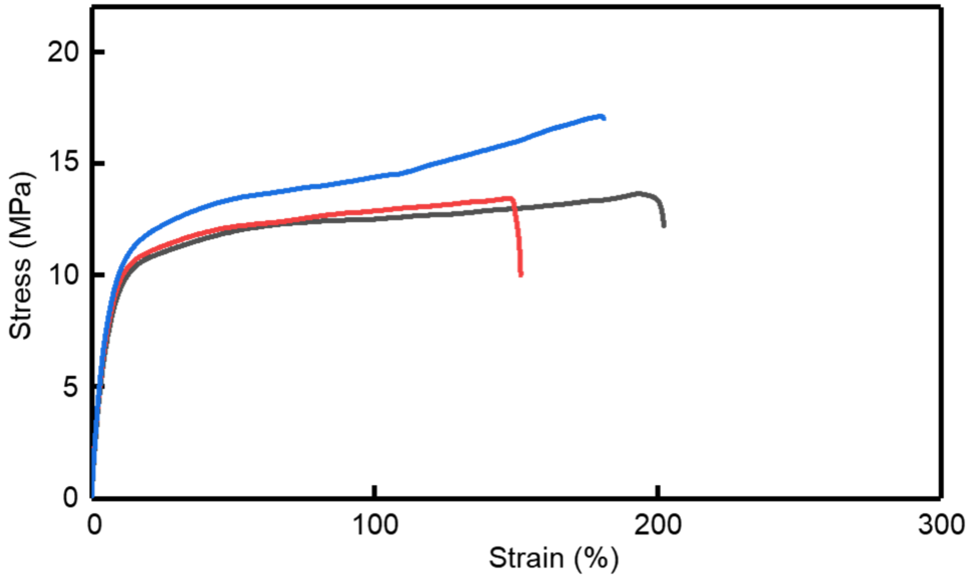

	E (MPa)	σ_y (MPa)	σ_u (MPa)	ε_{break} (%)
1	247	10.8	13.5	193
2	265	10.4	13.3	147
3	257	11.8	17.0	180
Average	256±9	11.0±0.7	14.6±2.1	173±23

Figure S48. Stress–strain curves for **LDPE–*g*–PMMA$_{5.5}$**

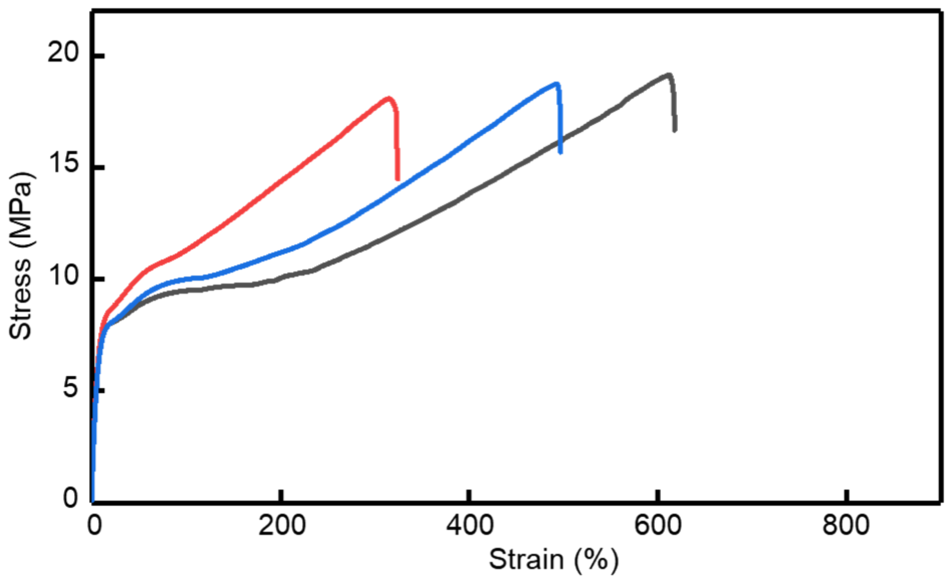

	E (MPa)	σ_y (MPa)	σ_u (MPa)	ε_{break} (%)
1	169	7.8	19.1	613
2	161	8.4	18.0	315
3	159	7.8	18.7	493
Average	163±5	8.0±0.4	18.6±0.5	474±150

Figure S49. Stress–strain curves for **LDPE–*g*–PS**$_{3.7}$

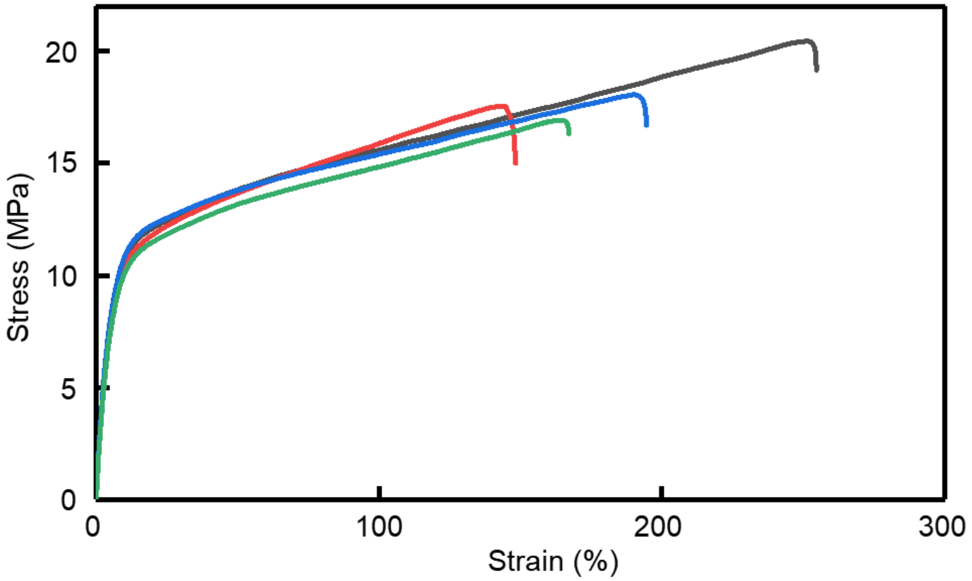

	E (MPa)	σ_y (MPa)	σ_u (MPa)	ε_{break} (%)
1	246	11.8	20.4	251
2	243	11.7	17.5	144
3	263	11.9	18.0	190
4	233	11.3	16.9	164
Average	246±12	11.7±0.3	18.2±1.5	187±47

Figure S50. Stress–strain curves for **LDPE–*g*–PNIPAM$_{6.8}$**

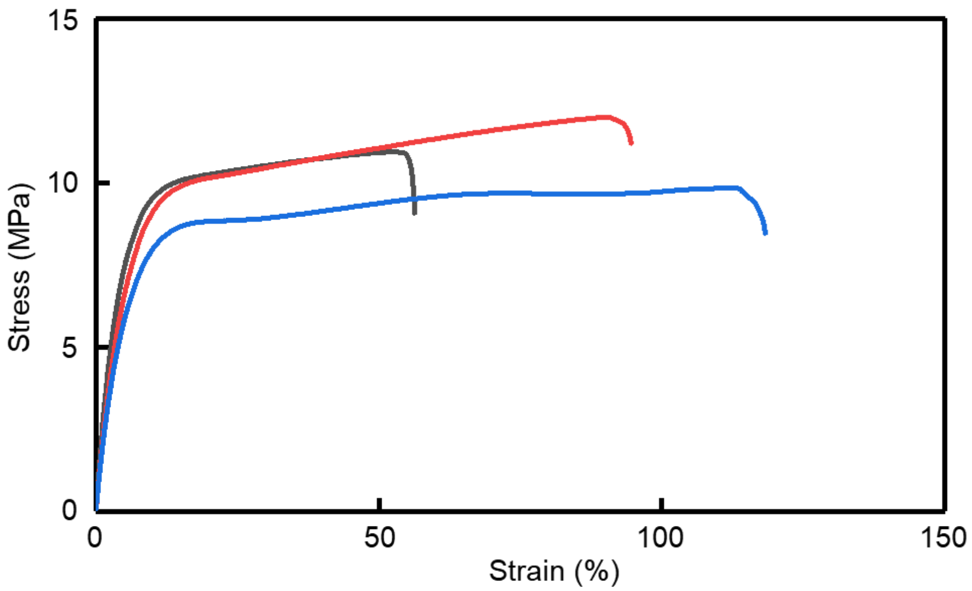

	E (MPa)	σ_y (MPa)	σ_u (MPa)	ε_{break} (%)
1	259	9.7	10.8	53
2	225	9.7	11.9	90
3	213	9.0	9.8	112
Average	232±24	9.5±0.4	10.8±1.1	85±30

Figure S51. Stress–strain curves for **LDPE–*g*–PNNPAM$_{4.3}$**

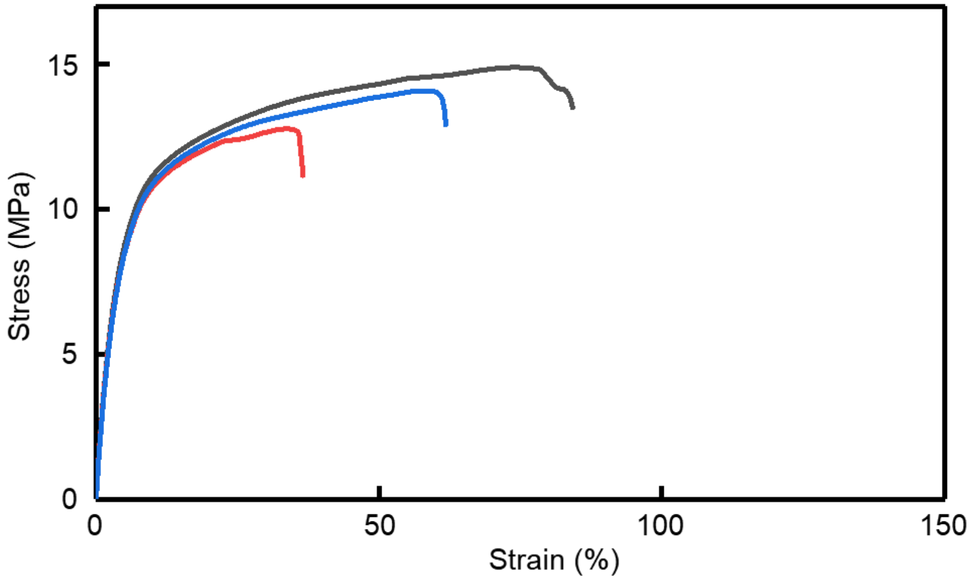

	E (MPa)	σ_y (MPa)	σ_u (MPa)	ε_{break} (%)
1	318	11.6	14.8	74
2	316	11.2	12.7	34
3	301	11.4	14.0	58
Average	312±9	11.4±0.2	13.8±1.1	55±20

Figure S52. Stress-strain curves for **LDPE–*g*–PNEtAM$_{6.5}$**

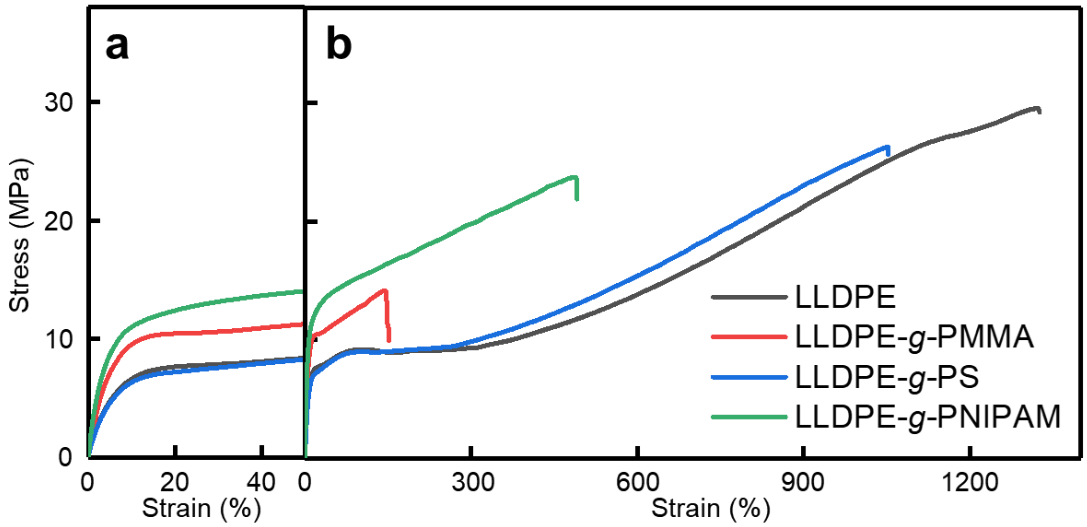

	LOF (%)	E (MPa)	σ_y (MPa)	σ_u (MPa)	ε_{break} (%)
LLDPE–g–PMMA	6.7	264±17	10.2±0.2	12.6±2.1	100±42
LLDPE–g–PS	1.9	159±4	7.0±0.1	26.6±1.4	1041±112
LLDPE–g–PNIPAM	6.4	361±14	12.1±0.3	21.2±2.8	394±113
LLDPE	-	187±4	7.4±0.2	28.9±0.7	1365±40

Figure S53. (**a**) Enlarged view and (**b**) full Stress–strain curves of reinforced LLDPE samples

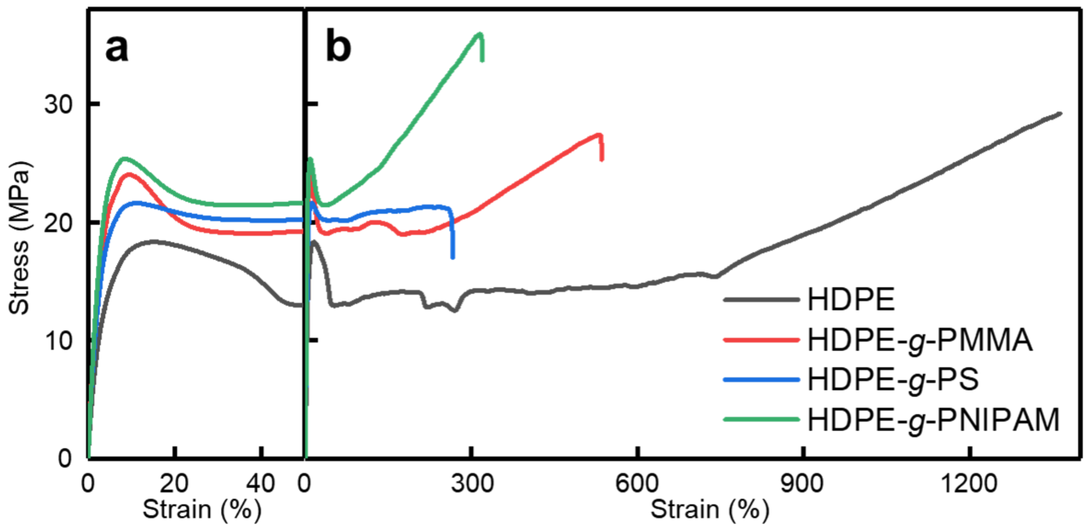

	LOF (%)	E (MPa)	σ_y (MPa)	σ_u (MPa)	ε_{break} (%)
HDPE–g–PMMA	5.8	924±8	23.9±1.2	26.3±2.7	496±173
HDPE–g–PS	4.1	820±76	21.7±0.9	23.7±2.2	104±101
HDPE–g–PNIPAM	6.8	856±18	25.6±0.5	33.5±3.8	262±46
HDPE	-	667±18	17.8±0.2	30.2±0.8	1409±33

Figure S54. (**a**) Enlarged view and (**b**) full Stress–strain curves of reinforced HDPE samples

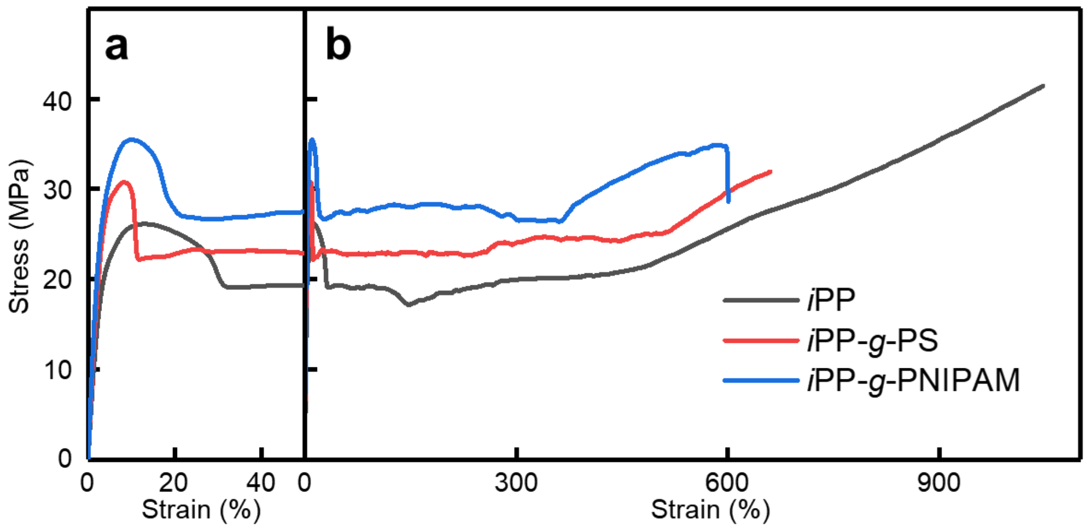

	LOF (%)	*E* (MPa)	σ_y (MPa)	σ_u (MPa)	ε_{break} (%)
*i*PP–g–PS	2.8	962±116	28.1±2.6.	31.5±7.8	579±198
*i*PP–g–PNIPAM	5.3	1201±45	34.2±1.4	37.2±2.3	677±147
*i*PP	-	849±35	25.3±0.8	43.9±2.3	1094±69

Figure S55. (**a**) Enlarged view and (**b**) full Stress–strain curves of reinforced *i*PP samples

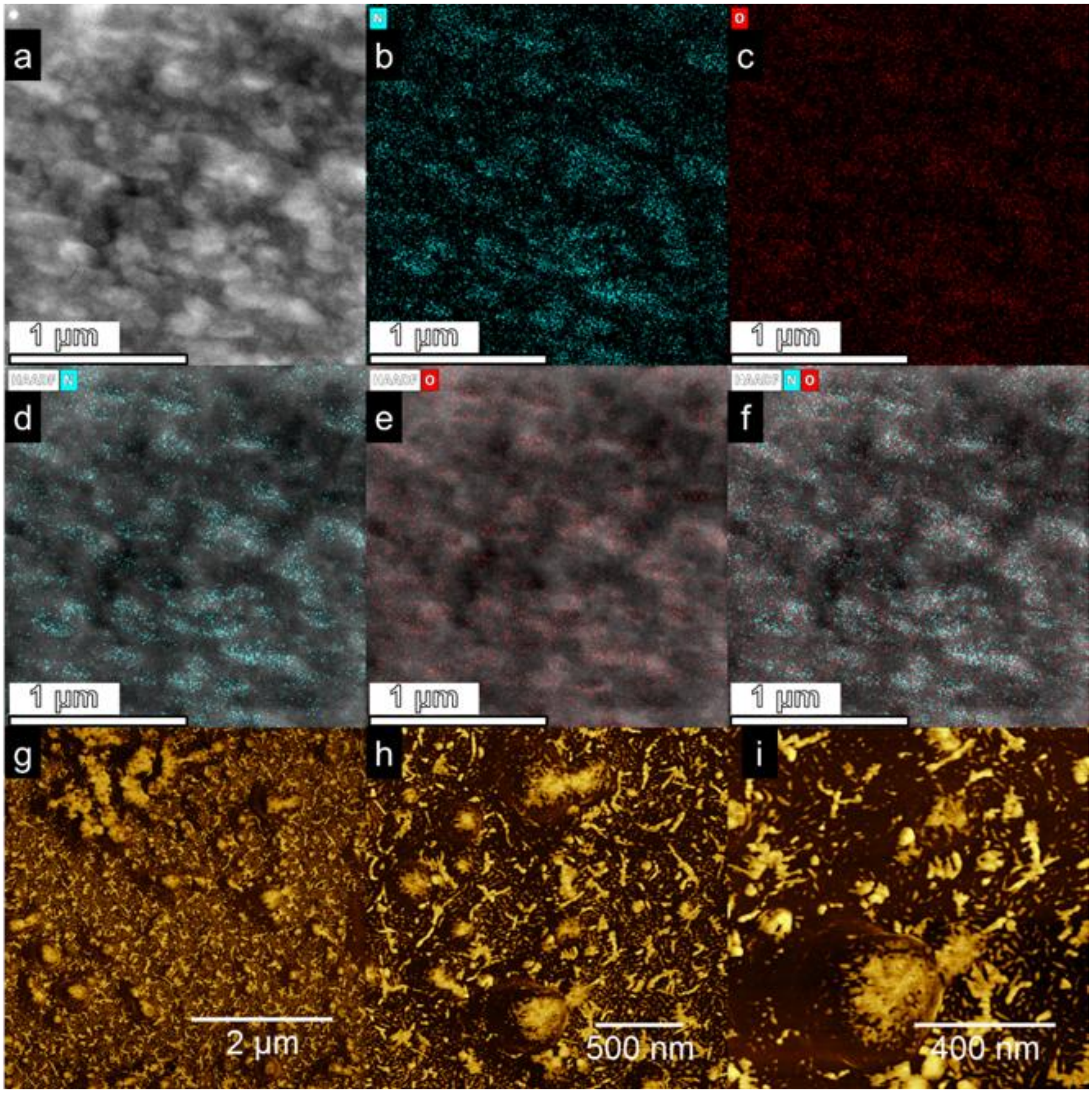

Figure S56. (a–f) Transmission electron microscopy (TEM) and energy-dispersive X-ray spectroscopy (EDX) analyses of LDPE–*g*–PNEtAM. (**a**) Bright-field TEM image. (**b**) Nitrogen elemental EDX map. (**c**) Oxygen elemental EDX map. (**d**) Overlay of the TEM image and the N EDX map. (**e**) Overlay of the TEM image and the O EDX map. (**f**) Overlay of the TEM image with N and O EDX maps. (**g–i**) Phase-mode AFM images at different magnifications.

Table S1. Summary of melting enthalpies.

	Molar Mass (g mol⁻¹)[a]	LOF (%)	f_{weight}[b]	$\Delta H_{m,obs.}$ (J g⁻¹)[c]	$X_{C,obs.}$ (%)[d]	$\Delta H_{m,norm.}$ (J g⁻¹)[e]	$X_{C\cdot norm.}$ (%)[f]
LDPE	-	-	-	94.53	32.3	-	-
LDPE–g–PMA	86.09	3.9	0.107	82.84	28.3	92.8	31.7
LDPE–g–PDEGA	174.3	1.5	0.085	69.82	23.8	76.3	26.0
LDPE–g–PMMA	100.13	5.3	0.159	76.30	26.0	90.7	31.0
LDPE–g–PNIPAM	113.16	6.8	0.215	60.71	20.7	77.4	26.4
LDPE–g–PNNPAM	113.16	4.3	0.148	81.66	27.9	95.8	32.7
LDPE–g–PNEtAM	99.15	6.3	0.182	60.71	20.7	74.2	25.3
LDPE–g–PDMAM	99.15	10.5	0.271	62.28	21.3	85.4	29.1
LDPE–g–PVA	86.09	2.6	0.074	76.71	26.2	82.8	28.3
LDPE–g–PVP	111.14	3.1	0.109	78.69	26.9	88.3	30.2
LDPE–g–PS	104.15	3.7	0.121	80.71	27.5	91.8	31.3
LDPE–g–P(4-ClS)	138.59	1.6	0.073	87.02	29.7	93.9	32.0
LDPE–g–P(4-BrS)	183.05	0.9	0.055	92.00	31.4	97.4	33.2
LLDPE	-	-	-	88.08	30.1	-	-
LLDPE–g–PMA	86.09	2.7	0.077	70.12	23.9	75.9	25.9
LLDPE–g–PMMA	100.13	6.7	0.193	69.40	23.7	86.0	29.4
LLDPE–g–PNIPAM	113.16	6.4	0.205	56.16	19.2	70.7	24.1
LLDPE–g–PS	104.15	1.9	0.066	84.51	28.8	90.5	30.9
HDPE	-	-	-	156.27	53.3	-	-
HDPE–g–PMA	86.09	2.8	0.079	132.88	45.4	144.3	49.2
HDPE–g–PMMA	100.13	5.8	0.172	137.52	46.9	166.0	56.7
HDPE–g–PNIPAM	113.16	6.8	0.215	107.23	36.6	136.6	46.6
HDPE–g–PS	104.15	4.1	0.132	149.17	50.9	171.9	58.7
*i*PP	-	-	-	98.55	47.6	-	-
HDPE–g–PMA	86.09	2.1	0.048	79.24	38.3	83.2	40.2
HDPE–g–PMMA	100.13	4.8	0.106	78.99	38.2	88.4	42.7
HDPE–g–PNIPAM	113.16	5.3	0.125	71.10	34.3	81.2	39.2
HDPE–g–PS	104.15	2.8	0.070	83.41	40.3	89.7	43.3
Plastic Bag	-	-	-	92.95	31.7	-	-
Plastic Bag–g–PMA	86.09	3.7	0.102	79.45	27.1	88.5	30.2
Plastic Bottle	-	-	-	156.89	53.5	-	-
Plastic Bottle–g–PMA	86.09	2.6	0.074	135.02	46.1	145.8	49.8
Falcon Tube	-	-	-	67.75	32.7	-	-
Falcon Tube–g–PMA	86.09	1.7	0.034	60.45	29.2	62.6	30.2

[a] Molar mass of the repeat unit of the grafted polymers.
[b] Weight fraction of grafted polymers, calculated from repeat unit molar masses and f_{vinyl}s.
[c] Melting enthalpies determined by DSC.
[d] Crystallinity calculated by dividing the melting enthalpy by the theoretical value for 100% crystalline material (293 J/g for PE; 207 J/g for PP).
[e] Normalized melting enthalpies obtained by dividing the melting enthalpy by (1–f_{weight}).
[f] Crystallinity derived from the normalized melting enthalpies.

Table S2. Summary of lap shear test. Curing time = 1 hour. Temperature = 190 °C

	LOF (%)	Substrate	Adhesion Strength (MPa)	Failure mode
LDPE	-	Aluminum	*fail*	-
EVA	-	Aluminum	1.59 ± 0.08	Cohesive
Gorilla Glue	-	Aluminum	2.63 ± 0.30	Cohesive
LDPE–*g*–PMMA	5.5	Aluminum	5.18 ± 0.45	Adhesive
LDPE–*g*–PNIPAM	3.8	Aluminum	6.04 ± 1.10	Adhesive
LDPE–*g*–PNNPAM	4.3	Aluminum	9.84 ± 1.23	Cohesive
LDPE–*g*–PNEtAM	6.3	Aluminum	12.70 ± 0.62[a]	Cohesive
LDPE–*g*–PNEtAM	6.3	Stainless Steel	8.79 ± 2.08	Cohesive
LDPE–*g*–PNEtAM	6.3	Copper	10.87 ± 0.83	Cohesive
LDPE–*g*–PNEtAM	6.3	Wood	10.24 ± 0.79	Cohesive

[a]Maximum value of 13.4 MPa among five specimens.

Table S3. Results of curing time screening for LDPE–*g*–PNEtAM$_{6.3}$. Temperature = 190 °C

	LOF (%)	Substrate	Curing time (min)	Adhesion Strength (MPa)	Failure mode
LDPE–*g*–PNEtAM	6.3	Aluminum	15	7.61 ± 0.50	Adhesive
LDPE–*g*–PNEtAM	6.3	Aluminum	30	8.13 ± 1.34	Adhesive
LDPE–*g*–PNEtAM	6.3	Aluminum	60	12.70 ± 0.62	Cohesive
LDPE–*g*–PNEtAM	6.3	Aluminum	90	12.12 ± 0.46	Cohesive

Table S4. Results of curing temperature screening for LDPE–*g*–PNEtAM$_{6.3}$. Curing Time = 60 min

	LOF (%)	Substrate	Curing Temperature (°C)	Adhesion Strength (MPa)	Failure mode
LDPE–*g*–PNEtAM	6.3	Aluminum	130	6.53 ± 1.47	Adhesive
LDPE–*g*–PNEtAM	6.3	Aluminum	160	10.04 ± 0.26	Adhesive
LDPE–*g*–PNEtAM	6.3	Aluminum	190	12.47 ± 0.32	Cohesive
LDPE–*g*–PNEtAM[a]	6.3	Aluminum	190	10.92 ± 1.20	Cohesive

[a] Measurements were conducted after the specimens were immersed in water for 10 min.

Reaction Procedures and Material Characterization

Synthesis of LDPE–*g*–PMA (Figure 2a). A 22 mL borosilicate test tube was charged with LDPE (281 mg, 10 mmol) and 1,2-dichlorobenzene (10 mL), and sealed with a rubber septum. The mixture was degassed by nitrogen bubbling at 120 °C until it became homogeneous. Methyl acrylate (0.3 equiv., 0.27 mL, 3 mmol) was added via syringe. The reaction mixture was stirred and irradiated with a 390 nm LED lamp for 6 hours at 120 °C. Upon completion of the reaction, the mixture was precipitated in cold methanol (ca. 40 mL). The resulting solid was collected by filtration and washed via Soxhlet extraction with acetone for 6 h (ca. 4 min per cycle). After the purification, the solid was dried in a vacuum oven (3 mbar) at 80 °C for 12 h. to afford the desired **LDPE–*g*–PMA** as a white solid (282 mg). The product was characterized by ^{1}H NMR (90 °C in $C_2D_2Cl_4$, 400 MHz), and DSC. Isolated yield = 53%; Graft ratio = 10.7%

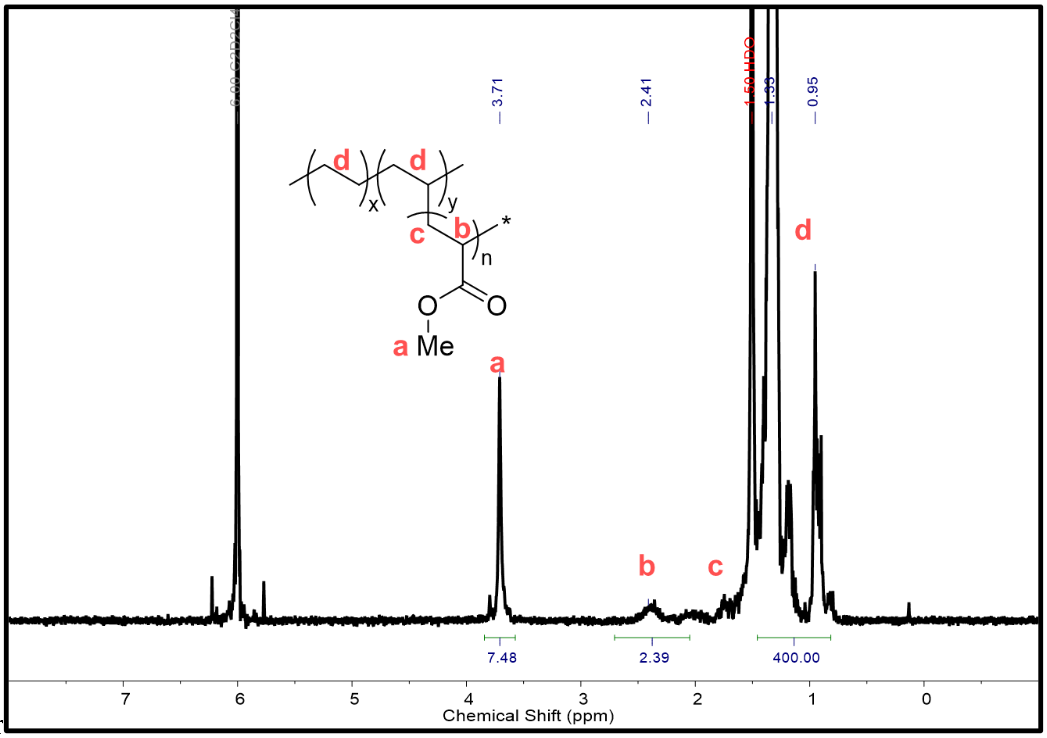

Figure S57. ^{1}H NMR spectrum (400 MHz, $C_2D_2Cl_4$) of LDPE–*g*–$PMA_{2.5}$ (T = 90 °C)

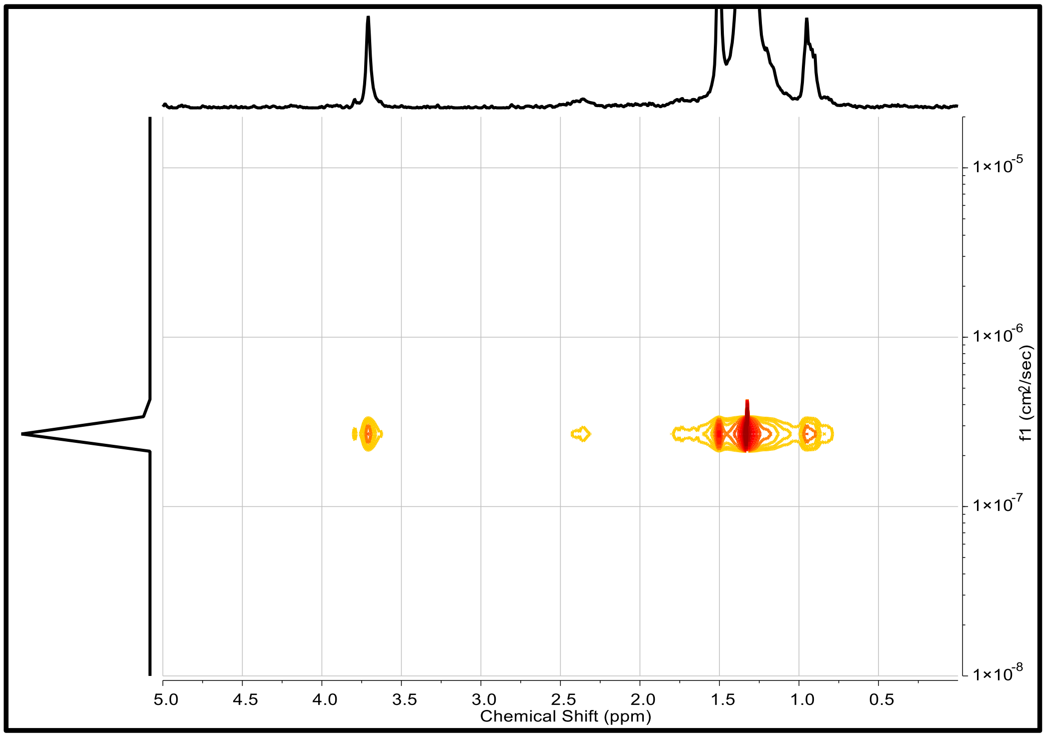

Figure S58. 2D DOSY NMR spectrum (400 MHz, $C_2D_2Cl_4$) of LDPE–*g*–$PMA_{2.5}$ (T = 90 °C)

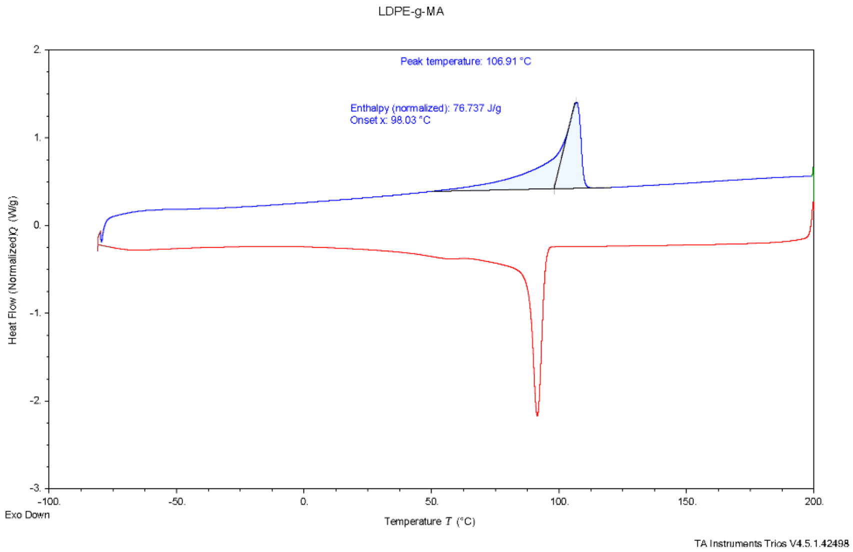

Figure S59. DSC Thermogram of LDPE–*g*–PMA (f_{vinyl} = 2.5%). T_m = 106.91 °C, ΔH_m = 76.737 J g^{-1}, X_C = 26.2%.

Synthesis of LDPE–*g*–PDEGA (Figure 3e). A 22 mL borosilicate test tube was charged with LDPE (281 mg, 10 mmol) and 1,2-dichlorobenzene (10 mL), and sealed with a rubber septum. The mixture was degassed by nitrogen bubbling at 120 °C until it became homogeneous. Di(ethylene glycol) ethyl ether acrylate (0.3 equiv., 0.56 mL, 3 mmol) was added via syringe. The reaction mixture was stirred and irradiated with a 390 nm LED lamp for 6 hours at 120 °C. Upon completion of the reaction, the mixture was precipitated in cold methanol (ca. 40 mL). The resulting solid was collected by filtration and washed via Soxhlet extraction with acetone for 6 h (ca. 4 min per cycle). After the purification, the solid was dried in a vacuum oven (3 mbar) at 80 °C for 12 h. to afford the desired **LDPE–*g*–PDEGA** as a white solid (278 mg). The product was characterized by ^{1}H NMR (90 °C in $C_2D_2Cl_4$, 400 MHz), 2D DOSY NMR, and DSC. Isolated yield = 34%; Graft yield = 5.3%

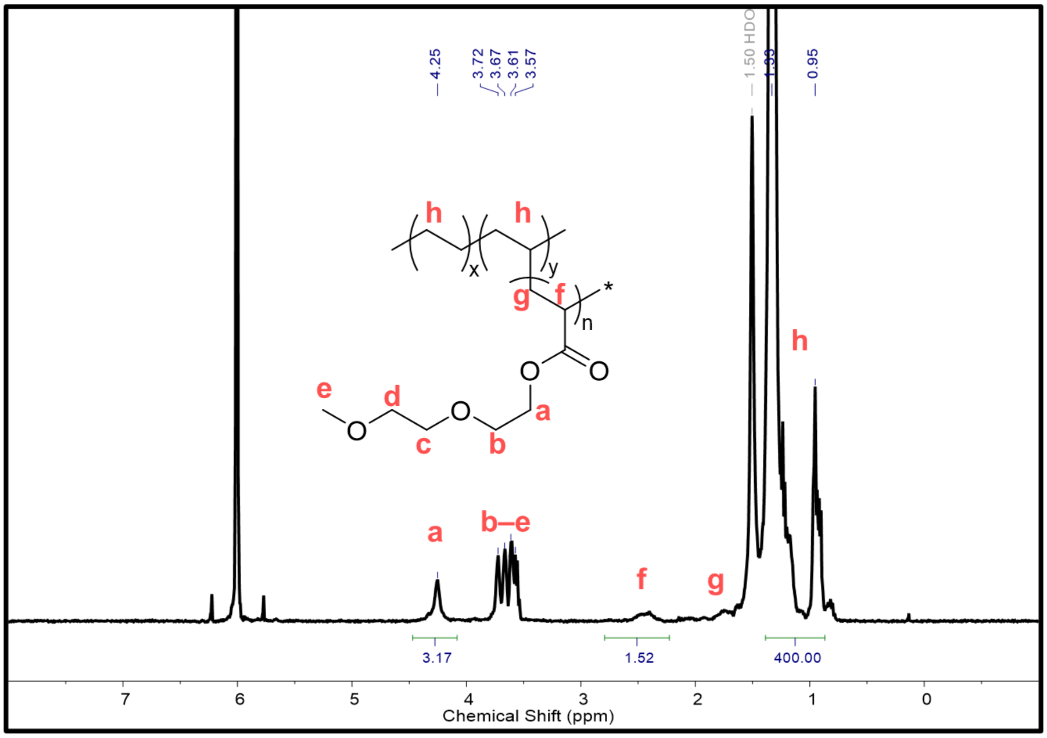

Figure S60. ^{1}H NMR spectrum (400 MHz, $C_2D_2Cl_4$) of LDPE–*g*–PDEGA$_{1.6}$ (T = 90 °C)

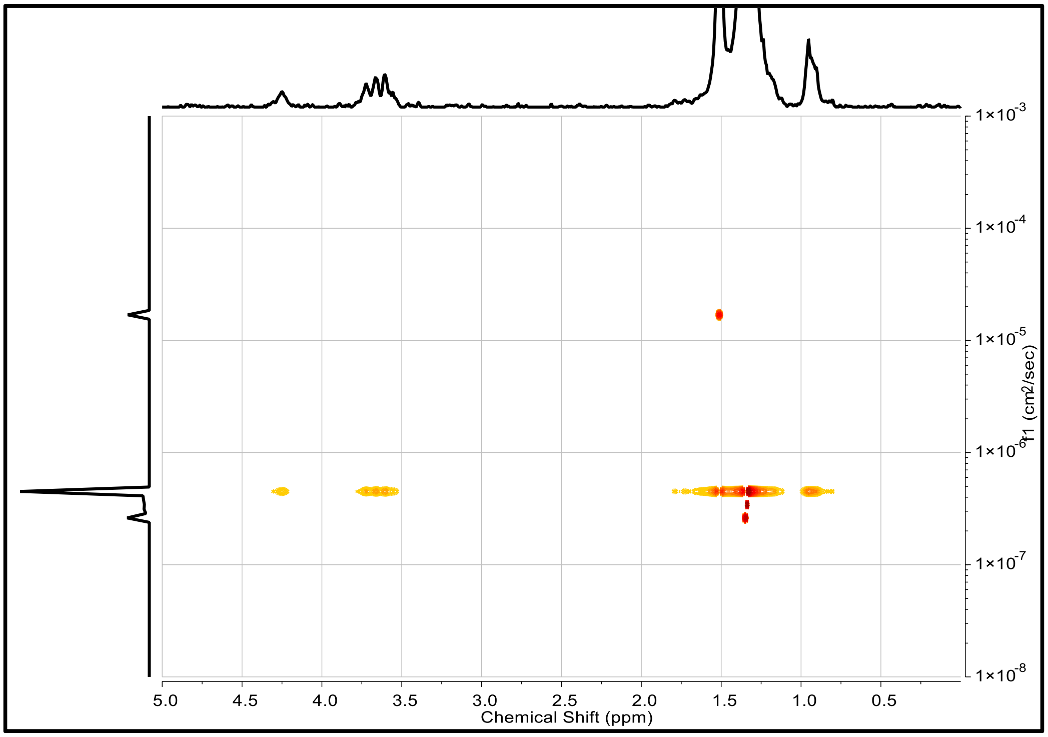

Figure S61. 2D DOSY NMR spectrum (400 MHz, $C_2D_2Cl_4$) of LDPE–*g*–$PDEGA_{1.6}$ (T = 90 °C)

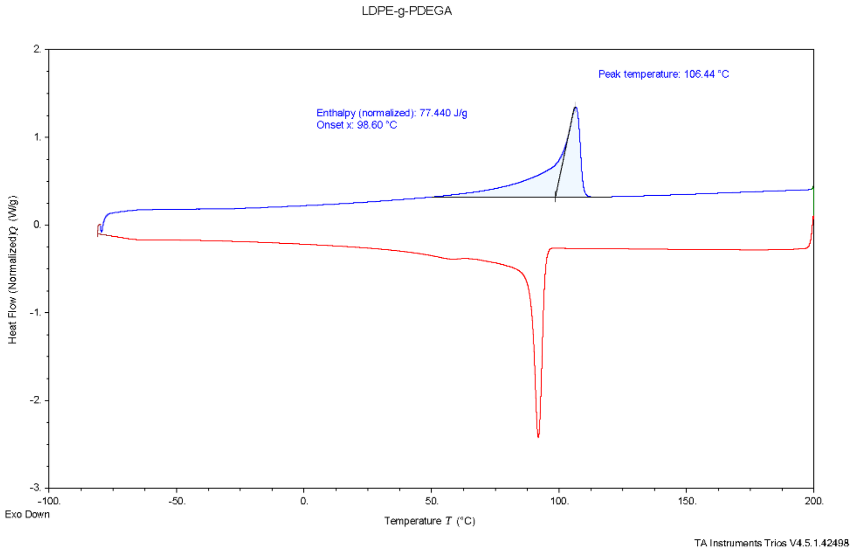

Figure S62. DSC Thermogram of LDPE–*g*–PDEGA (f_{vinyl} = 1.6%). T_m = 106.44 °C, ΔH_m = 77.440 J g^{-1}, X_C = 26.4%.

Synthesis of LDPE–g–MA (Figure 2b). A 22 mL borosilicate test tube was charged with LDPE (281 mg, 10 mmol), tetrabutylammonium iron (III) tetrachloride (TBAFeCl_4) and 1,2-dichlorobenzene (10 mL), and sealed with a rubber septum. The mixture was degassed by nitrogen bubbling at 120 °C until it became homogeneous. Methyl acrylate (0.3 equiv., 0.27 mL, 3 mmol) was added via syringe. The reaction mixture was stirred and irradiated with a 390 nm LED lamp for 6 hours at 120 °C. Upon completion of the reaction, the mixture was precipitated in cold methanol (ca. 40 mL). The resulting solid was collected by filtration and washed via Soxhlet extraction with acetone for 3 h (ca. 4 min per cycle). After the purification, the solid was dried in a vacuum oven (3 mbar) at 80 °C for 12 h. to afford the desired **LDPE–*g*–MA** as a pale-yellow solid (267 mg). The product was characterized by ^{1}H NMR (90 °C in $C_2D_2Cl_4$, 400 MHz) and DSC. Isolated yield = 50%; Graft yield = 8.4%

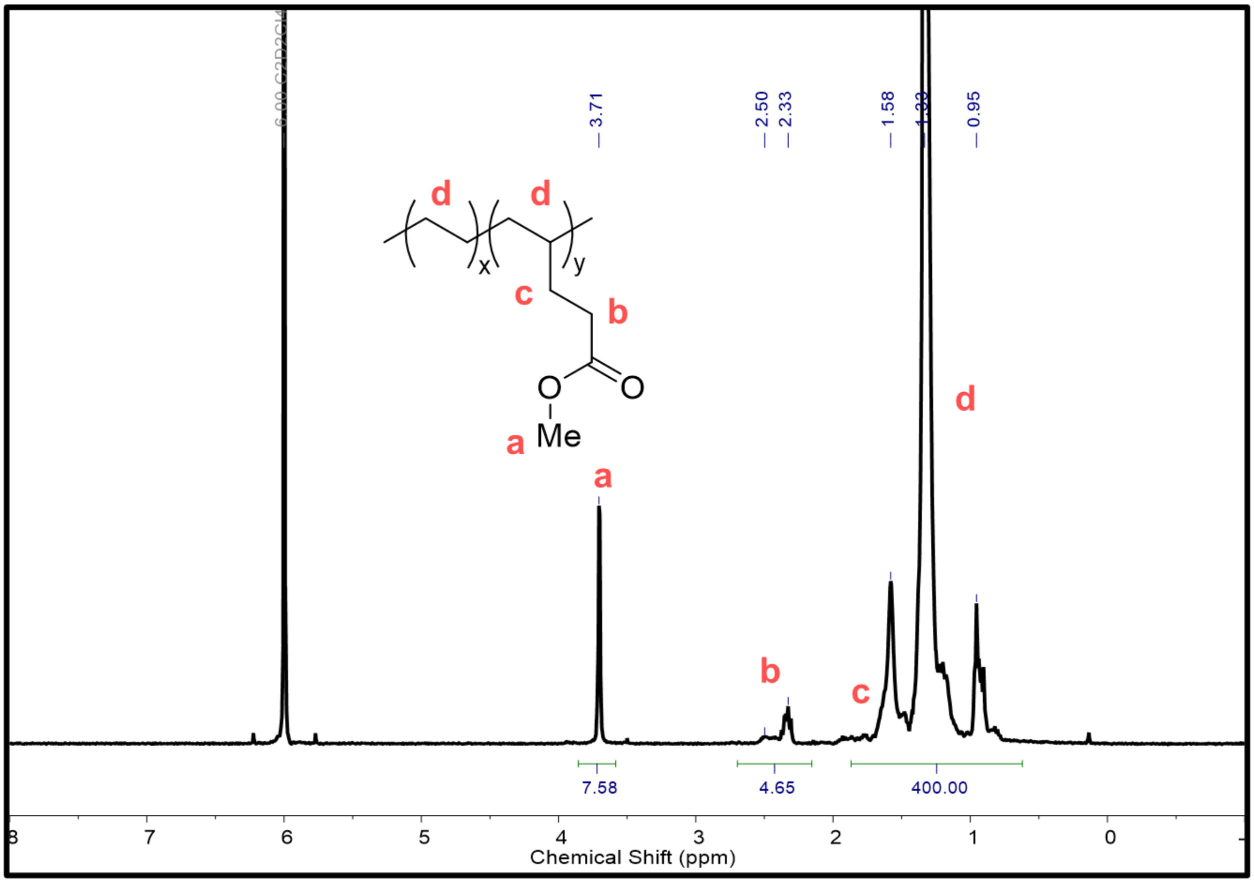

Figure S63. ^{1}H NMR spectrum (400 MHz, $C_2D_2Cl_4$) of LDPE–*g*–$MA_{2.5}$ (T = 90 °C)

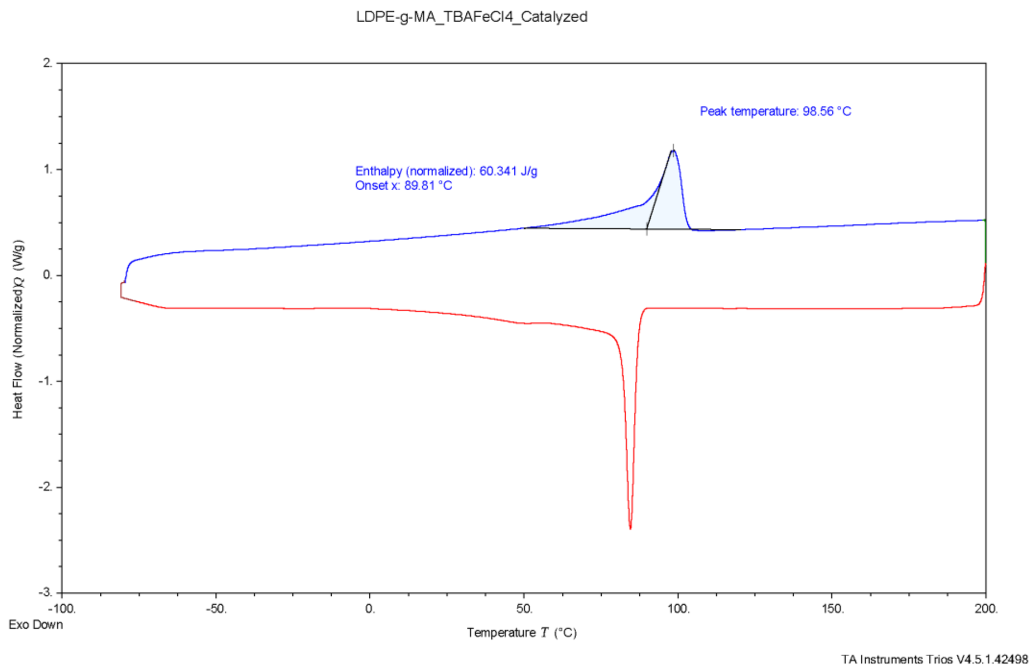

Figure S64. DSC Thermogram of LDPE–*g*–MA (f_{vinyl} = 2.5%). T_m = 98.56 °C, ΔH_m = 60.341 J g^{-1}, X_C = 20.6%.

Synthesis of LDPE–*g*–DEGA (Fig. 2a condition B). A 22 mL borosilicate test tube was charged with LDPE (281 mg, 10 mmol), tetrabutylammonium iron (III) tetrachloride (TBAFeCl$_4$) and 1,2-dichlorobenzene (10 mL), and sealed with a rubber septum. The mixture was degassed by nitrogen bubbling at 120 °C until it became homogeneous. Di(ethylene glycol) ethyl ether acrylate (0.3 equiv., 0.56 mL, 3 mmol) was added via syringe. The reaction mixture was stirred and irradiated with a 390 nm LED lamp for 6 hours at 120 °C. Upon completion of the reaction, the mixture was precipitated in cold methanol (ca. 40 mL). The resulting solid was collected by filtration and washed via Soxhlet extraction with acetone for 3 h (ca. 4 min per cycle). After the purification, the solid was dried in a vacuum oven (3 mbar) at 80 °C for 12 h. to afford the desired **LDPE–*g*–DEGA** as a pale-yellow solid (273 mg). The product was characterized by ^{1}H NMR (90 °C in $C_2D_2Cl_4$, 400 MHz) and DSC. Isolated yield = 32%; Graft yield = 6.5%

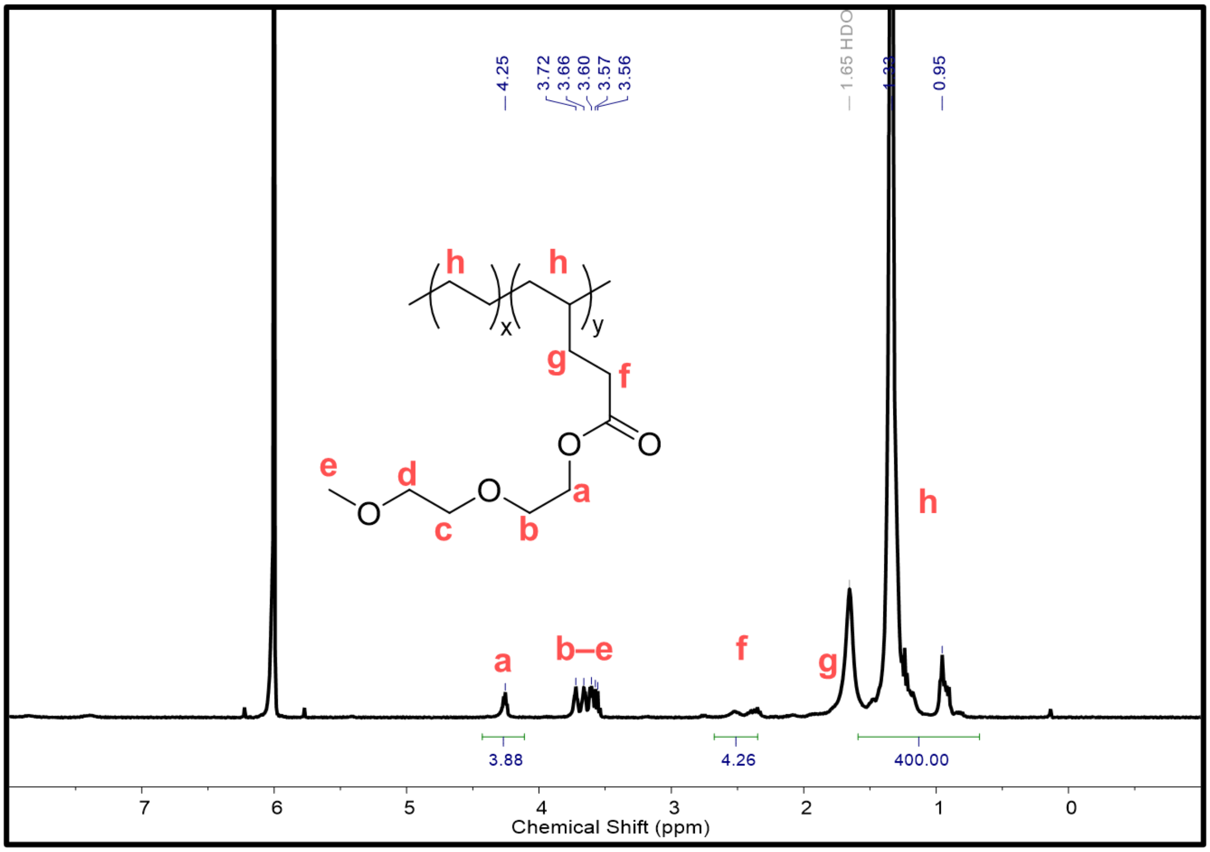

Figure S65. ^{1}H NMR spectrum (400 MHz, $C_2D_2Cl_4$) of LDPE–*g*–DEGA$_{1.9}$ (T = 90 °C)

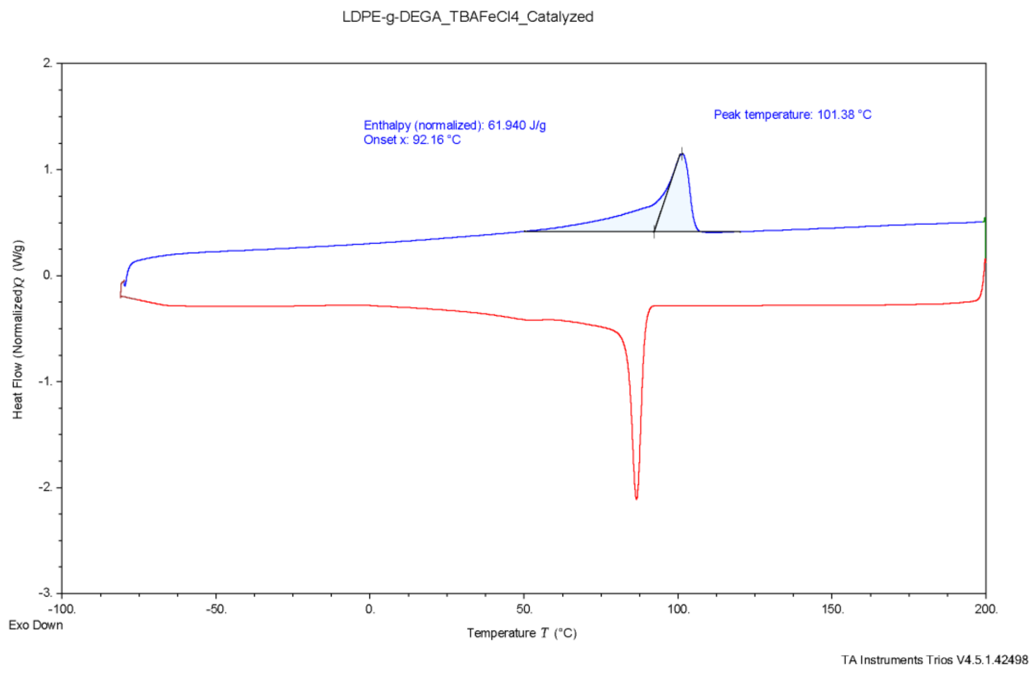

Figure S66. DSC Thermogram of LDPE–*g*–DEGA (f_{vinyl} = 2.5%). T_m = 101.38 °C, ΔH_m = 61.940 J g^{-1}, X_C = 21.1%.

Synthesis of LDPE–*g*–PMA (Figure 5). A 22 mL borosilicate test tube was charged with LDPE (281 mg, 10 mmol) and 1,2-dichlorobenzene (10 mL), and sealed with a rubber septum. The mixture was degassed by nitrogen bubbling at 90 °C until it became homogeneous. Methyl acrylate (0.3 equiv., 0.27 mL, 3 mmol) was added via syringe. The reaction mixture was stirred and irradiated with a 390 nm LED lamp for 6 hours at 90 °C. Upon completion of the reaction, the mixture was precipitated in cold methanol (ca. 40 mL). The resulting solid was collected by filtration and washed via Soxhlet extraction with acetone for 6 h (ca. 4 min per cycle). After the purification, the solid was dried in a vacuum oven (3 mbar) at 80 °C for 12 h. to afford the desired **LDPE–*g*–PMA** as a white solid (275 mg). The product was characterized by ^{1}H NMR (90 °C in $C_2D_2Cl_4$, 400 MHz), 2D DOSY NMR, DSC, and high temperature size exclusion chromatography. Isolated yield = 51%; Graft yield = 13%

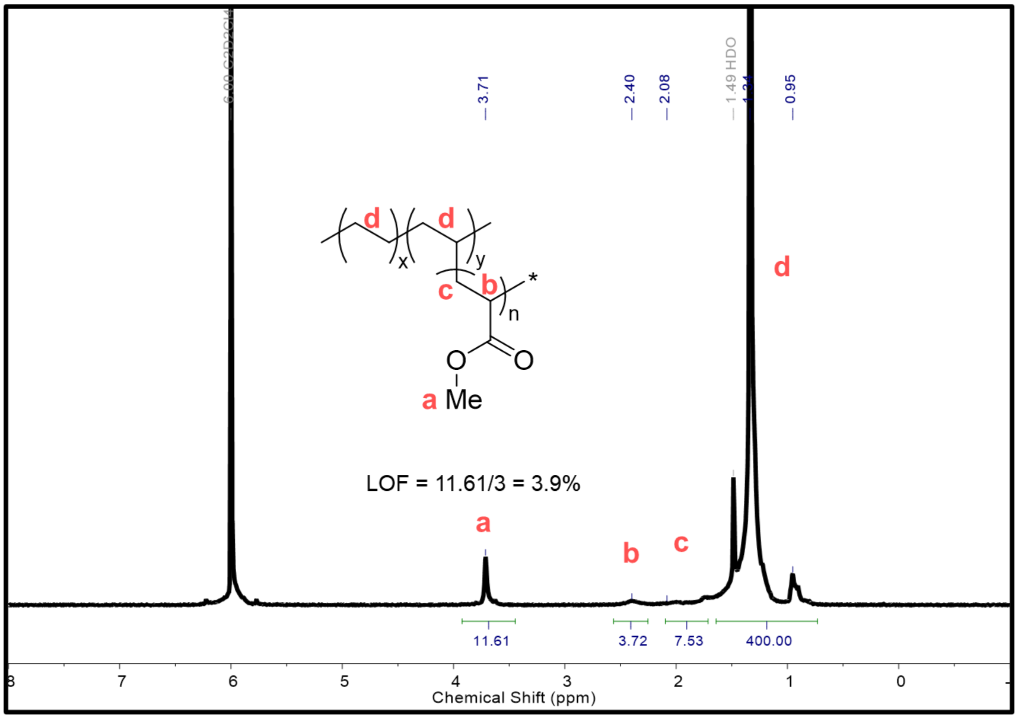

Figure S67. ^{1}H NMR spectrum (400 MHz, $C_2D_2Cl_4$) of LDPE–*g*–$PMA_{3.9}$ (*T* = 90 °C)

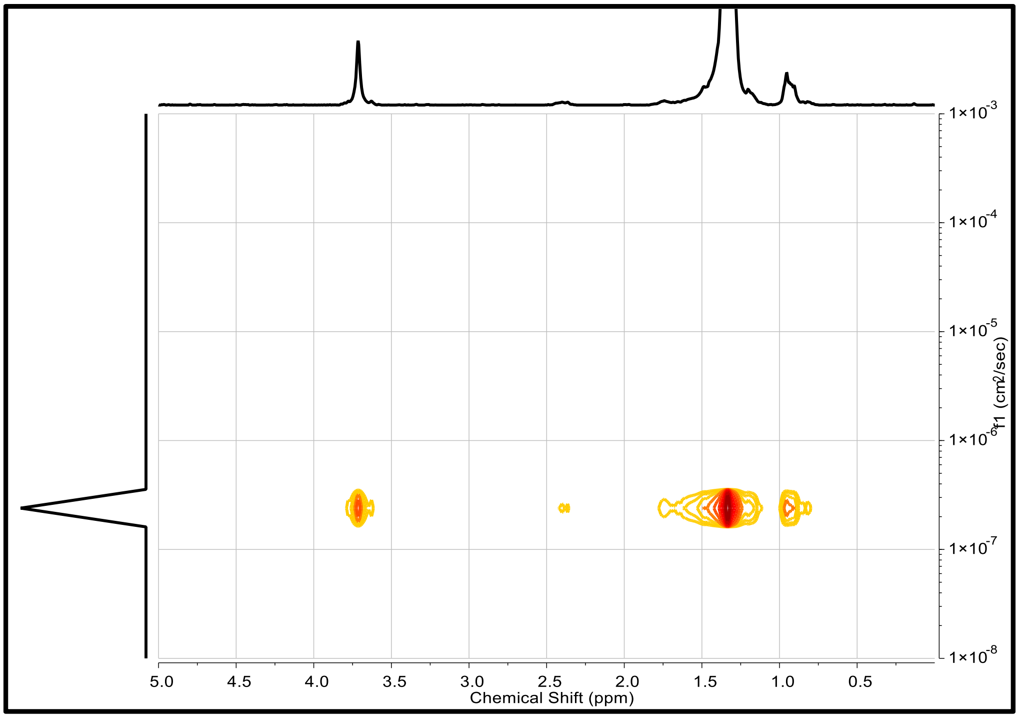

Figure S68. 2D DOSY NMR spectrum (400 MHz, $C_2D_2Cl_4$) of LDPE–*g*–PMA$_{3.9}$ (T = 90 °C)

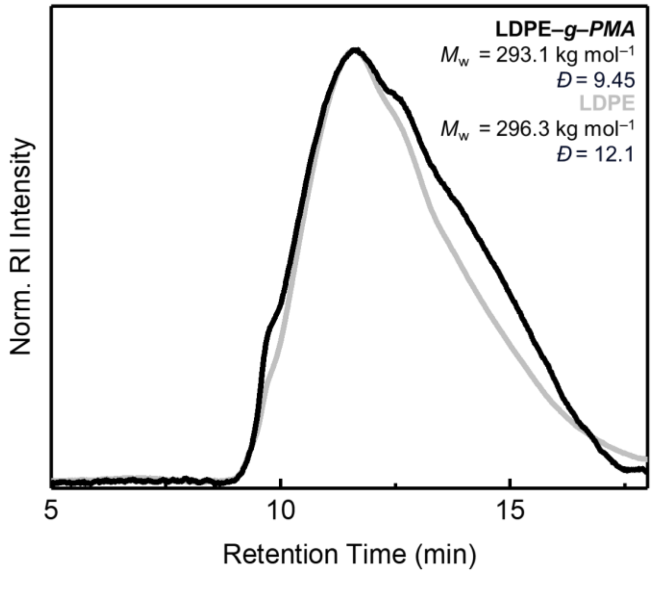

Figure S69. HT SEC trace of **LDPE–*g*–PMA$_{3.9}$** (1,2,4-TCB, 1.0 mL min^{-1} at 150 °C)

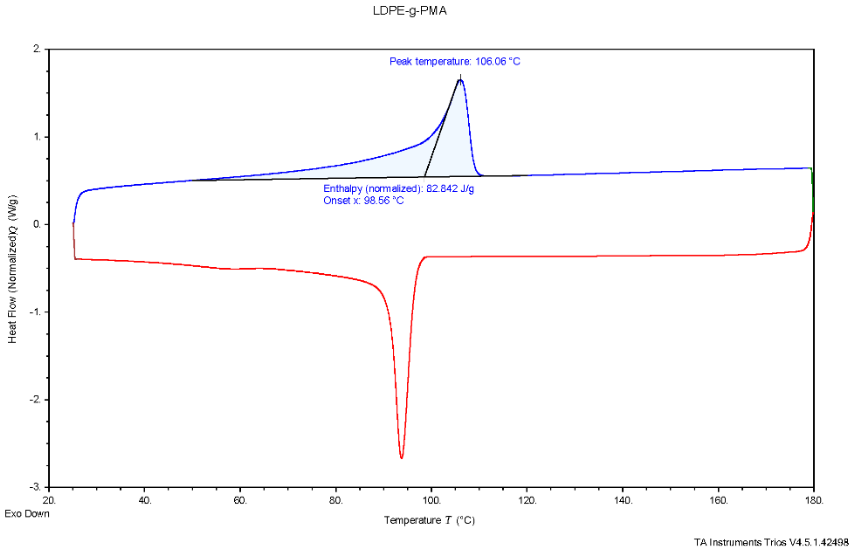

Figure S70. DSC Thermogram of LDPE–*g*–PMA (f_{vinyl} = 3.9%). T_m = 106.06 °C, ΔH_m = 82.842 J g^{-1}, X_C = 28.3%.

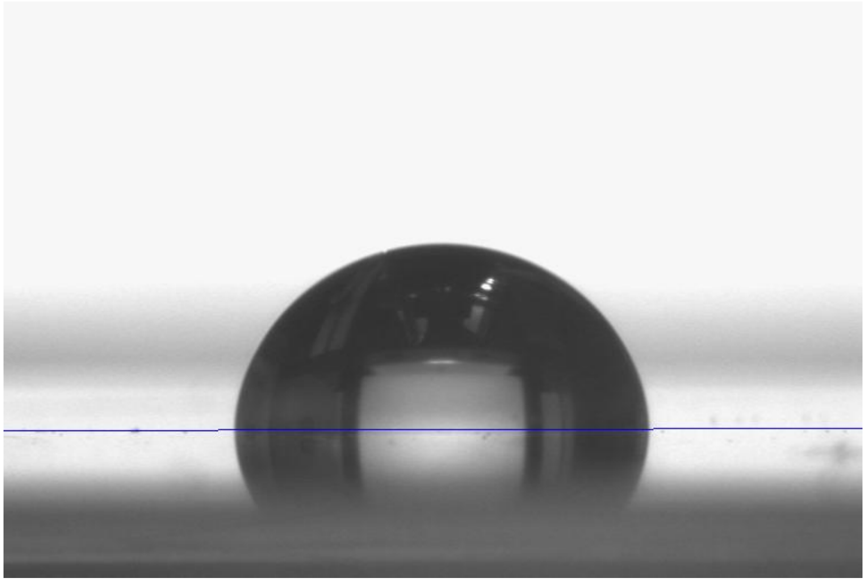

Figure S71. Water contact angle measured for LDPE–*g*–PMA (f_{vinyl} = 3.9%). WCA = 90.4°

Synthesis of LDPE–*g*–PDEGA (Figure 5). A 22 mL borosilicate test tube was charged with LDPE (281 mg, 10 mmol) and 1,2-dichlorobenzene (10 mL), and sealed with a rubber septum. The mixture was degassed by nitrogen bubbling at 90 °C until it became homogeneous. Di(ethylene glycol) ethyl ether acrylate (0.3 equiv., 0.56 mL, 3 mmol) was added via syringe. The reaction mixture was stirred and irradiated with a 390 nm LED lamp for 6 hours at 90 °C. Upon completion of the reaction, the mixture was precipitated in cold methanol (ca. 40 mL). The resulting solid was collected by filtration and washed via Soxhlet extraction with acetone for 3 h (ca. 4 min per cycle). After the purification, the solid was dried in a vacuum oven (3 mbar) at 80 °C for 12 h. to afford the desired **LDPE–*g*–PDEGA** as a white solid (271 mg). The product was characterized by ^{1}H NMR (90 °C in $C_2D_2Cl_4$, 400 MHz), 2D DOSY NMR, DSC, and high temperature size exclusion chromatography. Isolated yield = 32%; Graft yield = 5.1%

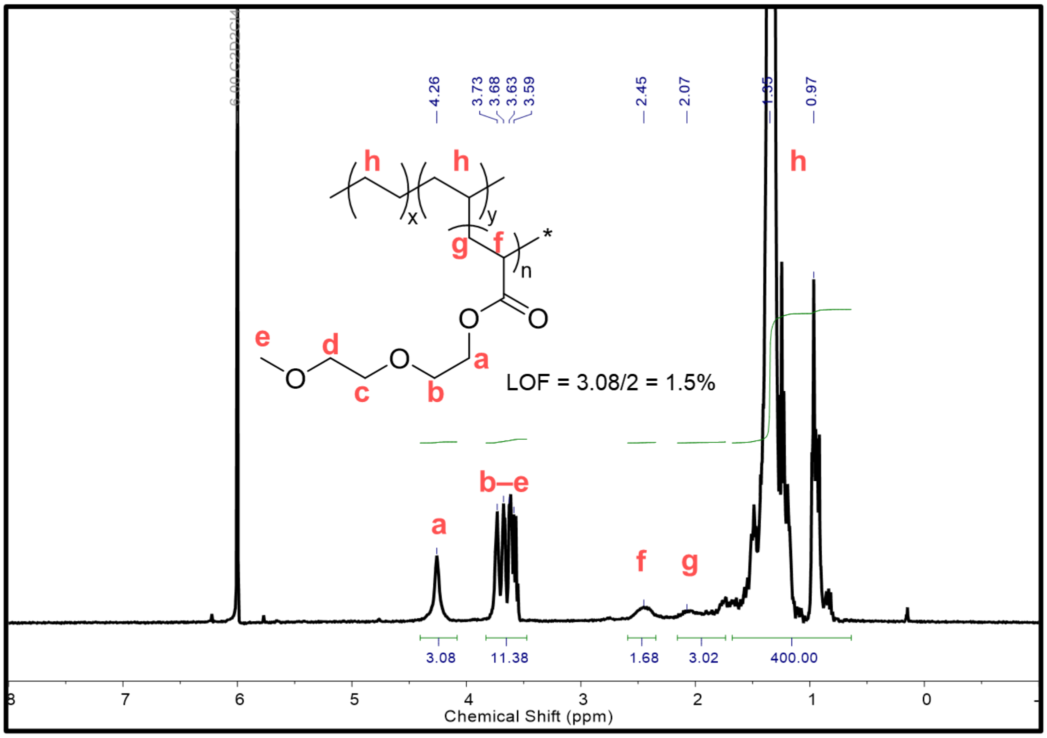

Figure S72. ^{1}H NMR spectrum (400 MHz, $C_2D_2Cl_4$) of LDPE–*g*–PDEGA$_{1.5}$ (T = 90 °C)

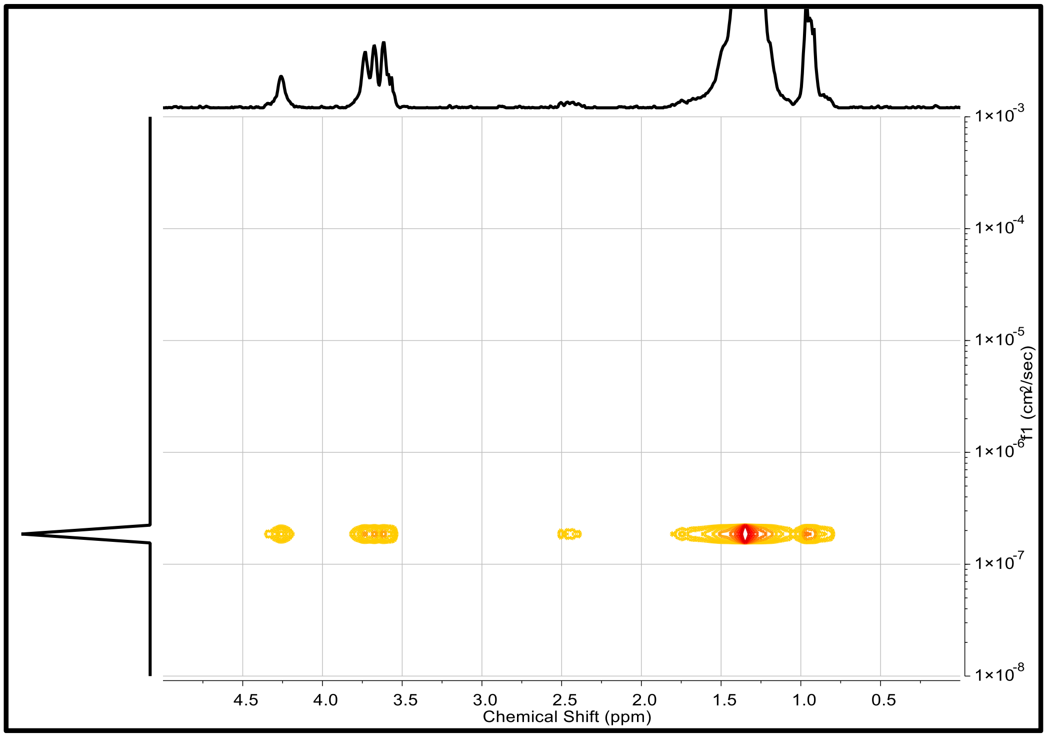

Figure S73. 2D DOSY NMR spectrum (400 MHz, $C_2D_2Cl_4$) of LDPE–*g*–PDEGA$_{1.5}$ (T = 90 °C)

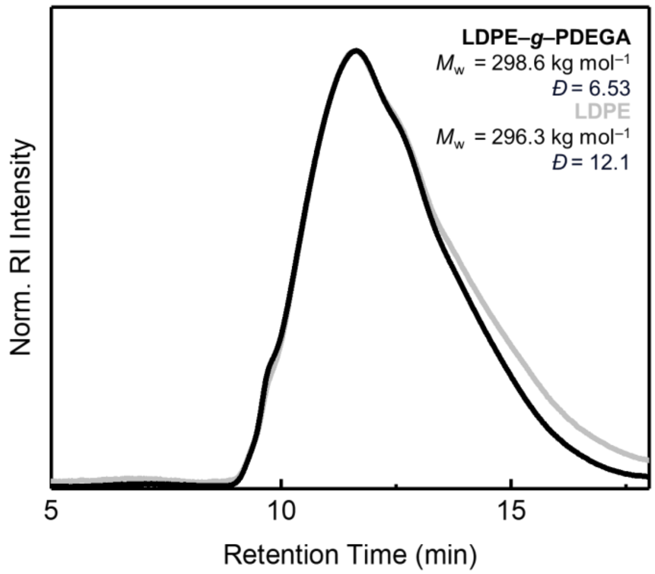

Figure S74. HT SEC trace of LDPE–*g*–PDEGA$_{1.5}$ (1,2,4-TCB, 1.0 mL min^{-1} at 150 °C)

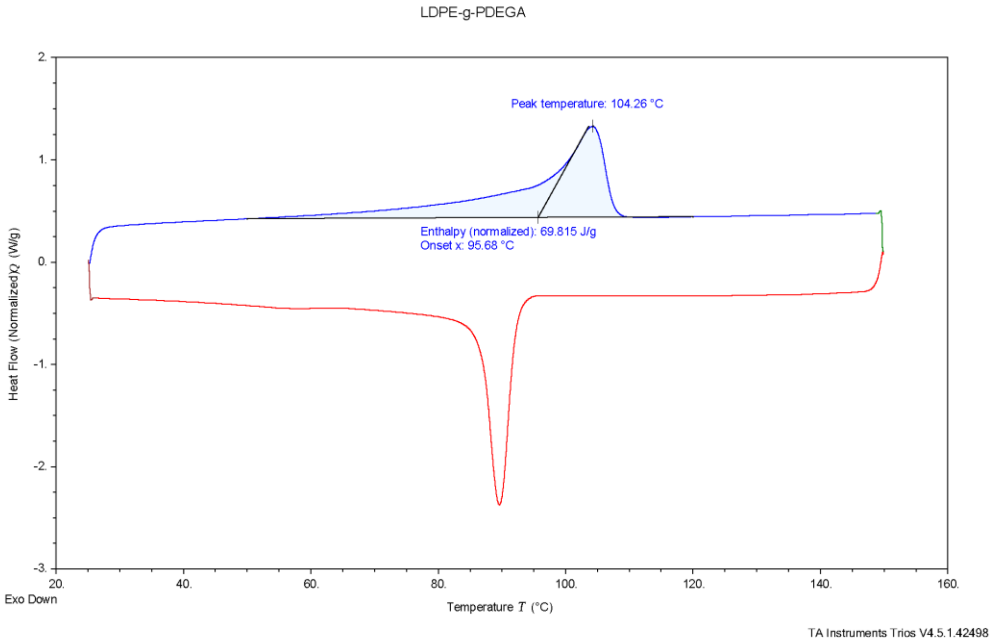

Figure S75. DSC Thermogram of LDPE–*g*–PDEGA (f_{vinyl} = 1.5%). T_m = 104.26 °C, ΔH_m = 69.815 J g^{-1}, X_C = 23.8%.

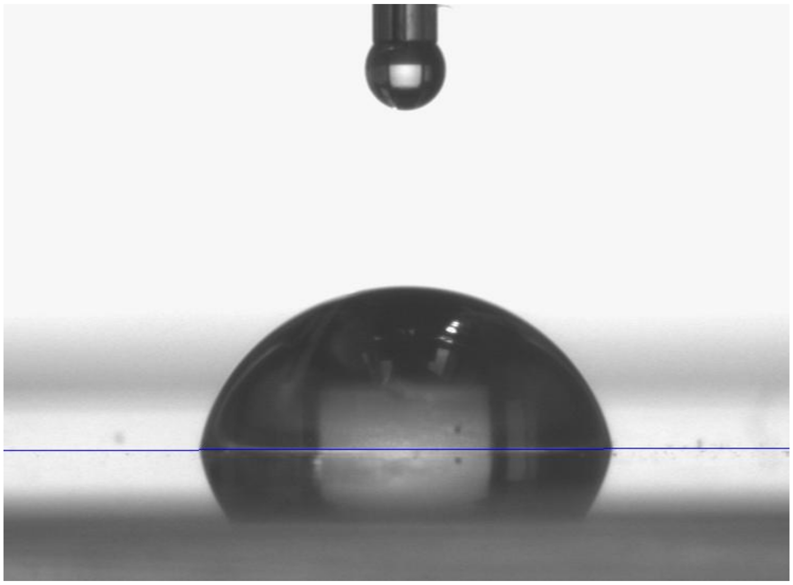

Figure S76. Water contact angle measured for LDPE–*g*–PDEGA (f_{vinyl} = 1.5%). WCA = 80.8°

Synthesis of LDPE–*g*–PMMA (Figure 5). A 22 mL borosilicate test tube was charged with LDPE (281 mg, 10 mmol) and 1,2-dichlorobenzene (10 mL), and sealed with a rubber septum. The mixture wasdegassed by nitrogen bubbling at 90 °C until it became homogeneous. Methyl methacrylate (0.2 equiv., 0.213 mL, 2 mmol) was added via syringe. The reaction mixture was stirred and irradiated with a 390 nm LED lamp for 12 hours at 90 °C. Upon completion of the reaction, the mixture was precipitated in cold methanol (ca. 40 mL). The resulting solid was collected by filtration and washed via Soxhlet extraction with acetone for 3 h (ca. 4 min per cycle). After the purification, the solid was dried in a vacuum oven (3 mbar) at 80 °C for 12 h. to afford the desired **LDPE–*g*–PMMA** as a white solid (281 mg). The product was characterized by ^{1}H NMR (90 °C in $C_2D_2Cl_4$, 400 MHz), 2D DOSY NMR, DSC, and high temperature size exclusion chromatography. Isolated yield = 58%; Graft yield = 28%

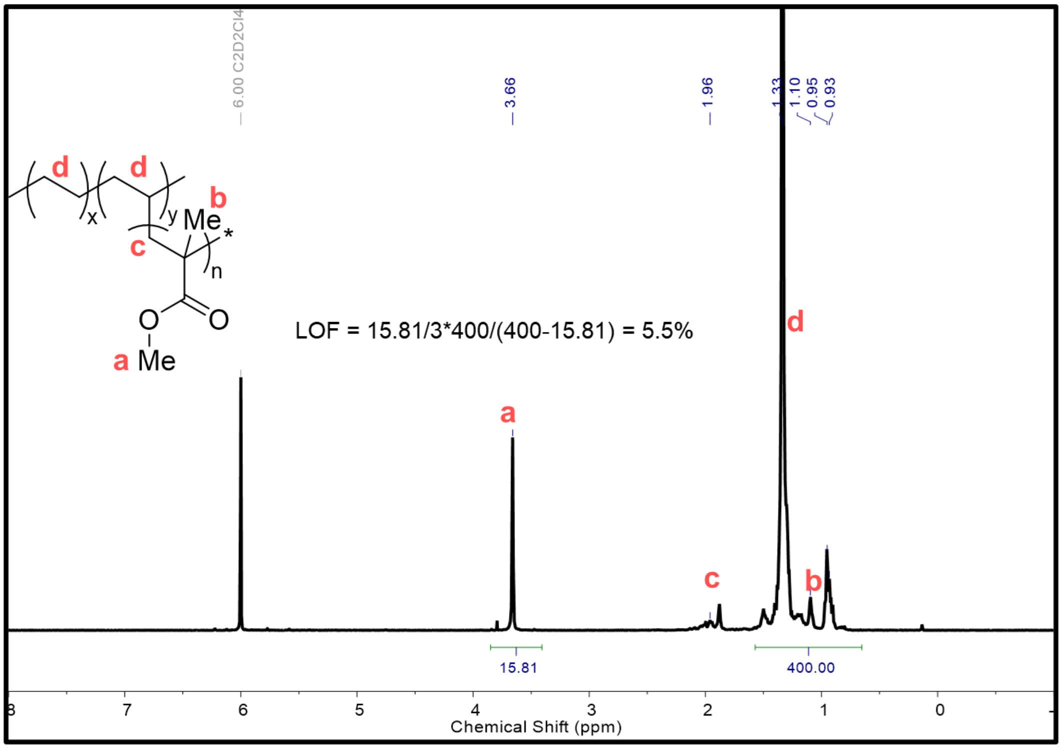

Figure S77. ^{1}H NMR spectrum (400 MHz, $C_2D_2Cl_4$) of LDPE–*g*–PMMA$_{5.5}$ (T = 90 °C)

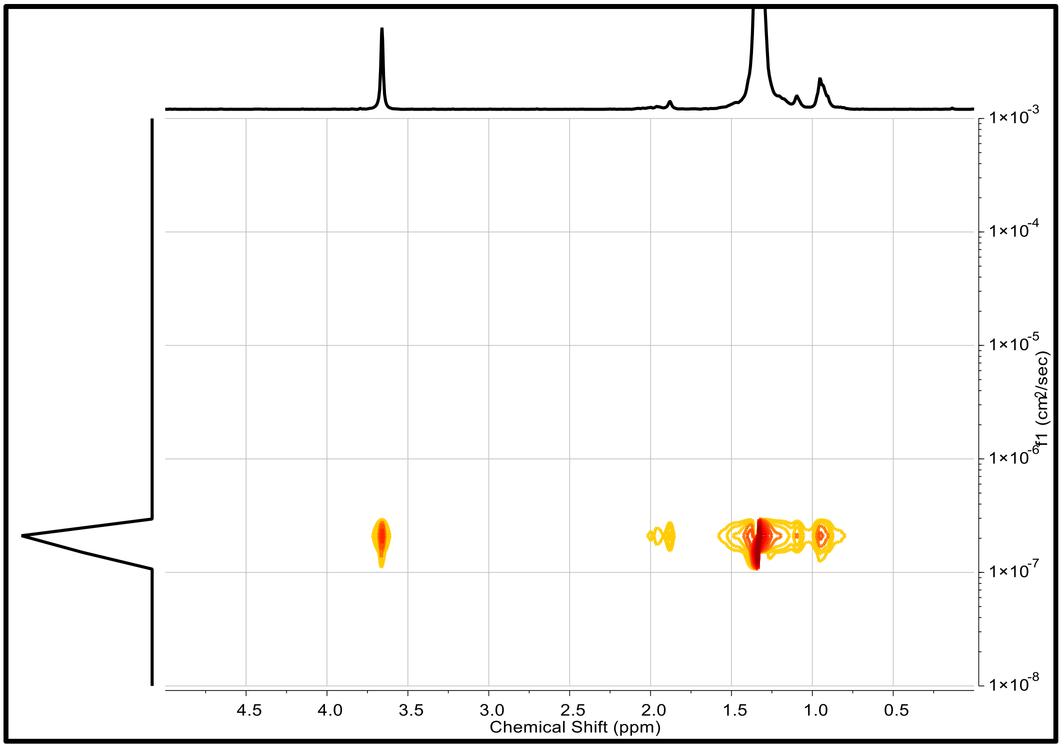

Figure S78. 2D DOSY NMR spectrum (400 MHz, $C_2D_2Cl_4$) of LDPE–*g*–$PMMA_{5.3}$ (T = 90 °C)

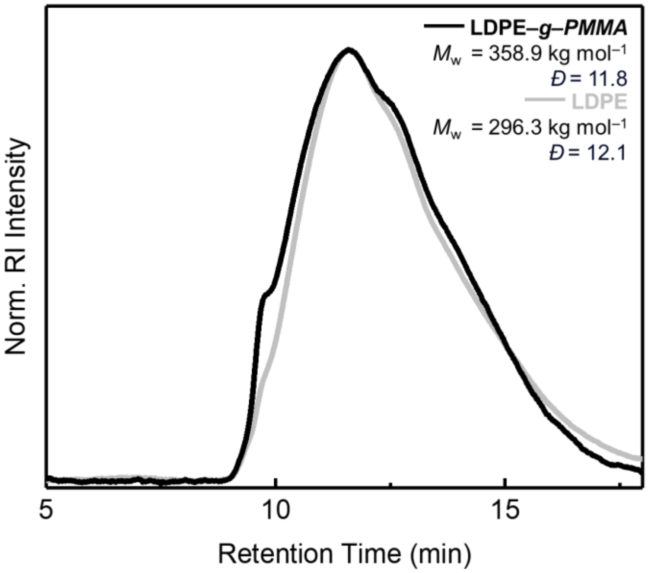

Figure S79. HT SEC trace of LDPE–*g*–$PMMA_{5.5}$ (1,2,4-TCB, 1.0 mL min^{-1} at 150 °C)

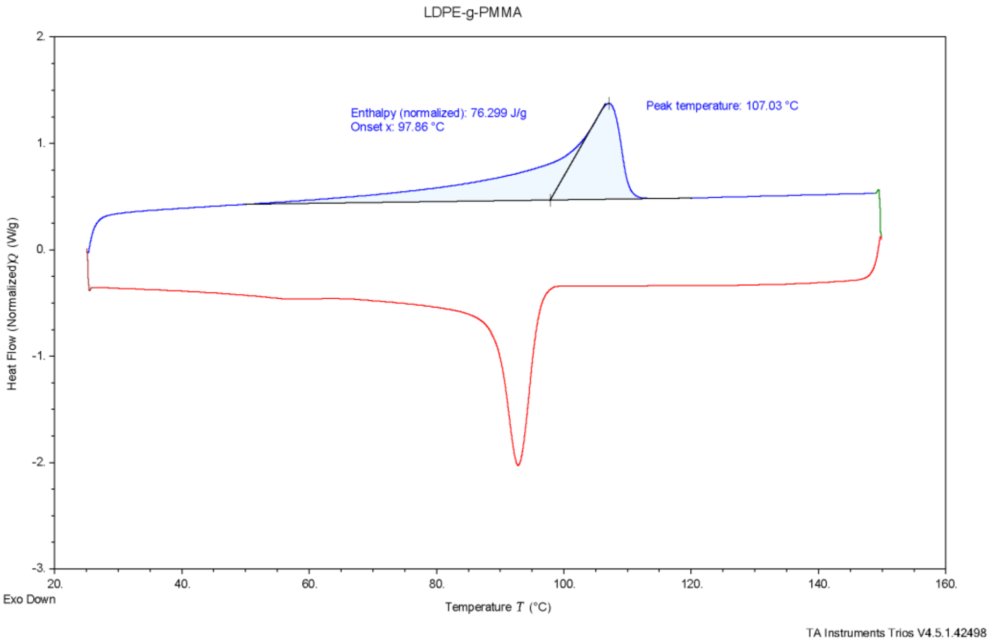

Figure S80. DSC Thermogram of LDPE–*g*–PMMA (f_{vinyl} = 5.3%). T_m = 107.0 °C, ΔH_m = 76.299 J g^{-1}, X_C = 26.0%.

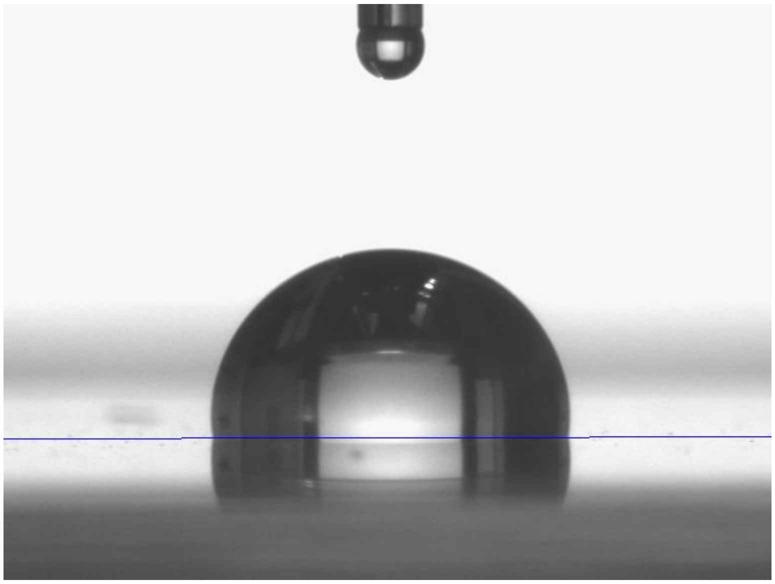

Figure S81. Water contact angle measured for LDPE–*g*–PMMA (f_{vinyl} = 5.5%). WCA = 97.4°

Synthesis of LDPE–*g*–PNIPAM (Figure 5). A 22 mL borosilicate test tube was charged with LDPE (281 mg, 10 mmol) and 1,2-dichlorobenzene (8 mL), and sealed with a rubber septum. The mixture was degassed by nitrogen bubbling at 120 °C until it became homogeneous. *N*-isopropyl acrylamide (0.2 equiv., 226 mg, 2 mmol) in 2 mL of 1,2-dichlorobenzene was added via syringe. The reaction mixture was stirred and irradiated with a 390 nm LED lamp for 6 hours at 120 °C. Upon completion of the reaction, the mixture was precipitated in cold methanol (ca. 40 mL). The resulting solid was collected by filtration and washed via Soxhlet extraction with acetone for 6 h (ca. 4 min per cycle). After the purification, the solid was dried in a vacuum oven (3 mbar) at 80 °C for 12 h. to afford the desired **LDPE–*g*–PNIPAM** as a white solid (273 mg). The product was characterized by ^{1}H NMR (90 °C in $C_2D_2Cl_4$, 400 MHz), 2D DOSY NMR, DSC, and high temperature size exclusion chromatography. Isolated yield = 55%; Graft yield = 34%

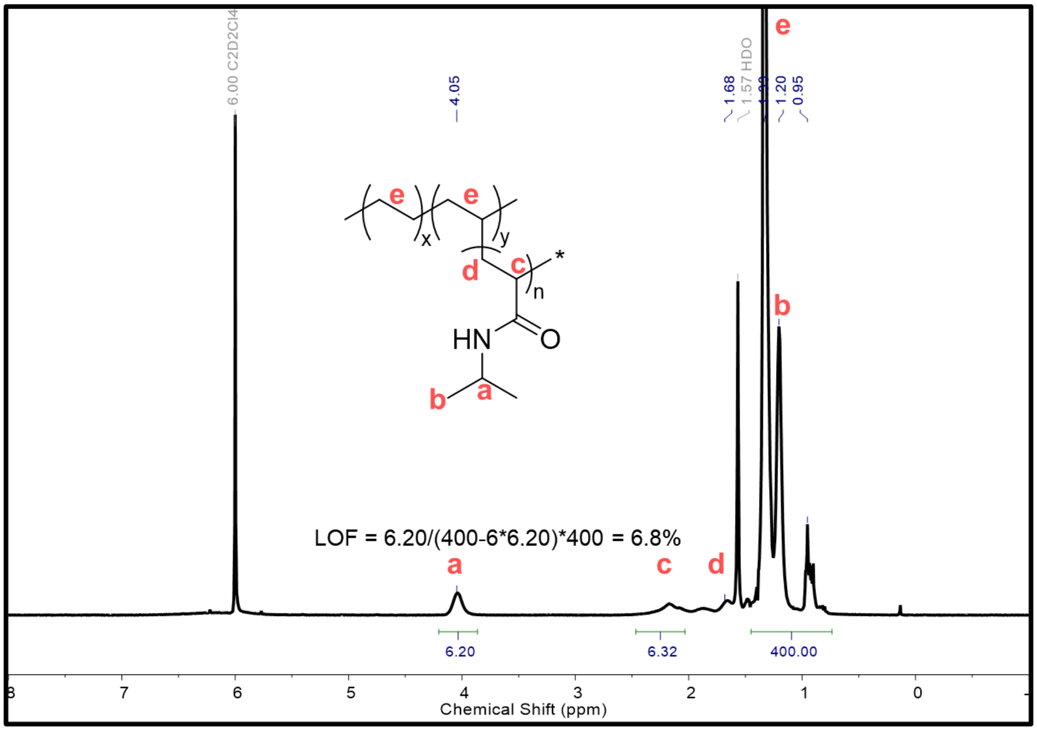

Figure S82. ^{1}H NMR spectrum (400 MHz, $C_2D_2Cl_4$) of LDPE–*g*–PNIPAM$_{6.8}$ (T = 90 °C)

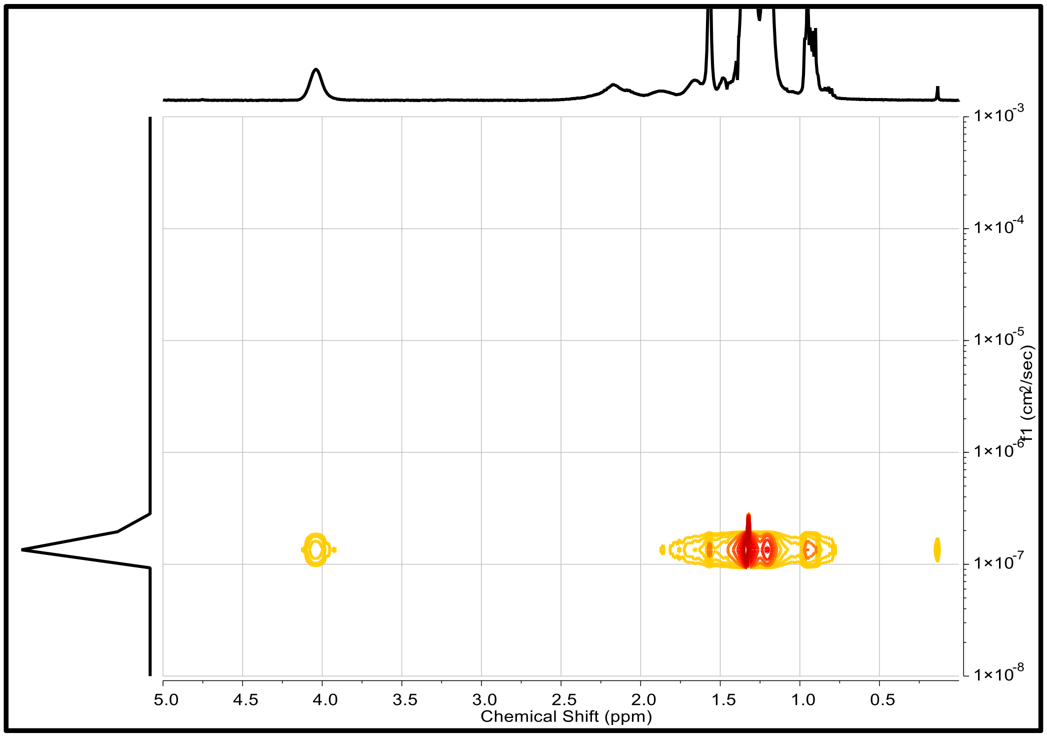

Figure S83. 2D DOSY NMR spectrum (400 MHz, $C_2D_2Cl_4$) of LDPE–*g*–PNIPAM$_{6.8}$ (T = 90 °C)

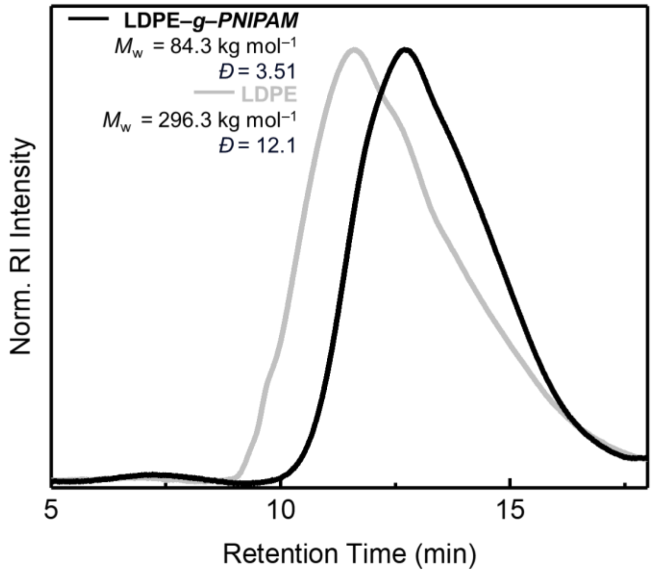

Figure S84. HT SEC trace of LDPE–*g*–PNIPAM$_{6.8}$ (1,2,4-TCB, 1.0 mL min^{-1} at 150 °C)

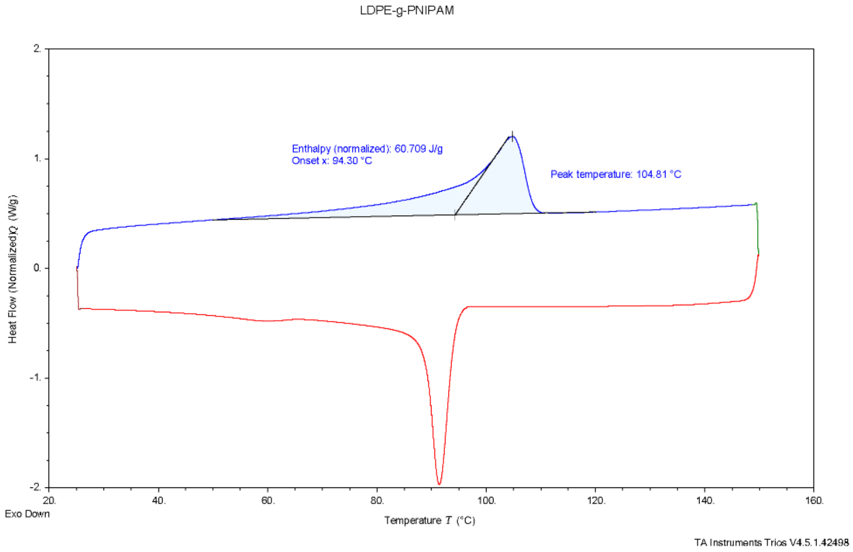

Figure S85. DSC Thermogram of LDPE–*g*–PNIPAM (f_{vinyl} = 6.8%). T_m = 104.8 °C, ΔH_m = 60.709 J g^{-1}, X_C = 20.7%.

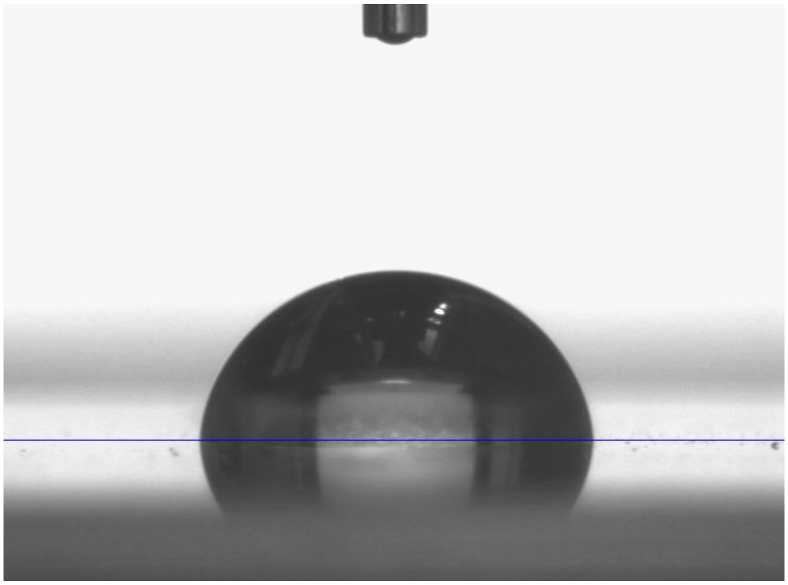

Figure S86. Water contact angle measured for LDPE–*g*–PNIPAM (f_{vinyl} = 6.8%). WCA = 87.3°

Synthesis of LDPE–*g*–PNNPAM (Figure 5). A 22 mL borosilicate test tube was charged with LDPE (281 mg, 10 mmol) and 1,2-dichlorobenzene (10 mL), and sealed with a rubber septum. The mixture was degassed by nitrogen bubbling at 120 °C until it became homogeneous. *N*-n-propyl acrylamide (0.2 equiv., 0.23 mL, 2 mmol) was added via syringe. The reaction mixture was stirred and irradiated with a 390 nm LED lamp for 4 hours at 120 °C. Upon completion of the reaction, the mixture was precipitated in cold methanol (ca. 40 mL). The resulting solid was collected by filtration and washed via Soxhlet extraction with acetone for 3 h (ca. 4 min per cycle). After the purification, the solid was dried in a vacuum oven (3 mbar) at 80 °C for 12 h. to afford the desired **LDPE–*g*–PMA** as a white solid (275 mg). The product was characterized by ^{1}H NMR (90 °C in $C_2D_2Cl_4$, 400 MHz), 2D DOSY NMR, DSC, and high temperature size exclusion chromatography. Isolated yield = 54%; Graft yield = 22%

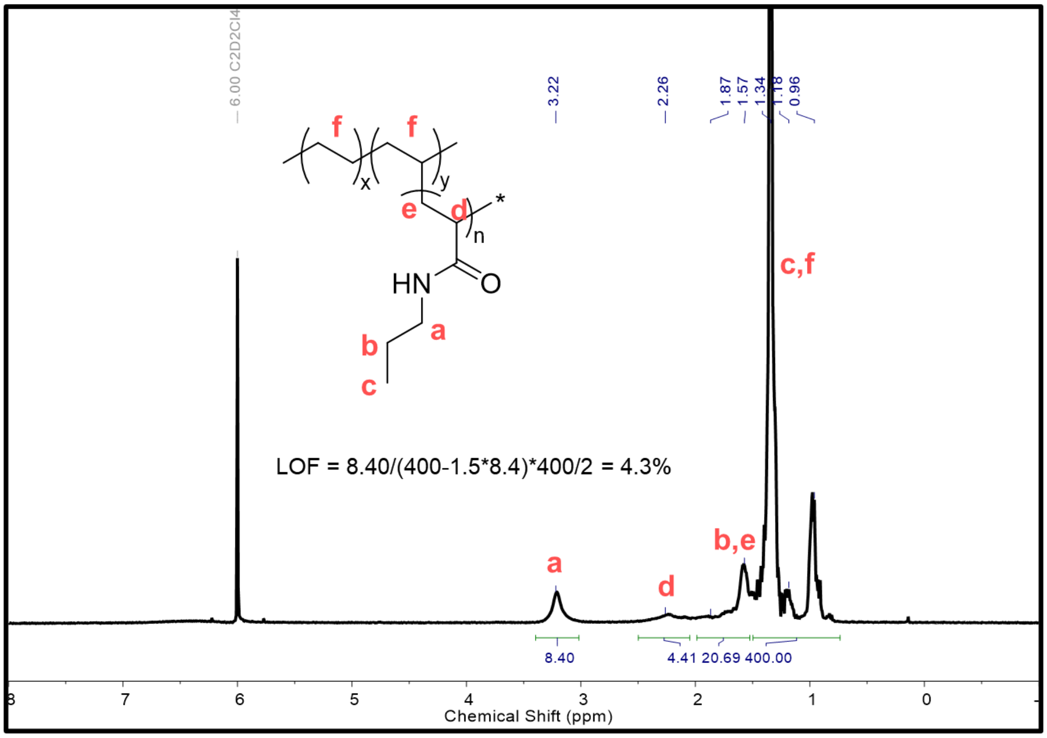

Figure S87. ^{1}H NMR spectrum (400 MHz, $C_2D_2Cl_4$) of LDPE–*g*–PNNPAM$_{4.3}$ (T = 90 °C)

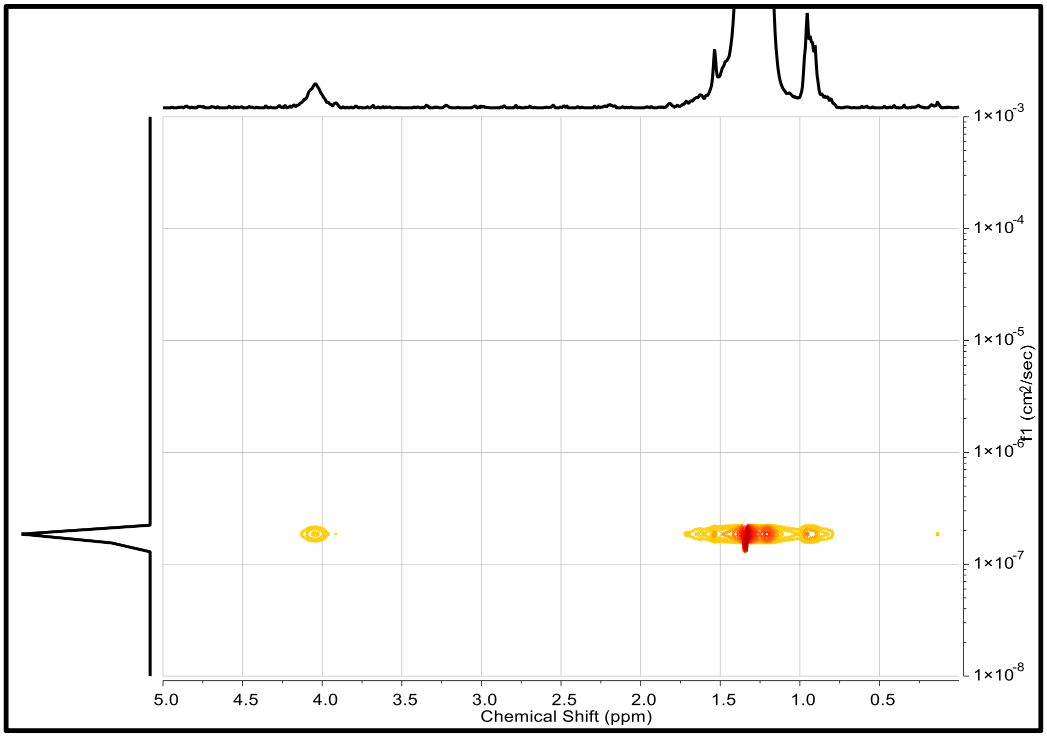

Figure S88. 2D DOSY NMR spectrum (400 MHz, $C_2D_2Cl_4$) of LDPE–*g*–PNNPAM$_{4.3}$ (T = 90 °C)

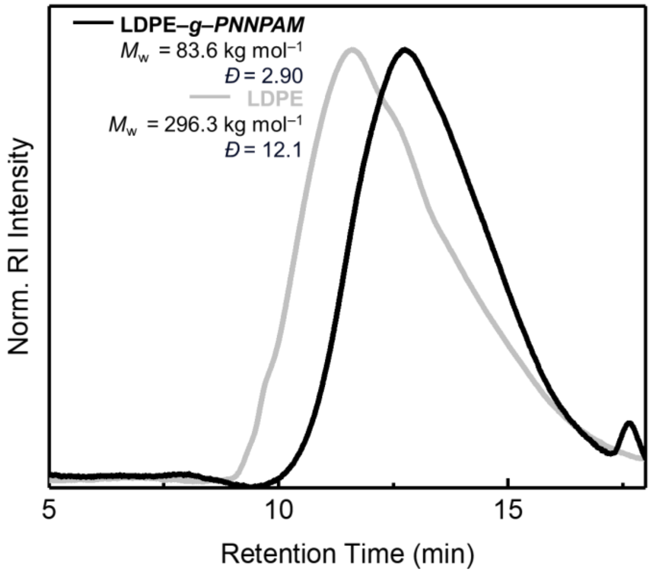

Figure S89. HT SEC trace of LDPE–*g*–PNNPAM$_{4.3}$ (1,2,4-TCB, 1.0 mL min^{-1} at 150 °C)

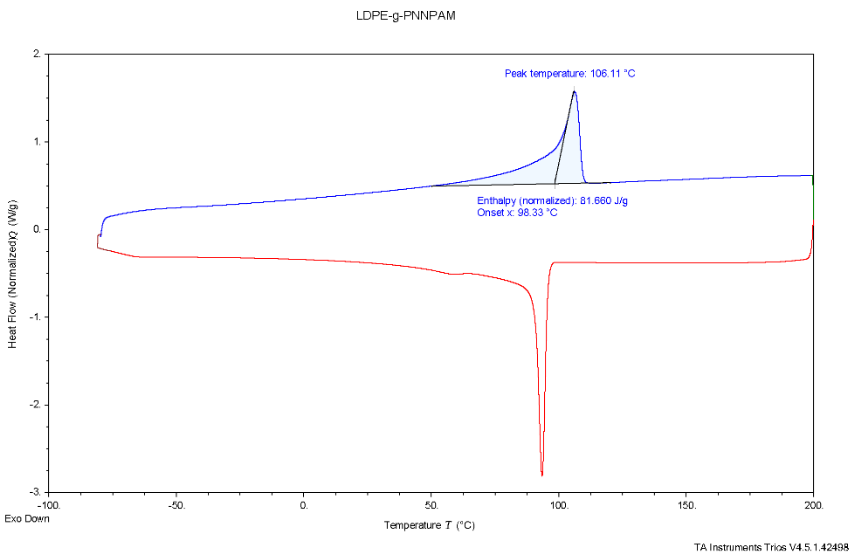

Figure S90. DSC Thermogram of LDPE–*g*–PNNPAM (f_{vinyl} = 4.3%). T_m = 106.1 °C, ΔH_m = 81.66 J g^{-1}, X_C = 27.9%.

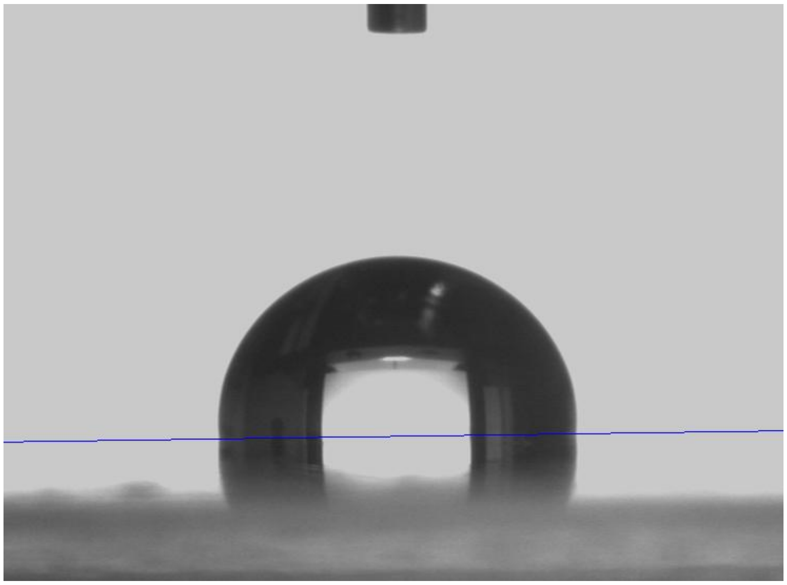

Figure S91. Water contact angle measured for LDPE–*g*–PNNPAM (f_{vinyl} = 4.3%). WCA = 96.3°

Synthesis of LDPE–*g*–PNEtAM (Figure 5). A 22 mL borosilicate test tube was charged with LDPE (281 mg, 10 mmol) and 1,2-dichlorobenzene (10 mL), and sealed with a rubber septum. The mixture was degassed by nitrogen bubbling at 120 °C until it became homogeneous. Methyl acrylate (0.2 equiv., 0.20 mL, 2 mmol) was added via syringe. The reaction mixture was stirred and irradiated with a 390 nm LED lamp for 6 hours at 120 °C. Upon completion of the reaction, the mixture was precipitated in cold methanol (ca. 40 mL). The resulting solid was collected by filtration and washed via Soxhlet extraction with isopropanol for 12 h (ca. 6 min per cycle). After the purification, the solid was dried in a vacuum oven (3 mbar) at 80 °C for 12 h. to afford the desired **LDPE–*g*–PNEtAM** as a white solid (298 mg). The product was characterized by ^{1}H NMR (90 °C in $C_2D_2Cl_4$, 400 MHz), 2D DOSY NMR, DSC, and high temperature size exclusion chromatography. Isolated yield = 62%; Graft yield = 32%

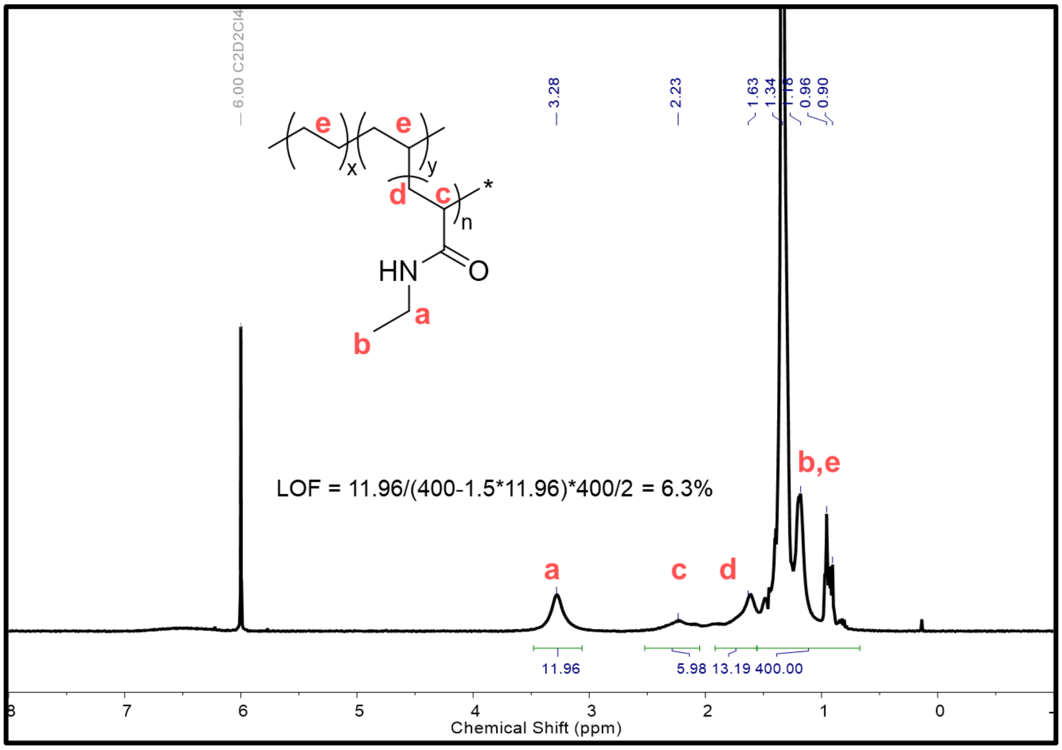

Figure S92. ^{1}H NMR spectrum (400 MHz, $C_2D_2Cl_4$) of LDPE–*g*–PNEtAM$_{6.3}$ (T = 90 °C)

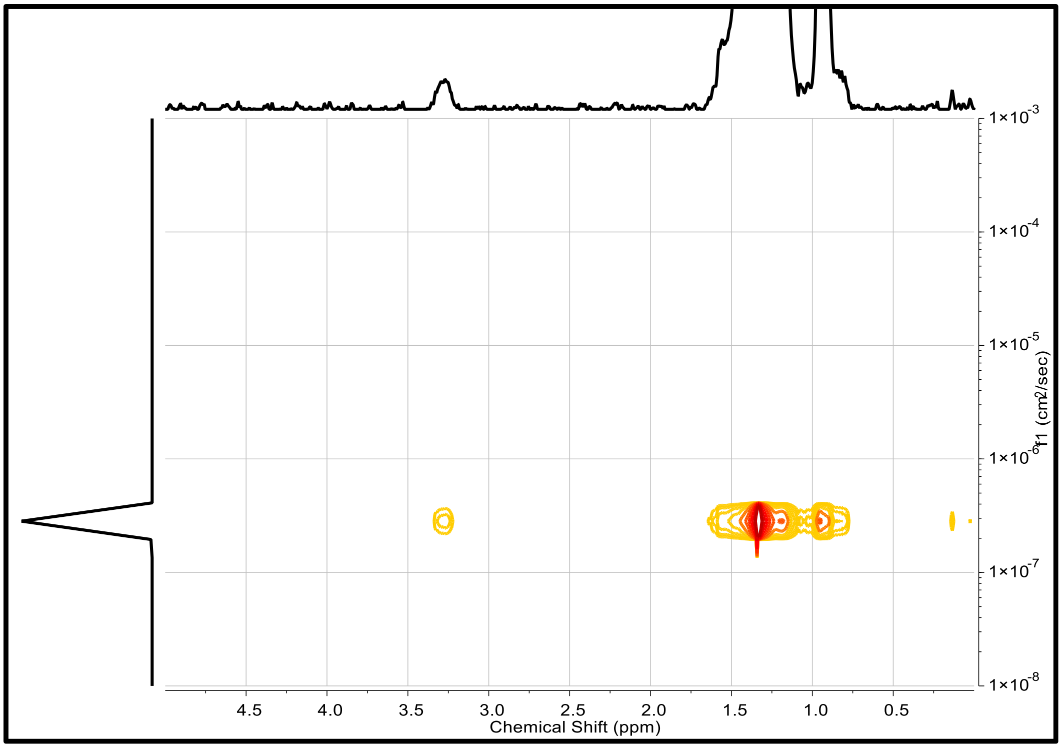

Figure S93. 2D DOSY NMR spectrum (400 MHz, $C_2D_2Cl_4$) of LDPE–*g*–PNEtAM$_{6.3}$ (T = 90 °C)

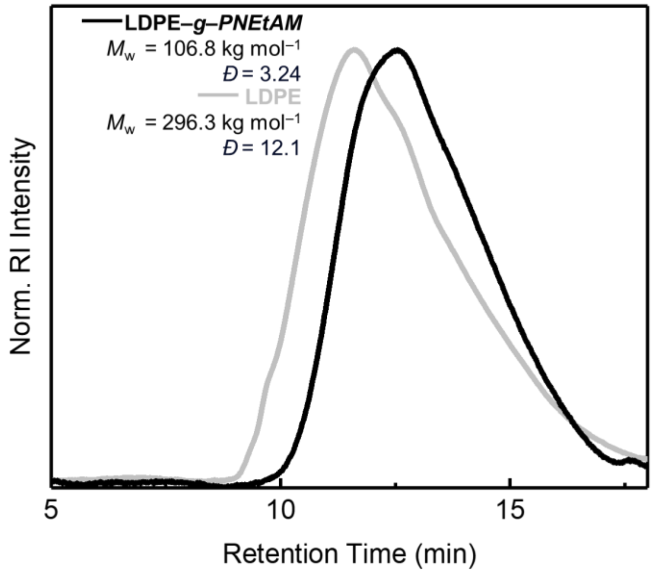

Figure S94. HT SEC trace of LDPE–*g*–PNEtAM$_{6.3}$ (1,2,4-TCB, 1.0 mL min^{-1} at 150 °C)

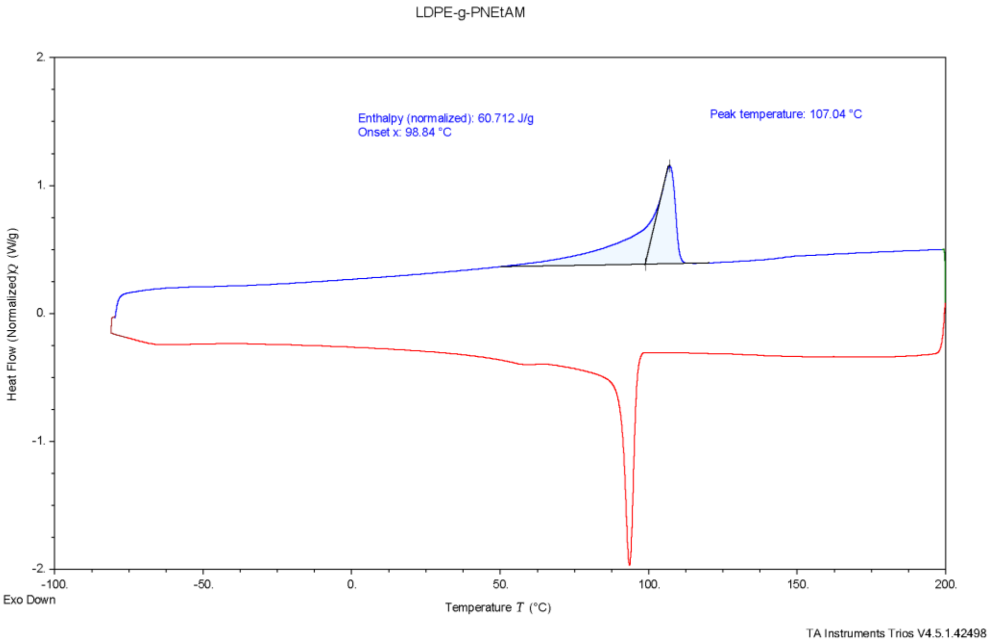

Figure S95. DSC Thermogram of LDPE–*g*–PNEtPAM (f_{vinyl} = 6.3%). T_m = 107.04 °C, ΔH_m = 60.712 J g^{-1}, X_C = 20.7%.

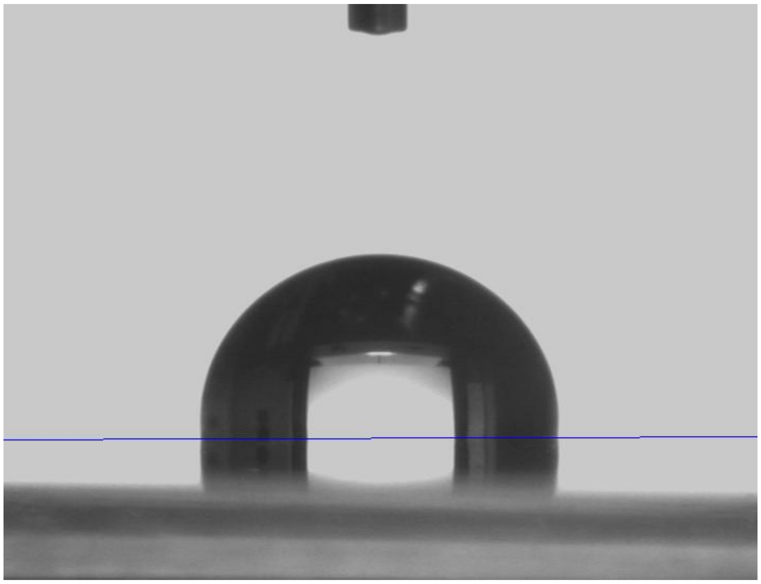

Figure S96. Water contact angle measured for LDPE–*g*–PNEtAM (f_{vinyl} = 4.3%). WCA = 95.5°

Synthesis of LDPE–*g*–PNDMAM (Figure 5). A 22 mL borosilicate test tube was charged with LDPE (281 mg, 10 mmol) and 1,2-dichlorobenzene (10 mL), and sealed with a rubber septum. The mixture was degassed by nitrogen bubbling at 120 °C until it became homogeneous. *N*,*N*-dimethyle acrylamide (0.2 equiv., 0.20 mL, 2 mmol) was added via syringe. The reaction mixture was stirred and irradiated with a 390 nm LED lamp for 6 hours at 120 °C. Upon completion of the reaction, the mixture was precipitated in cold methanol (ca. 40 mL). The resulting solid was collected by filtration and washed via Soxhlet extraction with isopropanol for 8 h (ca. 6 min per cycle). After the purification, the solid was dried in a vacuum oven (3 mbar) at 80 °C for 12 h. to afford the desired **LDPE–*g*–PNDMAM** as a white solid (288 mg). The product was characterized by ^{1}H NMR (90 °C in $C_2D_2Cl_4$, 400 MHz), 2D DOSY NMR, DSC, and high temperature size exclusion chromatography. Isolated yield = 60%; Graft yield = 53%

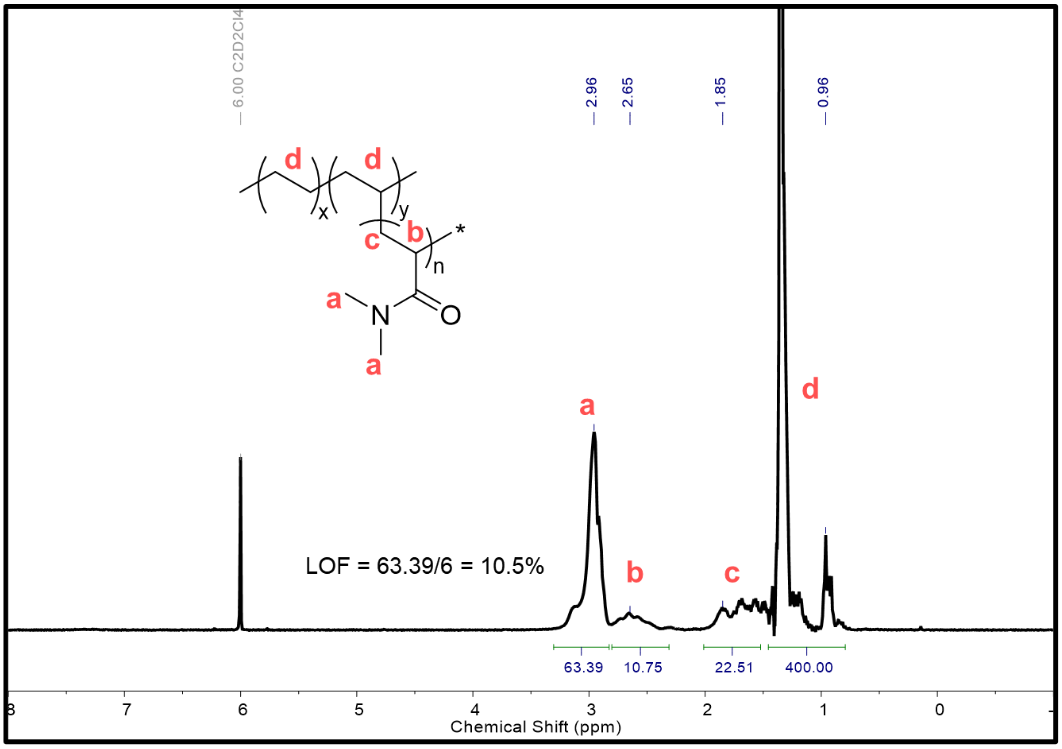

Figure S97. ^{1}H NMR spectrum (400 MHz, $C_2D_2Cl_4$) of LDPE–*g*–PNDMAM$_{10.5}$ (T = 90 °C)

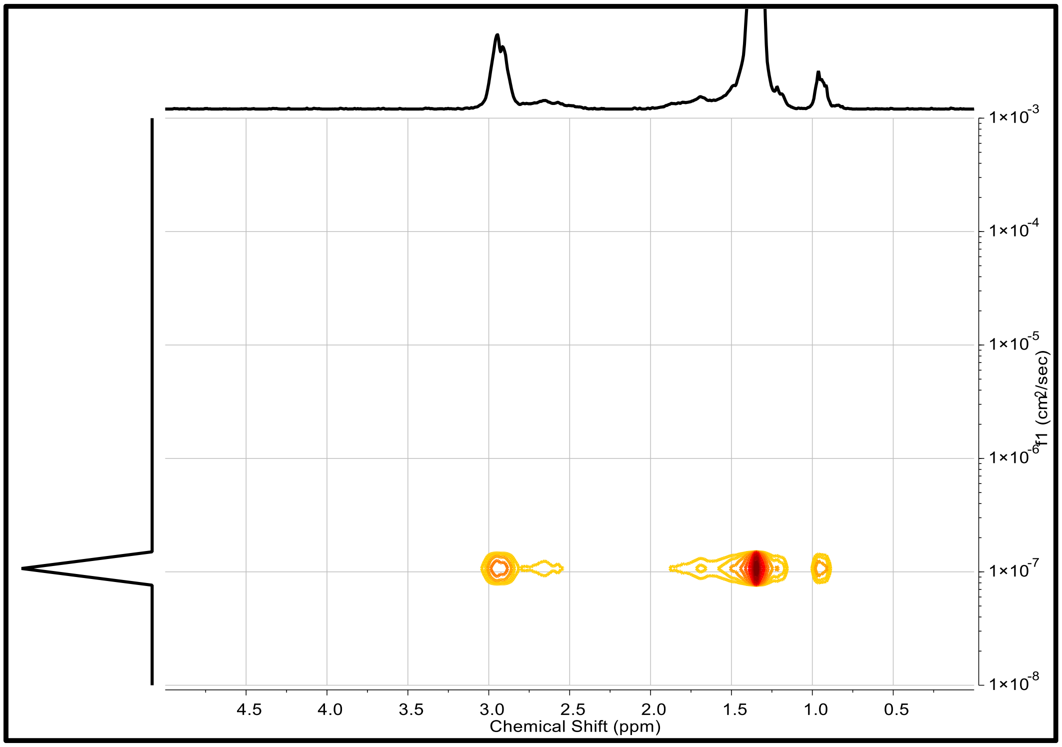

Figure S98. 2D DOSY NMR spectrum (400 MHz, $C_2D_2Cl_4$) of LDPE–*g*–PNDMAM$_{10.5}$ (T = 90 °C)

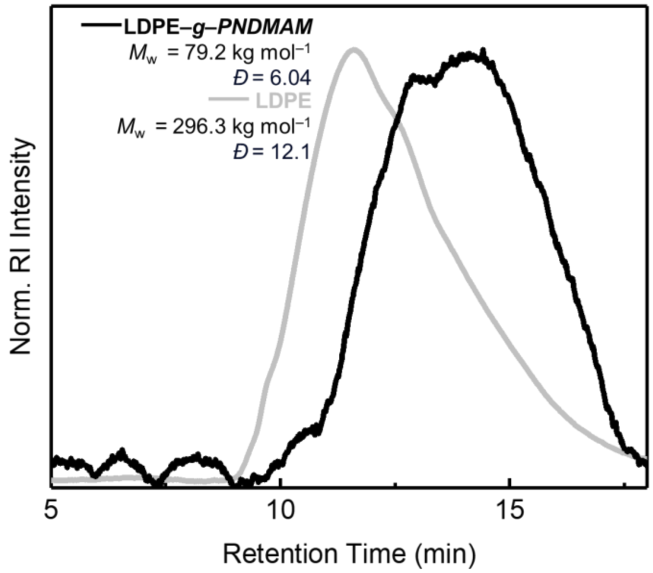

Figure S99. HT SEC trace of LDPE–*g*–PNDMAM$_{10.5}$ (1,2,4-TCB, 1.0 mL min^{-1} at 150 °C)

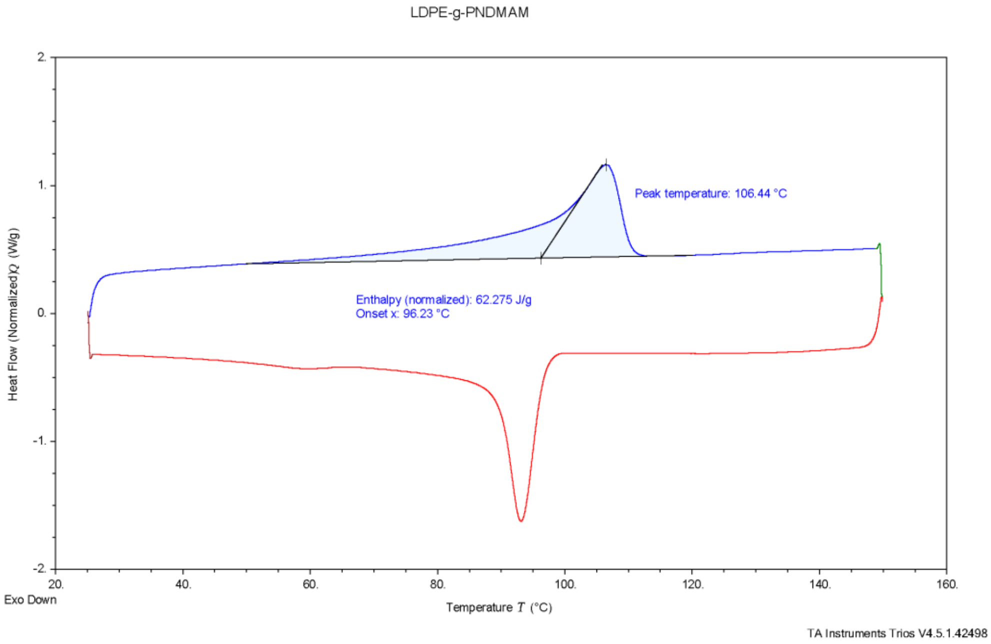

Figure S100. DSC Thermogram of LDPE–*g*–PNDMAM (f_{vinyl} = 10.5%). T_m = 106.44 °C, ΔH_m = 62.275 J g^{-1}, X_C = 21.3%.

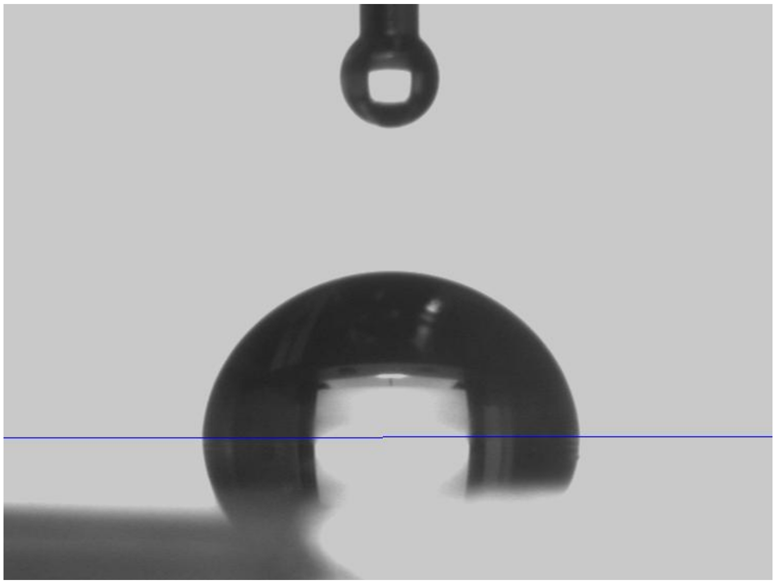

Figure S101. Water contact angle measured for LDPE–*g*–PNDMAM (f_{vinyl} = 10.5%). WCA = 88.0°

Synthesis of LDPE–*g*–PVA (Figure 5). A 22 mL borosilicate test tube was charged with LDPE (281 mg, 10 mmol) and 1,2-dichlorobenzene (10 mL), and sealed with a rubber septum. The mixture was degassed by nitrogen bubbling at 90 °C until it became homogeneous. Vinyl acetate (0.3 equiv., 0.28 mL, 3 mmol) was added via syringe. The reaction mixture was stirred and irradiated with a 390 nm LED lamp for 6 hours at 90 °C. Upon completion of the reaction, the mixture was precipitated in cold methanol (ca. 40 mL). The resulting solid was collected by filtration and washed via Soxhlet extraction with acetone for 3 h (ca. 4 min per cycle). After the purification, the solid was dried in a vacuum oven (3 mbar) at 80 °C for 12 h. to afford the desired **LDPE–*g*–PVA** as a white solid (273 mg). The product was characterized by ^{1}H NMR (90 °C in $C_2D_2Cl_4$, 400 MHz), 2D DOSY NMR, DSC, and high temperature size exclusion chromatography.
Isolated yield = 51%; Graft yield = 8.9%

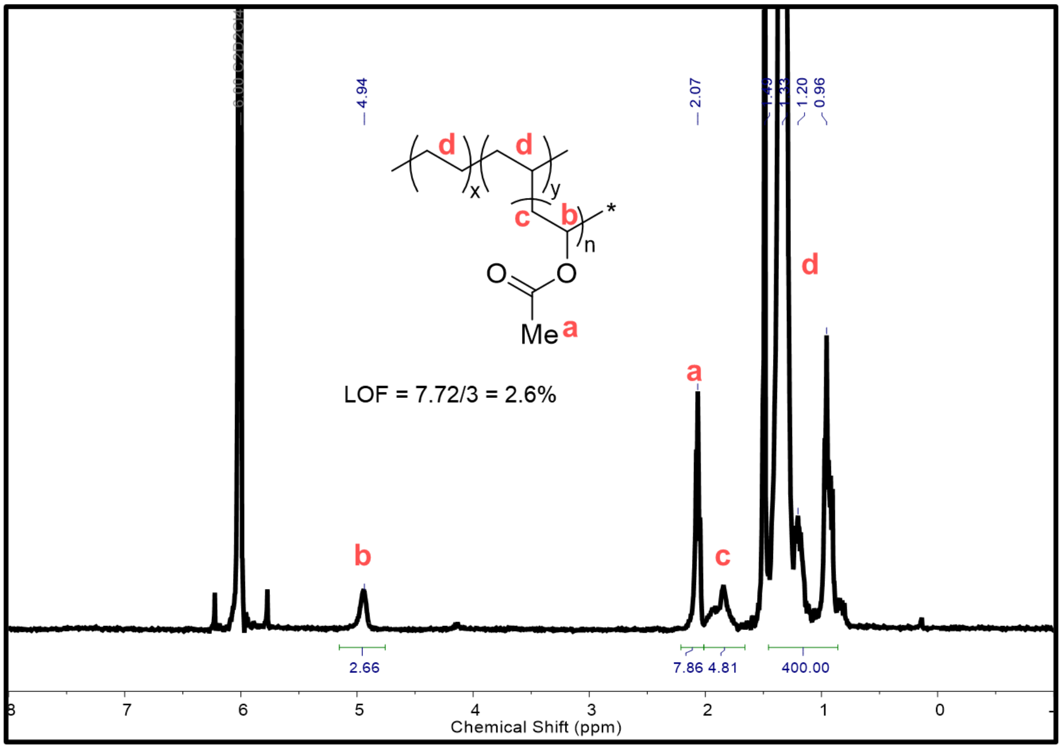

Figure S102. ^{1}H NMR spectrum (400 MHz, $C_2D_2Cl_4$) of LDPE–*g*–PVA$_{2.6}$ (T = 90 °C)

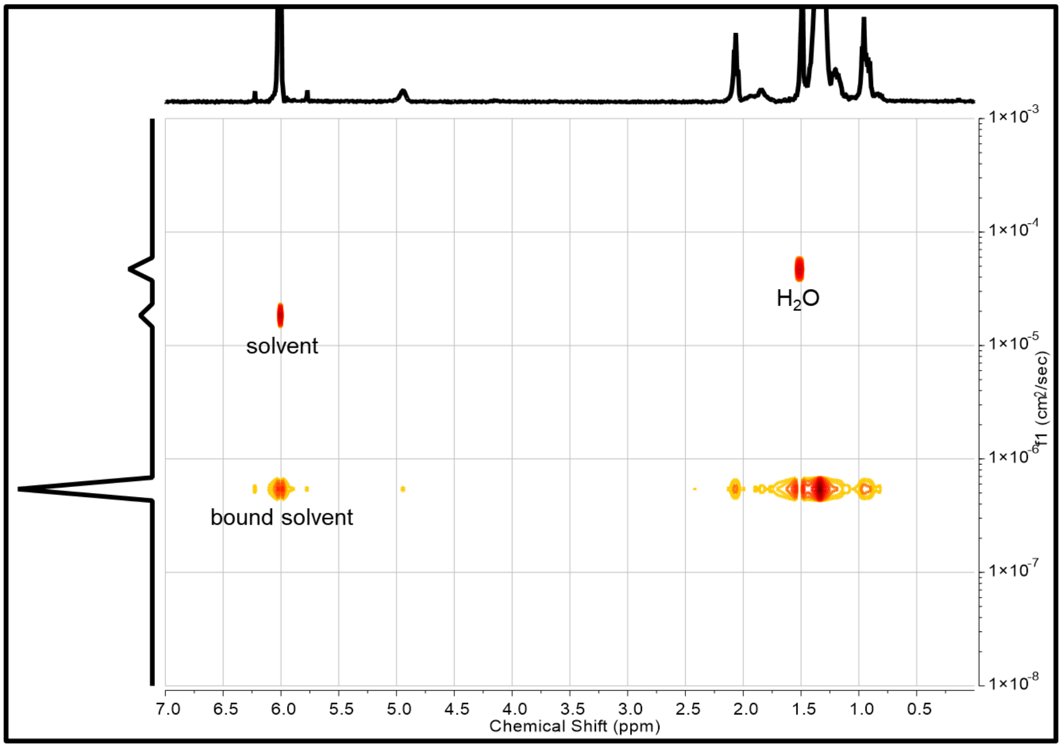

Figure S103. 2D DOSY NMR spectrum (400 MHz, $C_2D_2Cl_4$) of LDPE–*g*–PVA$_{2.6}$ (T = 90 °C)

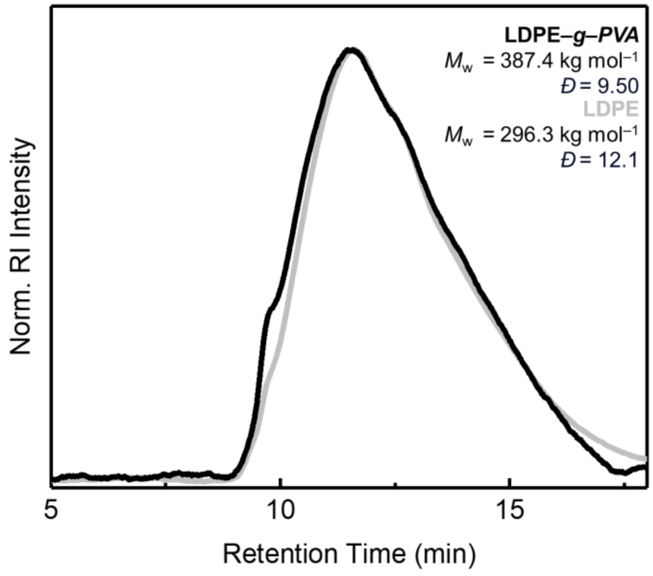

Figure S104. HT SEC trace of LDPE–*g*–PVA$_{2.6}$ (1,2,4-TCB, 1.0 mL min^{-1} at 150 °C)

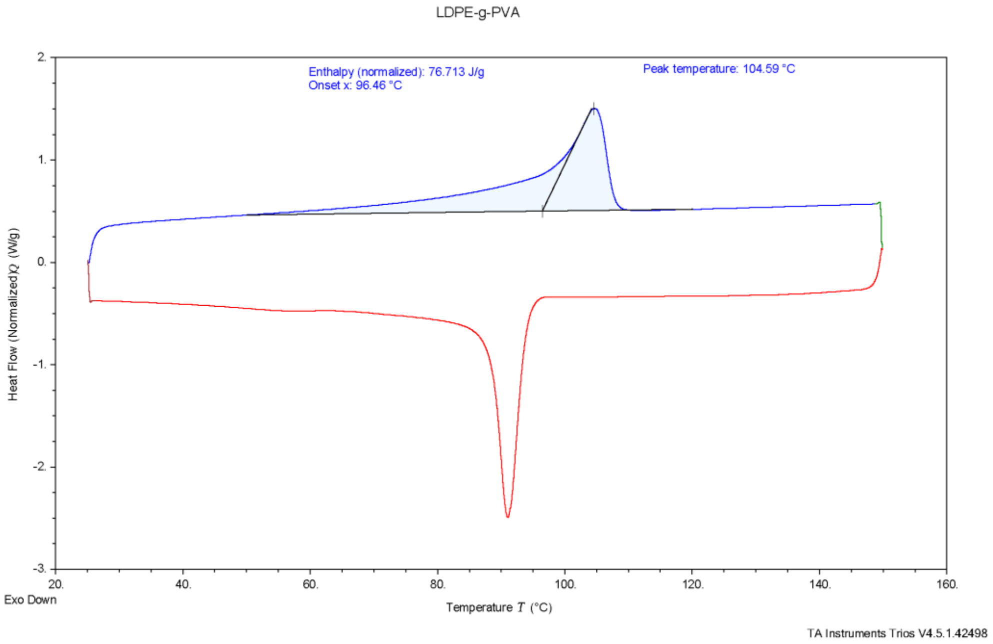

Figure S105. DSC Thermogram of LDPE–*g*–PVA (f_{vinyl} = 2.6%). T_m = 104.59 °C, ΔH_m = 76.713 J g^{-1}, X_C = 26.2%.

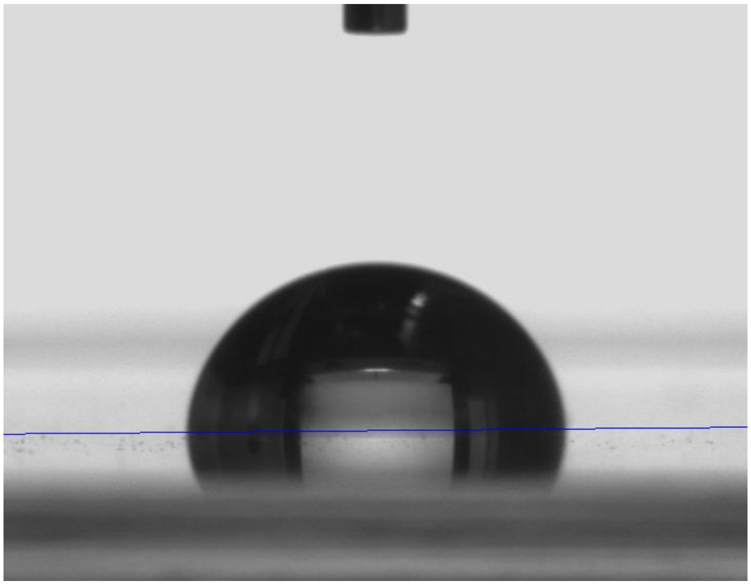

Figure S106. Water contact angle measured for LDPE–*g*–PVA (f_{vinyl} = 2.6%). WCA = 87.7°

Synthesis of LDPE–*g*–PVP (Figure 5). A 22 mL borosilicate test tube was charged with LDPE (281 mg, 10 mmol) and 1,2-dichlorobenzene (10 mL), and sealed with a rubber septum. The mixture was degassed by nitrogen bubbling at 90 °C until it became homogeneous. *N*-Vinylpyrrolidone (0.2 equiv., 0.21 mL, 2 mmol) was added via syringe. The reaction mixture was stirred and irradiated with a 390 nm LED lamp for 6 hours at 90 °C. Upon completion of the reaction, the mixture was precipitated in cold methanol (ca. 40 mL). The resulting solid was collected by filtration and washed via Soxhlet extraction with acetone for 6 h (ca. 4 min per cycle). After the purification, the solid was dried in a vacuum oven (3 mbar) at 80 °C for 12 h. to afford the desired **LDPE–*g*–PVP** as a white solid (288 mg). The product was characterized by ^{1}H NMR (90 °C in $C_2D_2Cl_4$, 400 MHz), 2D DOSY NMR, DSC, and high temperature size exclusion chromatography. Isolated yield = 57%; Graft yield = 16%

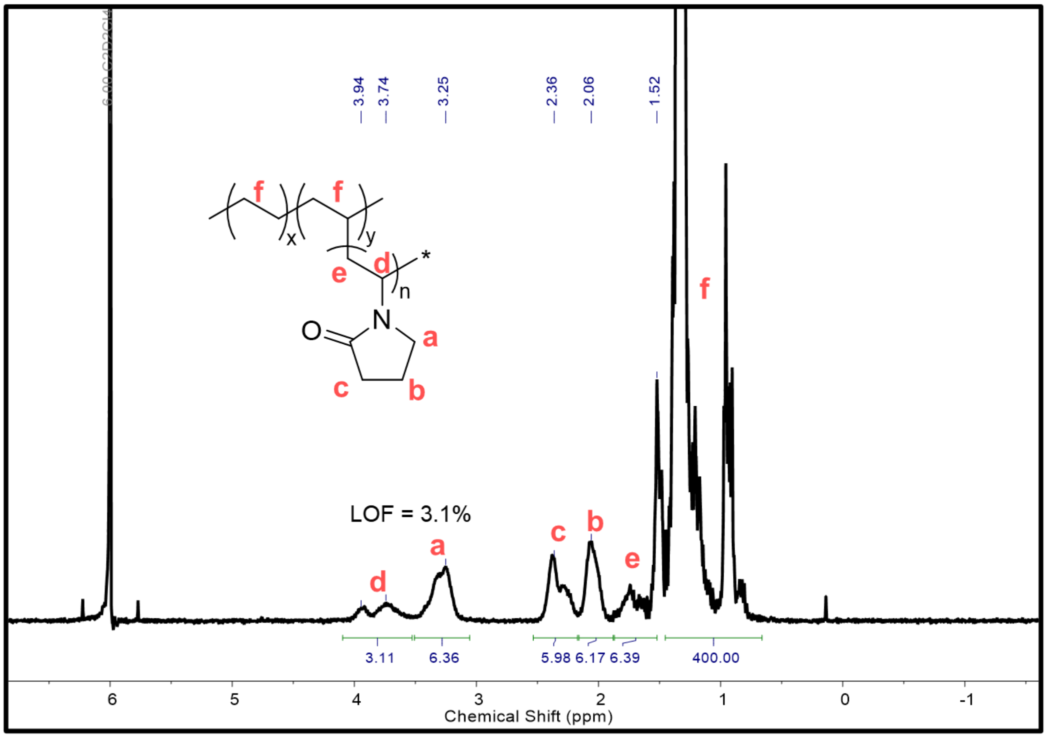

Figure S107. ^{1}H NMR spectrum (400 MHz, $C_2D_2Cl_4$) of LDPE–*g*–$PVP_{3.1}$ (T = 90 °C)

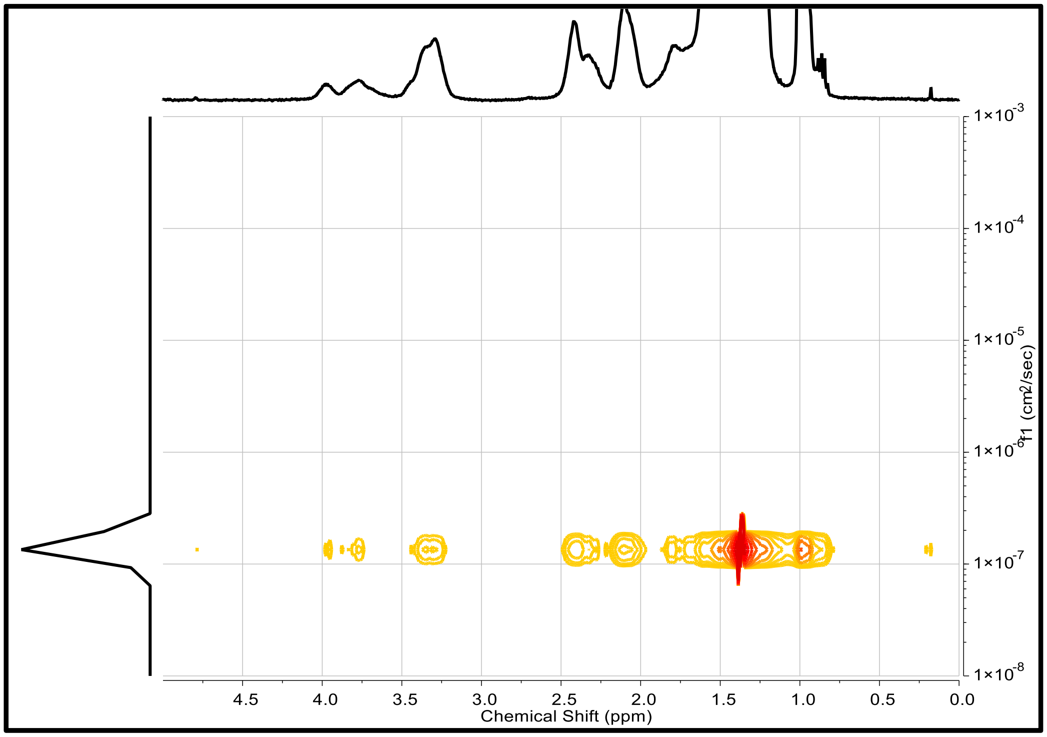

Figure S108. 2D DOSY NMR spectrum (400 MHz, $C_2D_2Cl_4$) of LDPE–*g*–PVP$_{3.1}$ (T = 90 °C)

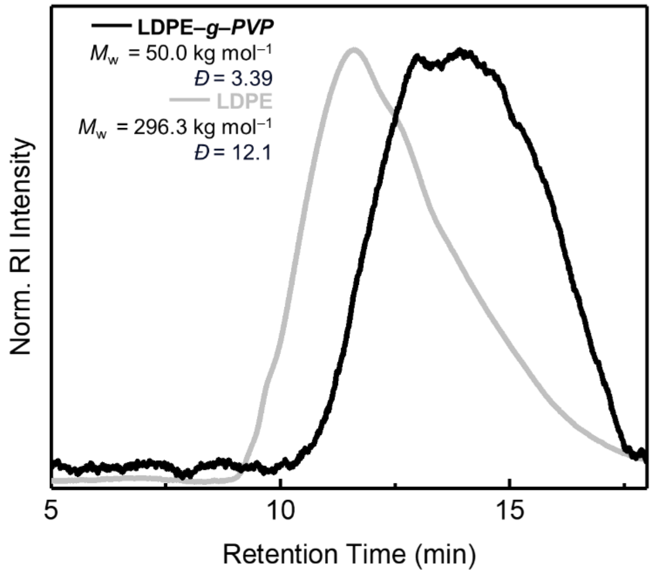

Figure S109. HT SEC trace of LDPE–*g*–PVP$_{3.1}$ (1,2,4-TCB, 1.0 mL min^{-1} at 150 °C)

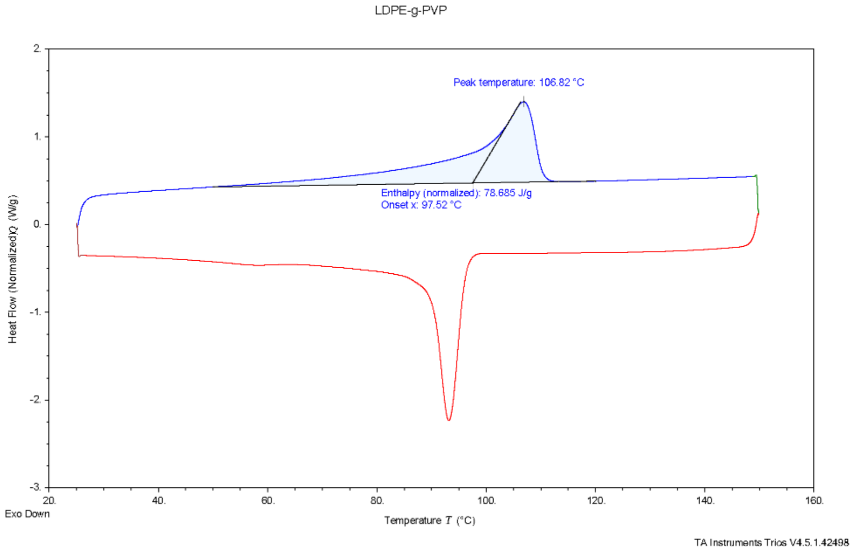

Figure S110. DSC Thermogram of LDPE–*g*–PVP (f_{vinyl} = 3.1%). T_m = 106.82 °C, ΔH_m = 78.685 J g^{-1}, X_C = 26.9%.

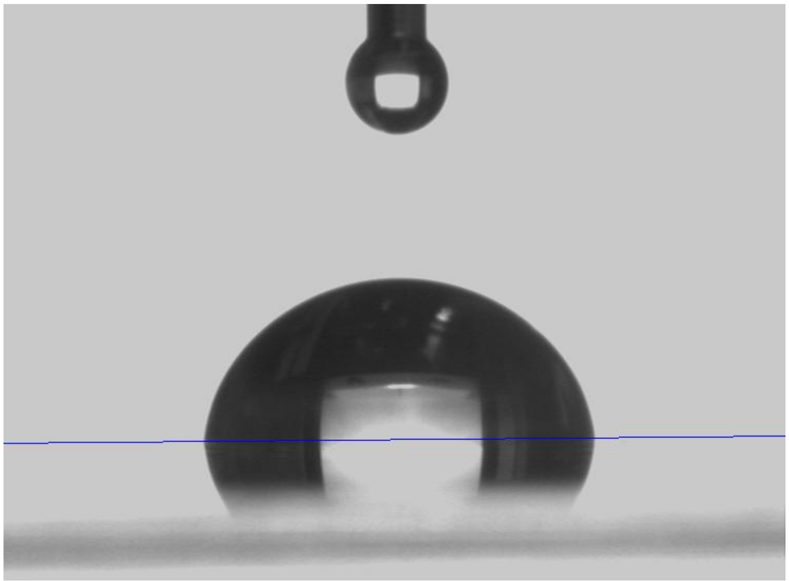

Figure S111. Water contact angle measured for LDPE–*g*–PVP (f_{vinyl} = 3.1%). WCA = 83.4°

Synthesis of LDPE–*g*–PS (Figure 5). A 22 mL borosilicate test tube was charged with LDPE (281 mg, 10 mmol) and 1,2-dichlorobenzene (10 mL), and sealed with a rubber septum. The mixture was degassed by nitrogen bubbling at 90 °C until it became homogeneous. Styrene (0.3 equiv., 0.34 mL, 3 mmol) was added via syringe. The reaction mixture was stirred and irradiated with a 390 nm LED lamp for 12 hours at 90 °C. Upon completion of the reaction, the mixture was precipitated in cold methanol (ca. 40 mL). The resulting solid was collected by filtration and washed via Soxhlet extraction with acetone for 6 h (ca. 4 min per cycle). After the purification, the solid was dried in a vacuum oven (3 mbar) at 80 °C for 12 h. to afford the desired **LDPE–*g*–PS** as a white solid (269 mg). The product was characterized by ^{1}H NMR (90 °C in $C_2D_2Cl_4$, 400 MHz), 2D DOSY NMR, DSC, and high temperature size exclusion chromatography. Isolated yield = 45%; Graft yield = 12%

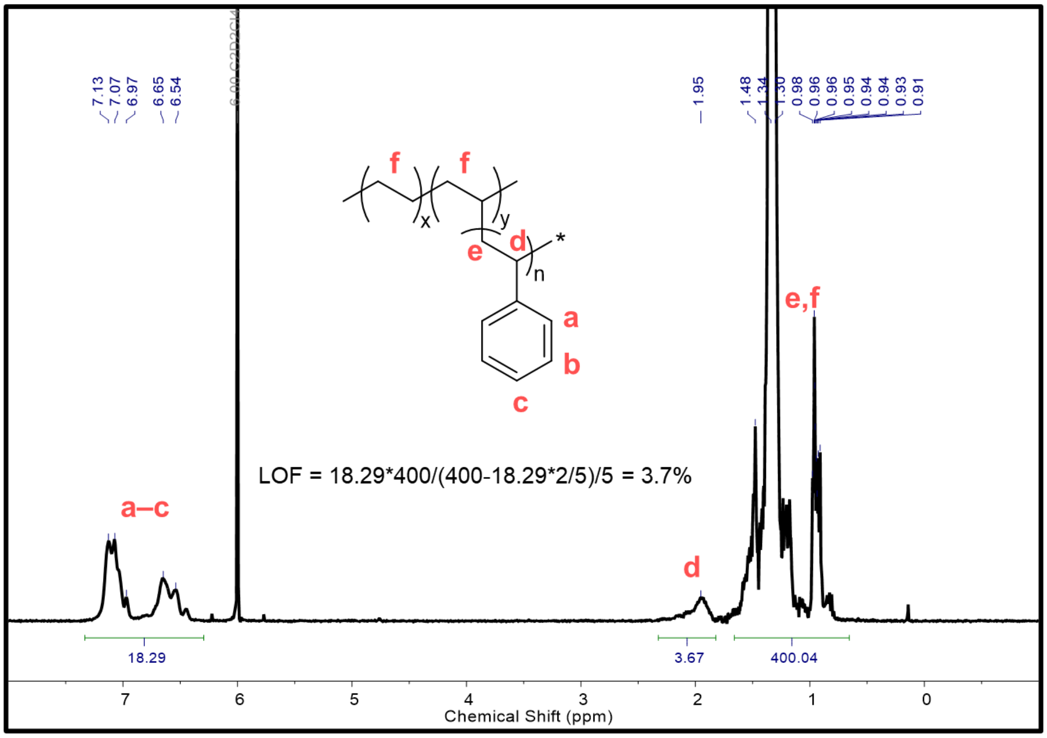

Figure S112. ^{1}H NMR spectrum (400 MHz, $C_2D_2Cl_4$) of LDPE–*g*–$PS_{3.7}$ (T = 90 °C)

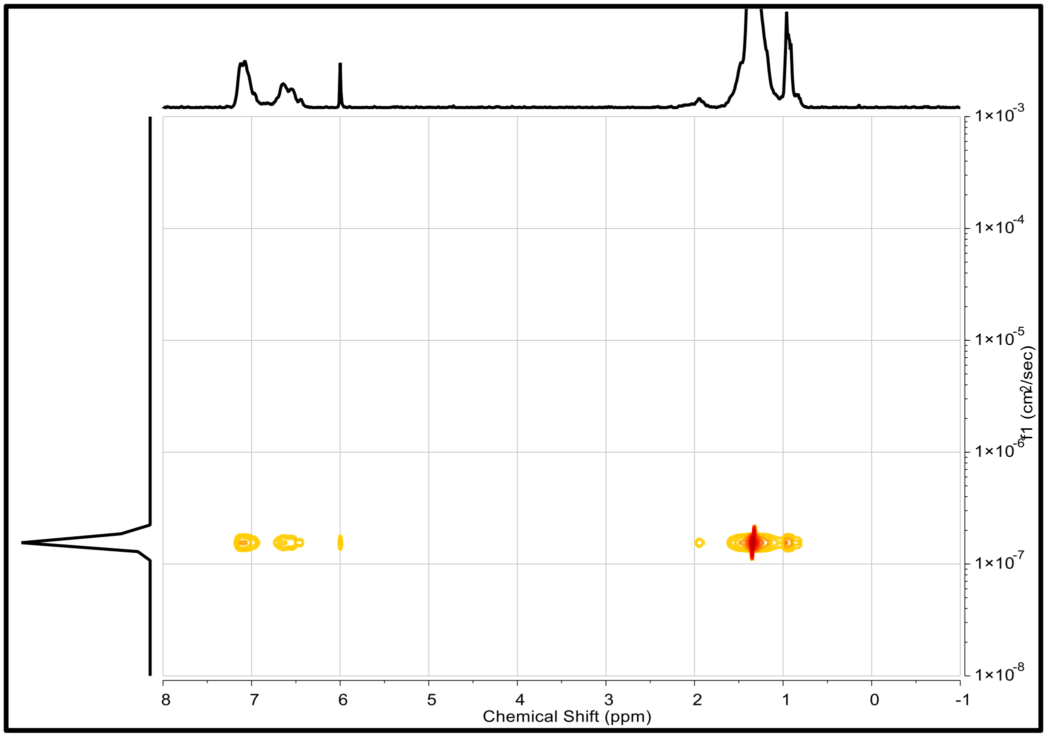

Figure S113. 2D DOSY NMR spectrum (400 MHz, $C_2D_2Cl_4$) of LDPE–*g*–$PS_{3.7}$ (T = 90 °C)

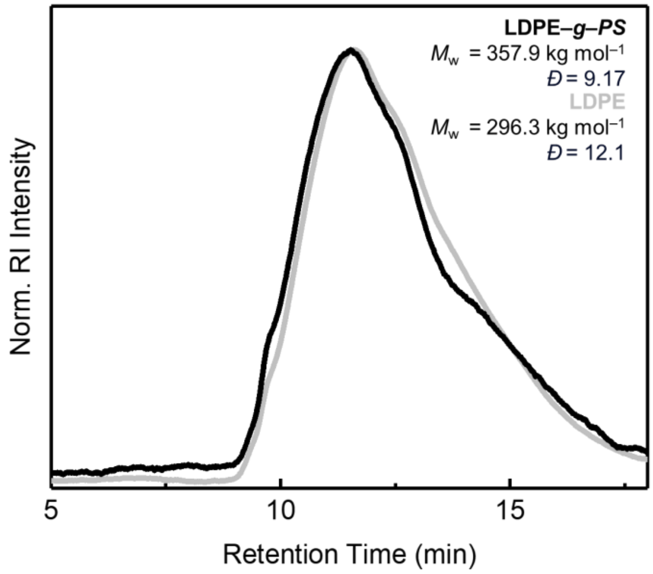

Figure S114. HT SEC trace of LDPE–*g*–$PS_{3.7}$ (1,2,4-TCB, 1.0 mL min^{-1} at 150 °C)

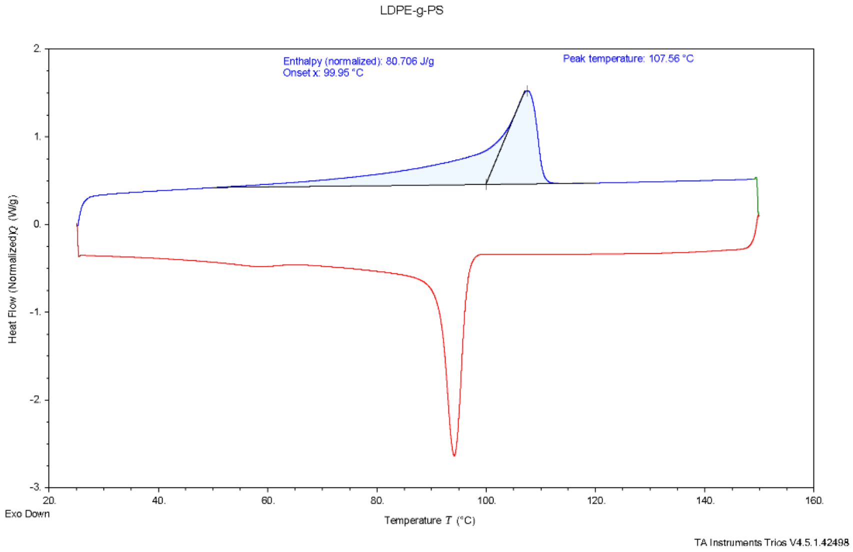

Figure S115. DSC Thermogram of LDPE–*g*–PS (f_{vinyl} = 3.7%). T_m = 107.56 °C, ΔH_m = 80.706 J g^{-1}, X_C = 27.5%.

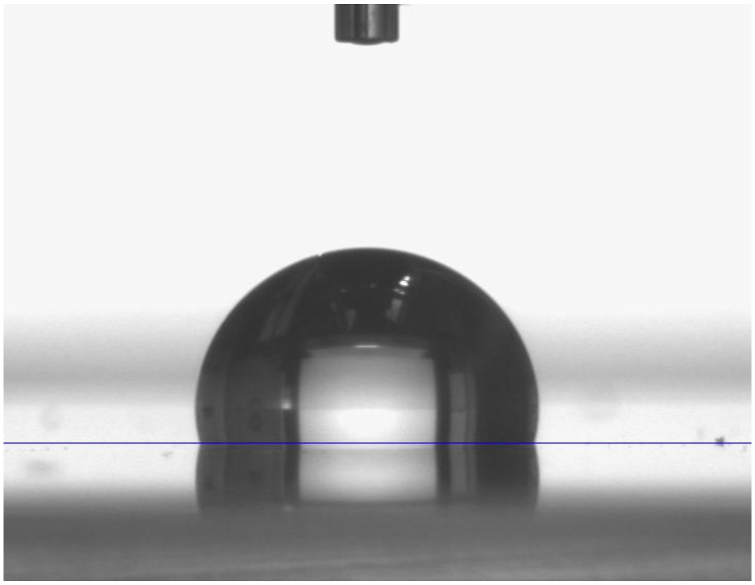

Figure S116. Water contact angle measured for LDPE–*g*–PS (f_{vinyl} = 3.7%). WCA =103.0°

Synthesis of LDPE–*g*–P(4-ClS) (Figure 5). A 22 mL borosilicate test tube was charged with LDPE (281 mg, 10 mmol) and 1,2-dichlorobenzene (10 mL), and sealed with a rubber septum. The mixture was degassed by nitrogen bubbling at 90 °C until it became homogeneous. 4-chlorostyrene (0.3 equiv., 0.36 mL, 3 mmol) was added via syringe. The reaction mixture was stirred and irradiated with a 390 nm LED lamp for 6 hours at 90 °C. Upon completion of the reaction, the mixture was precipitated in cold methanol (ca. 40 mL). The resulting solid was collected by filtration and washed via Soxhlet extraction with dichloromethane for 3 h (ca. 3 min per cycle). After the purification, the solid was dried in a vacuum oven (3 mbar) at 80 °C for 12 h. to afford the desired **LDPE–*g*–P(4-ClS)** as a white solid (261 mg). The product was characterized by ^{1}H NMR (90 °C in $C_2D_2Cl_4$, 400 MHz), 2D DOSY NMR, DSC, and high temperature size exclusion chromatography. Isolated yield = 38%; Graft yield = 5.3%

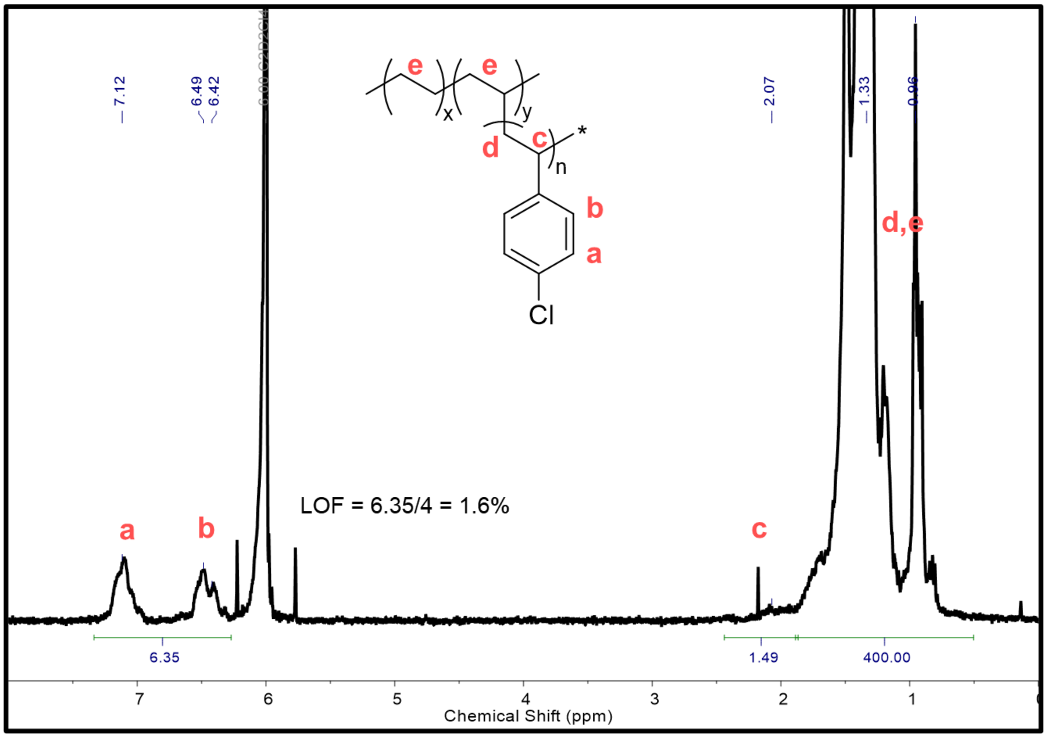

Figure S117. ^{1}H NMR spectrum (400 MHz, $C_2D_2Cl_4$) of LDPE–*g*–P(4-ClS)$_{1.6}$ (T = 90 °C)

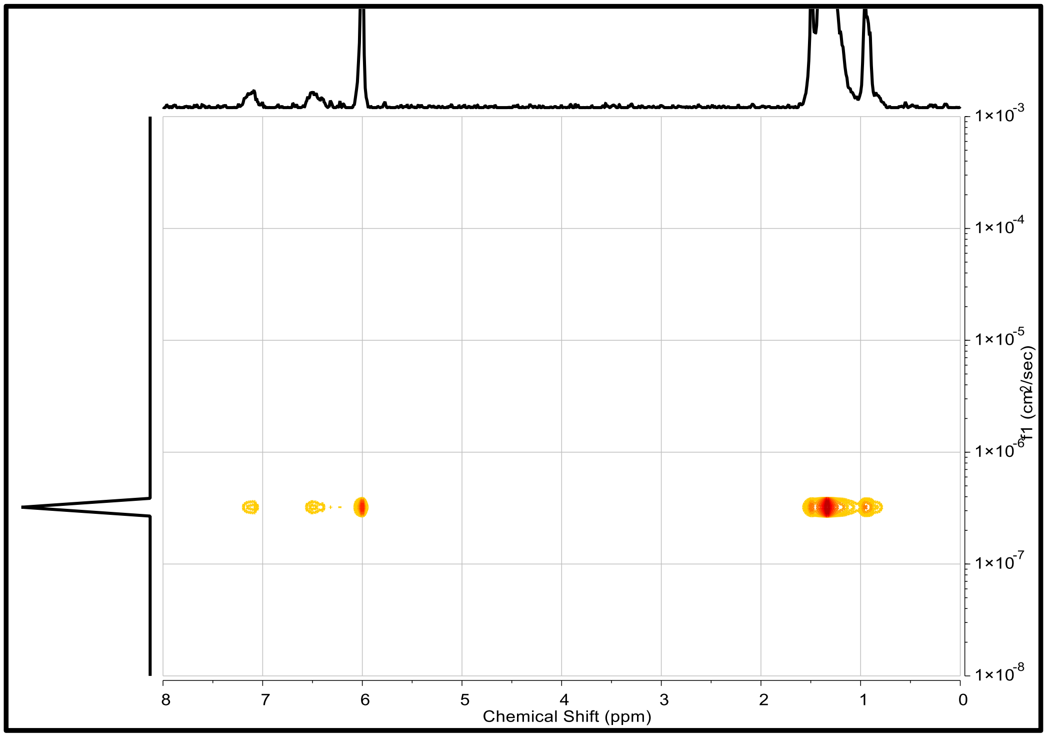

Figure S118. 2D DOSY NMR spectrum (400 MHz, $C_2D_2Cl_4$) of LDPE–*g*–P(4-ClS)$_{1.6}$ (T = 90 °C)

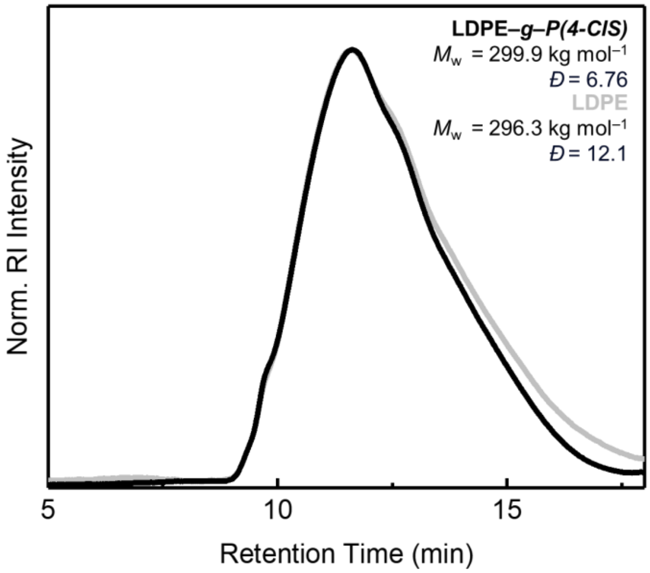

Figure S119. HT SEC trace of LDPE–*g*–P(4-ClS)$_{1.6}$ (1,2,4-TCB, 1.0 mL min^{-1} at 150 °C)

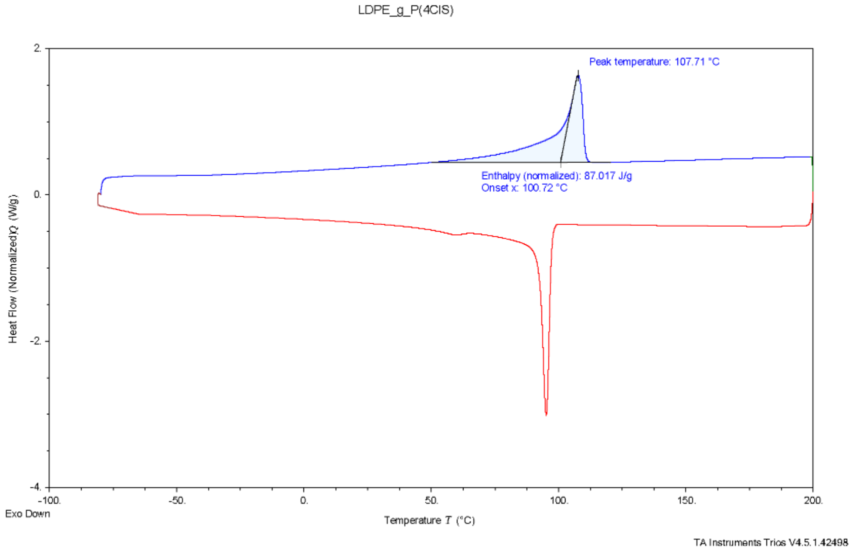

Figure S120. DSC Thermogram of LDPE–*g*–P(4-ClS) (f_{vinyl} = 1.6%). T_m = 107.71 °C, ΔH_m = 87.02 J g^{-1}, X_C = 29.1%.

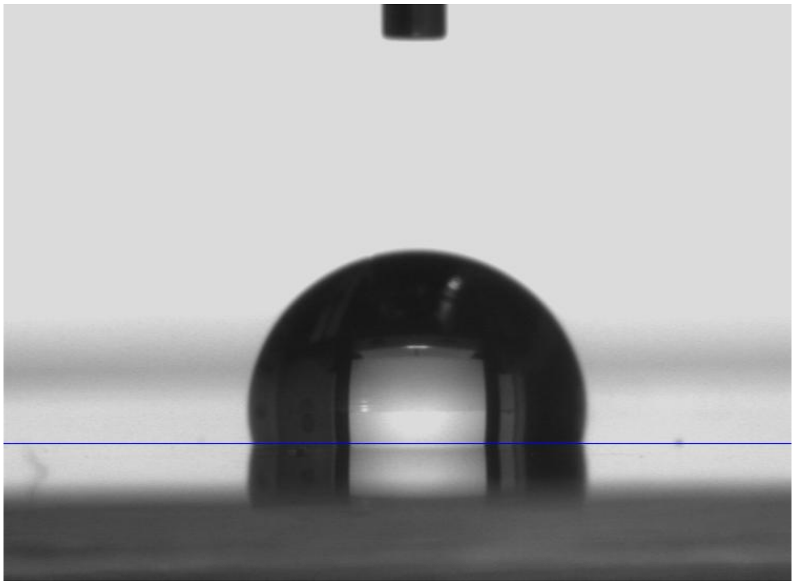

Figure S121. Water contact angle measured for LDPE–*g*–P(4-ClS) (f_{vinyl} = 1.6%). WCA = 104.3°

Synthesis of LDPE–*g*–P(4-BrS) (Figure 5). A 22 mL borosilicate test tube was charged with LDPE (281 mg, 10 mmol) and 1,2-dichlorobenzene (10 mL), and sealed with a rubber septum. The mixture was degassed by nitrogen bubbling at 90 °C until it became homogeneous. 4-bromostyrene (0.3 equiv., 0.40 mL, 3 mmol) was added via syringe. The reaction mixture was stirred and irradiated with a 390 nm LED lamp for 12 hours at 90 °C. Upon completion of the reaction, the mixture was precipitated in cold methanol (ca. 40 mL). The resulting solid was collected by filtration and washed via Soxhlet extraction with acetone for 6 h (ca. 4 min per cycle). After the purification, the solid was dried in a vacuum oven (3 mbar) at 80 °C for 12 h. to afford the desired **LDPE–*g*–P(4-BrS)** as a white solid (268 mg). The product was characterized by ^{1}H NMR (90 °C in $C_2D_2Cl_4$, 400 MHz), 2D DOSY NMR, DSC, and high temperature size exclusion chromatography. Isolated yield = 32%; Graft yield = 3.1%

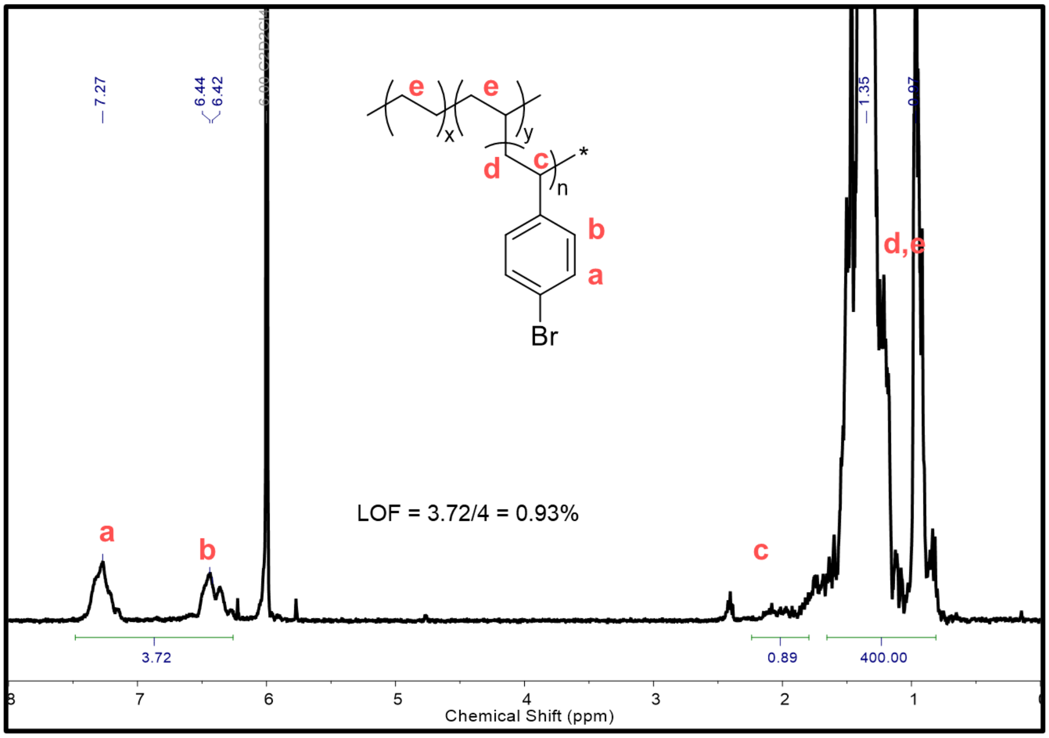

Figure S122. ^{1}H NMR spectrum (400 MHz, $C_2D_2Cl_4$) of LDPE–*g*–P(4-BrS)$_{0.9}$ (T = 90 °C)

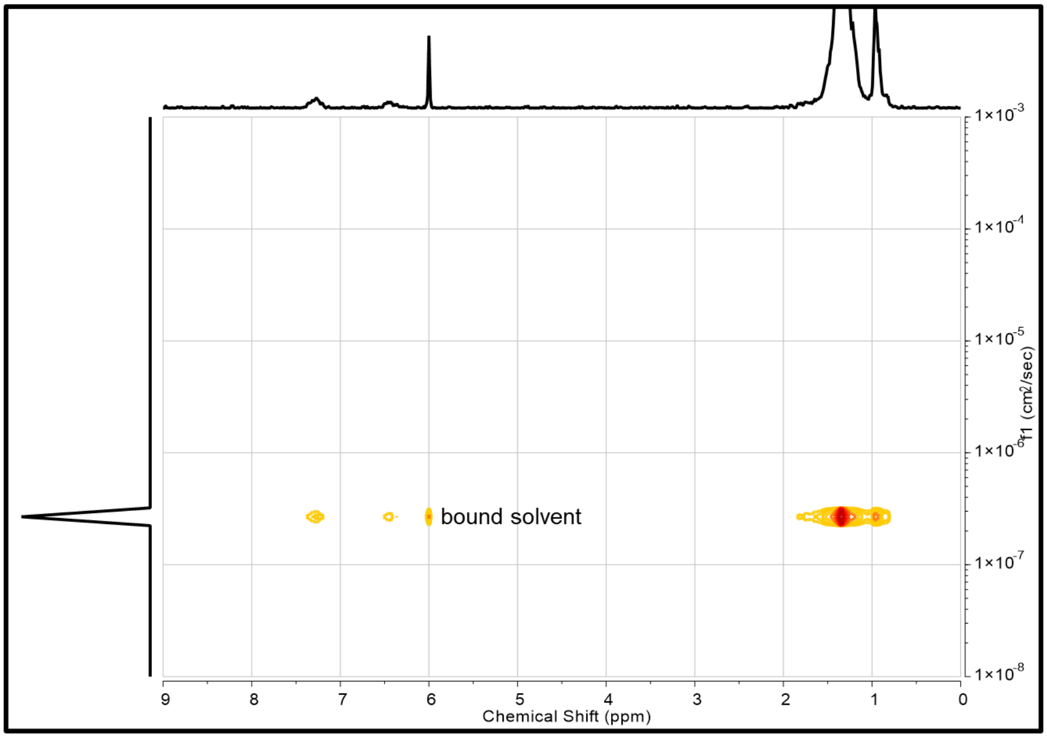

Figure S123. 2D DOSY NMR spectrum (400 MHz, $C_2D_2Cl_4$) of LDPE–*g*–P(4-BrS)$_{0.9}$ (T = 90 °C)

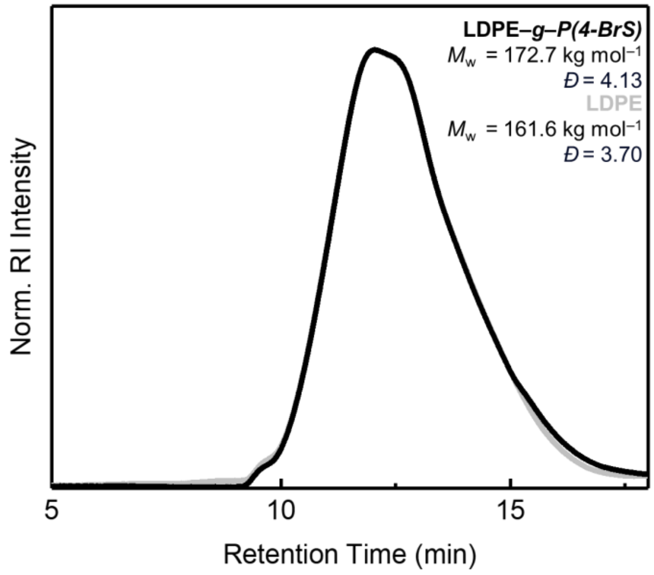

Figure S124. HT SEC trace of LDPE–*g*–P(4-BrS)$_{0.9}$ (1,2,4-TCB, 1.0 mL min^{-1} at 150 °C)

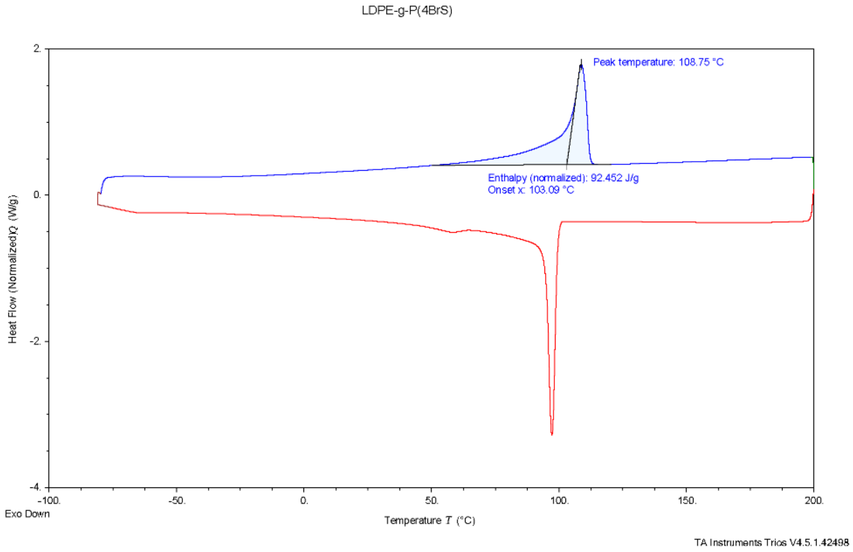

Figure S125. DSC Thermogram of LDPE–*g*–P(4-BrS) (f_{vinyl} = 0.9%). T_m = 108.75 °C, ΔH_m = 92.45 J g^{-1}, X_C = 31.4%.

Synthesis of LLDPE–*g*–PMA (Figure 5). A 22 mL borosilicate test tube was charged with LLDPE (281 mg, 10 mmol) and 1,2-dichlorobenzene (10 mL), and sealed with a rubber septum. The mixture was degassed by nitrogen bubbling at 105 °C until it became homogeneous. Methyl acrylate (0.3 equiv., 0.27 mL, 3 mmol) was added via syringe. The reaction mixture was stirred and irradiated with a 390 nm LED lamp for 6 hours at 105 °C. Upon completion of the reaction, the mixture was precipitated in cold methanol (ca. 40 mL). The resulting solid was collected by filtration and washed via Soxhlet extraction with acetone for 3 h (ca. 4 min per cycle). After the purification, the solid was dried in a vacuum oven (3 mbar) at 80 °C for 12 h. to afford the desired **LDPE–*g*–PMA** as a white solid (277 mg). The product was characterized by ^{1}H NMR (90 °C in $C_2D_2Cl_4$, 400 MHz), 2D DOSY NMR, DSC, and high temperature size exclusion chromatography. Isolated yield = 51%; Graft yield = 9.0%

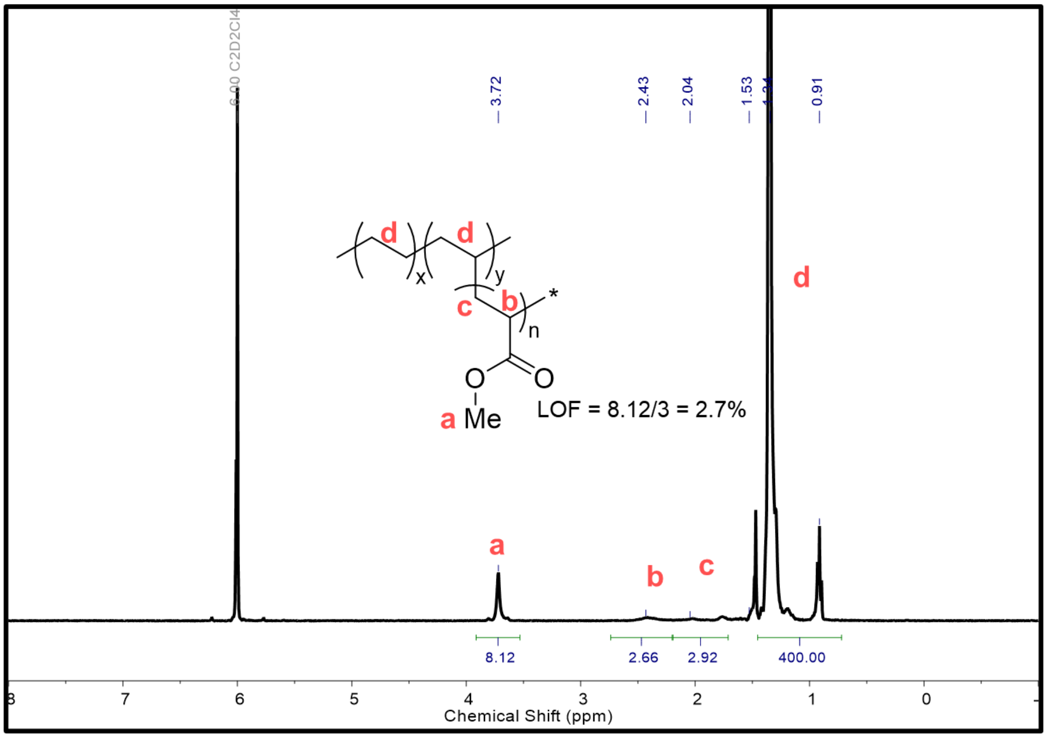

Figure S126. ^{1}H NMR spectrum (400 MHz, $C_2D_2Cl_4$) of LLDPE–*g*–$PMA_{2.7}$ (*T* = 105 °C)

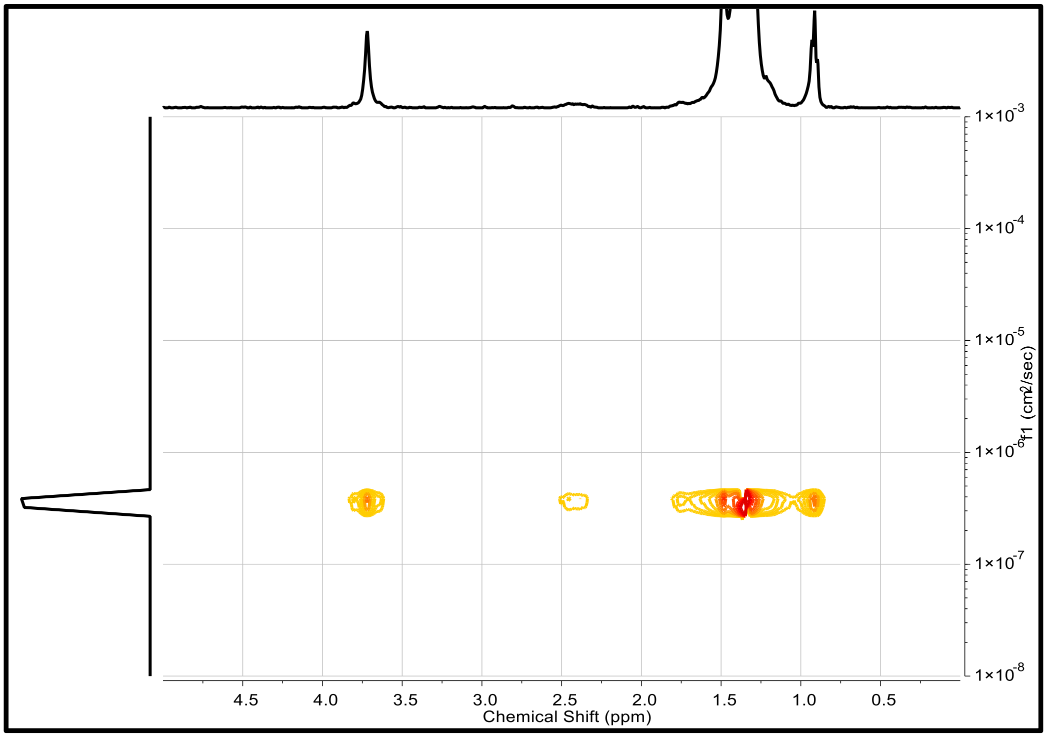

Figure S127. 2D DOSY NMR spectrum (400 MHz, $C_2D_2Cl_4$) of LLDPE–*g*–$PMA_{2.7}$ (T = 105 °C)

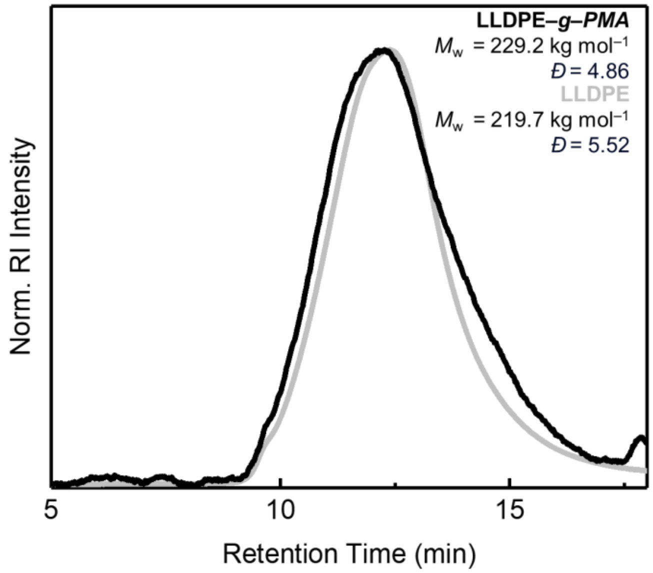

Figure S128. HT SEC trace of LLDPE–*g*–PMA (1,2,4-TCB, 1.0 mL min^{-1} at 150 °C)

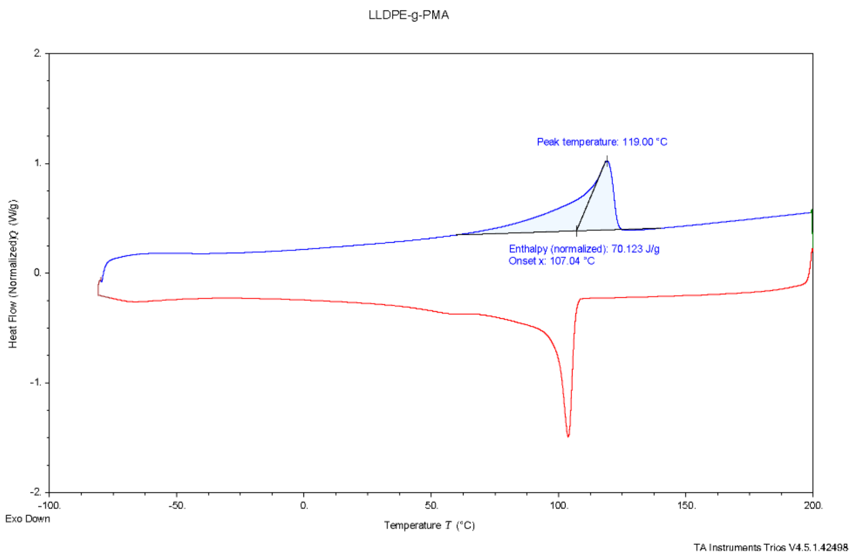

Figure S129. DSC Thermogram of LLDPE–*g*–PMA (f_{vinyl} = 2.7%). T_m = 119.00 °C, ΔH_m = 70.123 J g^{-1}, X_C = 23.9%.

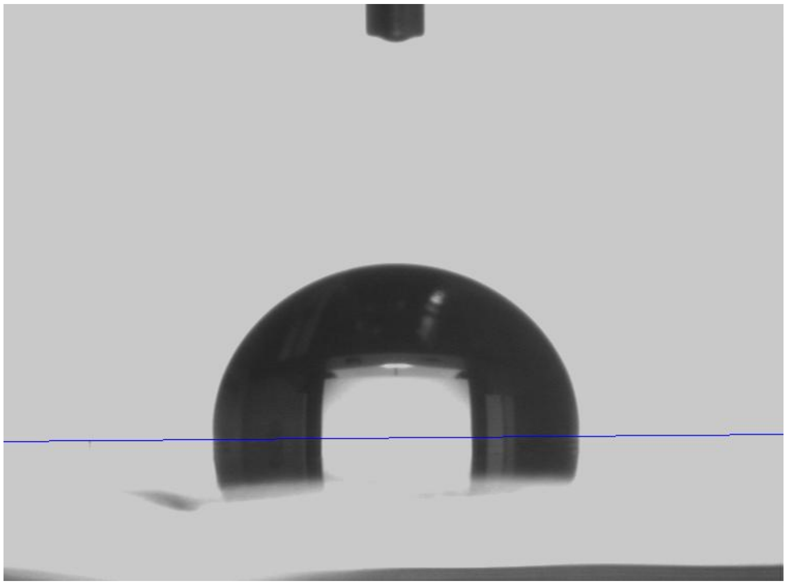

Figure S130. Water contact angle measured for LLDPE–*g*–PMA (f_{vinyl} = 2.7%). WCA = 94.2°

Synthesis of LLDPE–*g*–PMMA (Figure 5). A 22 mL borosilicate test tube was charged with LLDPE (281 mg, 10 mmol) and 1,2-dichlorobenzene (10 mL),and sealed with a rubber septum. The mixture was degassed by nitrogen bubbling at 105 °C until it became homogeneous. Methyl methacrylate (0.3 equiv., 0.32 mL, 3 mmol) was added via syringe. The reaction mixture was stirred and irradiated with a 390 nm LED lamp for 12 hours at 105 °C. Upon completion of the reaction, the mixture was precipitated in cold methanol (ca. 40 mL). The resulting solid was collected by filtration and washed via Soxhlet extraction with acetone for 3 h (ca. 4 min per cycle). After the purification, the solid was dried in a vacuum oven (3 mbar) at 80 °C for 12 h. to afford the desired **LLDPE–*g*–PMMA** as a white solid (283 mg). The product was characterized by ^{1}H NMR (90 °C in $C_2D_2Cl_4$, 400 MHz), 2D DOSY NMR, DSC, and high temperature size exclusion chromatography. Isolated yield = 49%; Graft yield = 22%

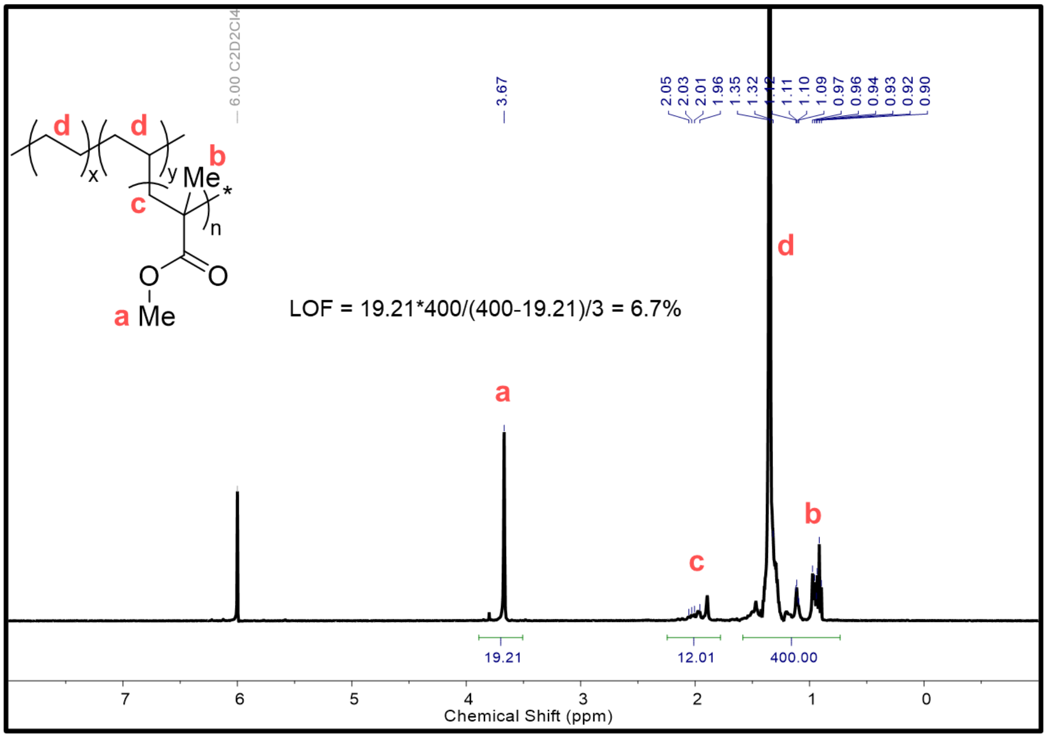

Figure S131. ^{1}H NMR spectrum (400 MHz, $C_2D_2Cl_4$) of LLDPE–*g*–PMMA$_{6.7}$ (T = 105 °C)

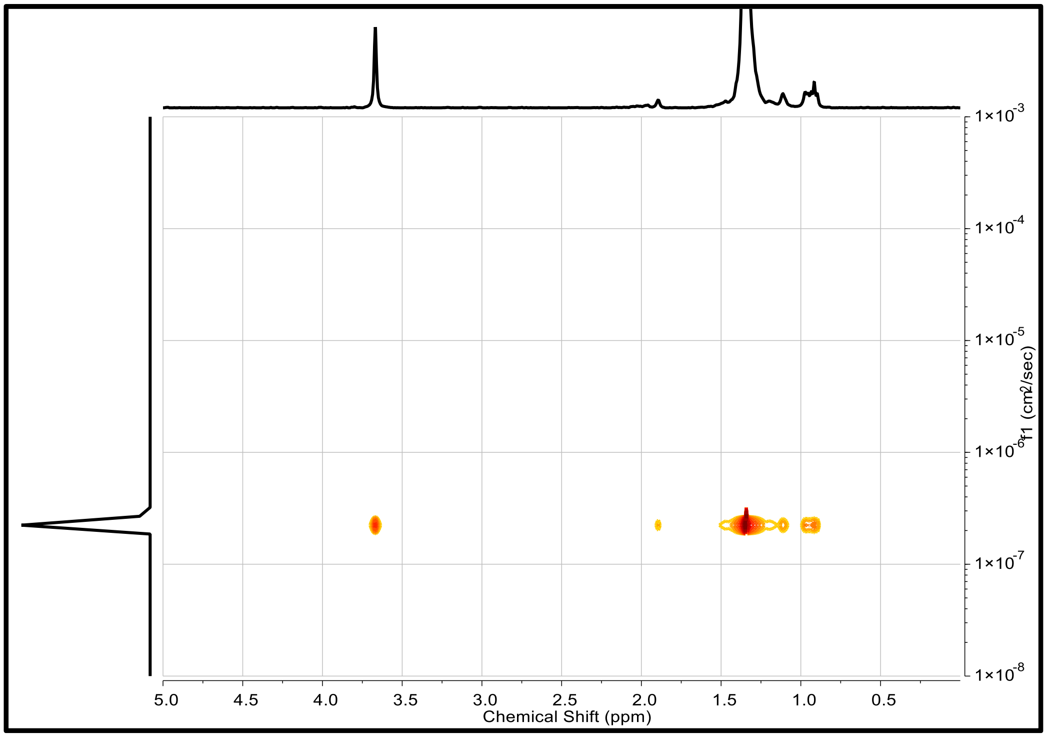

Figure S132. 2D DOSY NMR spectrum (400 MHz, $C_2D_2Cl_4$) of LLDPE–*g*–PMMA$_{6.7}$ (T = 105 °C)

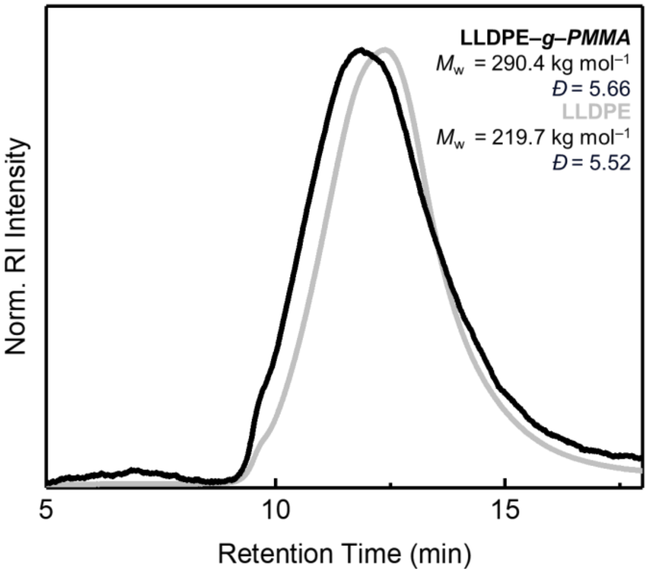

Figure S133. HT SEC trace of LLDPE–*g*–PMMA (1,2,4-TCB, 1.0 mL min^{-1} at 150 °C)

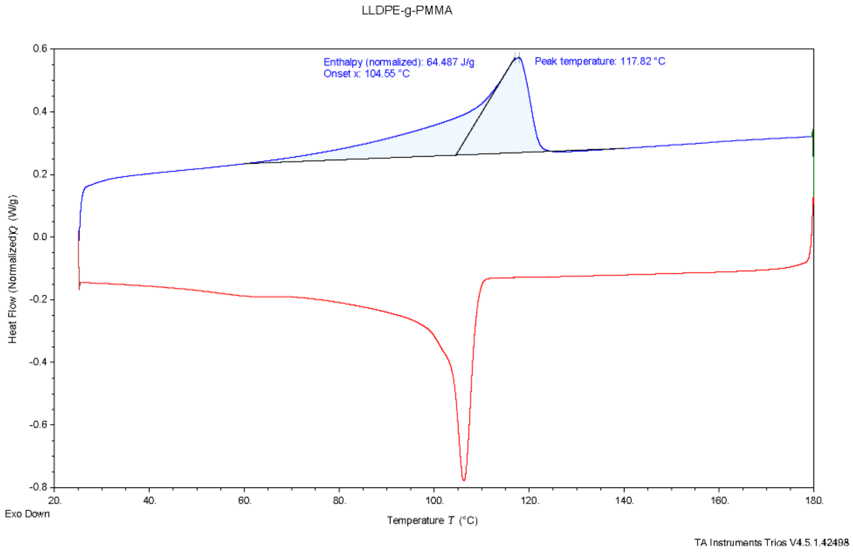

Figure S134. DSC Thermogram of LLDPE–*g*–PMMA (f_{vinyl} = 6.7%). T_m = 117.82 °C, ΔH_m = 64.487 J g^{-1}, X_C = 23.7%.

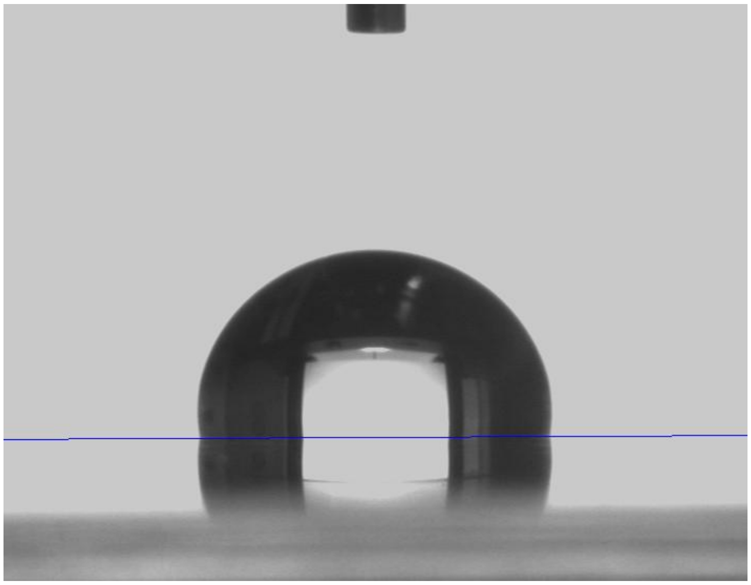

Figure S135. Water contact angle measured for LLDPE–*g*–PMMA (f_{vinyl} = 6.7%). WCA = 100.0°

Synthesis of LLDPE–*g*–PNIPAM (Figure 5). A 22 mL borosilicate test tube was charged with LLDPE (281 mg, 10 mmol) and 1,2-dichlorobenzene (8 mL),and sealed with a rubber septum. The mixture was degassed by nitrogen bubbling at 120 °C until it became homogeneous. *N*-isopropyl acrylamide (0.2 equiv., 226 mg, 2 mmol) in 2 mL of DCB was added via syringe. The reaction mixture was stirred and irradiated with a 390 nm LED lamp for 6 hours at 120 °C. Upon completion of the reaction, the mixture was precipitated in cold methanol (ca. 40 mL). The resulting solid was collected by filtration and washed via Soxhlet extraction with acetone for 6 h (ca. 4 min per cycle). After the purification, the solid was dried in a vacuum oven (3 mbar) at 80 °C for 12 h. to afford the desired **LLDPE–*g*–PNIPAM** as a white solid (288 mg). The product was characterized by ^1H NMR (90 °C in $C_2D_2Cl_4$, 400 MHz), 2D DOSY NMR, DSC, and high temperature size exclusion chromatography. Isolated yield = 57%; Graft yield = 32%

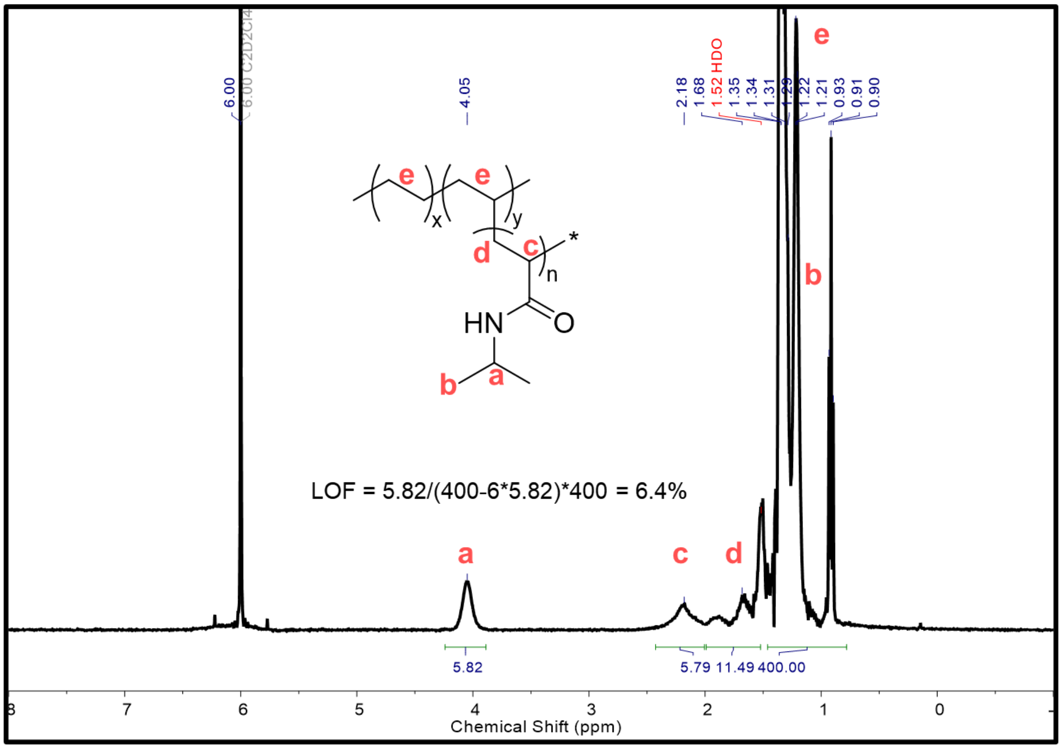

Figure S136. ^1H NMR spectrum (400 MHz, $C_2D_2Cl_4$) of LLDPE–*g*–PNIPAM$_{6.4}$ (T = 105 °C)

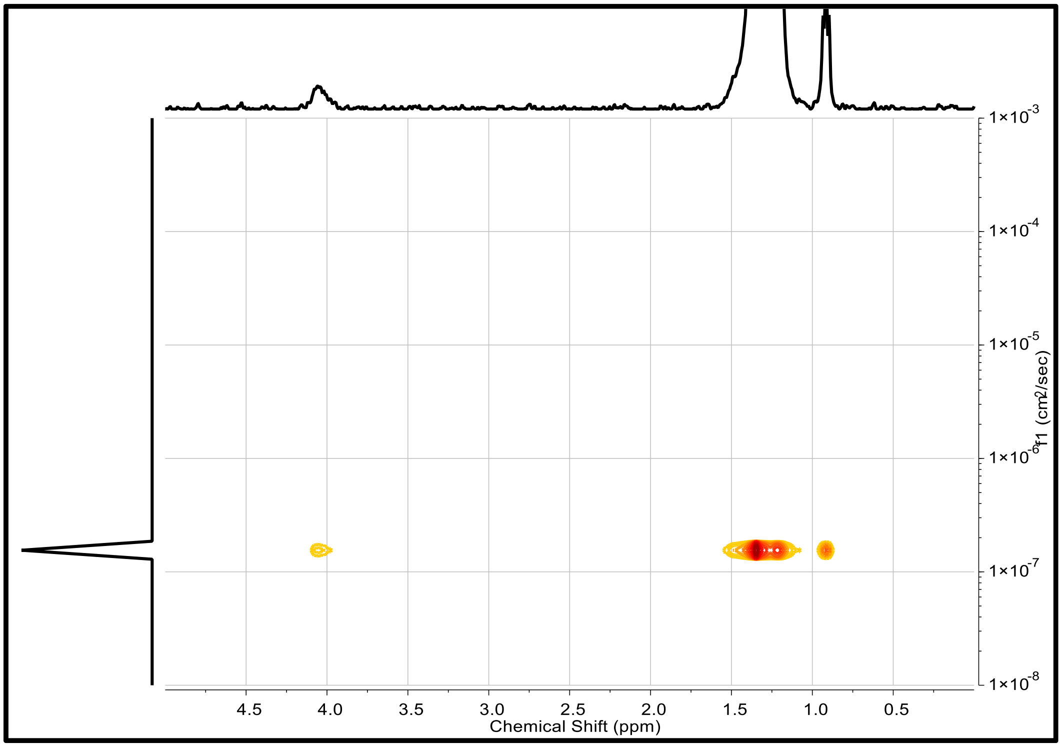

Figure S137. 2D DOSY NMR spectrum (400 MHz, $C_2D_2Cl_4$) of LLDPE–*g*–PNIPAM$_{6.4}$ (T = 105 °C)

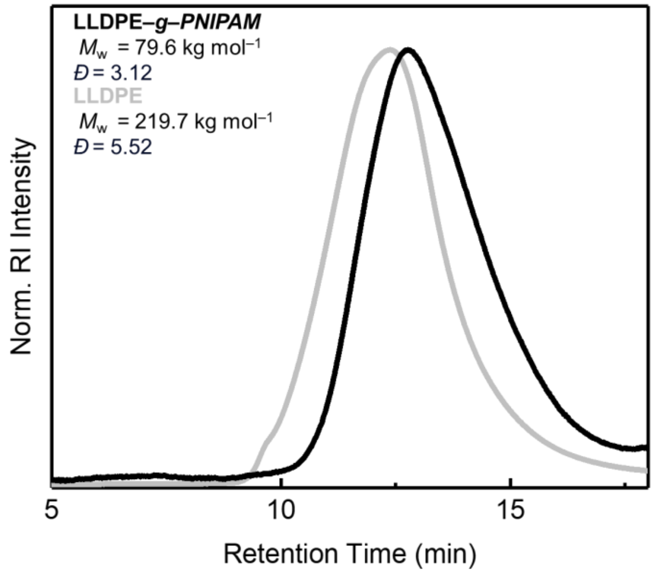

Figure S138. HT SEC trace of LLDPE–*g*–PNIPAM (1,2,4-TCB, 1.0 mL min^{-1} at 150 °C)

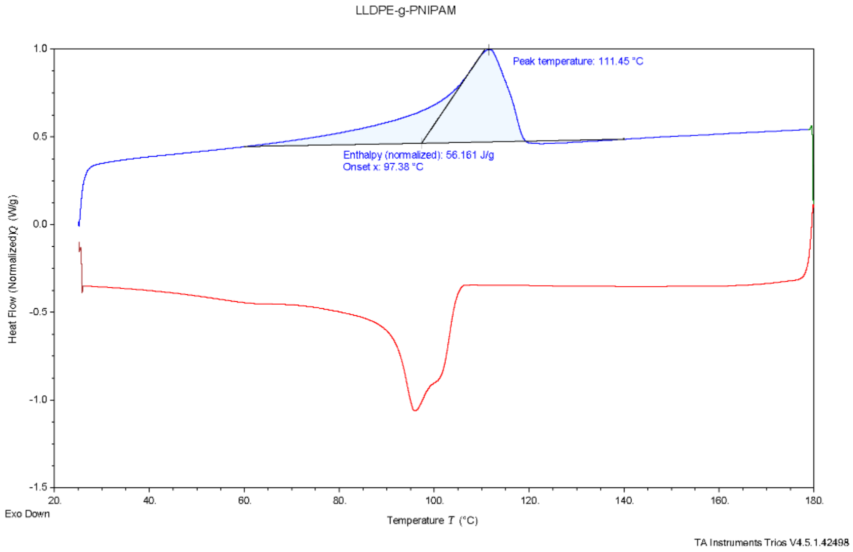

Figure S139. DSC Thermogram of LLDPE–*g*–PNIPAM (f_{vinyl} = 6.4%). T_m = 111.45 °C, ΔH_m = 56.161 J g^{-1}, X_C = 19.2%.

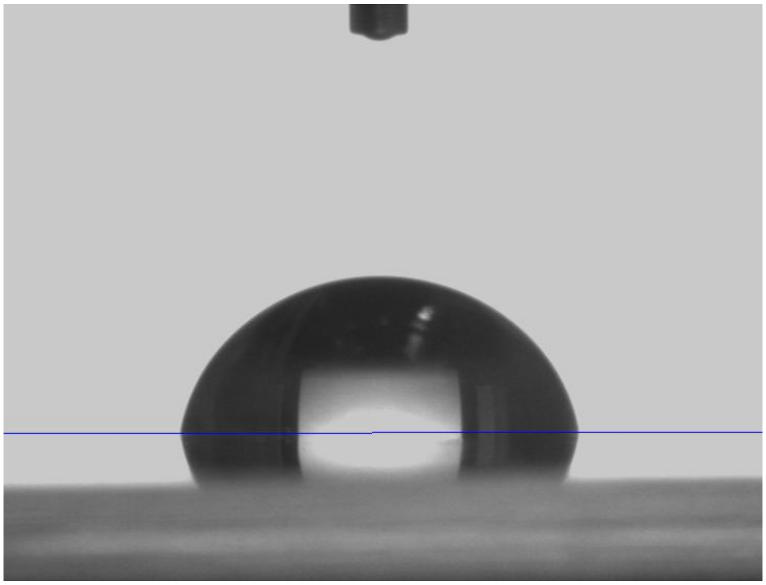

Figure S140. Water contact angle measured for LLDPE–*g*–PNIPAM (f_{vinyl} = 6.4%). WCA = 82.1°

Synthesis of LLDPE–*g*–PS (Figure 5). A 22 mL borosilicate test tube was charged with LLDPE (281 mg, 10 mmol) and 1,2-dichlorobenzene (10 mL),and sealed with a rubber septum. The mixture was degassed by nitrogen bubbling at 105 °C until it became homogeneous. Methyl acrylate (0.3 equiv., 0.34 mL, 3 mmol) was added via syringe. The reaction mixture was stirred and irradiated with a 390 nm LED lamp for 12 hours at 105 °C. Upon completion of the reaction, the mixture was precipitated in cold methanol (ca. 40 mL). The resulting solid was collected by filtration and washed via Soxhlet extraction with acetone for 5 h (ca. 4 min per cycle). After the purification, the solid was dried in a vacuum oven (3 mbar) at 80 °C for 12 h. to afford the desired **LLDPE–*g*–PS** as a white solid (283 mg). The product was characterized by ^{1}H NMR (90 °C in $C_2D_2Cl_4$, 400 MHz), 2D DOSY NMR, DSC, and high temperature size exclusion chromatography. Isolated yield = 48%; Graft yield = 6.3%

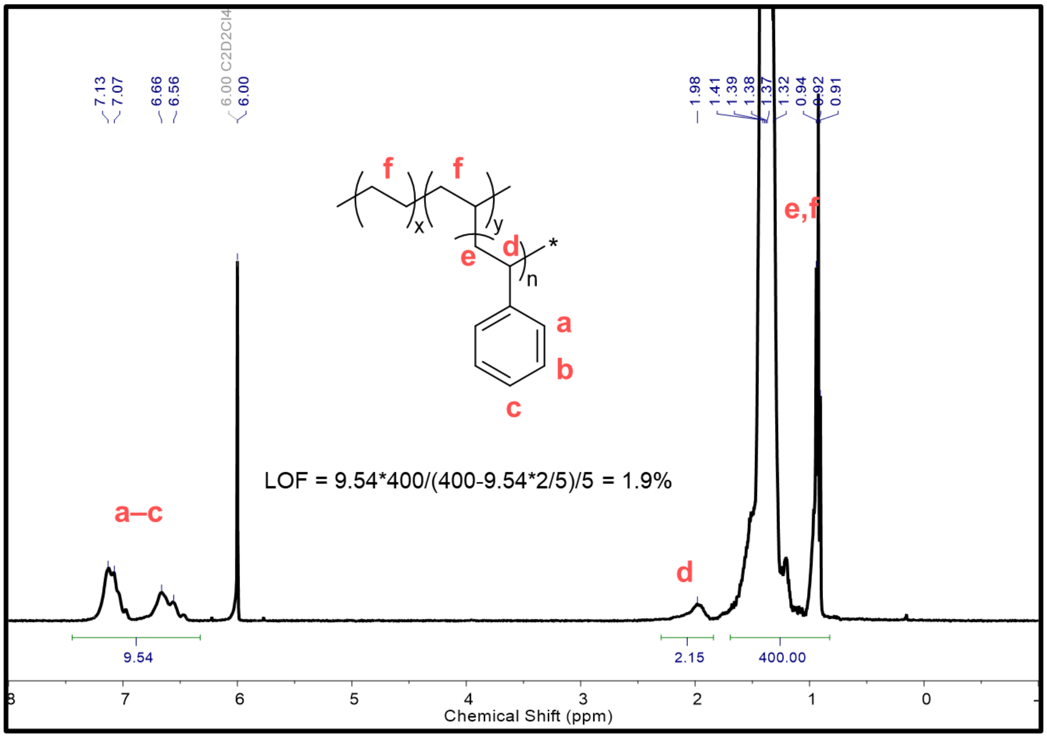

Figure S141. ^{1}H NMR spectrum (400 MHz, $C_2D_2Cl_4$) of LLDPE–*g*–$PS_{1.9}$ (T = 105 °C)

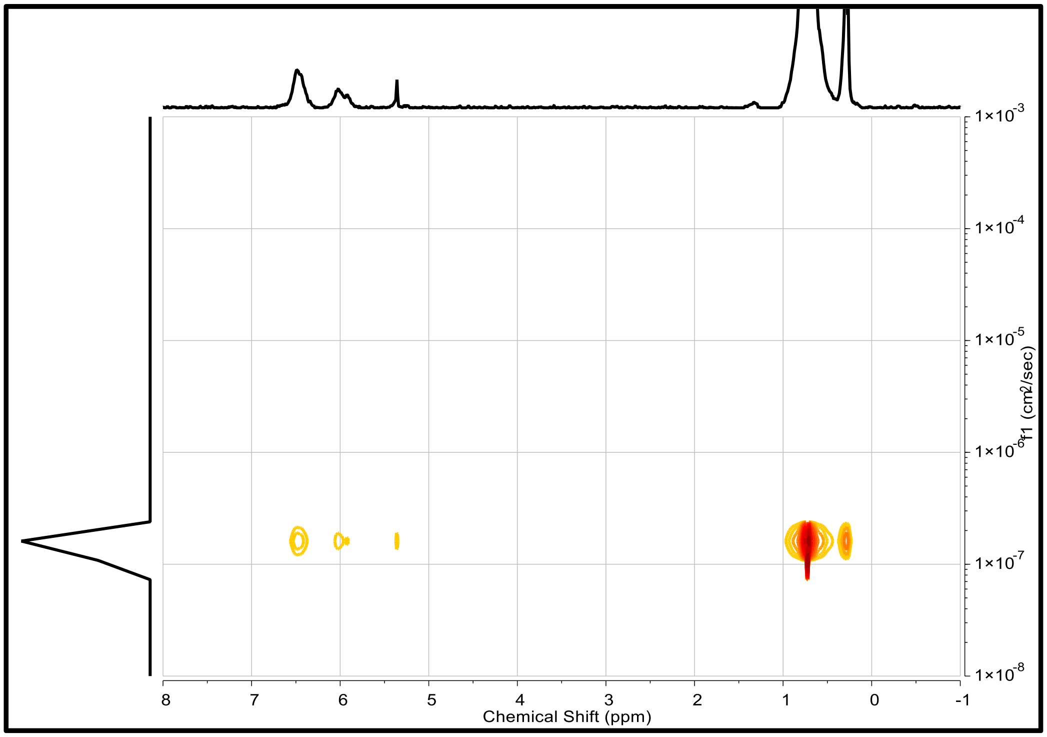

Figure S142. 2D DOSY NMR spectrum (400 MHz, $C_2D_2Cl_4$) of LLDPE–*g*–$PS_{1.9}$ (T = 105 °C)

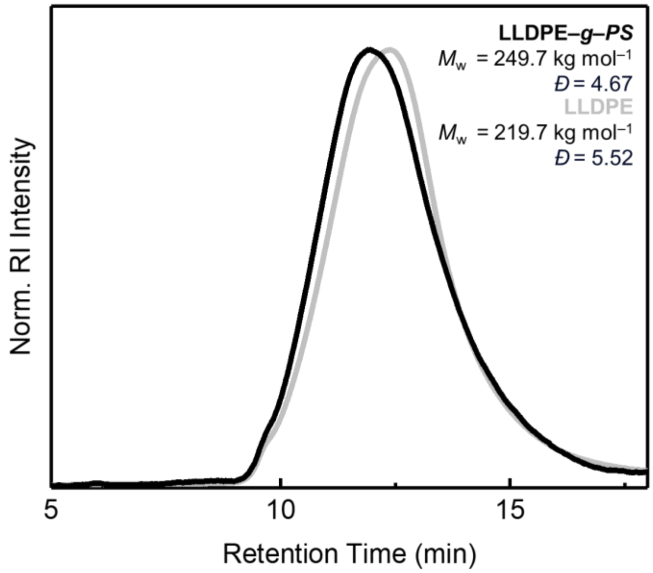

Figure S143. HT SEC trace of LLDPE–*g*–PS (1,2,4-TCB, 1.0 mL min^{-1} at 150 °C)

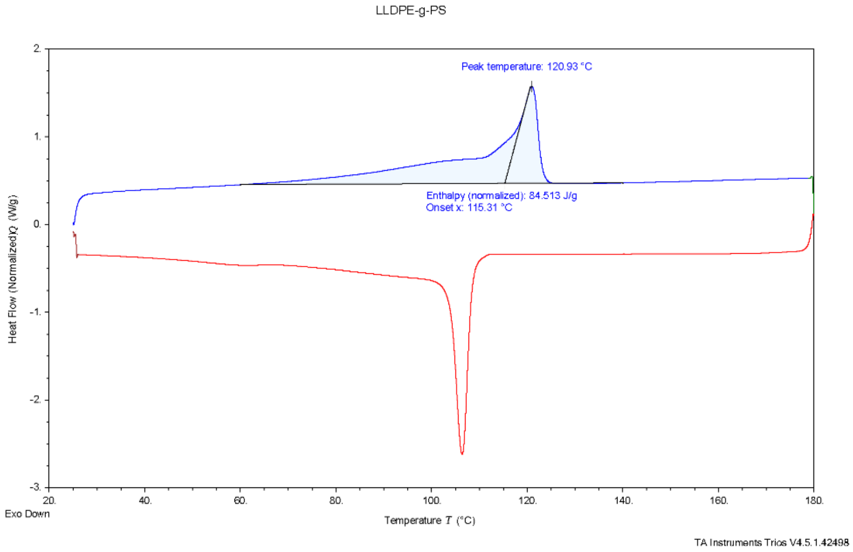

Figure S144. DSC Thermogram of LLDPE–*g*–PS (f_{vinyl} = 1.9%). T_m = 120.93 °C, ΔH_m = 84.513 J g^{-1}, X_C = 28.8%.

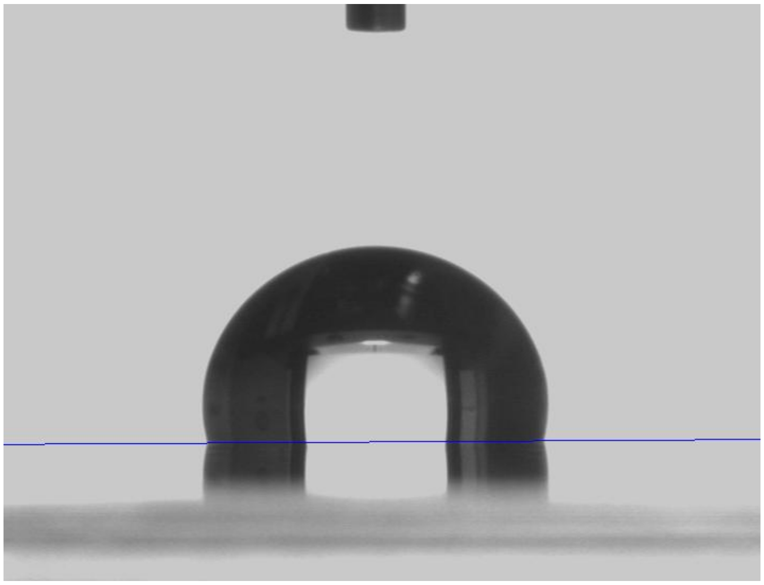

Figure S145. Water contact angle measured for LLDPE–*g*–PS (f_{vinyl} = 1.9%). WCA = 103.0°

Synthesis of HDPE–*g*–PMA (Figure 5). A 22 mL borosilicate test tube was charged with HDPE (281 mg, 10 mmol) and 1,2-dichlorobenzene (10 mL),and sealed with a rubber septum. The mixture was degassed by nitrogen bubbling at 120 °C until it became homogeneous. Methyl acrylate (0.3 equiv., 0.27 mL, 3 mmol) was added via syringe. The reaction mixture was stirred and irradiated with a 390 nm LED lamp for 6 hours at 120 °C. Upon completion of the reaction, the mixture was precipitated in cold methanol (ca. 40 mL). The resulting solid was collected by filtration and washed via Soxhlet extraction with acetone for 8 h (ca. 4 min per cycle). After the purification, the solid was dried in a vacuum oven (3 mbar) at 80 °C for 12 h. to afford the desired **HDPE–*g*–PMA** as a white solid (286 mg). The product was characterized by ^{1}H NMR (90 °C in $C_2D_2Cl_4$, 400 MHz), 2D DOSY NMR, DSC, and high temperature size exclusion chromatography. Isolated yield = 53%; Graft yield = 9.3%

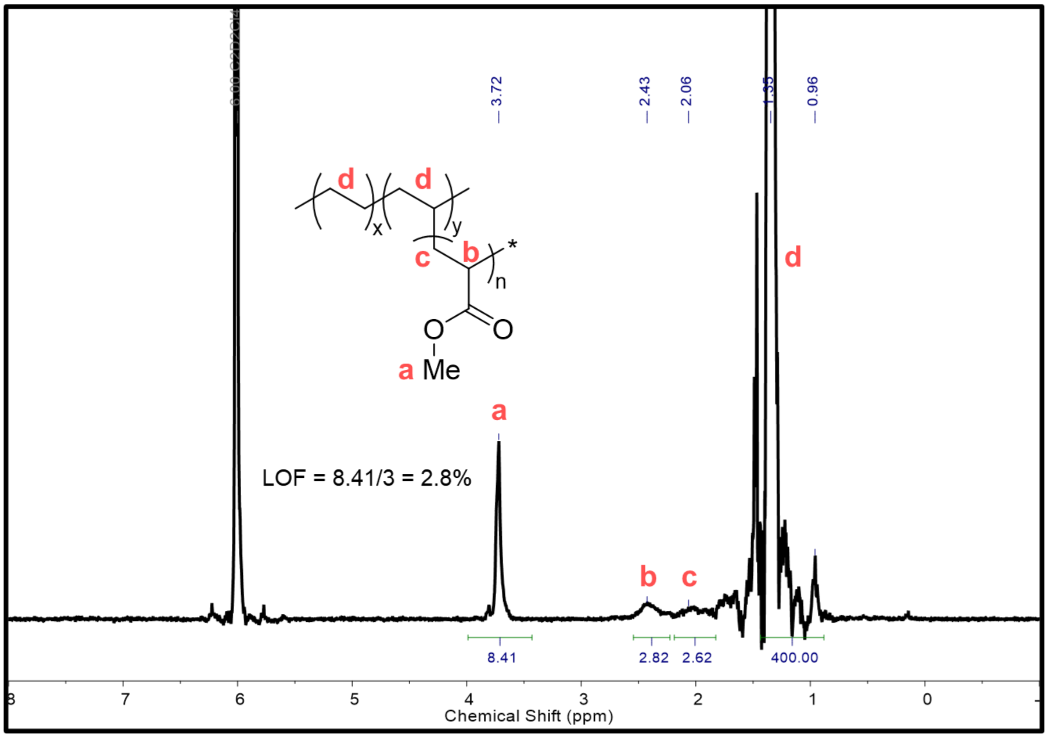

Figure S146 ^{1}H NMR spectrum (400 MHz, $C_2D_2Cl_4$) of HDPE–*g*–$PMA_{2.8}$ (T = 105 °C)

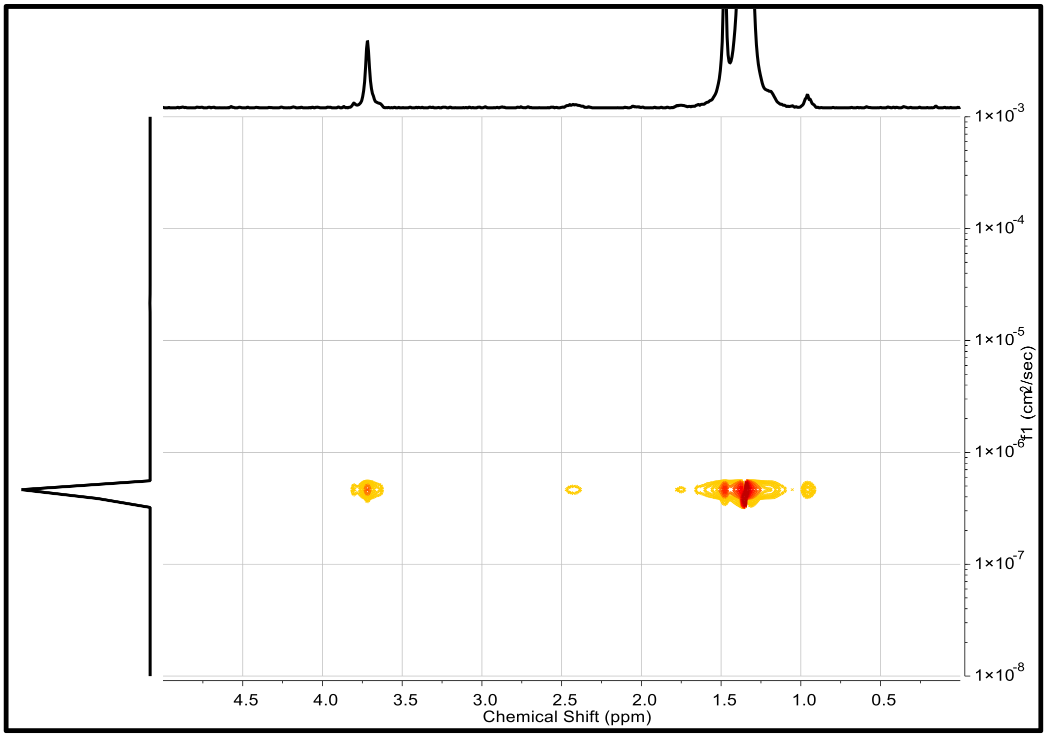

Figure S147. 2D DOSY NMR spectrum (400 MHz, $C_2D_2Cl_4$) of HDPE–*g*–$PMA_{2.8}$ (T = 105 °C)

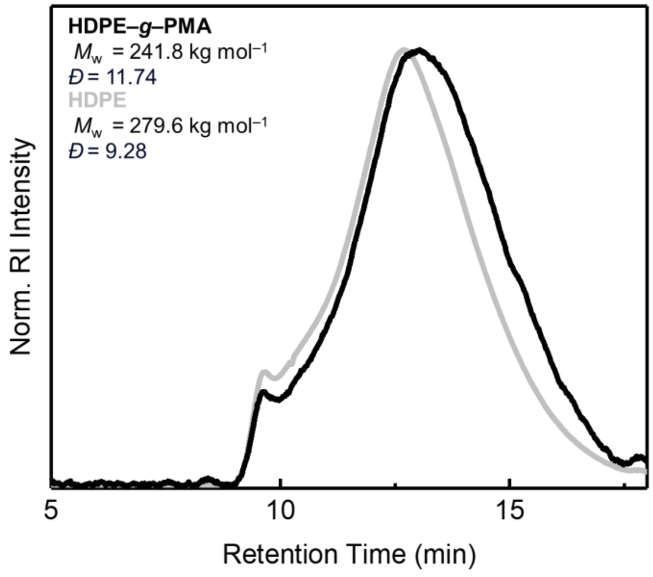

Figure S148. HT SEC trace of HDPE–*g*–PMA (1,2,4-TCB, 1.0 mL min^{-1} at 150 °C)

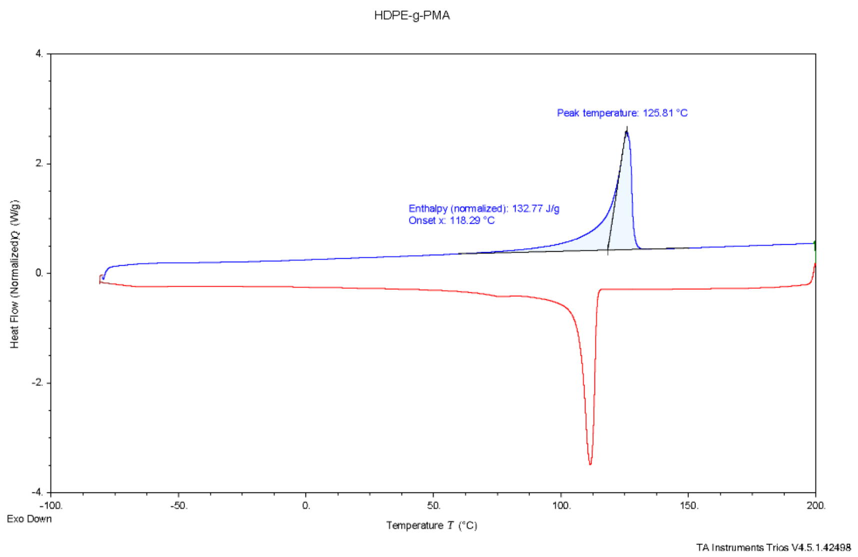

Figure S149. DSC Thermogram of HDPE–*g*–PMA (f_{vinyl} = 2.8%). T_m = 125.81 °C, ΔH_m = 132.77 J g^{-1}, X_C = 45.4%.

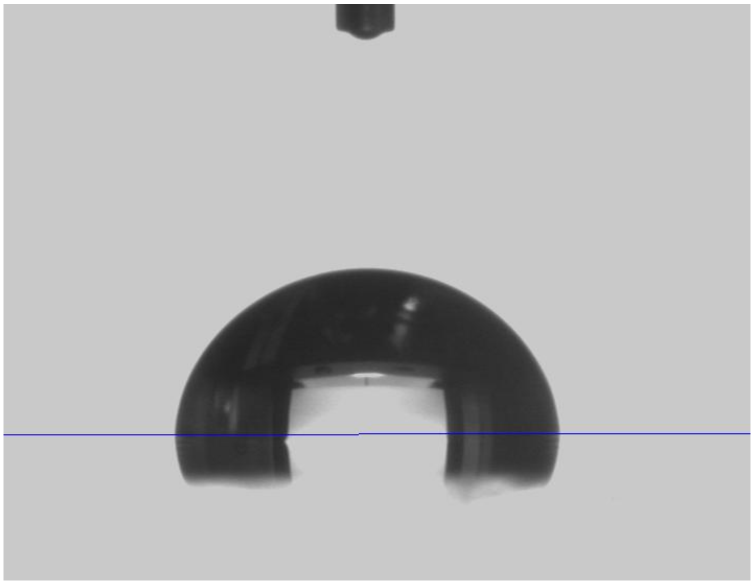

Figure S150. Water contact angle measured for HDPE–*g*–PMA (f_{vinyl} = 2.8%). WCA = 85.9°

Synthesis of HDPE–*g*–PMMA (Figure 5). A 22 mL borosilicate test tube was charged with HDPE (281 mg, 10 mmol) and 1,2-dichlorobenzene (10 mL),and sealed with a rubber septum. The mixture was degassed by nitrogen bubbling at 120 °C until it became homogeneous. Methyl methacrylate (0.4 equiv., 0.43 mL, 4 mmol) was added via syringe. The reaction mixture was stirred and irradiated with a 390 nm LED lamp for 12 hours at 120 °C. Upon completion of the reaction, the mixture was precipitated in cold methanol (ca. 40 mL). The resulting solid was collected by filtration and washed via Soxhlet extraction with acetone for 8 h (ca. 4 min per cycle). After the purification, the solid was dried in a vacuum oven (3 mbar) at 80 °C for 12 h. to afford the desired **HDPE–*g*–PMMA** as a white solid (284 mg). The product was characterized by ^{1}H NMR (90 °C in $C_2D_2Cl_4$, 400 MHz), 2D DOSY NMR, DSC, and high temperature size exclusion chromatography. Isolated yield = 42%; Graft yield = 15%

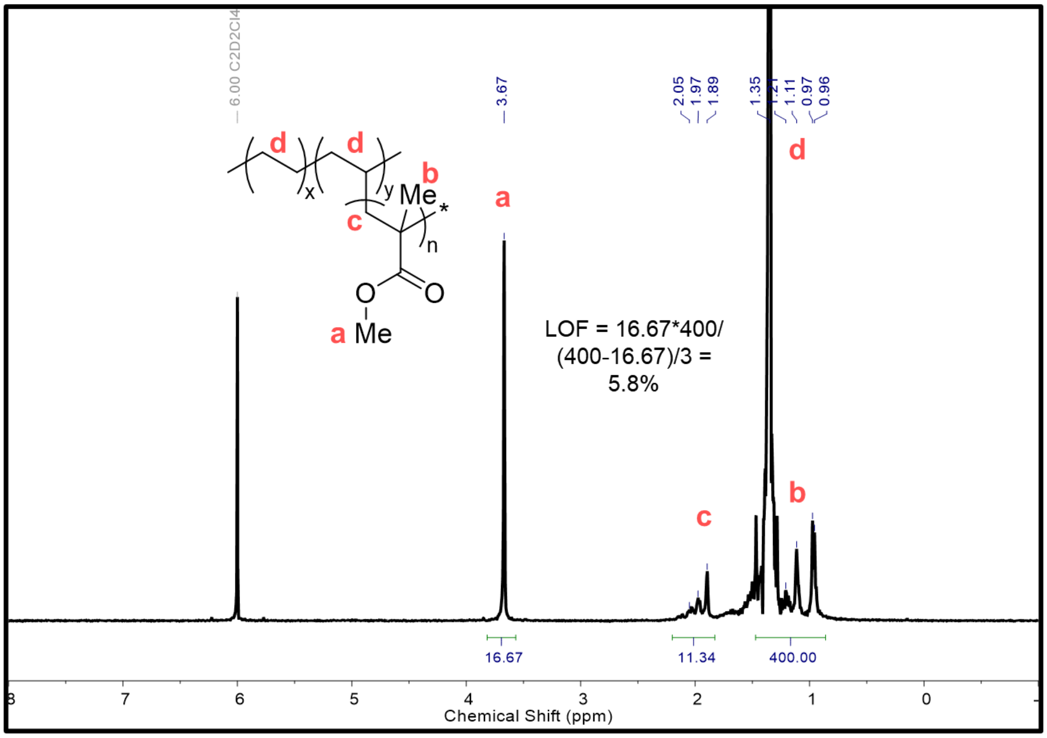

Figure S151. ^{1}H NMR spectrum (400 MHz, $C_2D_2Cl_4$) of HDPE–*g*–$PMMA_{5.8}$ (T = 105 °C)

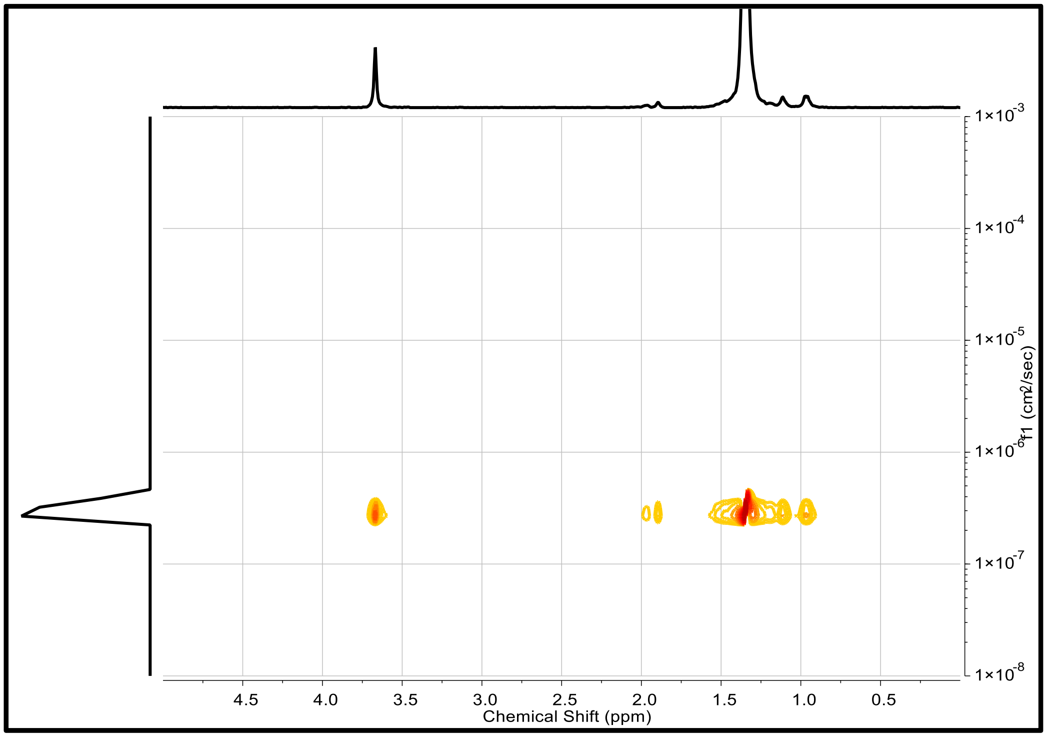

Figure S152. 2D DOSY NMR spectrum (400 MHz, $C_2D_2Cl_4$) of HDPE–*g*–PMMA$_{5.8}$ (T = 105 °C)

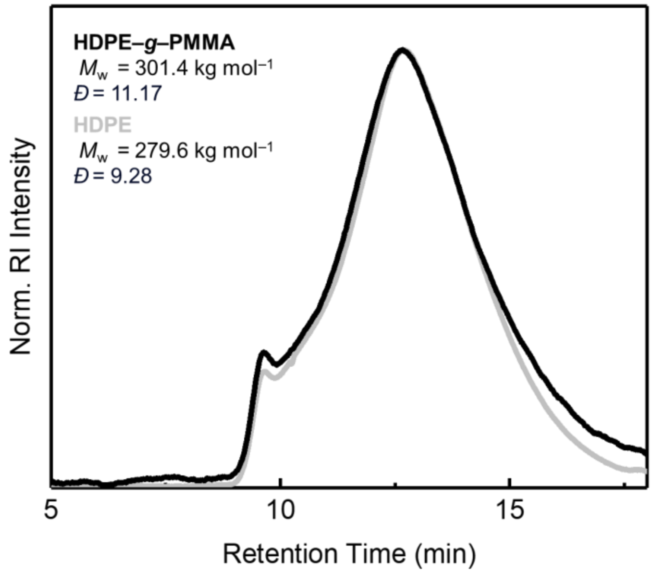

Figure S153. HT SEC trace of HDPE–*g*–PMA (1,2,4-TCB, 1.0 mL min^{-1} at 150 °C)

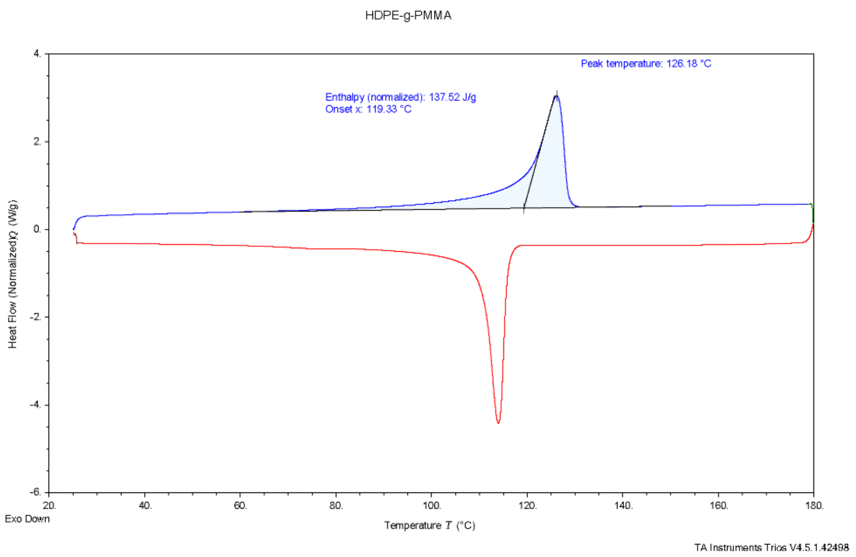

Figure S154. DSC Thermogram of HDPE–*g*–PMMA (f_{vinyl} = 5.8%). T_m = 126.18 °C, ΔH_m = 137.52 J g^{-1}, X_C = 46.9%.

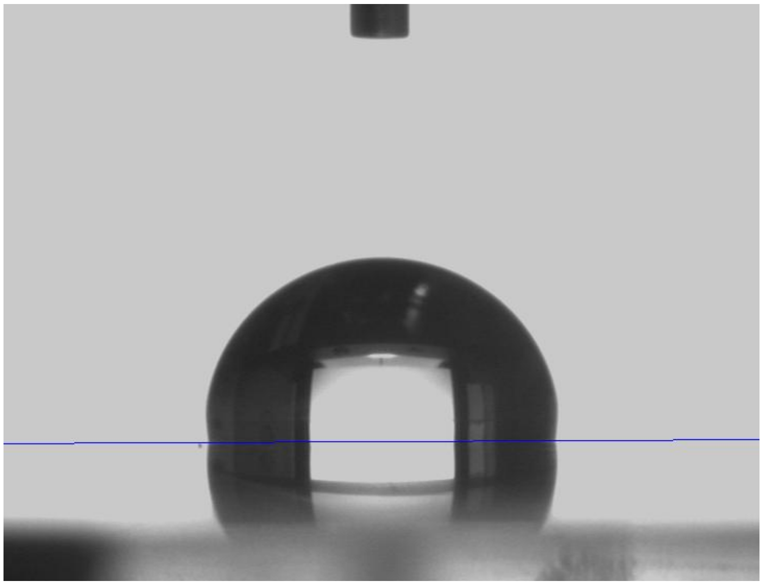

Figure S155. Water contact angle measured for HDPE–*g*–PMMA (f_{vinyl} = 5.8%). WCA = 99.4°

Synthesis of HDPE–*g*–PNIPAM (Figure 5). A 22 mL borosilicate test tube was charged with HDPE (281 mg, 10 mmol) and 1,2-dichlorobenzene (8 mL), and sealed with a rubber septum. The mixture was degassed by nitrogen bubbling at 120 °C until it became homogeneous. *N*-isopropyl acrylamide (0.2 equiv., 226 mg, 2 mmol) in 2 mL of DCB was added via syringe. The reaction mixture was stirred and irradiated with a 390 nm LED lamp for 6 hours at 120 °C. Upon completion of the reaction, the mixture was precipitated in cold methanol (ca. 40 mL). The resulting solid was collected by filtration and washed via Soxhlet extraction with acetone for 16 h (ca. 4 min per cycle). After the purification, the solid was dried in a vacuum oven (3 mbar) at 80 °C for 12 h. to afford the desired **HDPE–*g*–PNIPAM** as a white solid (291 mg). The product was characterized by ^{1}H NMR (90 °C in $C_2D_2Cl_4$, 400 MHz), 2D DOSY NMR, DSC, and high temperature size exclusion chromatography. Isolated yield = 57%; Graft yield = 32%

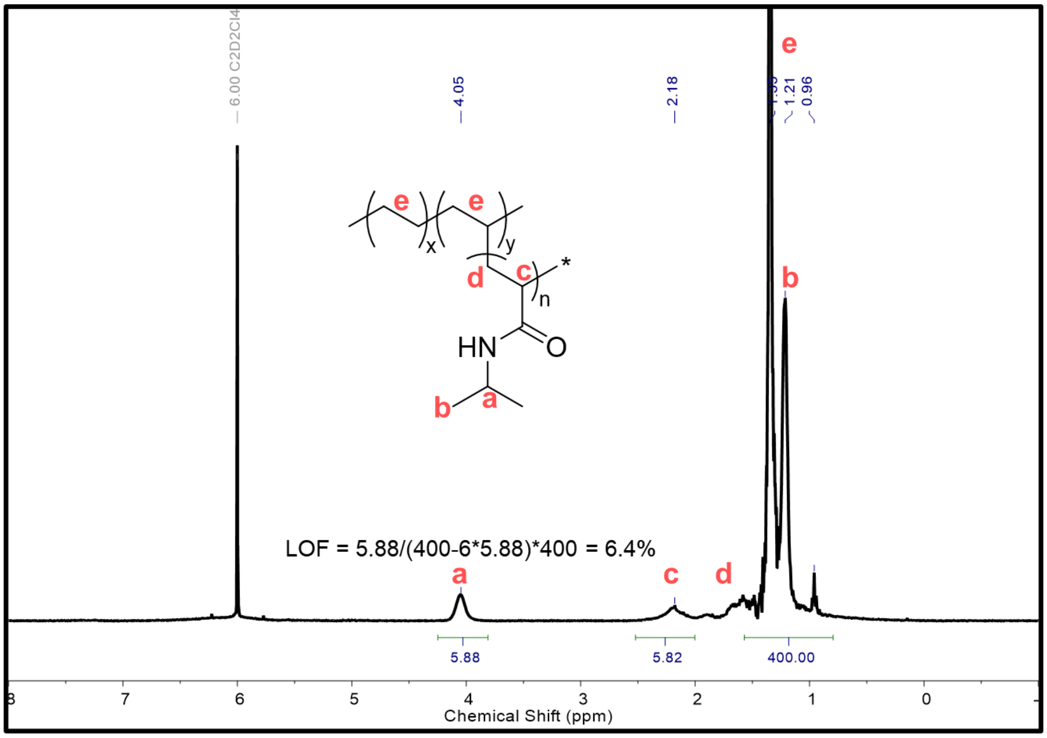

Figure S156. ^{1}H NMR spectrum (400 MHz, $C_2D_2Cl_4$) of HDPE–*g*–PNIPAM$_{6.4}$ (T = 105 °C)

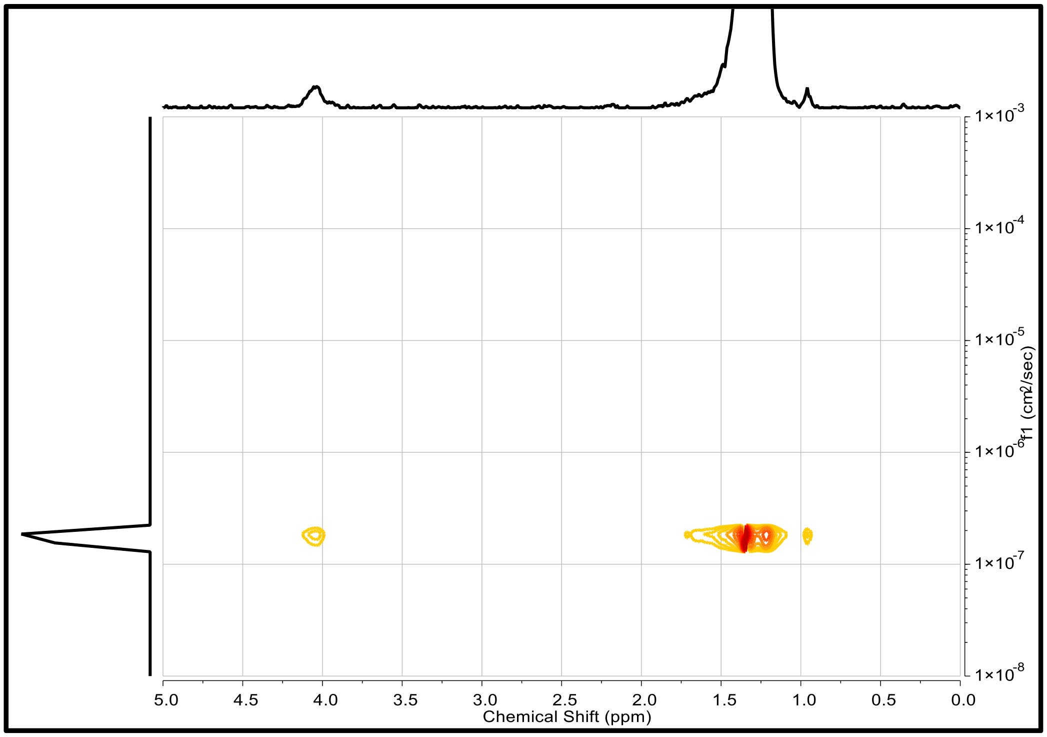

Figure S157. 2D DOSY NMR spectrum (400 MHz, $C_2D_2Cl_4$) of HDPE–*g*–PNIPAM$_{6.4}$ (T = 105 °C)

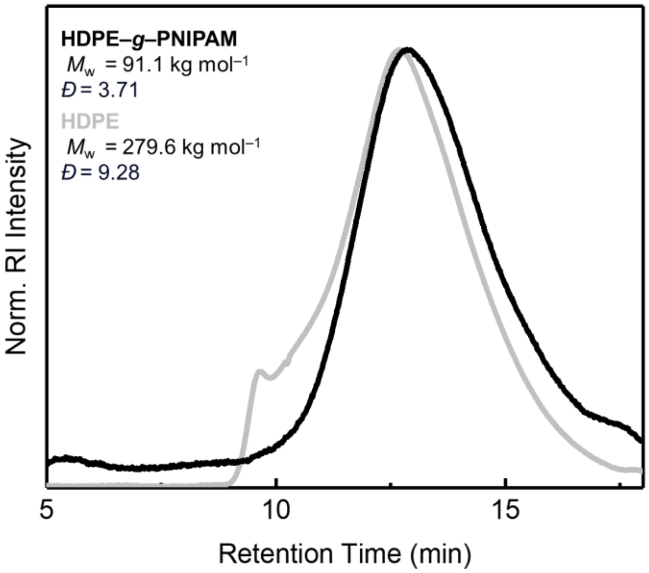

Figure S158. HT SEC trace of HDPE–*g*–PNIPAM (1,2,4-TCB, 1.0 mL min^{-1} at 150 °C)

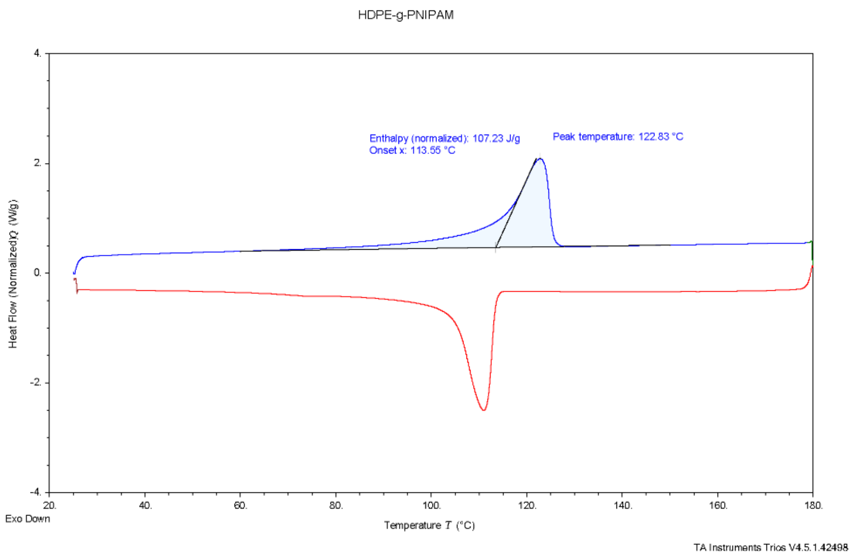

Figure S159. DSC Thermogram of HDPE–*g*–PNIPAM (f_{vinyl} = 6.4%). T_m = 122.83 °C, ΔH_m = 107.23 J g^{-1}, X_C = 36.6%.

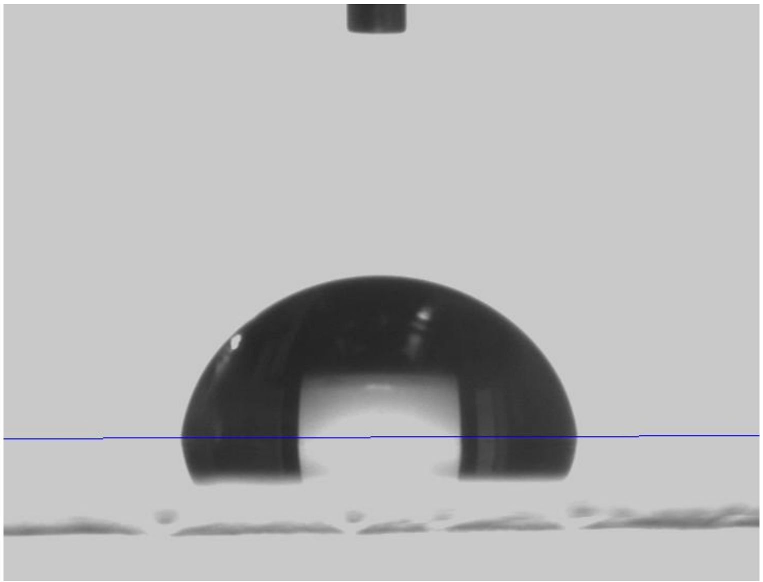

Figure S160. Water contact angle measured for HDPE–*g*–PNIPAM (f_{vinyl} = 6.4%). WCA = 83.2°

Synthesis of HDPE–*g*–PS (Figure 5). A 22 mL borosilicate test tube was charged with HDPE (281 mg, 10 mmol) and 1,2-dichlorobenzene (10 mL),and sealed with a rubber septum. The mixture was degassed by nitrogen bubbling at 120 °C until it became homogeneous. Styrene (0.3 equiv., 0.34 mL, 3 mmol) was added via syringe. The reaction mixture was stirred and irradiated with a 390 nm LED lamp for 12 hours at 120 °C. Upon completion of the reaction, the mixture was precipitated in cold methanol (ca. 40 mL). The resulting solid was collected by filtration and washed via Soxhlet extraction with acetone for 7 h (ca. 4 min per cycle). After the purification, the solid was dried in a vacuum oven (3 mbar) at 80 °C for 12 h. to afford the desired **HDPE–*g*–PS** as a white solid (285 mg). The product was characterized by ^{1}H NMR (90 °C in $C_2D_2Cl_4$, 400 MHz), 2D DOSY NMR, DSC, and high temperature size exclusion chromatography.
Isolated yield = 48%; Graft yield = 14%

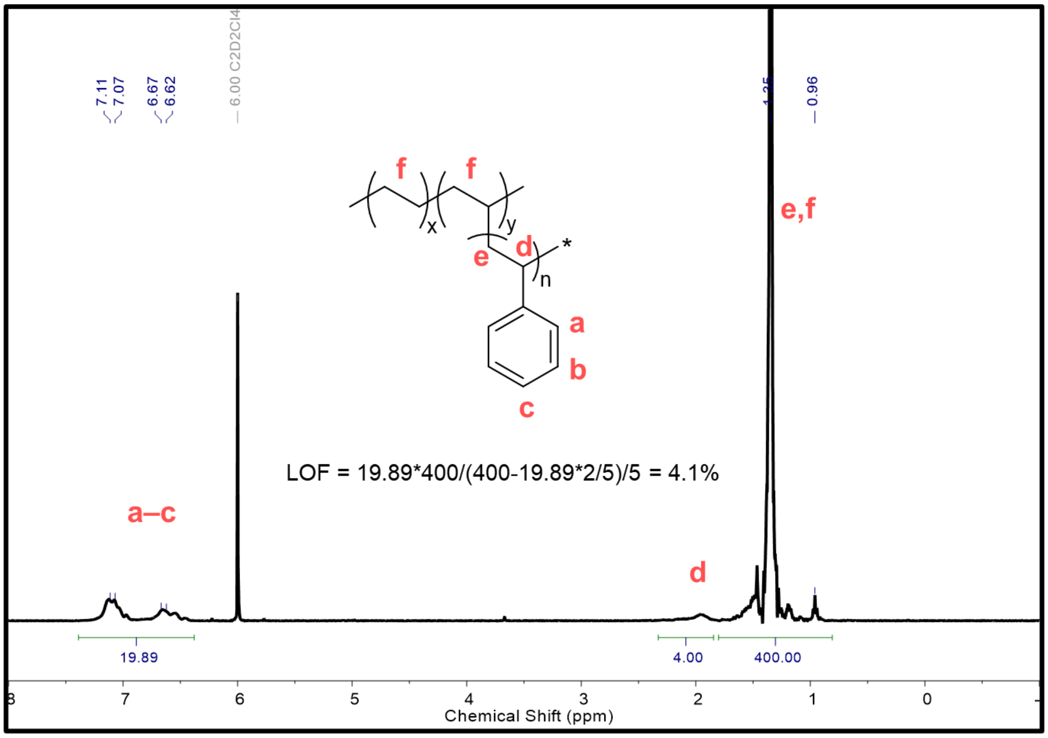

Figure S161. ^{1}H NMR spectrum (400 MHz, $C_2D_2Cl_4$) of HDPE–*g*–$PS_{4.1}$ (T = 105 °C)

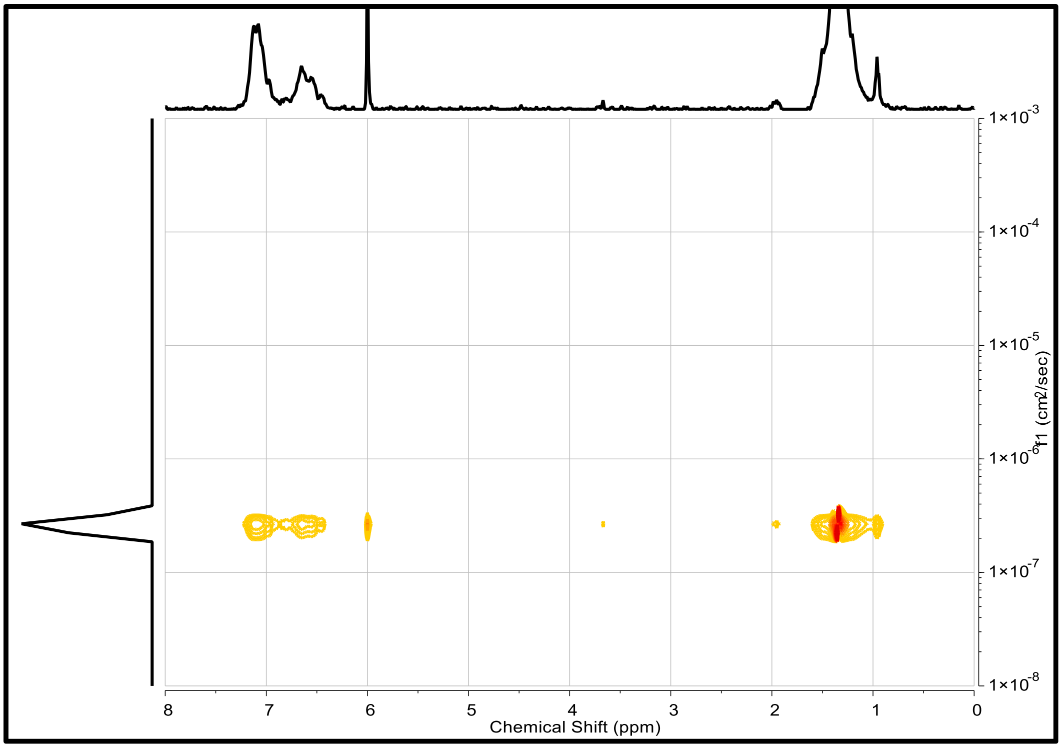

Figure S162. 2D DOSY NMR spectrum (400 MHz, $C_2D_2Cl_4$) of HDPE–*g*–PS$_{4.1}$ (T = 105 °C)

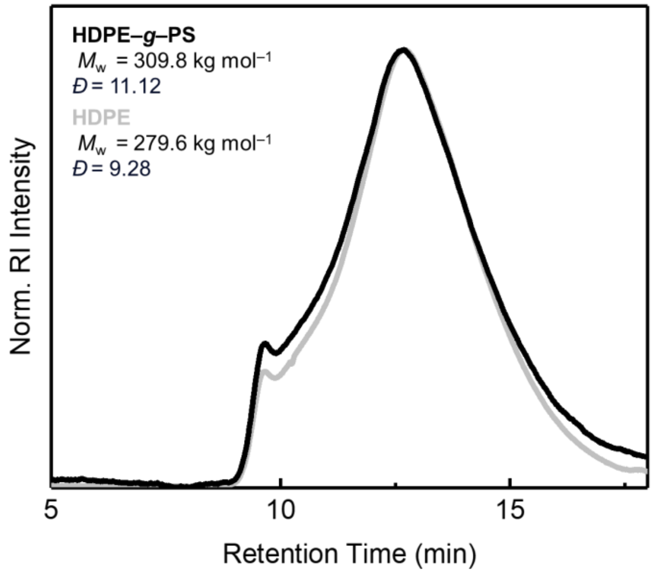

Figure S163. HT SEC trace of HDPE–*g*–PS (1,2,4-TCB, 1.0 mL min^{-1} at 150 °C)

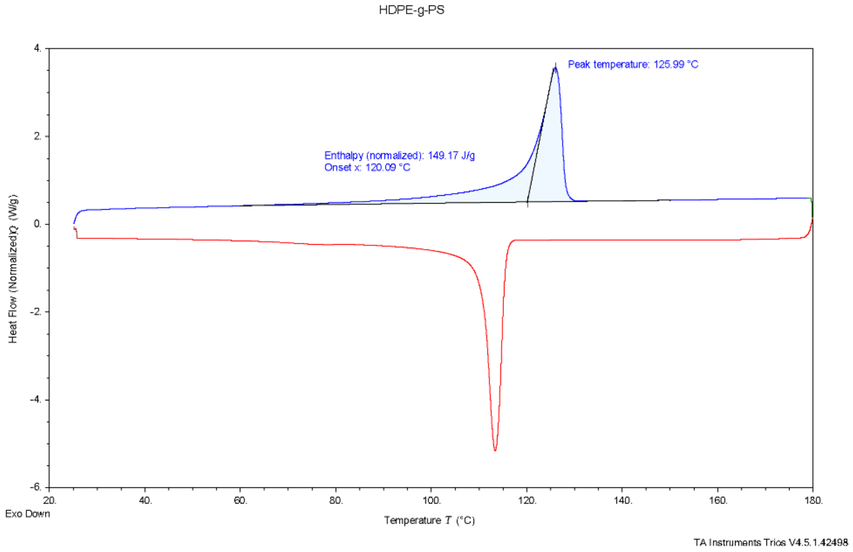

Figure S164. DSC Thermogram of HDPE–*g*–PS (f_{vinyl} = 4.1%). T_m = 125.99 °C, ΔH_m = 149.17 J g^{-1}, X_C = 50.9%.

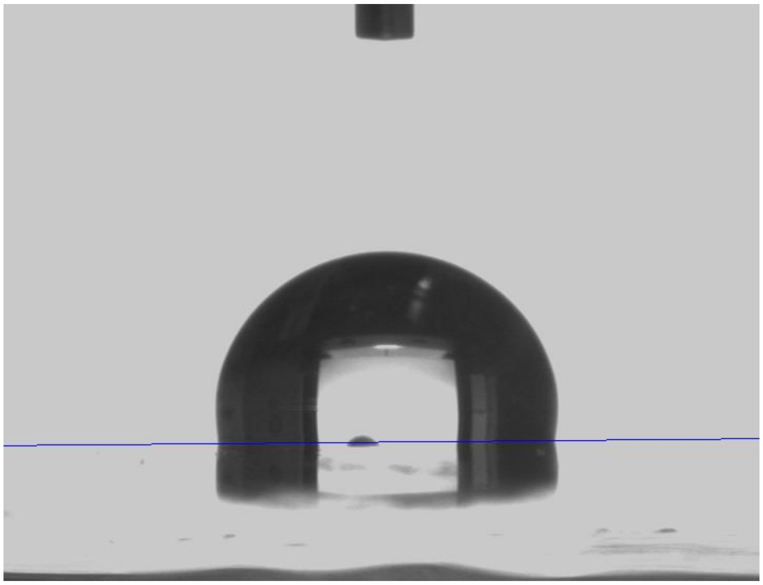

Figure S165. Water contact angle measured for HDPE–*g*–PS (f_{vinyl} = 4.1%). WCA = 97.8°

Synthesis of *i*PP–*g*–PMA(Figure 5). A 22 mL borosilicate test tube was charged with *i*PP (281 mg, 6.7 mmol) and 1,2-dichlorobenzene (10 mL),and sealed with a rubber septum. The mixture was degassed by nitrogen bubbling at 120 °C until it became homogeneous. Methyl acrylate (0.45 equiv., 0.27 mL, 3 mmol) was added via syringe. The reaction mixture was stirred and irradiated with a 390 nm LED lamp for 6 hours at 120 °C. Upon completion of the reaction, the mixture was precipitated in cold methanol (ca. 40 mL). The resulting solid was collected by filtration and washed via Soxhlet extraction with acetone for 6 h (ca. 4 min per cycle). After the purification, the solid was dried in a vacuum oven (3 mbar) at 80 °C for 12 h. to afford the desired ***i*PP–*g*–PMA** as a white solid (273 mg). The product was characterized by ^{1}H NMR (90 °C in $C_2D_2Cl_4$, 400 MHz), 2D DOSY NMR, DSC, and high temperature size exclusion chromatography. Isolated yield = 51%; Graft yield = 7.0%

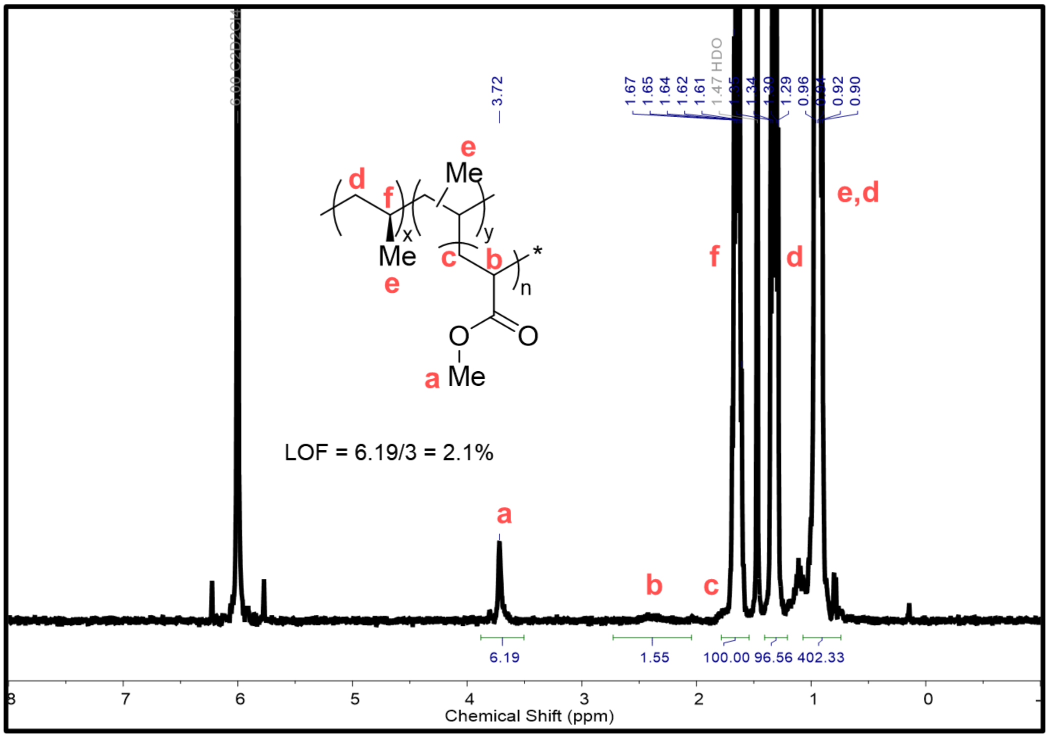

Figure S166. ^{1}H NMR spectrum (400 MHz, $C_2D_2Cl_4$) of *i*PP–*g*–$PMA_{2.1}$ (T = 105 °C)

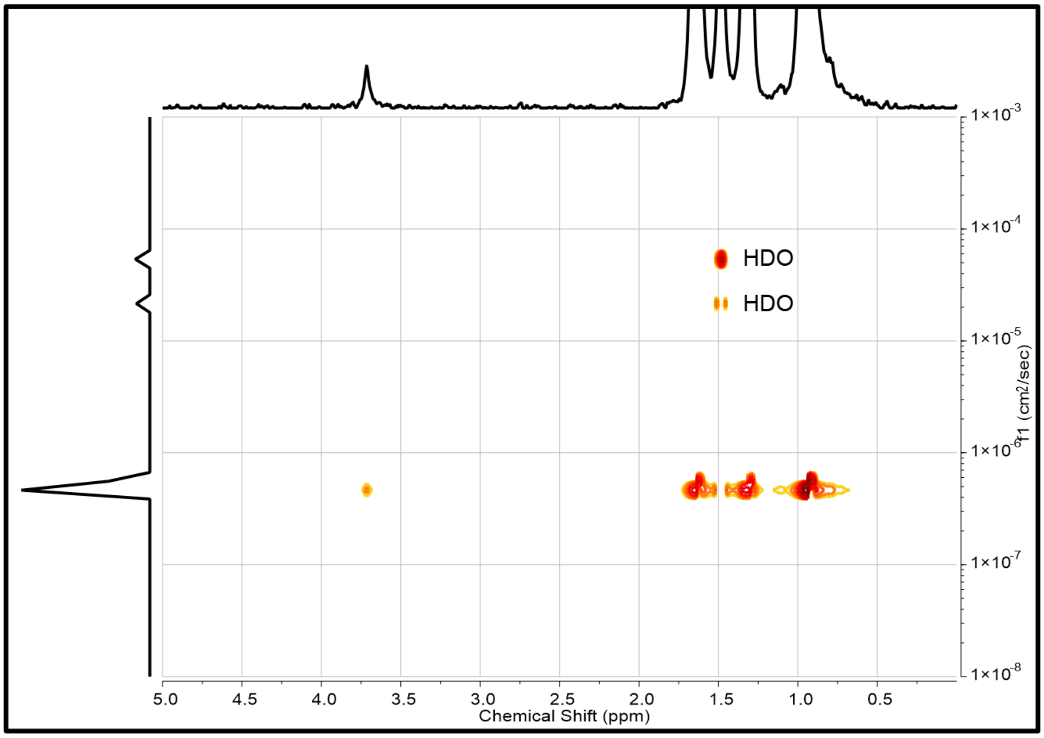

Figure S167. 2D DOSY NMR spectrum (400 MHz, $C_2D_2Cl_4$) of *i*PP–*g*–PMA$_{2.1}$ (T = 105 °C)

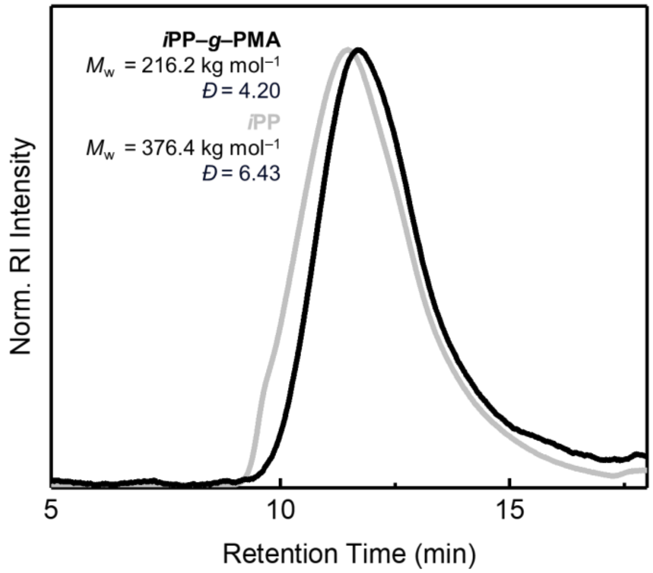

Figure S168. HT SEC trace of *i*PP–*g*–PMA (1,2,4-TCB, 1.0 mL min^{-1} at 150 °C)

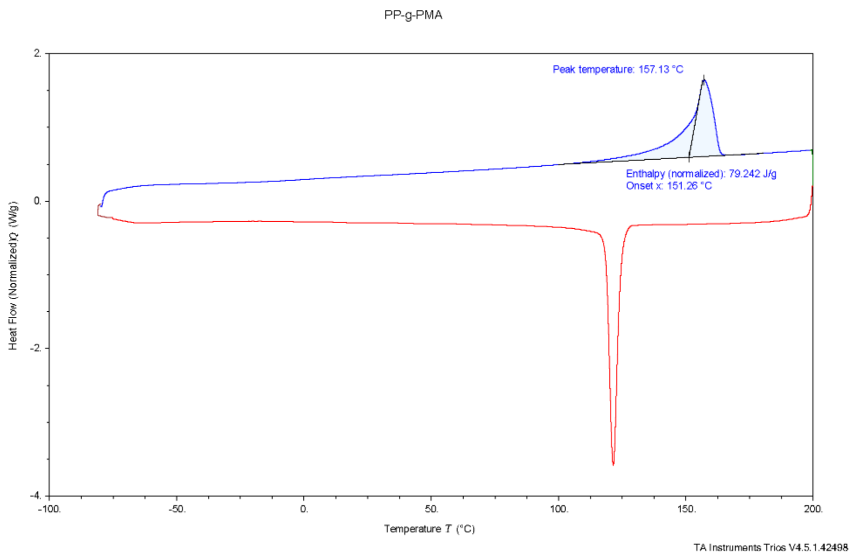

Figure S169. DSC Thermogram of *i*PP–*g*–PMA (f_{vinyl} = 2.1%). T_m = 157.13 °C, ΔH_m = 79.242 J g^{-1}, X_C = 38.3%.

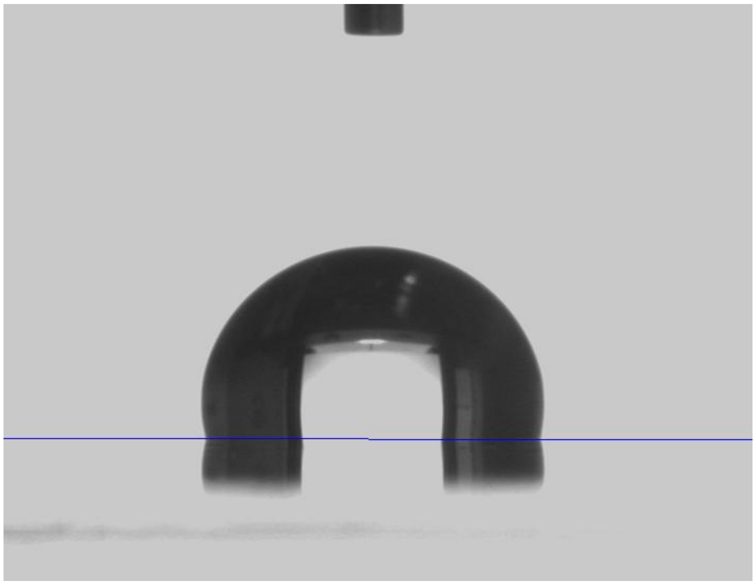

Figure S170. Water contact angle measured for *i*PP–*g*–PMA (f_{vinyl} = 2.1%). WCA = 101.9°

Synthesis of *i*PP–*g*–PMMA(Figure 5). A 22 mL borosilicate test tube was charged with *i*PP (281 mg, 6.7 mmol) and 1,2-dichlorobenzene (10 mL),and sealed with a rubber septum. The mixture was degassed by nitrogen bubbling at 120 °C until it became homogeneous. Methyl methacrylate (0.4 equiv., 0.43 mL, 4 mmol) was added via syringe. The reaction mixture was stirred and irradiated with a 390 nm LED lamp for 12 hours at 120 °C. Upon completion of the reaction, the mixture was precipitated in cold methanol (ca. 40 mL). The resulting solid was collected by filtration and washed via Soxhlet extraction with acetone for 8 h (ca. 4 min per cycle). After the purification, the solid was dried in a vacuum oven (3 mbar) at 80 °C for 12 h. to afford the desired ***i*PP–*g*–PMMA** as a white solid (273 mg). The product was characterized by ^{1}H NMR (90 °C in $C_2D_2Cl_4$, 400 MHz), 2D DOSY NMR, DSC, and high temperature size exclusion chromatography. Isolated yield = 40%; Graft yield = 12%

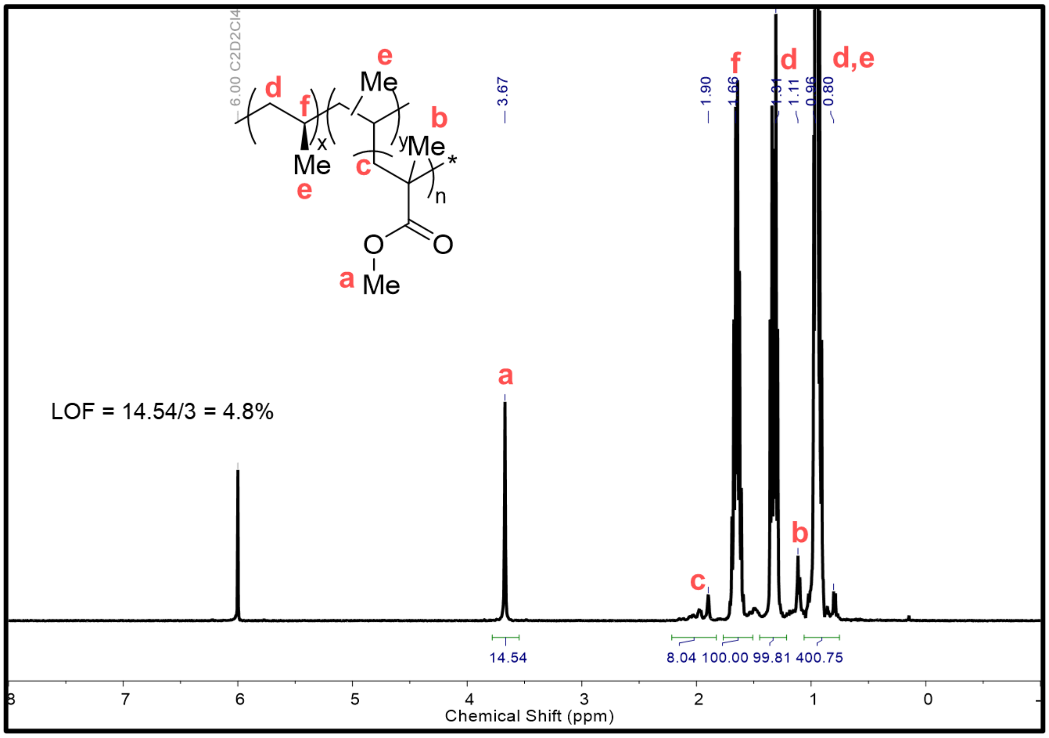

Figure S171. ^{1}H NMR spectrum (400 MHz, $C_2D_2Cl_4$) of *i*PP–*g*–$PMMA_{4.8}$ (T = 105 °C)

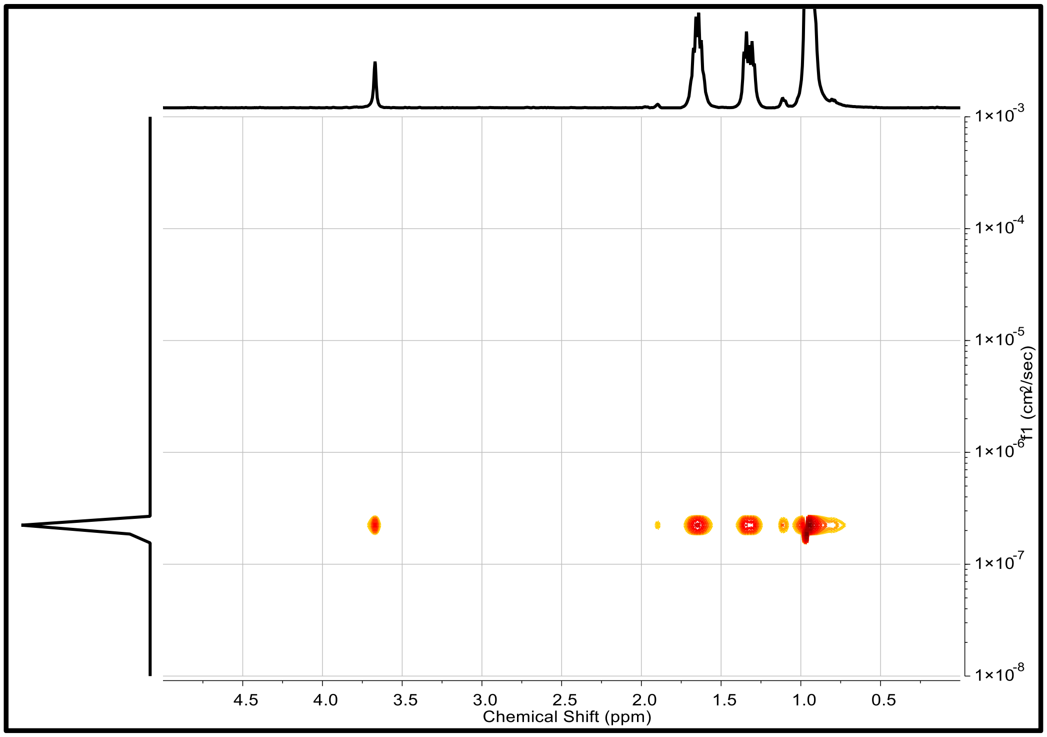

Figure S172. 2D DOSY NMR spectrum (400 MHz, $C_2D_2Cl_4$) of *i*PP–*g*–PMMA$_{4.8}$ (T = 105 °C).

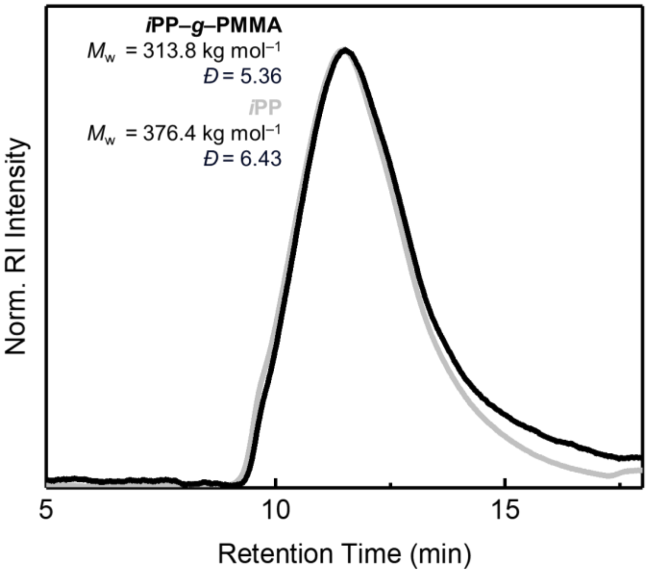

Figure S173. HT SEC trace of *i*PP–*g*–PMMA (1,2,4-TCB, 1.0 mL min^{-1} at 150 °C)

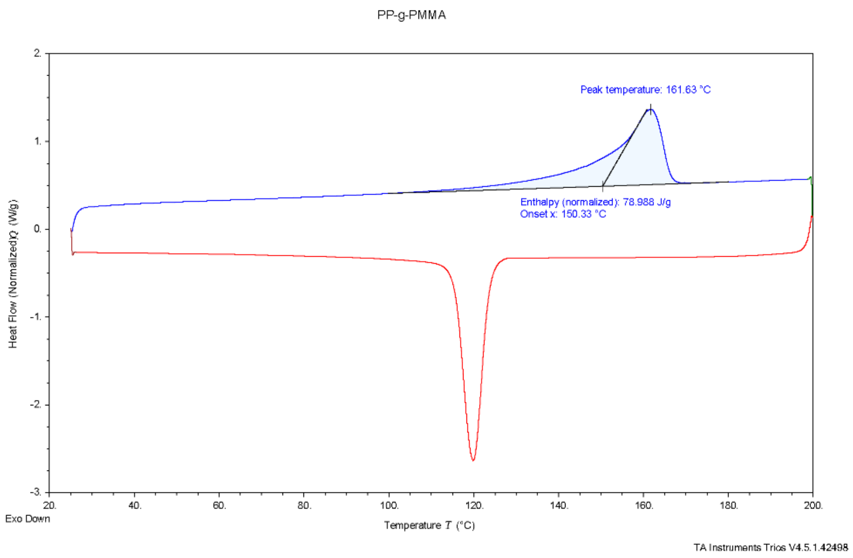

Figure S174. DSC Thermogram of *i*PP–*g*–PMMA (f_{vinyl} = 4.8%). T_m = 161.63 °C, ΔH_m = 78.988 J g^{-1}, X_C = 38.2%.

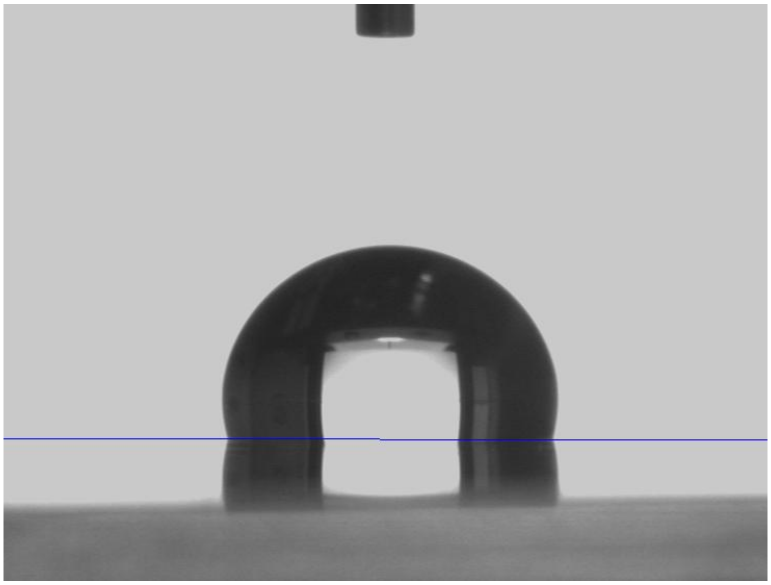

Figure S175. Water contact angle measured for *i*PP–*g*–PMMA (f_{vinyl} = 4.8%). WCA = 104.3°

Synthesis of *i*PP–*g*–PNIPAM (Figure 5). A 22 mL borosilicate test tube was charged with *i*PP (281 mg, 6.7 mmol) and 1,2-dichlorobenzene (8 mL),and sealed with a rubber septum. The mixture was degassed by nitrogen bubbling at 120 °C until it became homogeneous. *N*-isopropyl acrylamide (0.2 equiv., 0.226 mg, 2 mmol) in 2 mL of DCB was added via syringe. The reaction mixture was stirred and irradiated with a 390 nm LED lamp for 6 hours at 120 °C. Upon completion of the reaction, the mixture was precipitated in cold methanol (ca. 40 mL). The resulting solid was collected by filtration and washed via Soxhlet extraction with acetone for 16 h (ca. 4 min per cycle). After the purification, the solid was dried in a vacuum oven (3 mbar) at 80 °C for 12 h. to afford the desired ***i*PP–*g*–PNIPAM** as a white solid (279 mg). The product was characterized by ^1H NMR (90 °C in $C_2D_2Cl_4$, 400 MHz), 2D DOSY NMR, DSC, and high temperature size exclusion chromatography. Isolated yield = 55%; Graft yield = 27%

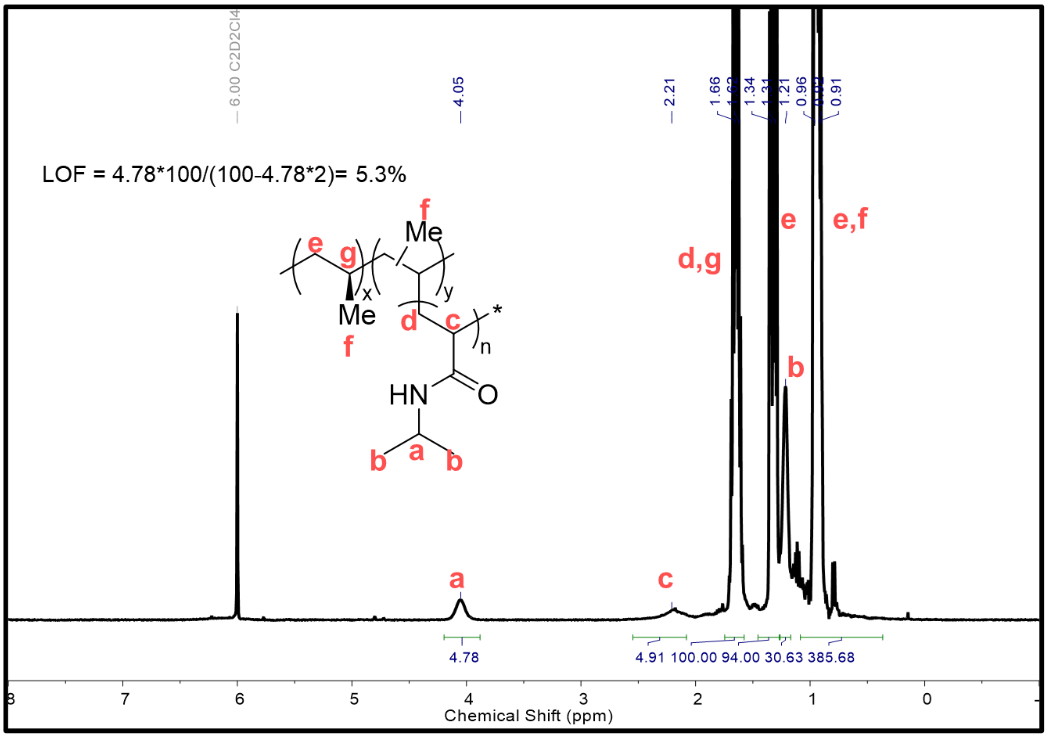

Figure S176. ^1H NMR spectrum (400 MHz, $C_2D_2Cl_4$) of *i*PP–*g*–PNIPAM$_{5.3}$ (T = 105 °C)

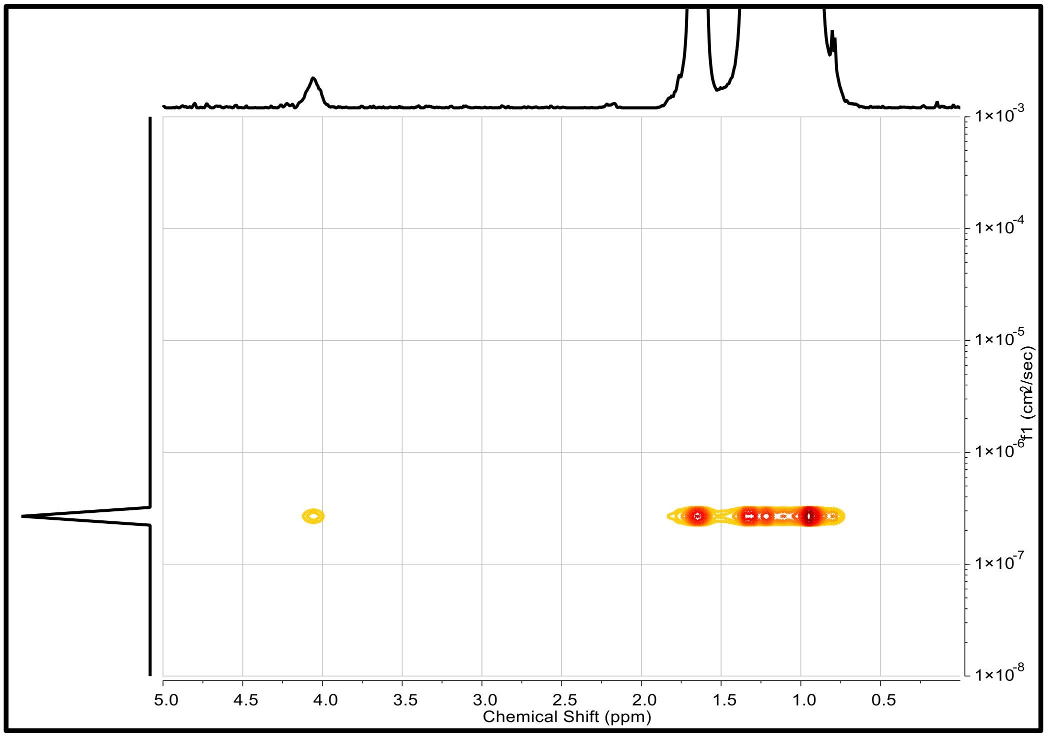

Figure S177. 2D DOSY NMR spectrum (400 MHz, $C_2D_2Cl_4$) of PP–*g*–PNIPAM$_{5.3}$ (T = 105 °C)

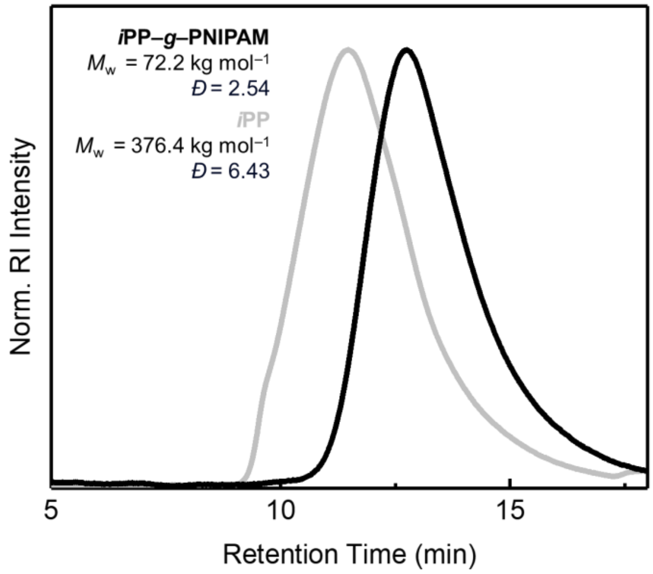

Figure S178. HT SEC trace of *i*PP–*g*–PNIPAM$_{5.3}$ (1,2,4-TCB, 1.0 mL min^{-1} at 150 °C)

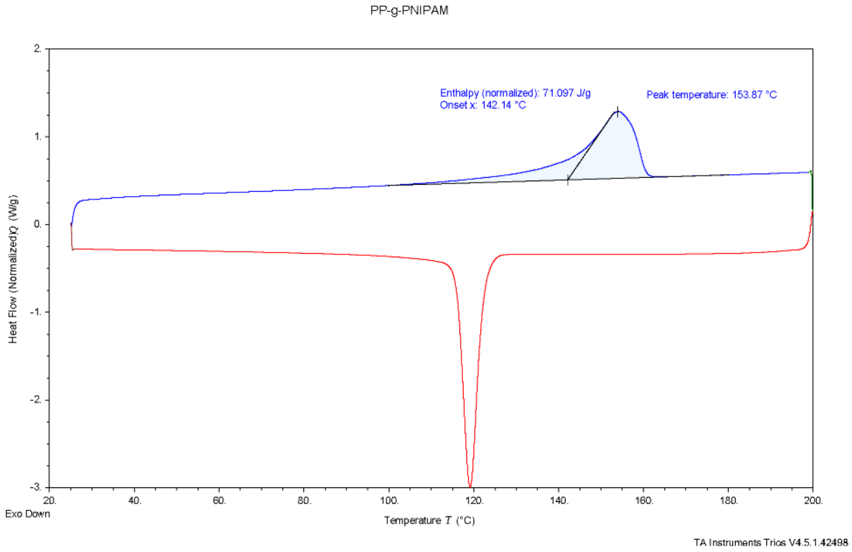

Figure S179. DSC Thermogram of *i*PP–*g*–PNIPAM (f_{vinyl} = 5.3%). T_m = 153.87 °C, ΔH_m = 71.097 J g^{-1}, X_C = 34.3%.

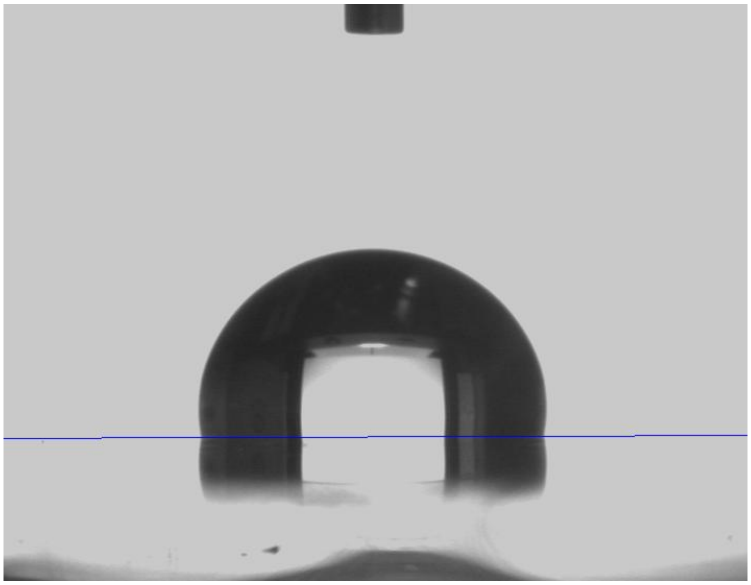

Figure S180. Water contact angle measured for *i*PP–*g*–PNIPAM (f_{vinyl} = 5.3%). WCA = 101.5°

Synthesis of *i*PP–*g*–PS (Fig. 5). A 22 mL borosilicate test tube was charged with *i*PP (281 mg, 6.7 mmol) and 1,2-dichlorobenzene (10 mL),and sealed with a rubber septum. The mixture was degassed by nitrogen bubbling at 120 °C until it became homogeneous. Styrene (0.3 equiv., 0.27 mL, 3 mmol) was added via syringe. The reaction mixture was stirred and irradiated with a 390 nm LED lamp for 6 hours at 120 °C. Upon completion of the reaction, the mixture was precipitated in cold methanol (ca. 40 mL). The resulting solid was collected by filtration and washed via Soxhlet extraction with acetone for 8 h (ca. 4 min per cycle). After the purification, the solid was dried in a vacuum oven (3 mbar) at 80 °C for 12 h. to afford the desired ***i*PP–*g*–PS** as a white solid (265 mg). The product was characterized by ^{1}H NMR (90 °C in $C_2D_2Cl_4$, 400 MHz), 2D DOSY NMR, DSC, and high temperature size exclusion chromatography. Isolated yield = 45%; Graft yield = 9.3%

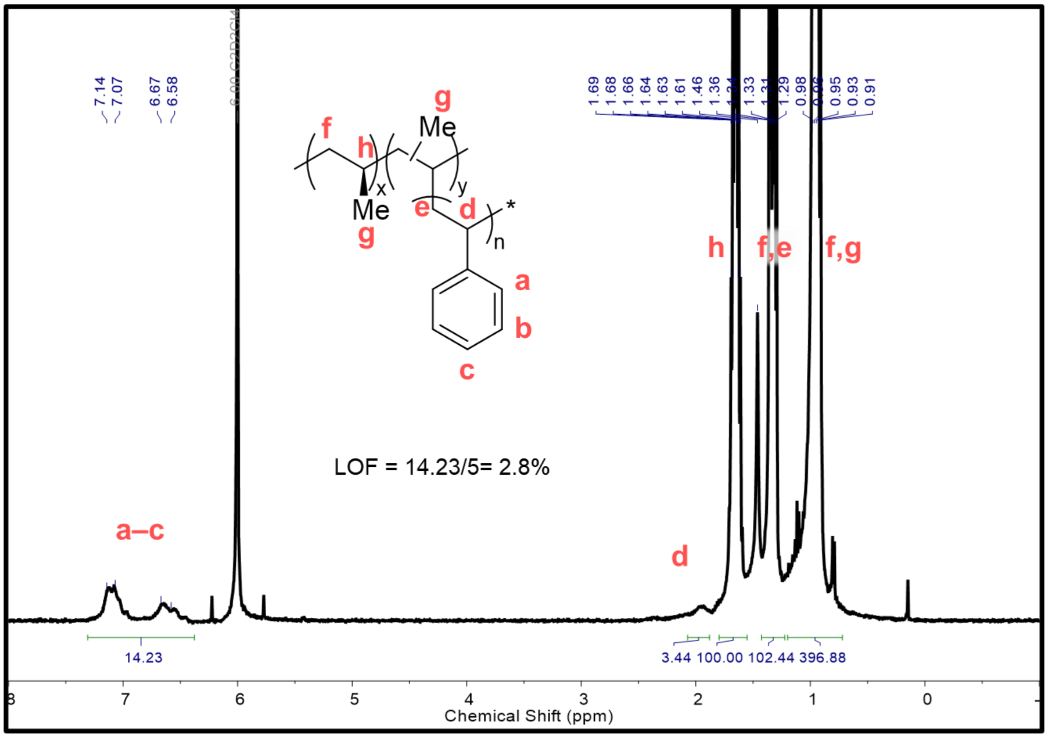

Figure S181 ^{1}H NMR spectrum (400 MHz, $C_2D_2Cl_4$) of *i*PP–*g*–$PS_{2.8}$ (T = 105 °C)

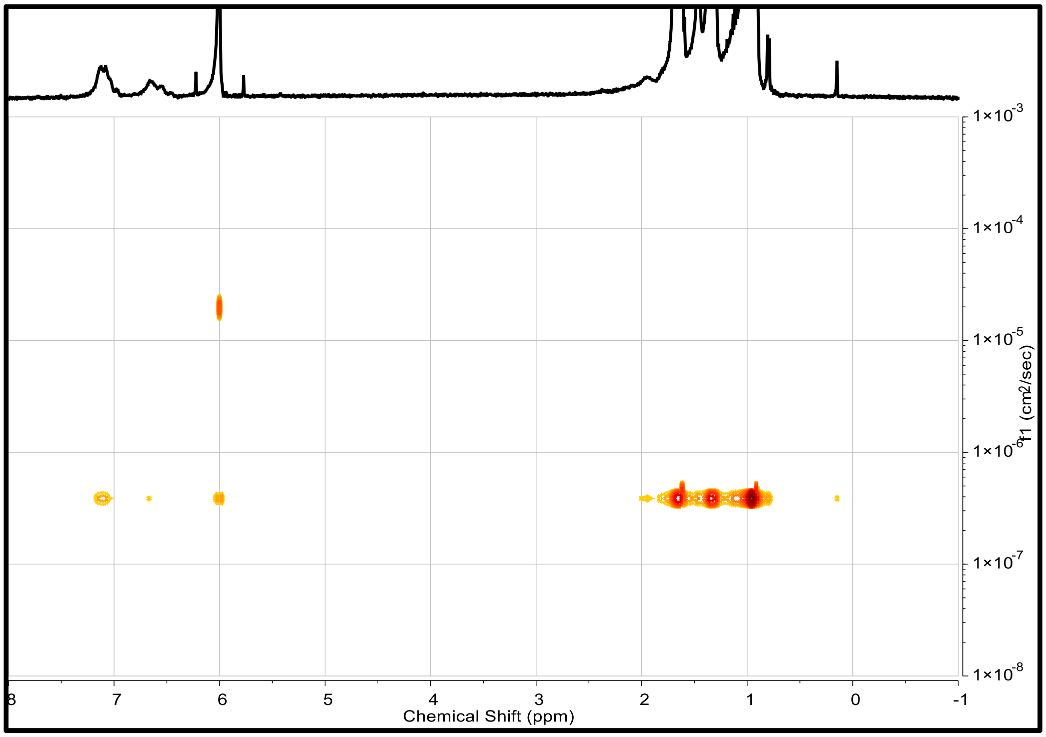

Figure S182. 2D DOSY NMR spectrum (400 MHz, $C_2D_2Cl_4$) of *i*PP–*g*–$PS_{2.8}$ (T = 105 °C)

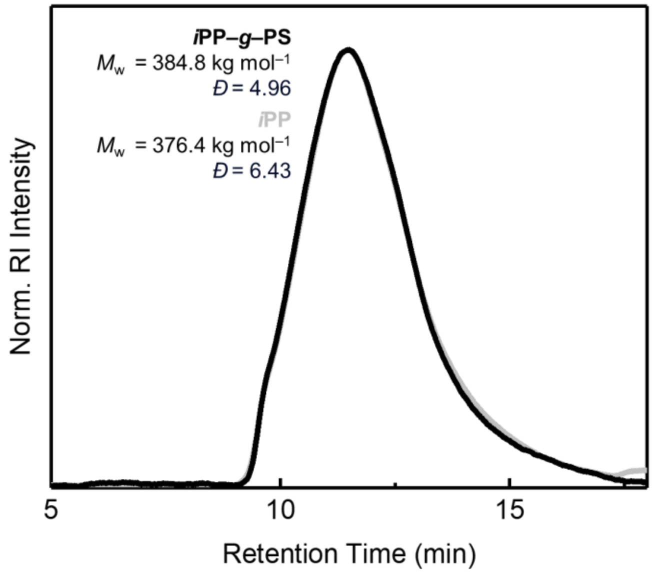

Figure S183. HT SEC trace of *i*PP–*g*–$PS_{2.8}$ (1,2,4-TCB, 1.0 mL min^{-1} at 150 °C)

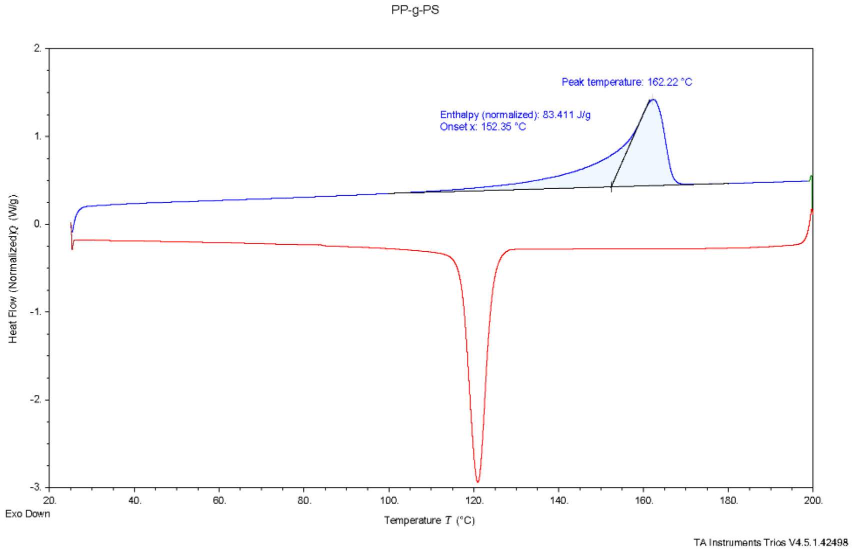

Figure S184. DSC Thermogram of *i*PP–*g*–PS (f_{vinyl} = 2.8%). T_m = 162.22 °C, ΔH_m = 83.411 J g^{-1}, X_C = 40.3%.

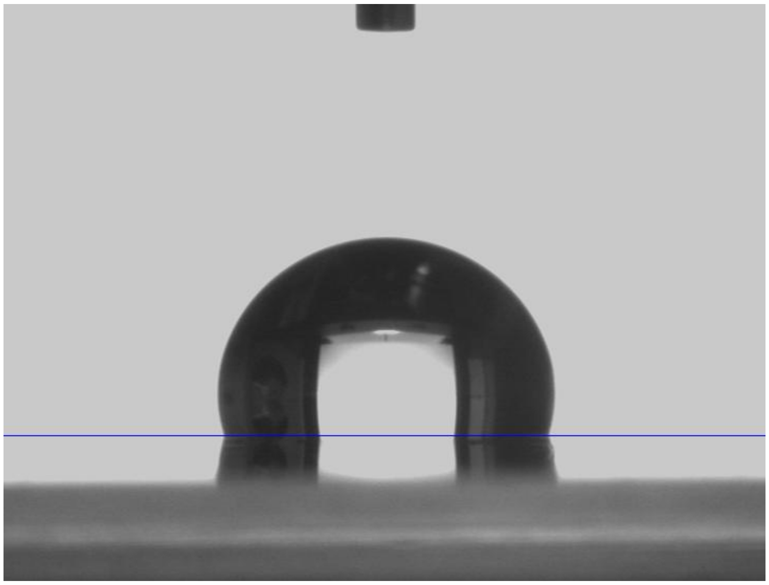

Figure S185. Water contact angle measured for *i*PP–*g*–PS (f_{vinyl} = 2.8%). WCA = 105.4°

Synthesis of Plastic Bag–*g*–PMA (Figure 5). A 22 mL borosilicate test tube was charged with cut pieces of a plastic bag (281 mg, 10 mmol) and 1,2-dichlorobenzene (10 mL), and sealed with a rubber septum. The mixture was degassed by nitrogen bubbling at 90 °C until it became homogeneous. Methyl acrylate (0.3 equiv., 0.27 mL, 3 mmol) was added via syringe. The reaction mixture was stirred and irradiated with a 390 nm LED lamp for 6 hours at 120 °C. Upon completion of the reaction, the mixture was precipitated in cold methanol (ca. 40 mL). The resulting solid was collected by filtration and washed via Soxhlet extraction with acetone for 3 h (ca. 4 min per cycle). After the purification, the solid was dried in a vacuum oven (3 mbar) at 80 °C for 12 h. to afford the desired **plastic bag–*g*–PMA** as a white solid (265 mg). The product was characterized by ^{1}H NMR (90 °C in $C_2D_2Cl_4$, 400 MHz), 2D DOSY NMR, DSC, and high temperature size exclusion chromatography. Isolated yield = 49%; Graft yield = 12.3%

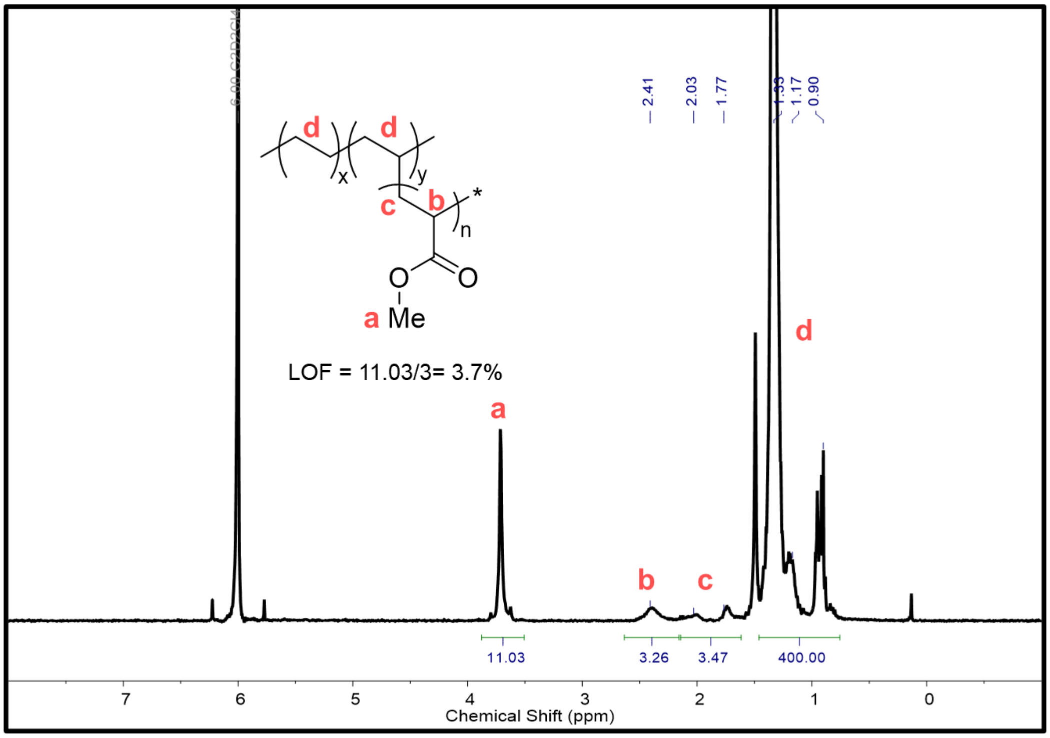

Figure S186. ^{1}H NMR spectrum (400 MHz, $C_2D_2Cl_4$) of Plastic bag–*g*–PMA$_{3.7}$ (T = 90 °C)

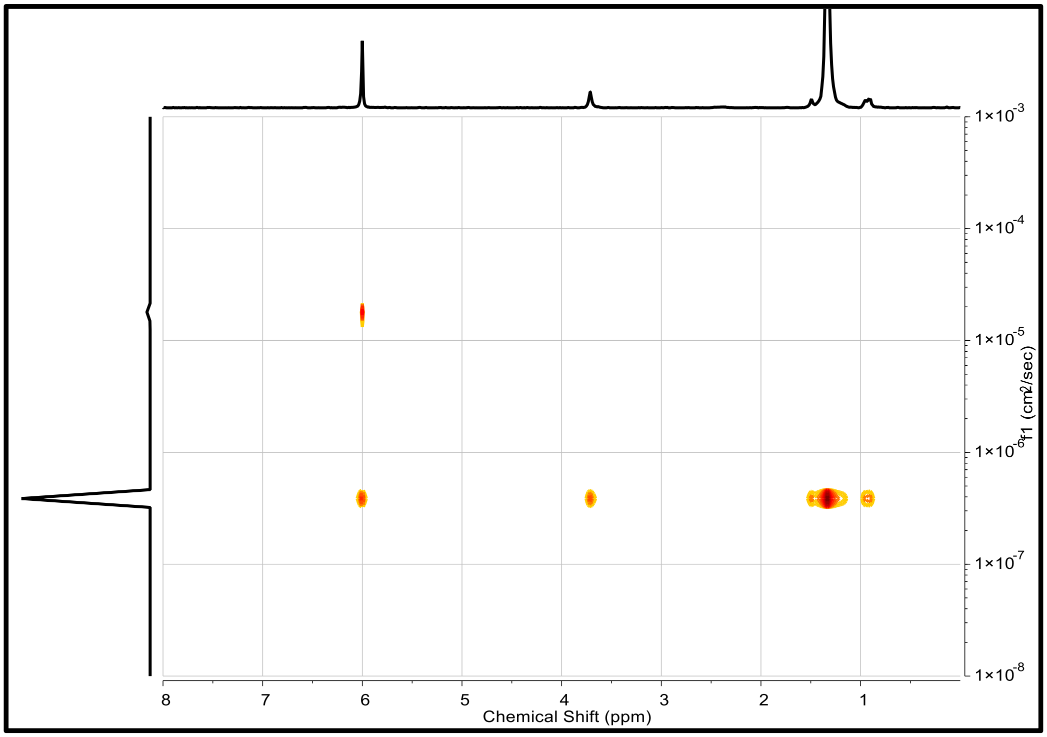

Figure S187. 2D DOSY NMR spectrum (400 MHz, $C_2D_2Cl_4$) of Plastic bag–*g*–$PMA_{3.7}$ (*T* = 90 °C)

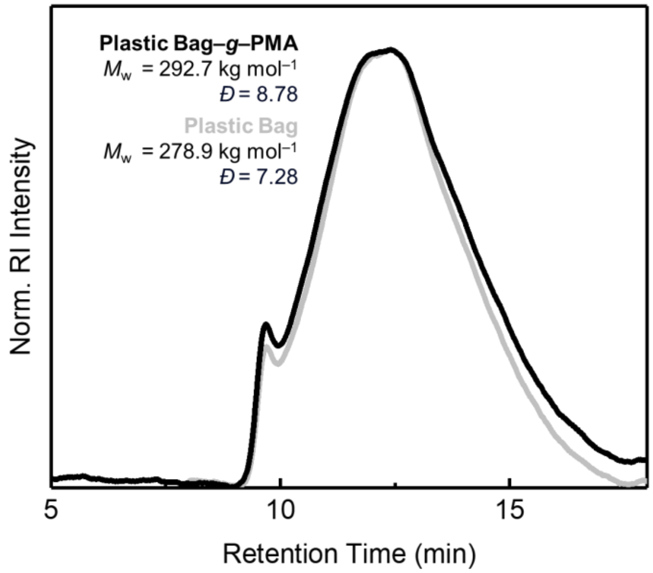

Figure S188. HT SEC trace of Plastic bag–*g*–PMA (1,2,4-TCB, 1.0 mL min^{-1} at 150 °C)

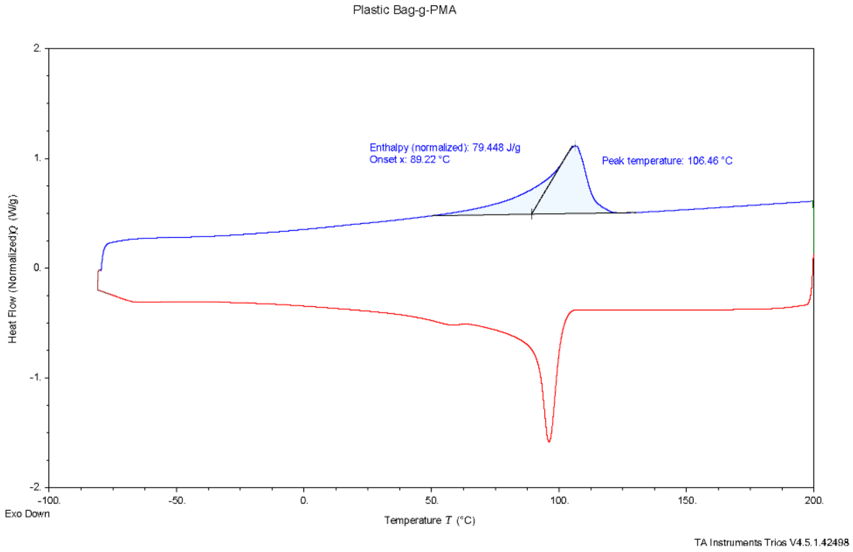

Figure S189. DSC Thermogram of Plastic bag–*g*–PMA (f_{vinyl} = 3.7%). T_m = 106.46 °C, ΔH_m = 79.448 J g^{-1}, X_C = 27.1%.

Synthesis of Plastic Bottle–*g*–PMA (Figure 5). A 22 mL borosilicate test tube was charged with cut pieces of a plastic bottle (281 mg, 10 mmol) and 1,2-dichlorobenzene (10 mL),and sealed with a rubber septum. The mixture was degassed by nitrogen bubbling at 120 °C until it became homogeneous. Methyl acrylate (0.3 equiv., 0.27 mL, 3 mmol) was added via syringe. The reaction mixture was stirred and irradiated with a 390 nm LED lamp for 6 hours at 120 °C. Upon completion of the reaction, the mixture was precipitated in cold methanol (ca. 40 mL). The resulting solid was collected by filtration and washed via Soxhlet extraction with acetone for 6 h (ca. 4 min per cycle). After the purification, the solid was dried in a vacuum oven (3 mbar) at 80 °C for 12 h. to afford the desired **HDPE–*g*–PMA** as a white solid (288 mg). The product was characterized by ^1H NMR (90 °C in $C_2D_2C288l_4$, 400 MHz), 2D DOSY NMR, DSC, and high temperature size exclusion chromatography. Isolated yield = 53%; Graft yield = 8.7%

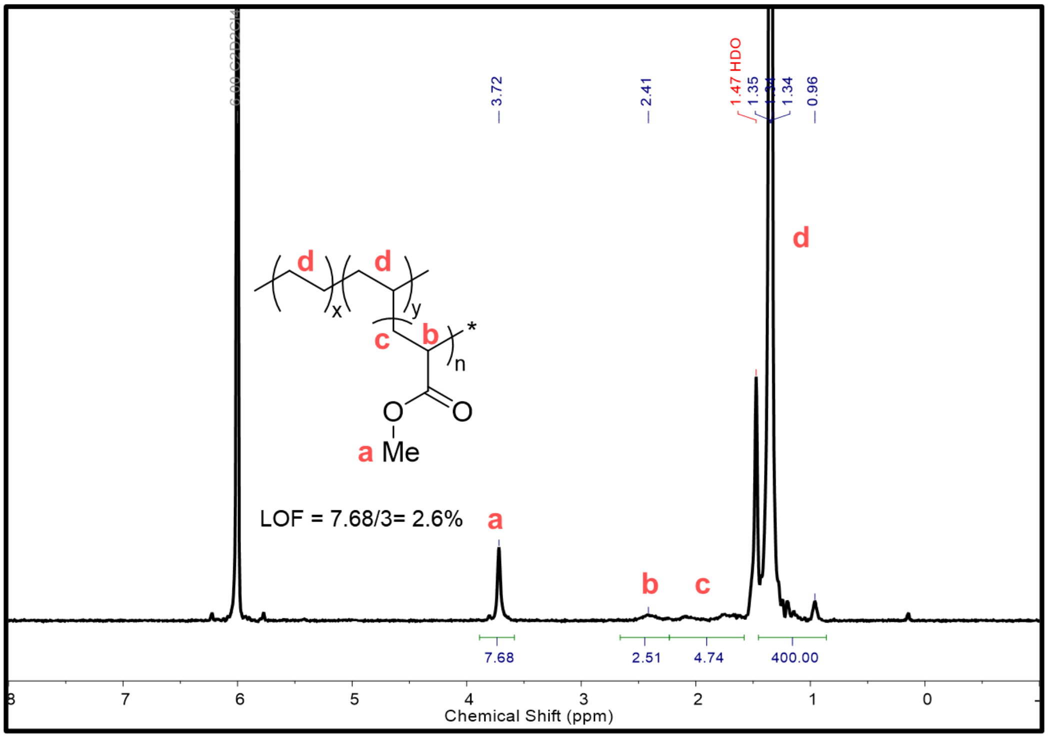

Figure S190. ^1H NMR spectrum (400 MHz, $C_2D_2Cl_4$) of Plastic bottle–*g*–$PMA_{2.6}$ (T = 105 °C)

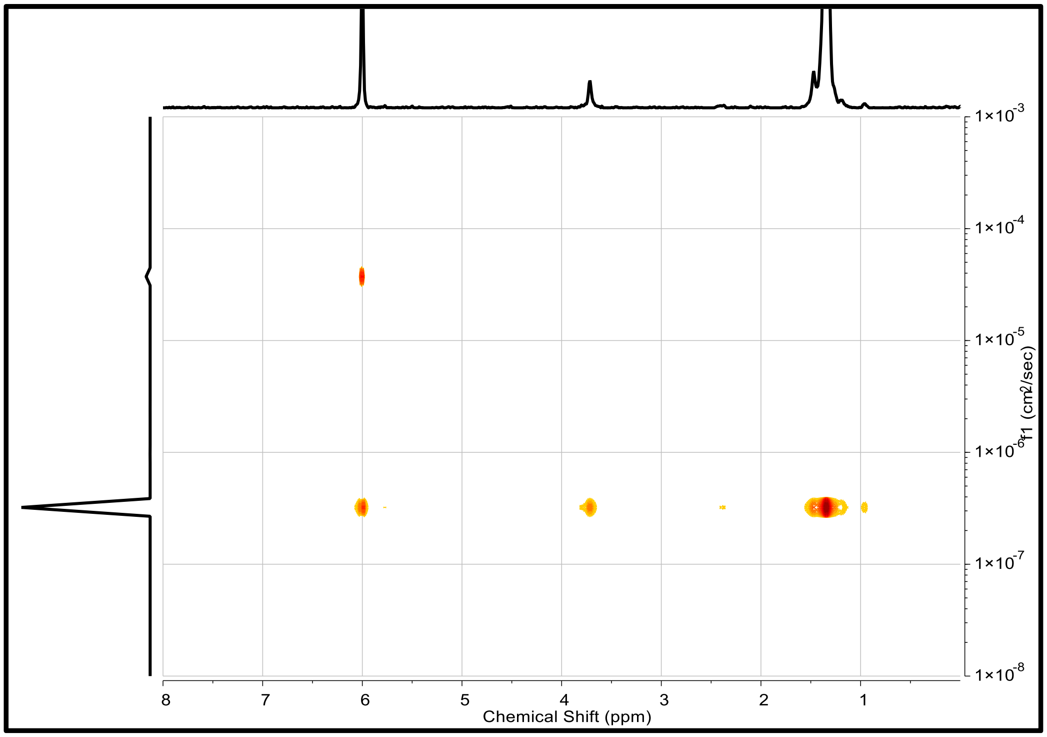

Figure S191. 2D DOSY NMR spectrum (400 MHz, $C_2D_2Cl_4$) of Plastic bottle–*g*–$PMA_{2.6}$ (T = 105 °C)

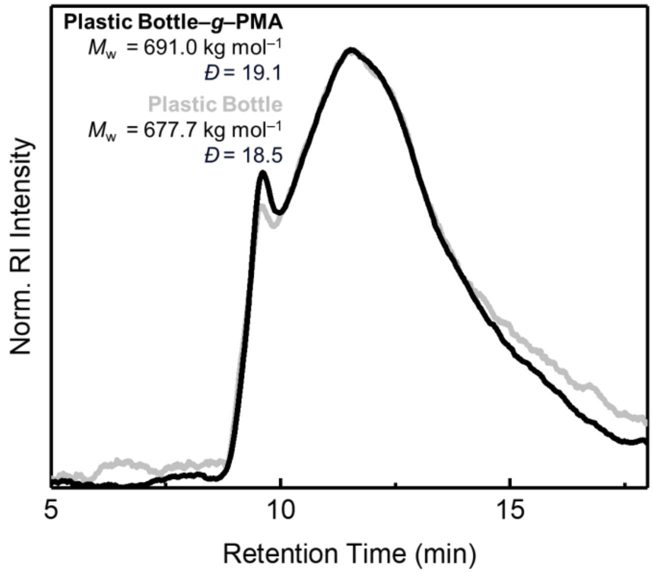

Figure S192. HT SEC trace of Plastic bottle–*g*–PMA (1,2,4-TCB, 1.0 mL min^{-1} at 150 °C)

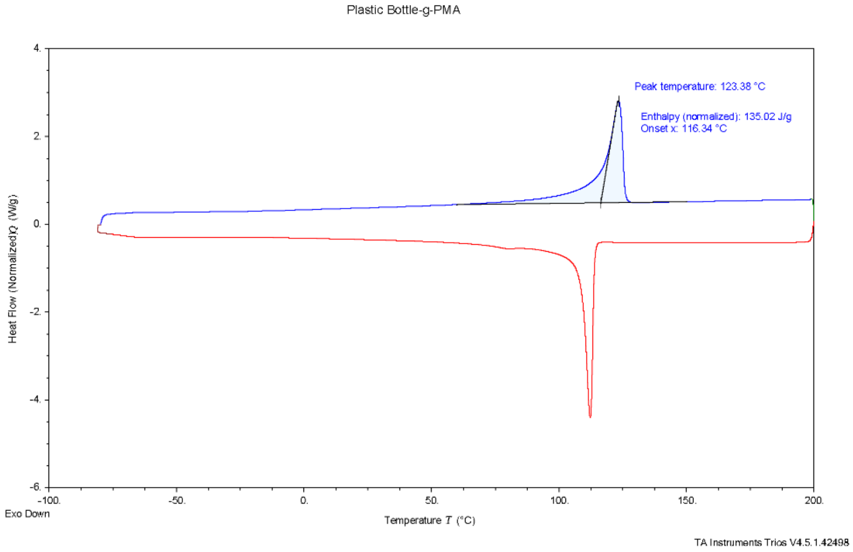

Figure S193. DSC Thermogram of Plastic bottle–*g*–PMA (f_{vinyl} = 2.6%). T_m = 123.38 °C, ΔH_m = 135.02 J g^{-1}, X_C = 46.1%.

Synthesis of Falcon Tube–*g*–PMA (Figure 5). A 22 mL borosilicate test tube was charged with cut pieces of a Falcon tube (281 mg, 6.7 mmol) and 1,2-dichlorobenzene (10 mL),and sealed with a rubber septum. The mixture was degassed by nitrogen bubbling at 120 °C until it became homogeneous. Methyl acrylate (0.45 equiv., 0.27 mL, 3 mmol) was added via syringe. The reaction mixture was stirred and irradiated with a 390 nm LED lamp for 6 hours at 120 °C. Upon completion of the reaction, the mixture was precipitated in cold methanol (ca. 40 mL). The resulting solid was collected by filtration and washed via Soxhlet extraction with acetone for 3 h (ca. 4 min per cycle). After the purification, the solid was dried in a vacuum oven (3 mbar) at 80 °C for 12 h. to afford the desired **Falcon Tube–*g*–PMA** as a white solid (268 mg). The product was characterized by ^{1}H NMR (90 °C in $C_2D_2Cl_4$, 400 MHz), 2D DOSY NMR, DSC, and high temperature size exclusion chromatography. Isolated yield = 50%; Graft yield = 3.8%

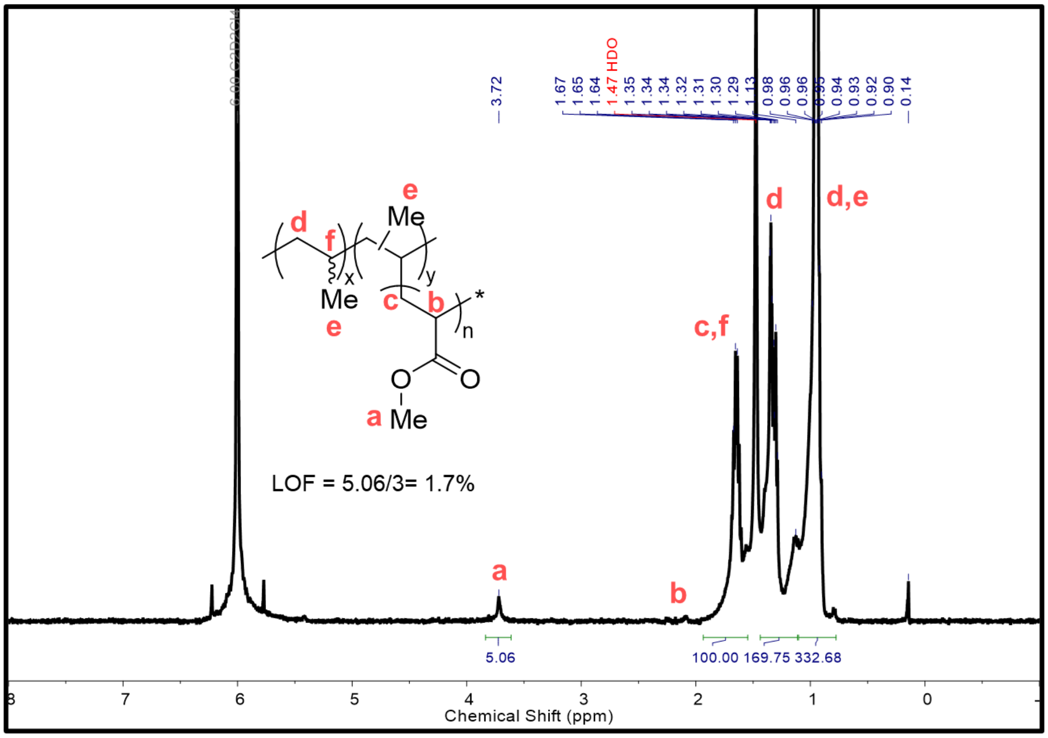

Figure S194. ^{1}H NMR spectrum (400 MHz, $C_2D_2Cl_4$) of Falcon tube–*g*–$PMA_{1.7}$ (T = 105 °C)

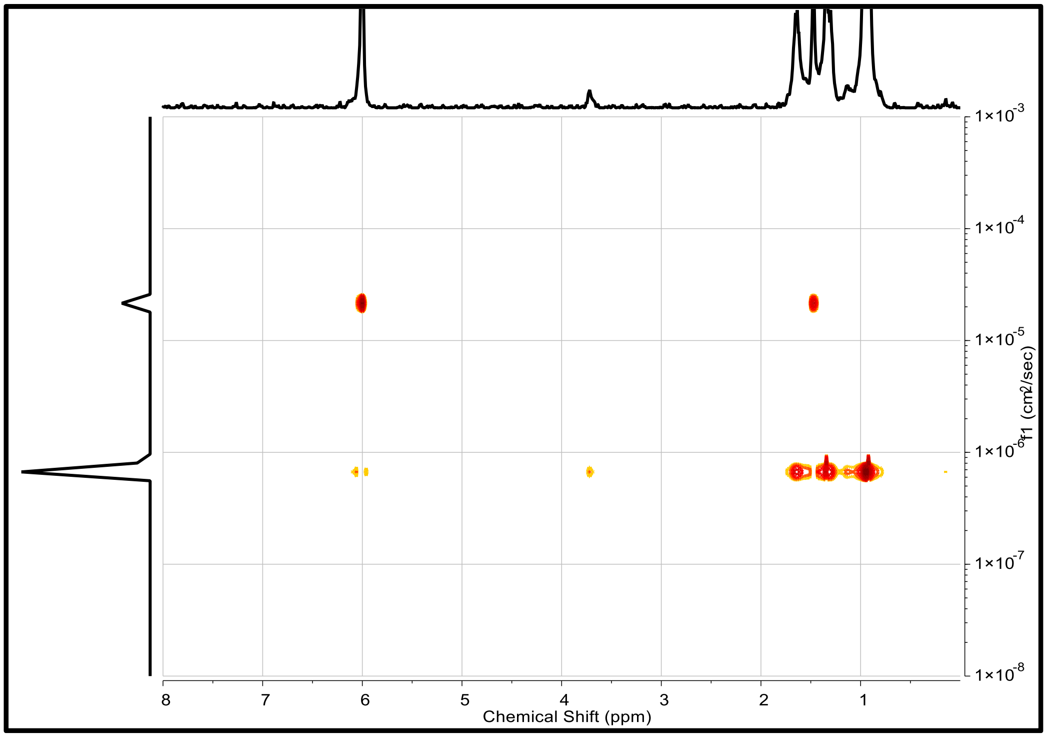

Figure S195. 2D DOSY NMR spectrum (400 MHz, $C_2D_2Cl_4$) of Falcon tube–*g*–$PMA_{1.7}$ (T = 105 °C)

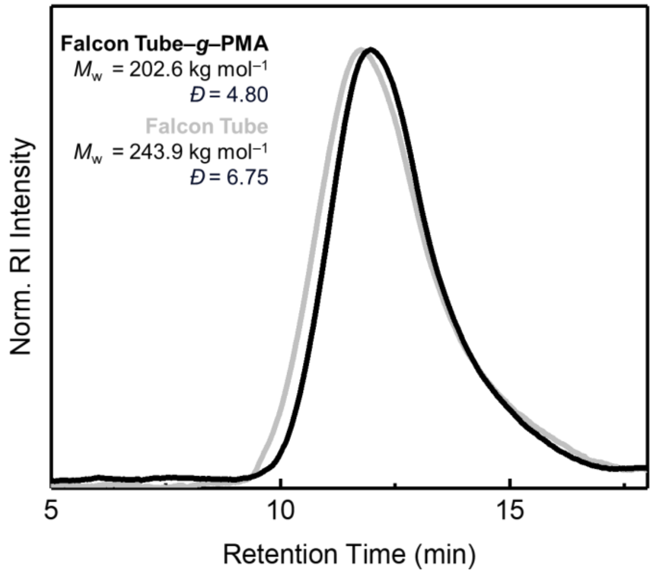

Figure S196. HT SEC trace of Falcon tube–*g*–PMA (1,2,4-TCB, 1.0 mL min^{-1} at 150 °C)

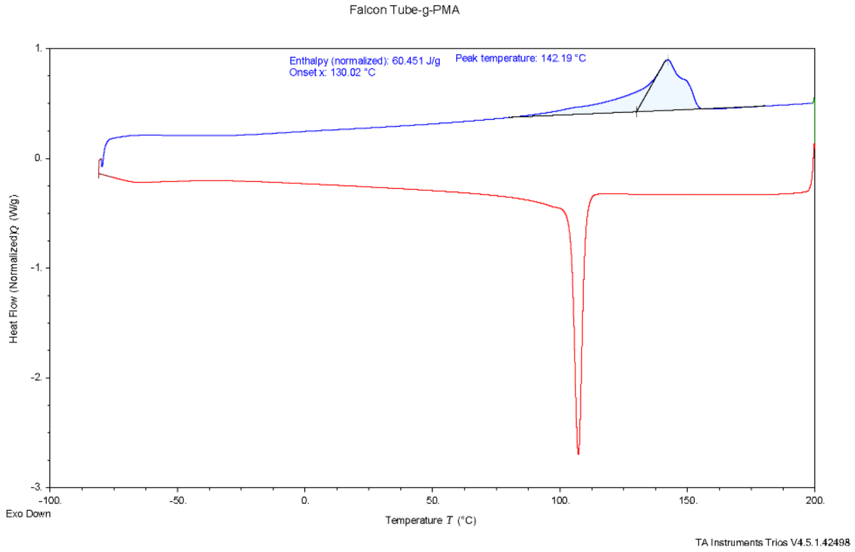

Figure S197. DSC Thermogram of Falcon tube–*g*–PMA (f_{vinyl} = 1.7%). T_m = 142.19 °C, ΔH_m = 60.451 J g^{-1}, X_C = 29.2%.

Synthesis of LDPE–*g*–PNEtAM (Fig. 5). A 500 mL borosilicate round-bottom flask was charged with LDPE (11.2 mg, 400 mmol) and 1,2-dichlorobenzene (200 mL), and sealed with a rubber septum. The mixture was degassed by nitrogen bubbling at 120 °C until it became homogeneous. *N*-ethyl acrylamide (0.2 equiv., 8.0 mL, 80 mmol) was added via syringe. The reaction mixture was stirred and irradiated with a 390 nm LED lamp for 2 hours at 120 °C. Upon completion of the reaction, the mixture was precipitated in cold methanol (ca. 40 mL). The resulting solid was collected by filtration and washed via Soxhlet extraction with isopropanol for 16 h (ca. 6 min per cycle). After the purification, the solid was dried in a vacuum oven (3 mbar) at 80 °C for 12 h. to afford the desired **LDPE–*g*–PNEtAM** as a white solid (13.5 g). The product was characterized by ^{1}H NMR (90 °C in $C_2D_2Cl_4$, 400 MHz), 2D DOSY NMR. Isolated yield = 71%; Graft yield = 34%

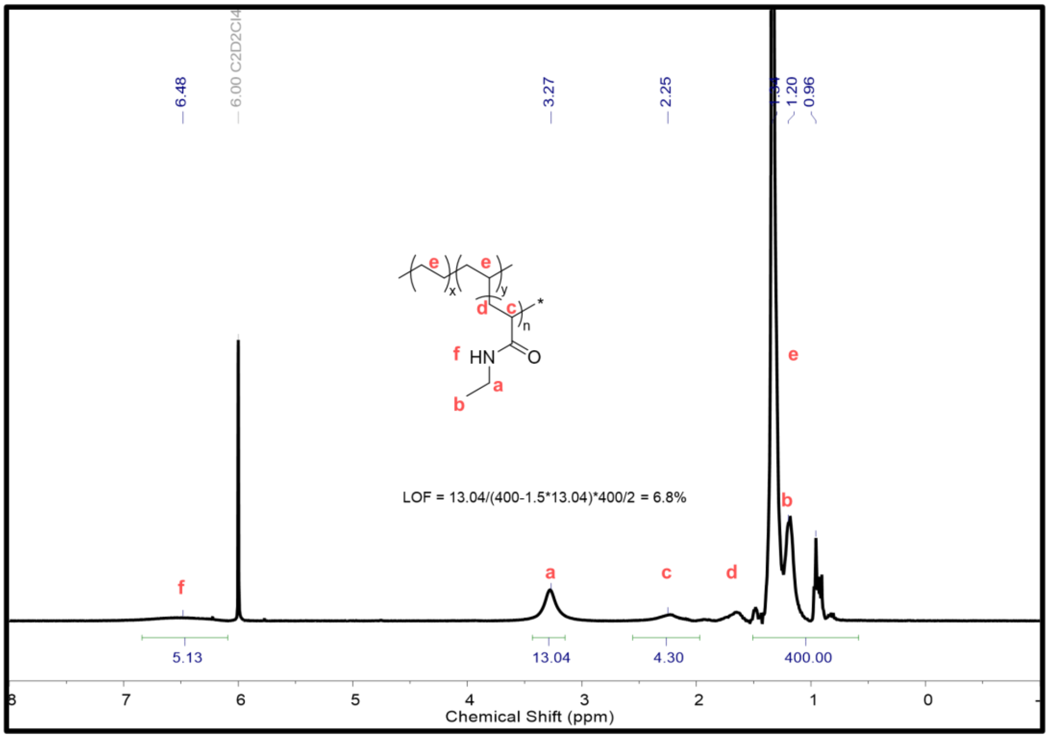

Figure S198. ^{1}H NMR spectrum (400 MHz, $C_2D_2Cl_4$) of LDPE–*g*–PNEtAM$_{6.8}$ (T = 90 °C)

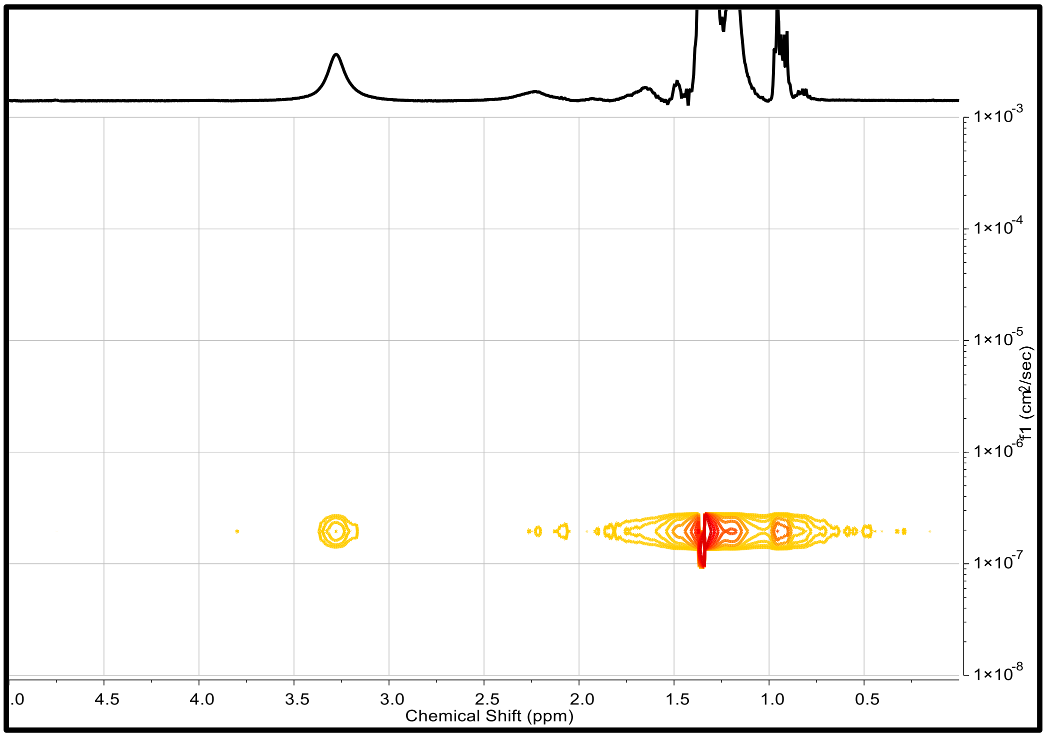

Figure S199. 2D DOSY NMR spectrum (400 MHz, $C_2D_2Cl_4$) of LDPE–*g*–PNEtAM$_{6.8}$ (T = 90 °C)

References

(1) Gao, G.; Hara, M.; Seki, T.; Takeoka, Y. Synthesis of Thermo-Responsive Polymer Gels Composed of Star-Shaped Block Copolymers by Copper-Catalyzed Living Radical Polymerization and Click Reaction. *Science and Technology of Advanced Materials* **2024**, *25* (1), 2302795. https://doi.org/10.1080/14686996.2024.2302795.
(2) Lee, J.; Ku, K. H.; Kim, M.; Shin, J. M.; Han, J.; Park, C. H.; Yi, G.-R.; Jang, S. G.; Kim, B. J. Stimuli-Responsive, Shape-Transforming Nanostructured Particles. *Advanced Materials* **2017**, *29* (29), 1700608. https://doi.org/10.1002/adma.201700608.
(3) Morrison, P. *The Merck Index: An Encyclopedia of Chemicals, Drugs, and Biologicals*; JSTOR, 1990.
(4) Owens, D. K.; Wendt, R. C. Estimation of the Surface Free Energy of Polymers. *Journal of Applied Polymer Science* **1969**, *13* (8), 1741–1747. https://doi.org/10.1002/app.1969.070130815.
(5) Kaelble, D. H. Dispersion-Polar Surface Tension Properties of Organic Solids. *The Journal of Adhesion* **1970**, *2* (2), 66–81. https://doi.org/10.1080/0021846708544582.
